# Supplementary material for: A Multicomponent Protocol for the Synthesis of Highly Functionalized γ-Lactam Derivatives and Their Applications as Antiproliferative Agents
Source: Pharmaceuticals (Basel). 2021 Aug 9;14(8):782. doi: 10.3390/ph14080782 (PMC8400033; doi:10.3390/ph14080782)
Supplement: Supplementary file 1 [file pharmaceuticals-14-00782-s001.zip › pharmaceuticals-1318653-supplementary.pdf]

pharmaceuticals

A multicomponent protocol for the synthesis of highly functionalized γ-lactam derivatives and their applications as antiproliferative agents.

Xabier del Corte[1], Adrián López-Francés[1], Aitor Maestro[1], Ilia Villate-Beitia[2,3,4], Myriam Sainz-Ramos[2,3,4], Edorta Martínez de Marigorta[1], José Luis Pedraz[2,3,4,*], Francisco Palacios[1,*] and Javier Vicario[1,*]

1 [1]*Departamento de Química Orgánica I, Centro de Investigación y Estudios Avanzados "Lucio Lascaray"- Facultad de Farmacia, University of the Basque Country, UPV/EHU Paseo de la Universidad 7, 01006 Vitoria-Gasteiz, SPAIN.*
2 *NanoBioCel Group, University of the Basque Country (UPV/EHU), Vitoria-Gasteiz, Spain*
3 *Biomedical Research Networking Center in Bioengineering, Biomaterials and Nanomedicine (CIBER-BBN), Spain.*
4 *Bioaraba, NanoBioCel Research Group, Vitoria-Gasteiz, Spain.*Affiliation 1; e-mail@e-mail.com
* *javier.vicario@ehu.eus (JV)* / *francisco.palacios@ehu.eus* (FP) / joseluis.pedraz@ehu.eus (JLP)

Supporting Information

Table of contents

1. Experimental procedures and characterization data for compounds **4-12** and **16-21** **S2**
2. ^{1}H NMR, ^{13}C NMR, ^{31}P NMR and ^{19}F NMR spectra of compounds **4-12** and **16-21** **S13**
3. HPLC chromatograms of compounds **4-12** and **16-21** **S125**
4. Flow cytometric assays on A-549 cells **S150**
5. Visualization of cell growth and morphology of A-549 cells **S153**
6. Calculation of Lipinski's rule of five and prediction of ADME properties. **S156**

1. Experimental procedures and characterization data for compounds 4-12 and 16-21.

General procedure for the synthesis of 3-amino-1,5-dihydro-1*H*-pyrrol-2-ones **4-12**.

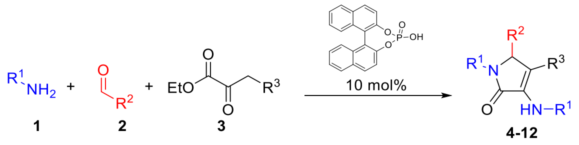

A solution of amine **1** (4 mmol), aldehyde **2** (2 mmol), ethyl pyruvate derivative **3** (6 mmol), BINOL derived phosphoric acid (70 mg, 0.2 mmol), in the presence of anhydrous $MgSO_4$, was stirred in diethy ether or MTBE (10 mL) at room temperature or 55°C for 48 h. The volatiles were distilled off at reduced pressure and the crude residue was purified by crystallization in diethyl ether or by column chromatography (Hexanes /AcOEt) to afford pure lactams **4-12**.

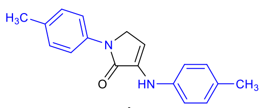

4a

1-(p-Tolyl)-3-(p-tolylamino)-1,5-dihydro-2H-pyrrol-2-one (***4a***). The general procedure was followed in diethyl ether at room temperature, using *p*-toluidine (429 mg, 4 mmol), 37 % aq. formaldehyde (150 µL, 2 mmol) and ethyl pyruvate (670 µL, 6 mmol). The residue was purified by column chromatography (Hexanes /AcOEt 9:1) affording 433 mg (78%) of **4a** as an orange solid. M.p. (Et_2O) = 178-180 °C. ^1H NMR (400 MHz, $CDCl_3$): δ 7.64 (d, $^3J_{HH}$ = 8.5 Hz, 2H), 7.20 (d, $^3J_{HH}$ = 8.2 Hz, 2H), 7.13 (d, $^3J_{HH}$ = 8.2 Hz, 2H), 7.1 (d, $^3J_{HH}$ = 8.5 Hz, 2H), 6.53 (s, 1H), 5.97 (t, $^3J_{HH}$ = 2.6 Hz, 1H), 4.37 (d, $^3J_{HH}$ = 2.6 Hz, 2H), 2.34 (s, 3H), 2.32 (s, 3H). ^{13}C {^1H} NMR (101 MHz, $CDCl_3$): δ 166.5 (C=O), 139.2 (C_{quat}), 136.8 (C_{quat}), 134.5 (C_{quat}), 134.3 (C_{quat}), 130.7 (=C_{quat}), 130.0 (2xCH), 129.8 (2xCH), 119.0 (2xCH), 116.8 (2xCH), 99.8 (CH), 49.8 (CH_2), 21.0 (CH_3), 20.8 (CH_3). FTIR (neat) ν_{max} : 3325 (N-H), 1671 (C=O), 1644 (C=CH). HRMS (ESI-TOF) m/z calcd for $C_{18}H_{18}N_2O$ $[M+H]^+$ 279.1497, found 279,1501.

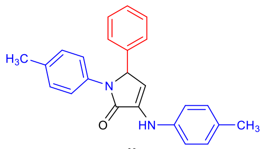

4b

5-Phenyl-1-(p-tolyl)-3-(p-tolylamino)-1H-pyrrol-2(5H)-one (***4b***). The general procedure was followed in diethyl ether at room temperature using *p*-toluidine (429 mg, 4 mmol), benzaldehyde (204 µL, 2 mmol) and ethyl pyruvate (670 µL, 6 mmol). The residue was crystallized in diethyl ether affording 638 mg (90%) of **4b** as a white solid. Physical and spectroscopic data are in agreement with literature data.[1]

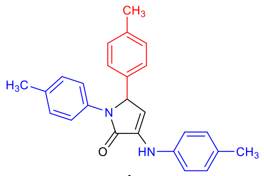

4c

1,5-Di-p-tolyl-3-(p-tolylamino)-1,5-dihydro-2H-pyrrol-2-one (***4c***). The general procedure was followed in diethyl ether at room temperature using *p*-toluidine (429 mg, 4 mmol), *p*-tolualdehyde (236 µL, 2 mmol) and ethyl pyruvate (670 µL, 6 mmol). The residue was crystallized in diethyl ether affording 648 mg (88%) of **4c** as an orange solid. M.p. (Et_2O) = 210-213. ^1H NMR (400 MHz, $CDCl_3$) δ 7.39 (d, $^3J_{HH}$ = 8.5 Hz, 2H), 7.11-7.06 (m, 8H), 6.97 (d, $^3J_{HH}$ = 8.4 Hz, 2H), 6.56 (bs, 1H), 5.99 (d, $^3J_{HH}$ = 2.5 Hz, 1H), 5.60 (d, $^3J_{HH}$ = 2.5 Hz, 1H), 2.29 (s, 3H), 2.28 (s, 3H), 2.26 (s, 3H) . ^{13}C {^1H} NMR (101 MHz, $CDCl_3$): δ 167.34 (C=O), 139.02 (C_{quat}), 138.0 (C_{quat}), 134.80 (C_{quat}), 134.70 (C_{quat}), 134.60 (C_{quat}), 132.4 (C_{quat}), 130.7 (C_{quat}), 129.9 (2xCH), 129.7 (2xCH), 129.6 (2xCH), 126.9 (2xCH), 121.9 (2xCH), 116.9 (2xCH), 107.7 (CH), 64.2 (CH), 21.3 (CH_3), 21.0 (CH_3), 20.8 (CH_3). FTIR (neat) ν_{max} : 3316 (N-H), 1666 (C=O), 1648 (C=CH) . HRMS (ESI-TOF) m/z calcd for $C_{25}H_{25}N_2O$ $[M+H]^+$ 369,1967, found 369,1952.

[1] del Corte, X.; Maestro, A.; Vicario, J.; Martínez de Marigorta, E.; Palacios. F. Brönsted-Acid-Catalyzed Asymmetric Three-Component Reaction of Amines, Aldehydes, and Pyruvate Derivatives. Enantioselective Synthesis of Highly Functionalized γ-Lactam Derivatives. *Org. Lett.* **2018**, *20*, 317-320. DOI: 10.1021/acs.orglett.7b03397.

*5-(m-Tolyl)-1-(p-tolyl)-3-(p-tolylamino)-1,5-dihydro-2H-pyrrol-2-one (**4d**).* The general procedure was followed in MTBE at 55°C (heating plate, Heat-On) using *p*-toluidine (429 mg, 4 mmol), *m*-tolualdehyde (235 µL, 2 mmol) and ethyl pyruvate (670 µL, 6 mmol). The residue was crystallized in diethyl ether affording 661 mg (90%) of **4d** as a white solid. Physical and spectroscopic data are in agreement with literature data.[1]

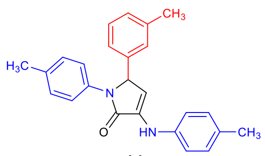

4d

*5-(o-Tolyl)-1-(p-tolyl)-3-(p-tolylamino)-1,5-dihydro-2H-pyrrol-2-one (**4e**).* The general procedure was followed in MTBE at 55°C (heating plate, Heat-On) using *p*-toluidine (429 mg, 4 mmol), *o*-tolualdehyde (231 µL, 2 mmol) and ethyl pyruvate (670 µL, 6 mmol). The residue was crystallized in diethyl ether affording 457 mg (62%) of **4e** as an orange solid. M.p. (Et_2O) = 198 (dec.). ^1H NMR (400 MHz, $CDCl_3$) δ 7.39 (d, $^3J_{HH}$ = 8.5 Hz, 2H), 7.14-7.11 (m, 2H), 7.11-7.07 (m, 5H), 7.03-7.00 (m, 1H), 6.97 (d, $^3J_{HH}$ = 8.5 Hz, 2H), 6.57 (bs, 1H), 5.99 (d, $^3J_{HH}$ = 2.6 Hz, 1H), 5.89 (d, $^3J_{HH}$ = 2.6 Hz, 1H), 2.45 (s, 3H), 2.29 (s, 3H), 2.26 (s, 3H). ^{13}C {^1H} NMR (101 MHz, $CDCl_3$): δ 167.4 (C=O), 139.0 (C_{quat}), 135.5 (C_{quat}), 135.4 (C_{quat}), 135.1 (C_{quat}), 134.6 (C_{quat}), 132.8 (C_{quat}), 131.1 (C_{quat}), 130.8 (2xCH, overlaped), 129.9 (2xCH), 129.6 (2xCH), 127.8 (CH), 127.0 (CH), 121.2 (2xCH), 116.9 (2xCH), 106.1 (CH), 61.4 (CH), 21.0 (CH_3), 20.8 (CH_3), 19.8 (CH_3). FTIR (neat) ν_{max} : 3310 (N-H), 1687 (C=O), 1645 (C=CH) . HRMS (ESI-TOF) m/z calcd for $C_{25}H_{25}N_2O$ $[M+H]^+$ 369,1967, found 369,1953.

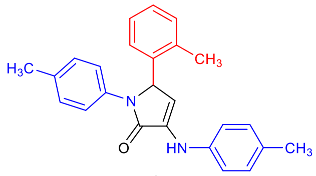

4e

*5-(p-Fluorophenyl)-1-(p-tolyl)-3-(p-tolylamino)-1,5-dihydro-2H-pyrrol-2-one (**4f**).* The general procedure was followed in diethyl ether at room temperature using *p*-toluidine (429 mg, 4 mmol), *p*-fluoroaldehyde (214 µL, 2 mmol) and ethyl pyruvate (670 µL, 6 mmol). The residue was crystallized in diethyl ether affording 599 mg (81%) of **4f** as a white solid. Physical and spectroscopic data are in agreement with literature data.[1]

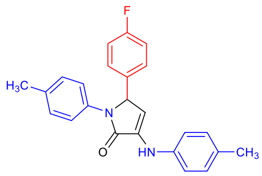

4f

*5-(p-Nitrophenyl)-1-(p-tolyl)-3-(p-tolylamino)-1H-pyrrol-2(5H)-one (**4g**).* The general procedure was followed in diethyl ether at room temperature using *p*-toluidine (429 mg, 4 mmol), *p*-nitrobenzaldehyde (302 mg, 2 mmol) and ethyl pyruvate (670 µL, 6 mmol). The residue was crystallized in diethyl ether affording 637 mg (80%) of **4g** as a yellow solid. Physical and spectroscopic data are in agreement with literature data.[1]

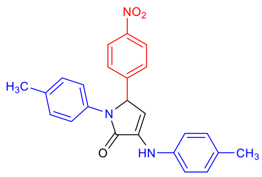

4g

*5-(m-Nitrophenyl)-1-(p-tolyl)-3-(p-tolylamino)-1,5-dihydro-2H-pyrrol-2-one (**4h**).* The general procedure was followed in diethyl ether at room temperature using *p*-toluidine (429 mg, 4 mmol), *m*-nitrobenzaldehyde (302 mg, 2 mmol) and ethyl pyruvate (670 µL, 6 mmol). The residue was crystallized in diethyl ether affording 580 mg (73%) of **4h** as a yellow solid. Physical and spectroscopic data are in agreement with literature data.[1]

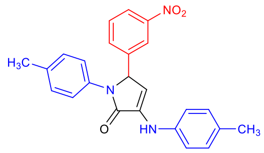

4h

*1-(p-Tolyl)-3-(p-tolylamino)-5-(p-(trifluoromethyl)phenyl)-1,5-dihydro-2H-pyrrol-2-one (**4i**).* The general procedure was followed in diethyl ether at room temperature using *p*-toluidine (429 mg, 4 mmol), *p*-(trifluoromethyl)benzaldehyde (273 µL, 2 mmol) and ethyl pyruvate (670 µL, 6 mmol). The residue was crystallized in diethyl ether affording 724 mg (86%) of **4i** as a white solid. Physical and spectroscopic data are in agreement with literature data.[1]

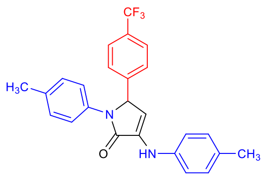

4i

5-(1-Methyl-1H-indol-6-yl)-1-(p-tolyl)-3-(p-tolylamino)-1,5-dihydro-2H-pyrrol-2-one (***4j***). The general procedure was followed in diethyl ether at room temperature using *p*-toluidine (429 mg, 4 mmol), 1-methyl-1H-indole-6-carboxaldehyde (318 mg, 2 mmol) and ethyl pyruvate (670 μL, 6 mmol). The residue was purified by column chromatography (Hexanes /AcOEt 9:1) affording 494 mg (61%) of **4j** as an orange solid. M.p. (Et_2O) = 185 (dec.). ^{1}H NMR (300 MHz, $CDCl_3$) δ 7.52 (d, $^3J_{HH}$ = 8.1 Hz, 1H), 7.44 (d, $^3J_{HH}$ = 8.5 Hz, 2H), 7.14 – 6.95 (m, 9H), 6.58 (bs, 1H), 6.41 (d, $^3J_{HH}$ = 2.8 Hz, 1H), 6.07 (d, $^3J_{HH}$ = 2.6 Hz, 1H), 5.75 (d, $^3J_{HH}$ = 2.6 Hz, 1H), 3.72 (s, 3H), 2.28 (s, 3H), 2.23 (s, 3H). . ^{13}C {^{1}H} NMR (75 MHz, $CDCl_3$): δ 167.6 (C=O), 139.2 (C_{quat}), 137.1 (C_{quat}), 135.2 (C_{quat}), 134.7 (C_{quat}), 132.1 (C_{quat}), 131.1 (C_{quat}), 130.7 (C_{quat}), 130.0 (2xCH), 129.6 (2xCH), 128.6 (C_{quat}), 127.3 (CH), 122.0 (2xCH), 121.5 (CH), 118.5 (CH), 116.9 (2xCH), 108.8 (CH), 107.7 (CH), 101.0 (CH), 65.4 (CH), 33.2 (CH_3), 21.1 (CH_3), 20.9 (CH_3) . FTIR (neat) ν_{max} : 3301 (N-H), 1672 (C=O), 1651 (C=CH) . HRMS (ESI-TOF) m/z calcd for $C_{27}H_{26}N_3O$ $[M+H]^+$ 408,2076, found 408,2071.

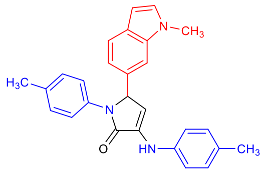

4j

5-(Furan-2-yl)-1-(p-tolyl)-3-(p-tolylamino)-1,5-dihydro-2H-pyrrol-2-one (***4k***). The general procedure was followed in diethyl ether at room temperature using *p*-toluidine (429 mg, 4 mmol), 2-furaldehyde (166 μL, 2 mmol) and ethyl pyruvate (670 μL, 6 mmol). The residue was purified by column chromatography (Hexanes /AcOEt 9:1) affording 392 mg (57%) of **4k** as a white solid. Physical and spectroscopic data are in agreement with literature data.[2]

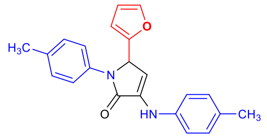

4k

5-(Thiophen-2-yl)-1-(p-tolyl)-3-(p-tolylamino)-1,5-dihydro-2H-pyrrol-2-one (***4l***). The general procedure was followed in MTBE at 55°C (heating plate, Heat-On) using *p*-toluidine (429 mg, 4 mmol), 2-thiophenecarboxaldehyde (187 μL, 2 mmol) and ethyl pyruvate (670 μL, 6 mmol). The residue was crystallized in diethyl ether affording 616 mg (86%) of **4l** as a yellow solid. Physical and spectroscopic data are in agreement with literature data.[1]

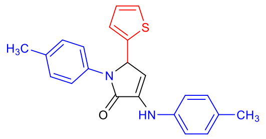

4l

5-(Naphthalen-2-yl)-1-(p-tolyl)-3-(p-tolylamino)-1,5-dihydro-2H-pyrrol-2-one (***4m***). The general procedure was followed in diethyl ether at room temperature using *p*-toluidine (429 mg, 4 mmol), 2-naphtaldehyde (312 mg, 2 mmol) and ethyl pyruvate (670 μL, 6 mmol). The residue was purified by column chromatography (Hexanes /AcOEt 9:1) affording 561 mg (70%) of **4m** as an orange solid. M.p. (Et_2O) = 189 (dec.). ^{1}H NMR (400 MHz, $CDCl_3$) δ 7.79-7.72 (m, 4H), 7.47-7.41 (m, 4H), 7.22 (dd, $^3J_{HH}$ = 8.5 Hz, $^3J_{HH}$ = 1.5 Hz, 1H), 7.09 (d, $^3J_{HH}$ = 8.3 Hz, 2H), 7.05 (d, $^3J_{HH}$ = 8.3 Hz, 2H), 6.99 (d, $^3J_{HH}$ = 8.5 Hz, 2H), 6.63 (bs, 1H), 6.05 (d, $^3J_{HH}$ = 2.6 Hz, 1H), 5.79 (d, $^3J_{HH}$ = 2.6 Hz, 1H), 2.28 (s, 3H), 2.22 (s, 3H) . ^{13}C {^{1}H} NMR (101 MHz, $CDCl_3$): δ 167.4 (C=O), 138.9 (C_{quat}), 135.3 (C_{quat}), 134.9 (C_{quat}), 134.8 (C_{quat}), 133.5 (C_{quat}), 133.3 (C_{quat}), 132.7 (C_{quat}), 130.8 (C_{quat}), 130.0 (2xCH), 129.6 (2xCH), 129.1 (CH), 127.9 (CH), 127.8 (CH), 126.7 (CH), 126.5 (CH), 126.3 (CH), 123.9 (CH), 122.0 (2xCH), 117.0 (2xCH), 107.3 (CH), 64.7 (CH), 21.0 (CH_3), 20.8 (CH_3). FTIR (neat) ν_{max} : 3316 (N-H), 1663 (C=O), 1648 (C=CH) . HRMS (ESI-TOF) m/z calcd for $C_{28}H_{25}N_2O$ $[M+H]^+$ 405,1967, found 405,1949.°

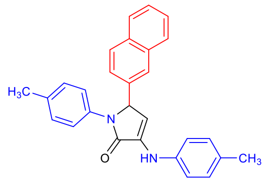

4m

5-Methyl-1-(p-tolyl)-3-(p-tolylamino)-1H-pyrrol-2(5H)-one (***4n***). The general procedure was followed in diethyl ether at room temperature using *p*-toluidine (429 mg, 4 mmol), acetaldehyde (112 μL, 2 mmol) and ethyl pyruvate (670 μL, 6 mmol). The residue was purified by column chromatography (Hexanes /AcOEt 9:1) affording 445 mg (78%) of **4n** as a white solid. Physical and spectroscopic data are in agreement with literature data.[2]

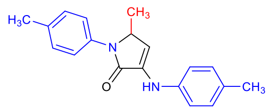

4n

[2] Palacios, F.; Vicario, J.; Aparicio, D. "An efficient synthesis of achiral and chiral cyclic dehydro-α-amino acid derivatives through nucleophilic addition of Amines to β,γ-unsaturated α-keto esters. *Eur. J. Org. Chem.* **2006**, 2843-2850. DOI: 10.1002/ejoc.200600092.

*5-Iso-propyl-1,3-di-p-tolyl-1H-pyrrol-2(5H)-one (**4o**).* The general procedure was followed in diethyl ether at room temperature using *p*-toluidine (429 mg, 4 mmol), acetaldehyde (112 µL, 2 mmol) and ethyl pyruvate (670 µL, 6 mmol). The residue was purified by column chromatography (Hexanes /AcOEt 85:15) affording 492 mg (77%) of **4o** as a white solid. Physical and spectroscopic data are in agreement with literature data.[1]

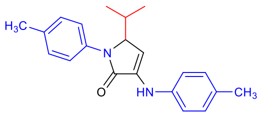

4o

*5-Iso-butyl-1-(p-tolyl)-3-(p-tolylamino)-1,5-dihydro-2H-pyrrol-2-one (**4p**).* The general procedure was followed in diethyl ether at room temperature using *p*-toluidine (429 mg, 4 mmol), isovaleraldehyde (214 µL, 2 mmol) and ethyl pyruvate (670 µL, 6 mmol). The residue was purified by column chromatography (Hexanes /AcOEt 9:1) affording 449 mg (67%) of **4p** as a yellow solid. M.p. (Et_2O) = 178-179 °C. ^{1}H NMR (400 MHz, $CDCl_3$) δ 7.36 (d, $^3J_{HH}$ = 8.4 Hz, 2H), 7.23 (d, $^3J_{HH}$ = 8.4 Hz, 2H), 7.13 (d, $^3J_{HH}$ = 8.4 Hz, 2H) 7.00 (d, $^3J_{HH}$ = 8.4 Hz, 2H), 6.49 (bs, 1H), 6.10 (d, $^3J_{HH}$ = 2.5 Hz, 1H), 4.74-4.78 (m, 1H), 2.37 (s, 3H), 2.31 (s, 3H), 1.77 (m, 1H), 1.25 (m, 2H), 1.00 (d, $^3J_{HH}$ = 6.5 Hz, 3H), 0.86 (d, $^3J_{HH}$ = 6.5 Hz, 3H) . ^{13}C {^{1}H} NMR (101 MHz, $CDCl_3$): δ 166.2 (C=O), 139.38 (C_{quat}), 135.2 (C_{quat}), 134.3 (C_{quat}), 133.5 (C_{quat}), 130.6 (C_{quat}), 130.0 (2xCH), 129.8 (2xCH), 123.0 (2xCH), 116.8 (2xCH), 105.6 (CH), 58.7 (CH), 41.8 (CH_2), 25.3 (CH), 24.1 (CH_3), 22.2 (CH_3), 21.1 (CH_3), 20.82 (CH_3) . FTIR (neat) ν_{max}: 3300 (N-H), 1675 (C=O), 1642 (C=CH) . HRMS (ESI-TOF) m/z calcd for $C_{22}H_{27}N_2O$ $[M+H]^+$ 335,2123, found 335,2124.

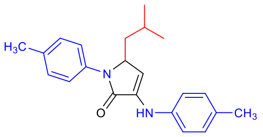

4p

*1-Cyclohexyl-5-(p-tolyl)-3-(p-tolylamino)-1,5-dihydro-2H-pyrrol-2-one (**4q**).* The general procedure was followed in diethyl ether at room temperature using *p*-toluidine (429 mg, 4 mmol), cyclohexanecarboxaldehyde (242 µL, 2 mmol) and ethyl pyruvate (670 µL, 6 mmol). The residue was crystallized in diethyl ether affording 655 mg (91%) of **4q** as a white solid. Physical and spectroscopic data are in agreement with literature data.[1]

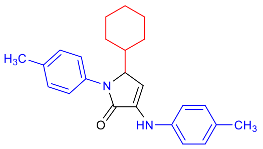

4q

*(E)-5-Styryl-1-(p-tolyl)-3-(p-tolylamino)-1,5-dihydro-2H-pyrrol-2-one (**4r**).* The general procedure was followed in diethyl ether at room temperature using *p*-toluidine (429 mg, 4 mmol), cinnamaldehyde (252 µL, 2 mmol) and ethyl pyruvate (670 µL, 6 mmol). The residue was purified by column chromatography (Hexanes /AcOEt 8:2) affording 448 mg (59%) of **4r** as a white solid. Physical and spectroscopic data are in agreement with literature data.[1]

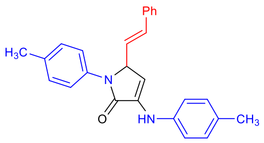

4r

*Ethyl 5-oxo-1, 4-di-p-tolyl-2,5-dihydro-1H-pyrrol-2-carboxylate (**4s**).* The general procedure was followed in diethyl ether at room temperature using *p*-toluidine (429 mg, 4 mmol), 50% solution in toluene (407 µL, 2 mmol) and ethyl pyruvate (670 µL, 6 mmol). The residue crystallized in diethyl ether affording 665 mg (95%) of **4s** as a white solid. Physical and spectroscopic data are in agreement with literature data.[1]

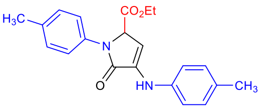

4s

*5-(Perfluorophenyl)-1-(p-tolyl-3-(p-tolylamino)-1H-pyrrol-2(5H)-one (**4t**).* The general procedure was followed in MTBE at 55°C (heating plate, Heat-On) using *p*-toluidine (429 mg, 4 mmol), 2,3,4,5,6-pentafluorobenzaldehyde (247 µL, 2 mmol) and ethyl pyruvate (670 µL, 6 mmol). The residue was purified by column chromatography (Hexanes /AcOEt 9:1) affording 731 mg (82%) of **4t** as a white solid. Physical and spectroscopic data are in agreement with literature data.[3]

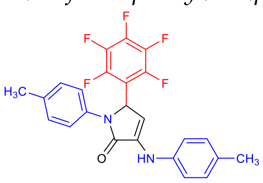

4t

[3] del Corte, X.; López-Francés, A.; Maestro, A.; Martinez de Marigorta, E.; Palacios, F.; Vicario. J. Brönsted Acid Catalyzed Multicomponent Synthesis of Phosphorated and Fluorinated γ-Lactam Derivatives. *J. Org. Chem.* **2020**, *85*, 14369-14383. DOI: 10.1021/acs.joc.0c00280.

1-(p-Tolyl)-3-(p-tolylamino)-5-(trifluoromethyl)-1,5-dihydro-2H-pyrrol-2-one (**4u**). A solution of *p*-toluidine (214 mg, 2 mmol), and 75 % aq. trifluoroacetaldehyde hydrate (107 μL, 1 mmol) in toluene (10 mL) was heated at 110 °C using a Dean-Stark on a heating plate (Heat-On) until water was fully removed. When the imine was completely formed and 2,2,2-trifluoroethane-1,1-diol was completely consumed (monitored by ^{19}F NMR: 2,2,2-trifluoroethane-1,1-diol: -85,5 ppm; imine: -79 ppm), ethyl pyruvate (670 μL, 6 mmol) and phosphoric acid catalyst (70 mg, 0.2 mmol) were added, and the reaction was stirred for 48h at room temperature. The volatiles were distilled off at reduced pressure and the crude residue was purified by column chromatography (Hexanes /AcOEt 9:1) to afford 520 mg (75%) of **4u** as a white solid. Physical and spectroscopic data are in agreement with literature data.[3]

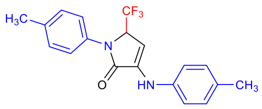

4u

Diethyl ((5-oxo-1-(p-tolyl)-4-(p-tolylamino)-2,5-dihydro-1H-pyrrol-2-yl)methyl)-phosphonate (**4v**). The general procedure was followed in diethyl ether at room temperature using *p*-toluidine (429 mg, 4 mmol), diethyl (2-oxoethyl)phosphonate (360 mg, 2 mmol) and ethyl pyruvate (670 μL, 6 mmol). The residue was purified by column chromatography (Hexanes /AcOEt 9:1) affording 729 mg (85%) of **4v** as a yellow solid. Physical and spectroscopic data are in agreement with literature data.[3]

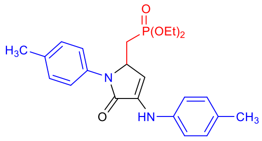

4v

5-((Diphenylphosphoryl)methyl)-1-(p-tolyl)-3-(p-tolylamino)-1H-pyrrol-2(5H)-one (**4w**). The general procedure was followed in diethyl ether at room temperature using *p*-toluidine (429 mg, 4 mmol), 2-(diphenylphosphoryl)acetaldehyde (488 mg, 2 mmol) and ethyl pyruvate (670 μL, 6 mmol). The residue was purified by column chromatography (Hexanes /AcOEt 9:1) affording 749 mg (76%) of **4w** as a white solid. Physical and spectroscopic data are in agreement with literature data.[3]

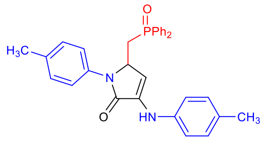

4w

4-Methyl-5-(4-nitrophenyl)-1-(p-tolyl)-3-(p-tolylamino)-1,5-dihydro-2H-pyrrol-2-one (**5a**). The general procedure was followed in diethyl ether at room temperature temperature using *p*-toluidine (429 mg, 4 mmol), *p*-nitrobenzaldehyde (302 mg, 2 mmol) and methyl 2-oxobutanoate (704 μL, 6 mmol). The residue was crystallized in diethyl ether affording 28.9 mg (70%) of **5a** as a yellow solid. Physical and spectroscopic data are in agreement with literature data.[1]

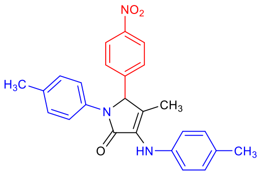

5a

4-Benzyl-5-(p-nitrophenyl)-1-(p-tolyl)-3-(p-tolylamino)-1,5-dihydro-2H-pyrrol-2-one (**5b**). The general procedure was followed in diethyl ether at room temperature using *p*-toluidine (429 mg, 4 mmol), *p*-nitrobenzaldehyde (302 mg, 2 mmol) and ethyl 2-oxo-4-phenylbutanoate (1.134 ml, 6 mmol). The residue was crystallized in diethyl ether affording 34.7 mg (71%) of **5b** as a yellow solid. Physical and spectroscopic data are in agreement with literature data.[1]

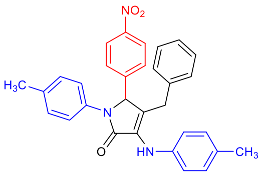

5b

Methyl 5-oxo-2-phenyl-1-(p-tolyl)-4-(p-tolylamino)-2,5-dihydro-1H-pyrrole-3-carboxylate (**5c**). The general procedure was followed using *p*-toluidine (429 mg, 4 mmol), benzaldehyde (204 μL, 2 mmol) and ethyl pyruvate (670 μL, 6 mmol). The residue was purified by column chromatography (Hexanes /AcOEt 90:10) affording 519 mg (63%) of **5c** as a withe solid. M.p. (Et_2O) = 179-181 °C. ^{1}H NMR (400 MHz, $CDCl_3$) δ 8.14 (s, 1H), 7.33 (d, $^3J_{HH}$ = 8.5Hz, 2H), 7.25 (m, 4H), 7.20 (m, 1H), 7.13 (d, $^3J_{HH}$= 8,3 Hz, 2H), 7.09 (d, $^3J_{HH}$= 8,5 Hz, 2H), 7.03 (d, $^3J_{HH}$= 8,3 Hz, 2H), 5.77 (s, 1H), 3.54 (s, 3H), 2.34 (s, 3H), 2.23 (s, 3H). ^{13}C {^{1}H} NMR (101 MHz, $CDCl_3$): δ 164.9 (C=O), 163.9 (C=O), 142.8 (C_{quat}), 137.2 (C_{quat}), 136.0 (C_{quat}), 135.5 (C_{quat}), 134.7 (C_{quat}), 134.1 (C_{quat}), 129.5 (2xCH), 129.1 (2xCH), 128.5 (2xCH), 128.1 (CH), 127.7 (2xCH), 123.3 (2xCH), 122.8 (2xCH), 108.9 (C_{quat}), 63.2 (CH), 51.2 (CH_3), 21.1 (CH_3), 21.0 (CH_3). FTIR (neat) ν_{max} : 3306 (N-H), 1702 (C=O), 1679 (C=O), 1632 (C=C). HRMS (ESI-TOF) m/z calcd for $C_{26}H_{25}N_2O_3$ $[M+H]^+$ 413,1865, found 413,1863.

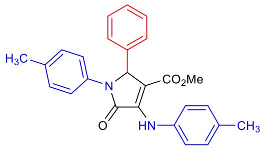

5c

Diethyl (5-oxo-1-(p-tolyl)-4-(p-tolylamino)-2,5-dihydro-1H-pyrrol-3-yl)phosphonate (***5d***). The general procedure was followed in MTBE at 55°C (heating plate, Heat-On) using *p*-toluidine (429 mg, 4 mmol), 37 % aq. formaldehyde (150 µL, 2 mmol) and ethyl 3-(diethoxyphosphoryl)-2-oxopropanoate (1.513 g, 6 mmol). The residue was purified by column chromatography (Hexanes /AcOEt 9:1) affording 631 mg (76%) of **5d** as an orange solid. Physical and spectroscopic data are in agreement with literature data.[3]

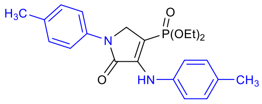

5d

4-(Diphenylphosphoryl)-1-(p-tolyl)-3-(p-tolylamino)-1,5-dihydro-2H-pyrrol-2-one (***5e***). The general procedure was followed in MTBE at 55°C (heating plate, Heat-On) using *p*-toluidine (429 mg, 4 mmol), 37 % aq. formaldehyde (150 µL, 2 mmol) and ethyl 3-(diphenylphosphoryl)-2-oxopropanoate (1.897 g, 6 mmol). The residue was purified by column chromatography (Hexanes /AcOEt 9:1) affording 698 mg (73%) of **5e** as an orange solid. Physical and spectroscopic data are in agreement with literature data.[3]

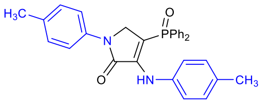

5e

1-(p-Methoxyphenyl)-3-((p-methoxyphenyl)amino)-5-phenyl-1,5-dihydro-2H-pyrrol-2-one (***6a***). The general procedure was followed in diethyl ether at room temperature using *p*-anisidine (492 mg, 4 mmol), benzaldehyde (204 µL, 2 mmol) and ethyl pyruvate (670 µL, 6 mmol). The residue was purified by column chromatography (Hexanes /AcOEt 8:2) affording 571 mg (74%) of **6a** as a white solid. Physical and spectroscopic data are in agreement with literature data.[1]

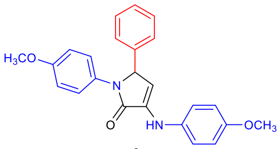

6a

1-(p-Methoxyphenyl)-3-((p-methoxyphenyl)amino)-5-(p-nitrophenyl)-1H-pyrrol-2(5H)-one (***6b***). The general procedure was followed in diethyl ether at room temperature using *p*-anisidine (492 mg, 4 mmol), *p*-nitrobenzaldehyde (302 mg, 2 mmol) and ethyl pyruvate (670 µL, 6 mmol). The residue was purified by column chromatography (Hexanes /AcOEt 8:2) affording 698 mg (81%) of **6b** as a yellow solid. Physical and spectroscopic data are in agreement with literature data.[1]

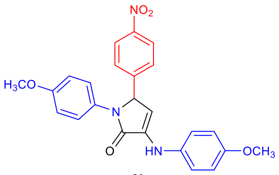

6b

Diethyl ((1-(p-methoxyphenyl)-4-((p-methoxyphenyl)amino)-5-oxo-2,5-dihydro-1H-pyrrol-2-yl)methyl)-phosphonate (***6c***). The general procedure was followed in diethyl ether at room temperature using *p*-anisidine (492 mg, 4 mmol), diethyl (2-oxoethyl)phosphonate (360 mg, 2 mmol) and ethyl pyruvate (670 µL, 6 mmol). The residue was purified by column chromatography (Hexanes /AcOEt 9:1) affording 737 mg (80%) of **6c** as a white solid. Physical and spectroscopic data are in agreement with literature data.[3]

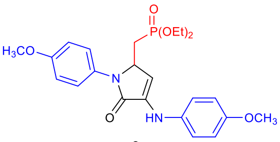

6c

1-(p-Chlorophenyl)-3-((p-chlorophenyl)amino)-5-(p-nitrophenyl)-1,5-dihydro-2H-pyrrol-2-one (***7***). The general procedure was followed in MTBE at 55°C (heating plate, Heat-On) using *p*-chloroaniline (510 mg, 4 mmol), *p*-nitrobenzaldehyde (302 mg, 2 mmol) and ethyl pyruvate (670 µL, 6 mmol). The residue was purified by column chromatography (Hexanes /AcOEt 90:1) affording 692 mg (80%) of **7** as a yellow solid. M.p. (Et_2O) = 151-153 °C. ^1H NMR (400 MHz, DMSO-d_6) δ 8.49 (bs, 1H), 8.15 (d, $^3J_{HH}$ = 8.8 Hz, 2H), 7.67 (d, $^3J_{HH}$ = 8.9 Hz, 2H), 7.56 (d, $^3J_{HH}$ = 8.7 Hz, 2H), 7.40 (d, $^3J_{HH}$ = 8.9 Hz, 2H), 7.32 (d, $^3J_{HH}$ = 9.1 Hz, 2H), 7.27 (d, $^3J_{HH}$ = 8.8 Hz, 2H), 6.43 (d, $^3J_{HH}$ = 2.7 Hz, 1H), 6.28 (d, $^3J_{HH}$ = 2.7 Hz, 1H) . ^{13}C {^1H} NMR (75 MHz, DMSO-d_6): δ 166.9 (C=O), 147.7 (C_{quat}), 146.1 (C_{quat}), 141.4 (C_{quat}), 136.3 (C_{quat}), 136.3 (C_{quat}), 132.7 (C_{quat}), 129.4 (2xCH), 129.3 (2xCH), 128.8 (2xCH), 124.6 (2xCH), 124.5 (C_{quat}), 123.5 (2xCH), 119.0 (2xCH), 110.0 (CH), 62.2 (CH). FTIR (neat) ν_{max} : 3310 (N-H), 1673 (C=O), 1645 (C=CH), 1516 (NO_2), 1347 (NO_2), 1110 (C-Cl) . HRMS (ESI-TOF) m/z calcd for $C_{22}H_{16}Cl_2N_3O_3$ $[M+H]^+$ 440,0568, found 440,0551.

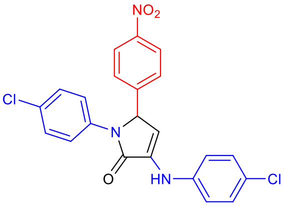

7

1,3-Bis(p-bromophenyl)-5-(p-nitrophenyl)-1H-pyrrol-2(5H)-one (***8a***). The general procedure was followed in diethyl ether at room temperature using *p*-bromoaniline (688 mg, 4 mmol), *p*-nitrobenzaldehyde (302 mg, 2 mmol) and ethyl pyruvate (670 µL, 6 mmol). The residue was purified by column chromatography (Hexanes /AcOEt 8:2) affording 938 mg (89%) of **8a** as a yellow solid. Physical and spectroscopic data are in agreement with literature data.[1]

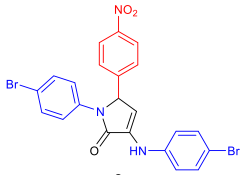

8a

1-(p-Bromophenyl)-3-((p-bromophenyl)amino)-5-(p-(trifluoromethyl)phenyl)-1,5-dihydro-2H-pyrrol-2-one (***8b***). The general procedure was followed in diethyl ether at room temperature using *p*-bromoaniline (688 mg, 4 mmol), *p*-(trifluoromethyl)benzaldehyde (273 µL, 2 mmol)and ethyl pyruvate (670 µL, 6 mmol). The residue was crystallized in diethyl ether affording 1.077 g of **8b** (98%) as a white solid. Physical and spectroscopic data are in agreement with literature data.[1]

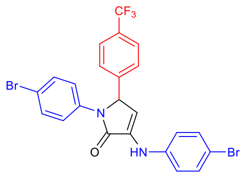

8b

1-(m-Chlorophenyl)-3-((m3-chlorophenyl)amino)-5-(p-nitrophenyl)-1,5-dihydro-2H-pyrrol-2-one (***9***). The general procedure was followed in MTBE at 55°C (heating plate, Heat-On) using *m*-chloroaniline (423 µL, 4 mmol), *p*-nitrobenzaldehyde (302 mg, 2 mmol) and ethyl pyruvate (670 µL, 6 mmol). The residue was crystallized in diethyl ether affording 737 mg (84%) of **9** as a yellow solid. Physical and spectroscopic data are in agreement with literature data.[1]

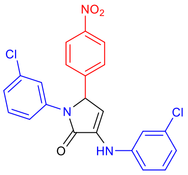

9

1-(o-Fluorophenyl)-3-((o-fluorophenyl)amino)-5-(p-nitrophenyl)-1H-pyrrol-2(5H)-one (***10***). The general procedure was followed in diethyl ether at room temperature using *o*-fluoroaniline (386 µL, 4 mmol), *p*-nitrobenzaldehyde (302 mg, 2 mmol) and ethyl pyruvate (670 µL, 6 mmol). The residue was purified by column chromatography (Hexanes /AcOEt 8:2) affording 610 mg (75%) of **10** as a yellow solid. Physical and spectroscopic data are in agreement with literature data.[1]

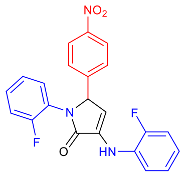

10

1-(m-(Trifluoromethyl)phenyl)-3-((m-(trifluoromethyl)phenyl)amino)-1,5-dihydro-2H-pyrrol-2-one (***11a***). The general procedure was followed in diethyl ether at room temperature using *m*-(trifluoromethyl)aniline (499 mg, 4 mmol), 37 % aq. formaldehyde (150 µL, 2 mmol) and ethyl pyruvate (670 µL, 6 mmol). The residue was purified by column chromatography (Hexanes /AcOEt 9:1) affording 562 mg (73%) of **11a** as an orange solid. M.p. (Et_2O) = 160-162 °C. ^1H NMR (300 MHz, $CDCl_3$) δ 8.08–8.03 (m, 2H), 7.58–7.23 (m, 6H), 6.76 (s, 1H), 6.17 (t, $^3J_{HH}$ = 2.6 Hz, 1H), 4.50 (d, $^3J_{HH}$ = 2.6 Hz, 2H) . ^{13}C {^1H} NMR (75 MHz, $CDCl_3$): δ 166.5 (C=O), 141.9 (C_{quat}), 139.6 (C_{quat}), 133.7 (C_{quat}), 132.1 (q, $^2J_{FC}$ = 32.4 Hz, C_{quat}), 131.9 (q, $^2J_{FC}$ = 32.4 Hz, C_{quat}), 130.2 (CH), 130.1 (CH), 124.1 (q, $^1J_{FC}$ = 272.5 Hz, CF_3), 124.0 (q, $^1J_{FC}$ = 272.4 Hz, CF_3), 121.8 (CH), 121.3 (q, $^3J_{FC}$ = 3.7 Hz, CH), 120.0 (CH), 118.1 (q, $^3J_{CF}$ = 3.9 Hz, CH), 115.3 (q, $^3J_{CF}$ = 3.9 Hz, CH), 112.9 (q, $^3J_{CF}$ = 3.8 Hz, CH), 102.4 (CH), 49.7 (CH_2) . ^{19}F NMR (282 MHz, $CDCl_3$): δ -63.2-63.3 . FTIR (neat) ν_{max}: 3342 (N-H), 1685 (C=O), 1644 (C=CH) . HRMS (ESI-TOF) m/z calcd for $C_{18}H_{13}F_6N_2O$ $[M+H]^+$ 387,0932, found 387,0934.

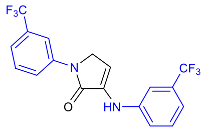

11a

5-(p-Nitrophenyl)-1-(m-(trifluoromethyl)phenyl)-3-((m-(trifluoromethyl)phenyl)amino)-1H-pyrrol-2(5H)-one (***11b***). The general procedure was followed in diethyl ether at room temperature using *m*-(trifluoromethyl)aniline (499 mg, 4 mmol), *p*-nitrobenzaldehyde (302 mg, 2 mmol) and ethyl pyruvate (670 µL, 6 mmol). The residue was purified by column chromatography (Hexanes /AcOEt 8:2) affording 638 mg (63%) of **11b** as a yellow solid. Physical and spectroscopic data are in agreement with literature data.[1]

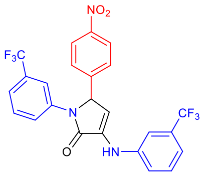

11b

5-(p-Nitrophenyl)-1-(quinolin-3-yl)-3-(quinolin-3-ylamino)-1H-pyrrol-2(5H)-one (***12***). The general procedure was followed in MTBE at 55°C (heating plate, Heat-On) using 2-aminoquinoline (576 mg, 4 mmol), *p*-nitrobenzaldehyde (302 mg, 2 mmol) and ethyl pyruvate (670 µL, 6 mmol). The residue was purified by column chromatography (Hexanes /AcOEt 8:2) affording 321 mg (34%) of **12** as a yellow solid. M.p. (Et_2O) = 201 °C (dec.) ^{1}H NMR (300 MHz, $CDCl_3$) δ 9.04 (d, $^4J_{HH}$ = 1.5 Hz, 1H), 8.79 (d, $^4J_{HH}$ = 1.7 Hz, 1H), 8.46 (bs, 1H), 8.17 (d, $^3J_{HH}$ = 7.4 Hz, 2H), 8.03 (t, $^3J_{HH}$ = 8.4 Hz, 2H), 7.83 – 7.42 (m, 8H), 7.04 (s, 1H), 6.32 (d, $^3J_{HH}$ = 1.5 Hz, 1H), 6.05 (d, $^3J_{HH}$ = 1.7 Hz, 1H) . ^{13}C {^{1}H} NMR (75 MHz, $CDCl_3$): δ 167.0 (C=O), 148.3 (C_{qua}), 145.8 (C_{quat}), 144.2 (CH), 144.0 (CH), 143.9 (C_{quat}), 134.4 (C_{quat}), 132.6 (C_{quat}), 130.4 (C_{quat}), 129.6 (CH), 129.5 (CH), 129.4 (CH), 128.5 (C_{quat}), 128.0 (CH), 127.9 (C_{quat}), 127.9 (2xCH), 127.8 (CH), 126.9 (CH), 126.7 (CH), 125.0 (2xCH), 120.6 (C_{quat}), 117.9 (CH), 108.5 (CH), 63.5 (CH). FTIR (neat) ν_{max} : 3305 (N-H), 1679 (C=O), 1640 (C=CH), 1508 (NO_2), 1338 (NO_2) . HRMS (ESI-TOF) m/z calcd for $C_{28}H_{20}N_5O_3$ [M+H]$^+$ 474,1566, found 474,1561.

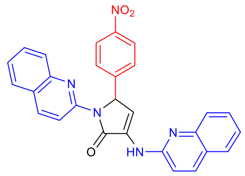

12

Procedure for the synthesis of γ-lactam **16**.

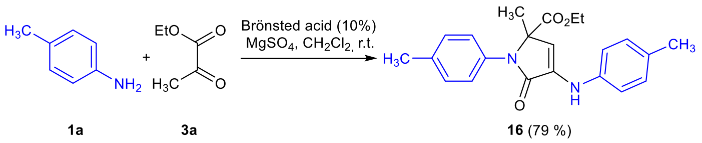

A solution of ethyl pyruvate (**3a**) (446 µL, 4 mmol), *p*-toluidine (**1a**) (429 mg, 4 mmol), BINOL derived phosphoric acid catalyst (70 mg, 0.2 mmol) and anhydrous $MgSO_4$ was stirred in DCM (10 mL) at room temperature for 48h. The volatiles were distilled off at reduced pressure and the crude residue was purified by column chromatography (Hexanes /AcOEt 8:2)

Ethyl 2-methyl-5-oxo-1-(p-tolyl)-4-(p-tolylamino)-2,5-dihydro-1H-pyrrole-2-carboxylate (***16***). The procedure was followed to afford 496 mg (79%) of **16** as a white solid. M.p. (Et_2O) = 188-190 °C (dec.). ^{1}H NMR (300 MHz, $CDCl_3$) δ 7.26 – 7.19 (m, 4H, 4H, 4xCH_{Ar}), 7.15 (d, $^3J_{HH}$ = 8.2 Hz, 2H, 2xCH_{Ar}), 6.99 (d, $^3J_{HH}$ = 8.3 Hz, 2H, 2xCH_{Ar}), 6.59 (s, 1H, NH), 5.92 (s, 1H, CHN), 4.31 – 4.06 (m, 2H, C<u>H</u>$_2$$CH_3$), 2.37 (s, 3H, CH_3 Tol), 2.33 (s, 3H, CH_3 Tol), 1.64 (s, 3H, CH_3), 1.25 (t, $^3J_{HH}$ = 7.1 Hz, 3H, CH_2C<u>H</u>$_3$) .^{13}C {^{1}H} NMR (75 MHz, $CDCl_3$): δ 171.9 (C=O), 167.8 (C=O), 138.8 (C_{quat}), 137.0, (C_{quat}), 134.2, (C_{quat}), 133.5, (C_{quat}), 131.3, (C_{quat}), 130.1, (2xCH_{Ar}), 130.0 (2xCH_{Ar}), 126.0 (2xCH_{Ar}), 117.3 (2xCH_{Ar}), 107.0 (=CH-), 68.9 (C_{quat}-N), 62.3 (CH_2), 21.5 (CH_3), 21.3 (CH_3), 20.9 (CH_3), 14.3 (CH_3). FTIR (neat) ν_{max} : 3305 (N-H), 1705 (C=O), 1679 (C=O), 1640 (-C=CH). HRMS (ESI-TOF) m/z calcd for $C_{22}H_{25}N_2O_3$ [M+H]$^+$ 365,1865, found 365,1860.

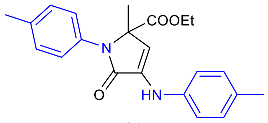

16

General procedure for the hydrogenation of γ-lactams **4u**, **4v**, **6a** and **16**.

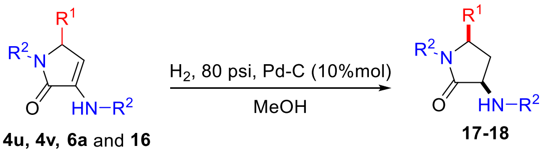

A mixture of γ-lactam **4u**, **4v**, **6a** or **16** (0.5 mmol) and 53.2 mg of 10% palladium on carbon (0.05 mmol Pd) in methanol (100 mL) was stirred for 10 hours under hydrogen pressure at 80 psi. The reaction mixture was filtered through celite, and the filter was washed with dichloromethane (2×20 mL). The combined organic fractions were distilled off at reduced pressure and the residue was crystallized in methanol to afford pure lactams **17-18.**

Ethyl (2S, 4R*)-2-methyl-5-oxo-1-(p-tolyl)-4-(p-tolylamino)pyrrolidine-2-carboxylate (**17**).* The general procedure was followed, using γ-lactam **16** (182 mg, 0.5 mmol), affording 159 mg (87%) of **17** as a white solid. M.p. (Et_2O) = 173-174 °C. ^{1}H NMR (300 MHz, $CDCl_3$) δ 7.21 (d, $^3J_{HH}$ = 8.4 Hz, 2H), 7.15 (d, $^3J_{HH}$ = 8.4 Hz, 2H), 7.02 (d, $^3J_{HH}$ = 8.1 Hz, 2H), 6.60 (d, $^3J_{HH}$ = 8.1 Hz, 2H), 4.42 (bs, 1H), 4.26 (m, 1H), 4.15 (q, $^3J_{HH}$ = 7.0, 2H), 2.61 (m, 1H), 2.46 (m, 1H), 2.36 (s, 3H), 2.26 (s, 3H), 1.63 (s, 3H), 1.21 (t, $^3J_{HH}$ = 7.0 Hz, 3H) .^{13}C {^{1}H} NMR (75 MHz, $CDCl_3$): δ 173.8 (C=O), 173.3 (C=O), 144.9 (C_{quat}), 138.2 (C_{quat}), 133.6 (C_{quat}), 130.1 (2xCH), 130.0 (2xCH), 128.0 (C_{quat}), 127.8 (2xCH), 114.1 (2xCH), 66.4 (C_{quat}), 62.2 (CH_2), 54.4 (CH), 41.7 (CH_2), 23.5 (CH_3), 21.3 (CH_3), 20.6 (CH_3), 14.2 (CH_3). FTIR (neat) ν_{max} : 3343 (N–H), 1701 (C=O), 1685 (C=O). HRMS (ESI-TOF) m/z calcd for $C_{22}H_{27}N_2O_3$ $[M+H]^+$ 367,2021, found 367,2016.

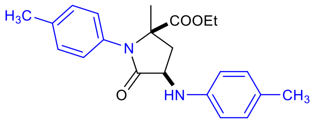

17

((3R, 5R*)-1-(p-tolyl)-3-(p-tolylamino)-5-(trifluoromethyl)pyrrolidin-2-one (**18a**).* The general procedure was followed, using γ-lactam **4u** (173 mg, 0.5 mmol), affording 154 mg (89%) of **18a** as a white solid. Physical and spectroscopic data are in agreement with literature data.[3]

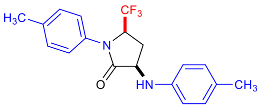

18a

Diethyl (((2R, 4R*)-5-oxo-1-(p-tolyl)-4-(p-tolylamino)pyrrolidin-2-yl)methyl)phosphonate (**18b**).* The general procedure was followed using γ-lactam **4v** (214 mg, 0.5 mmol), affording 199 mg (93%) of **18b** as a white solid. Physical and spectroscopic data are in agreement with literature data.[3]

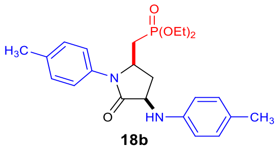

18b

(3R, 5R*)-1-(p-tolyl)-3-((p-methoxyphenyl)amino)-5-phenylpyrrolidin-2-one (**18c**).* The general procedure was followed, using γ-lactam **6a** (194 mg, 0.5 mmol), affording 178 mg (92%) of **18c** as a white solid. Physical and spectroscopic data are in agreement with literature data.[1]

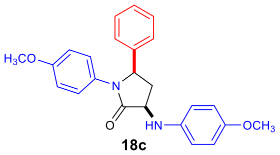

18c

Procedure for the synthesis of γ-lactam **19**.

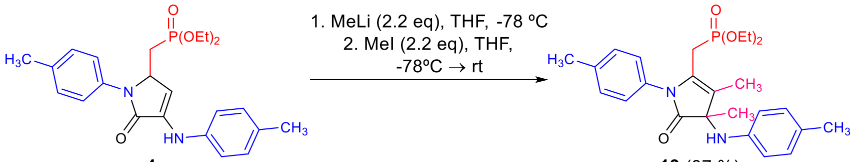

4v **19** (87 %)

A solution of γ-lactam **4v** (214 mg, 0.5 mmol) in THF was cooled to -78 °C, methyl lithium (700 µL, 1.6M, 1.1 mmol) was dropwise added in the mixture and stirred for 1 hour. After 1 hour at -78°C a solution of methyl iodide (70 µL, 1.1 mmol) was slowly added and the reaction was warmed to room temperature overnight. Water (5mL) was added and the resulting mixture was extracted with AcOEt (3x10mL). The combined organic phases were dried with anhydrous $MgSO_4$ and concentrated under reduced pressure. The crude residue was purified by column chromatography (Hexanes /AcOEt 7:3)

*Diethyl ((3,4-dimethyl-5-oxo-1-(p-tolyl)-4-(p-tolylamino)-4,5-dihydro-1H-pyrrol-2-yl)methyl)-phosphonate (**19**).* The procedure was followed, using γ-lactam **4v**, affording 196 mg (87%) of **19** as a yellow solid. Physical and spectroscopic data are in agreement with literature data.[3]

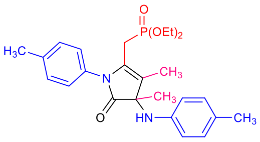

19

General procedure for the Horner-Wadsworth-Emmons reactions of γ-lactam **4v**.

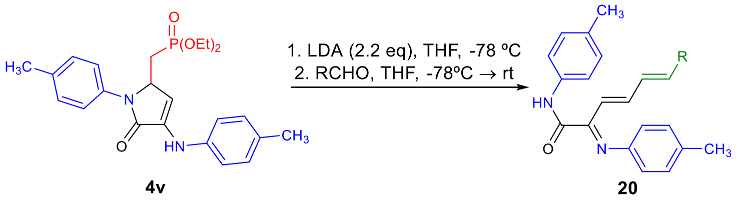

A solution of lactam **4v** (214 mg, 0.5 mmol) in THF (2 mL) was dropwise added to a cooled solution (-78 °C) of LDA (1.1 mmol, prepared from 690 µL of 1.6 M *n*-butyl lithium and 150 µL of diisopropylamine in 5 mL of THF) and the mixture was stirred for 1 hour at -78 °C. Then, the corresponding aldehyde (0.75 mmol) was added and the reaction was warmed to room temperature overnight. The solution was quenched with 10 mL of water and extracted with dichloromethane (3×10 mL), dried with anhydrous $MgSO_4$ and concentrated at reduced pressure. The crude residue was purified by column chromatography (Hexanes /AcOEt 9:1) to afford products **20a-b**.

*(2Z, 3E, 5E)-6-Phenyl-N-(p-tolyl)-2-(p-tolyl imino)hexa-3,5-dienamide (**20a**).* The general procedure was followed using benzaldehyde (76 µL, 0.75 mmol), affording 172 mg (90%) of **20a** as a yellow solid. Physical and spectroscopic data are in agreement with literature data.[3]

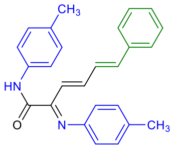

20a

*(2Z, 3E, 5E)-N-(p-Tolyl)-2-(p-tolylimino)-6-(p-(trifluoromethyl)phenyl)hexa-3,5-dienamide (**20b**).*The general procedure was followed using *p*-(trifluoromethyl)benzaldehyde (102 µL, 0.75 mmol), affording 204 mg (0.455 mmol, 91%) of **20b** as an orange solid. Physical and spectroscopic data are in agreement with literature data.[3]

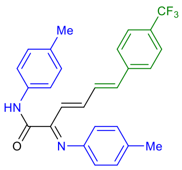

20b

Procedure for the synthesis of γ-lactam **21**.

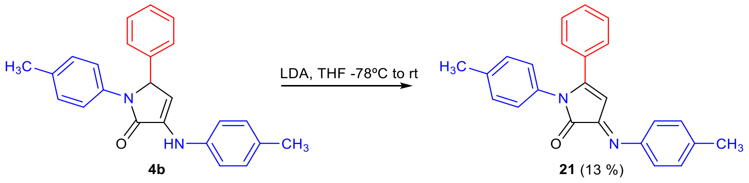

A solution of γ-lactam **4b** (0.214 g, 0.5 mmol) in THF (2 mL) was dropwise added to a cooled solution (-78 °C) of LDA (1.1 mmol, prepared from 0.69 mL of 1.6 M *n*-butyl lithium and 0.15 mL of diisopropylamine in 5 mL of THF) and the mixture was stirred for 1 h. at -78 °C and the reaction was warmed to room temperature overnight. The solution was quenched with 10 mL of water and extracted with dichloromethane (3×10 mL), dried with anhydrous $MgSO_4$ and concentrated at reduced pressure. The crude residue was purified by column chromatography (Hexanes /AcOEt 9:1.

(Z)-5-phenyl-1-(p-tolyl)-3-(p-tolylimino)-1,3-dihydro-2H-pyrrol-2-one (***21***). The procedure was followed, using γ-lactam **4b** to afford 23 mg (13%) of **21** as an orange solid. M.p. (Et_2O) = 207 °C (dec.).^{1}H NMR (400 MHz, $CDCl_3$) δ δ 7.35 (m, 1H, CH_{Ar}), 7.27 (d, $^3J_{HH}$ = 7.8 Hz, 2H, 2xCH_{Ar}), 7.23 – 7.20 (m, 4H, 4xCH_{Ar}), 7.15 - 7.13 (m, 4H, 4xCH_{Ar}), 6.99 (d, $^3J_{HH}$ = 8.3 Hz, 2H, 2xCH_{Ar}), 6.01 (s, 1H, CH), 2.38 (s, 3H, CH_3 Tol), 2.33 (s, 3H, CH_3 Tol) . ^{13}C {^{1}H} NMR (101 MHz, $CDCl_3$): δ 165.3 (C=O), 159.5 (C_{quat}), 156.3 (C_{quat}), 147.9 (C_{quat}), 137.5 (C_{quat}), 136.5 (C_{quat}), 132.2 (C_{quat}), 130.7 (CH_{Ar}), 129.9 (C_{quat}), 129.8 (2xCH_{Ar}), 129.7 (2xCH_{Ar}), 128.6 (2xCH_{Ar}), 128.2 (2xCH_{Ar}), 127.0 (2xCH_{Ar}), 122.3 (2xCH_{Ar}), 97.1 (CH), 21.3 (2xCH_3) . FTIR (neat) ν_{max}: 1690 (C=O), 1635 (C=N), 1640 (-C=CH). HRMS (ESI-TOF) m/z calcd for $C_{24}H_{21}N_2O$ $[M+H]^+$ 353,1662, found 353.1740.

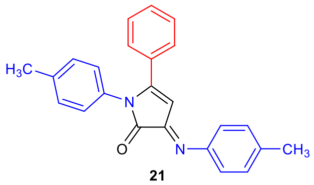

21

2. 1H NMR, 13C NMR, 31P NMR and 19F NMR spectra of compounds 4-12 and 16-21.

*1-(p-Tolyl)-3-(p-tolylamino)-1,5-dihidro-2H-pirrol-2-ona (**4a**).*

^{1}H NMR (400 MHz, $CDCl_3$)

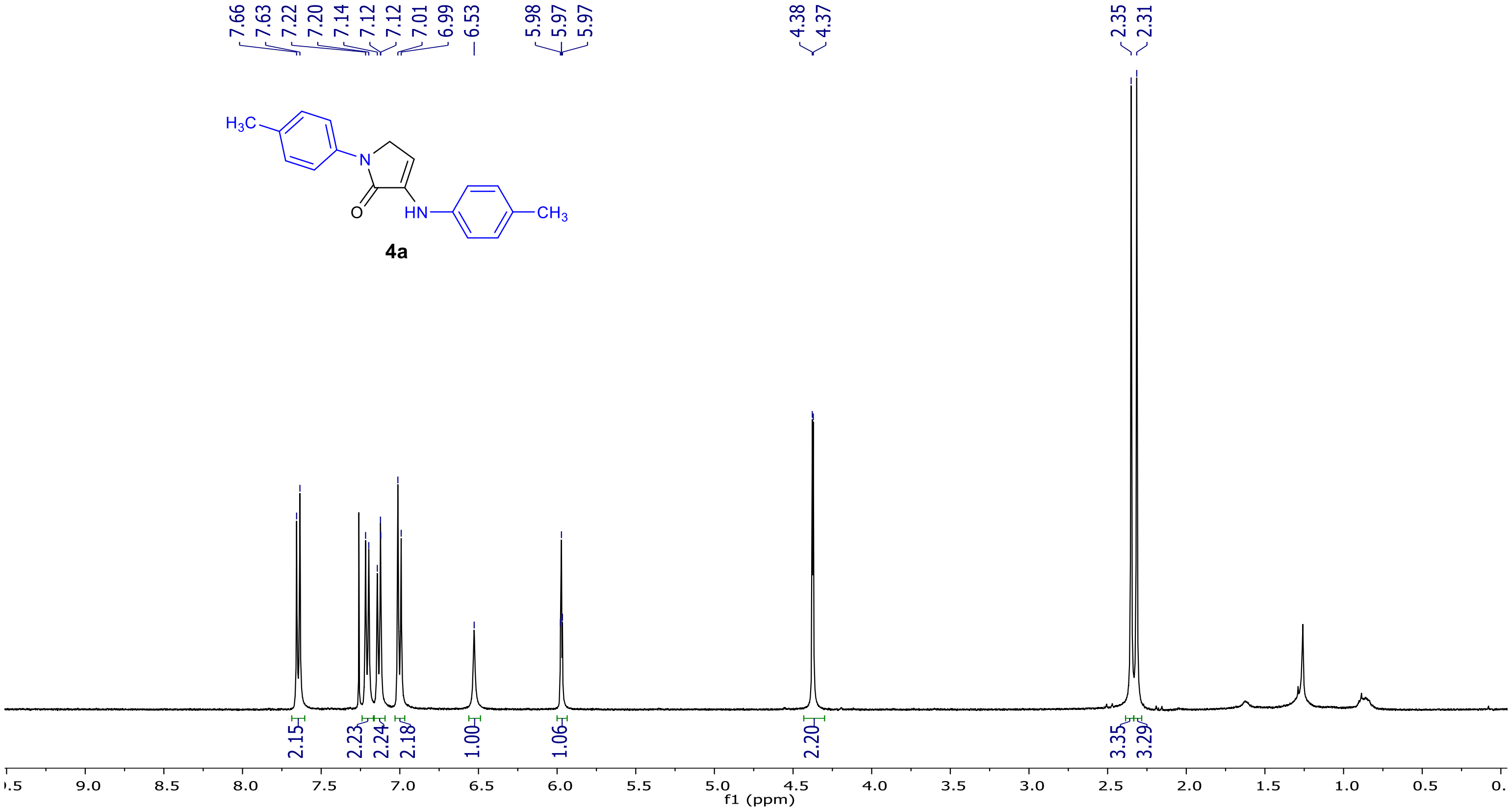

^{13}C NMR (101 MHz, $CDCl_3$)

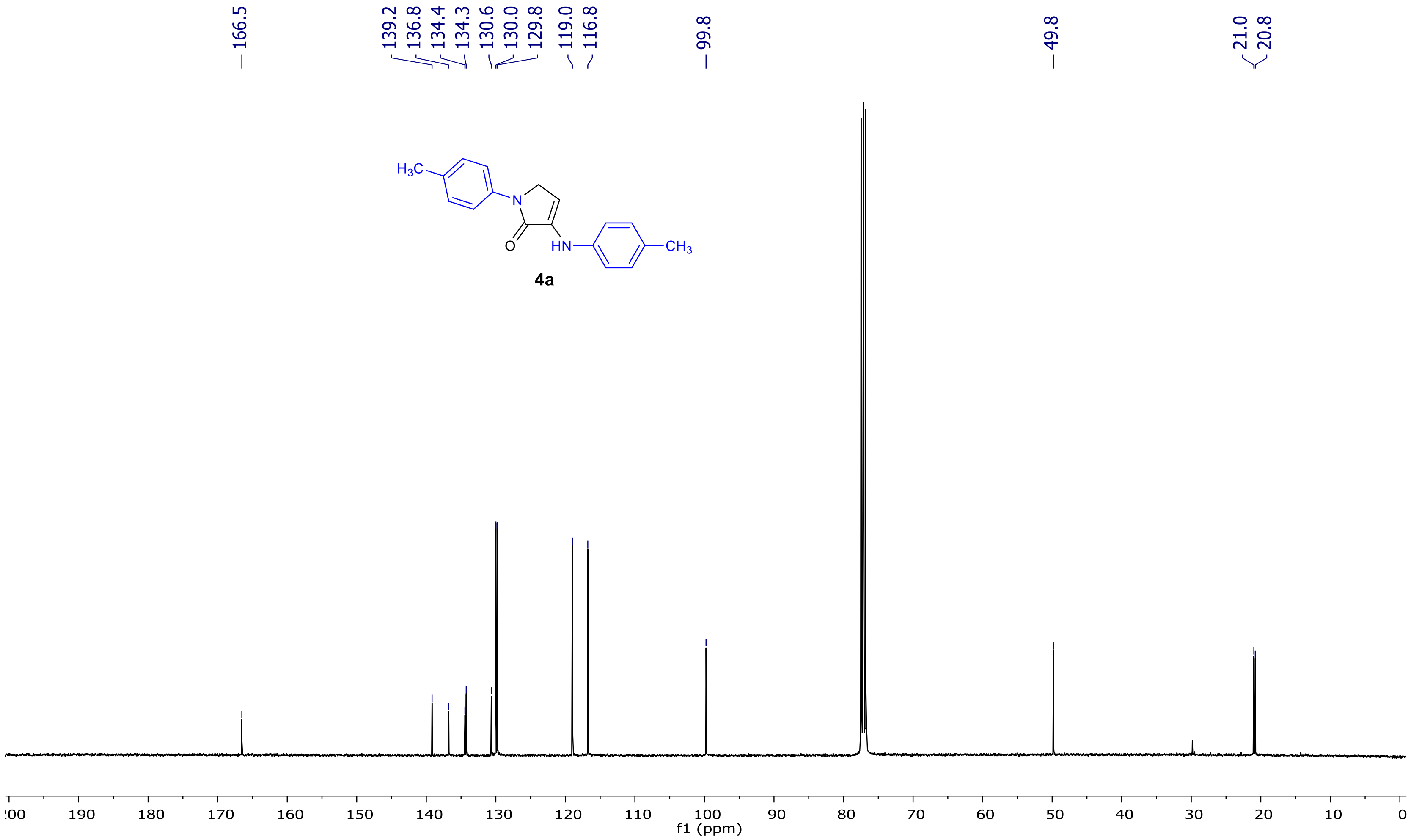

*5-Phenyl-1-(p-tolyl)-3-(p-tolylamino)-1H-pyrrol-2(5H)-one (**4b**).*

1H NMR (400 MHz, $CDCl_3$)

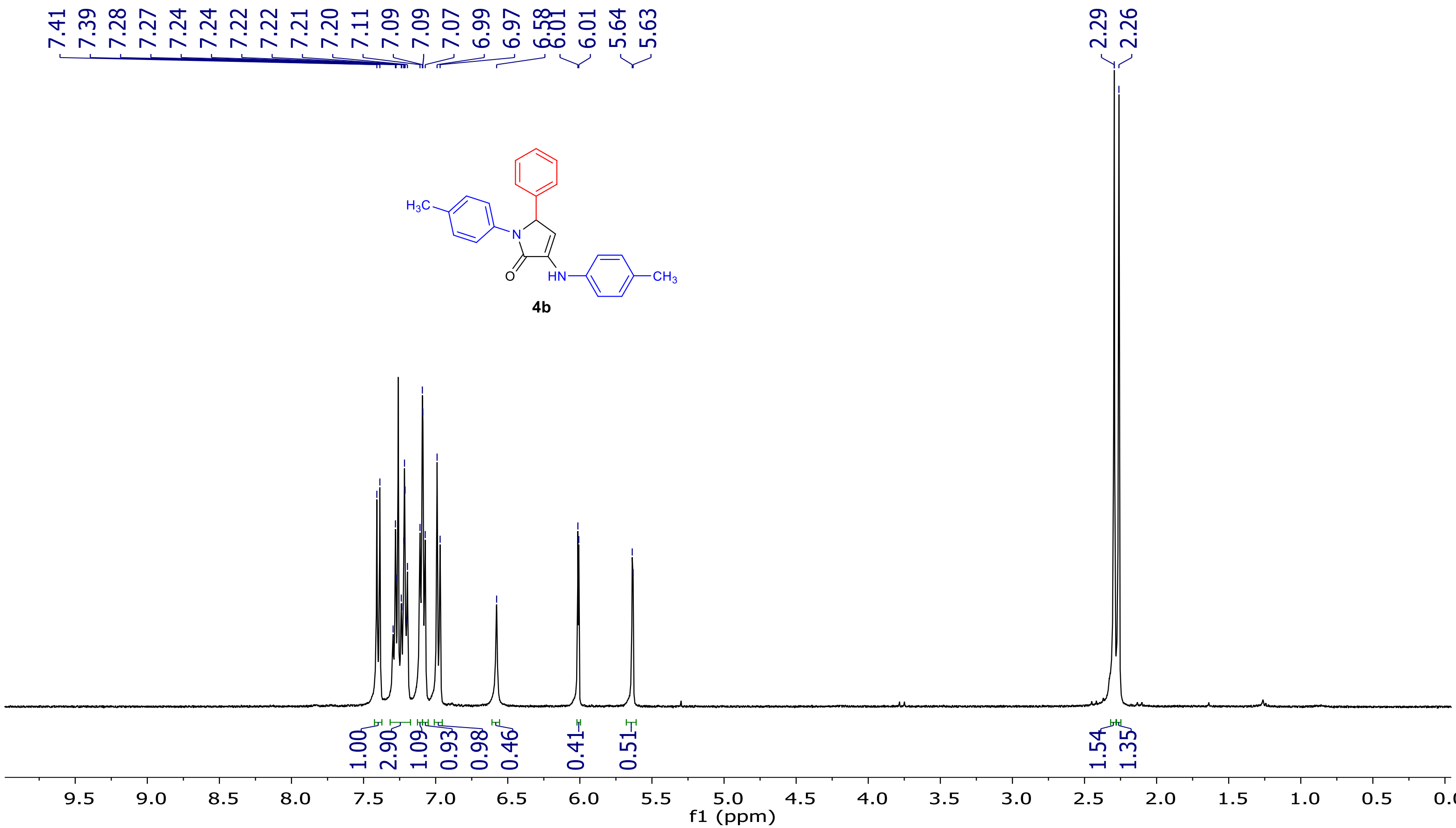

^{13}C NMR (100 MHz, CDCl3)

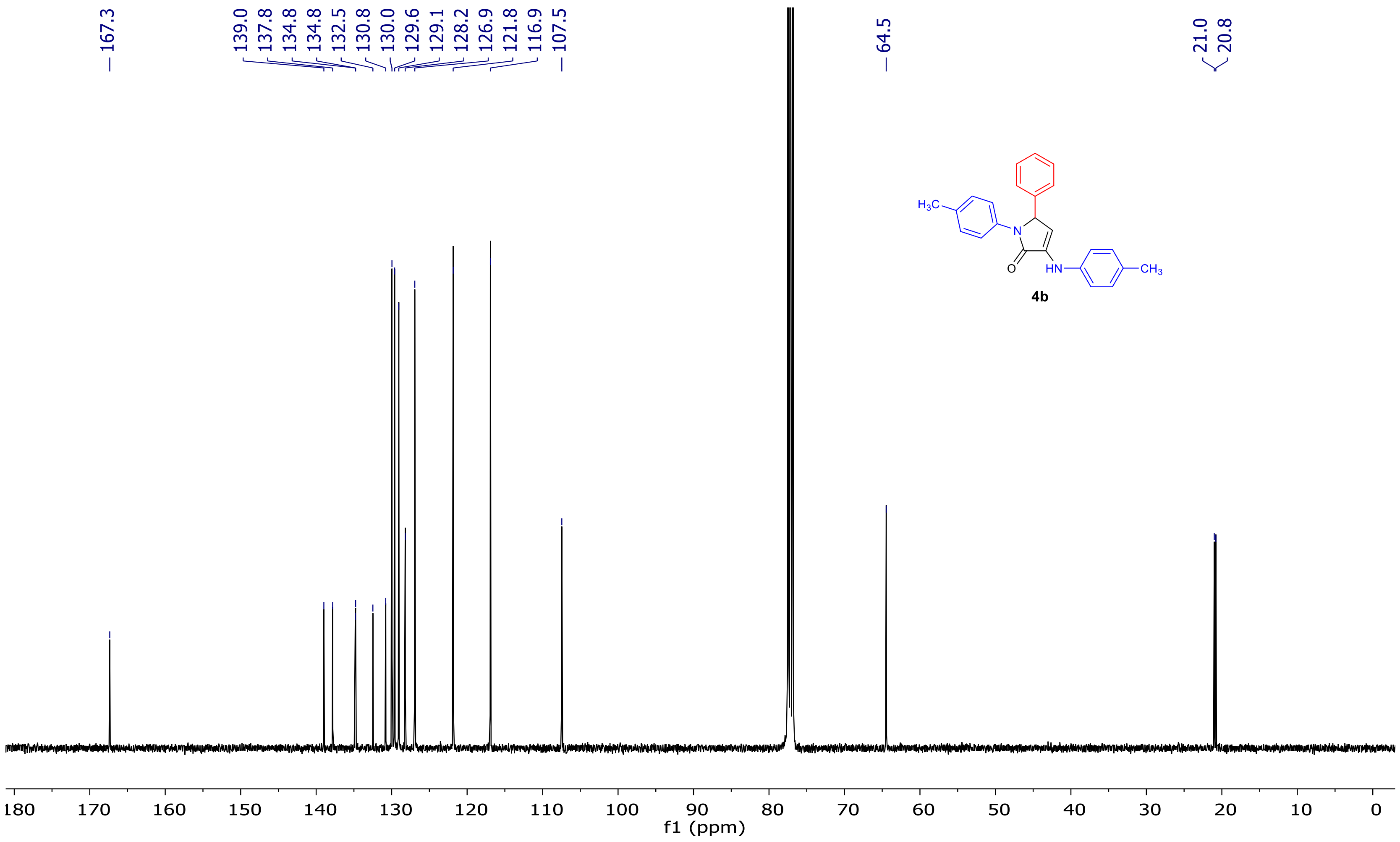

1,*5-Di-p-tolyl-3-(p-tolylamino)-1,5-dihydro-2H-pyrrol-2-one* (**4c**).

^{1}H NMR (400 MHz, $CDCl_3$)

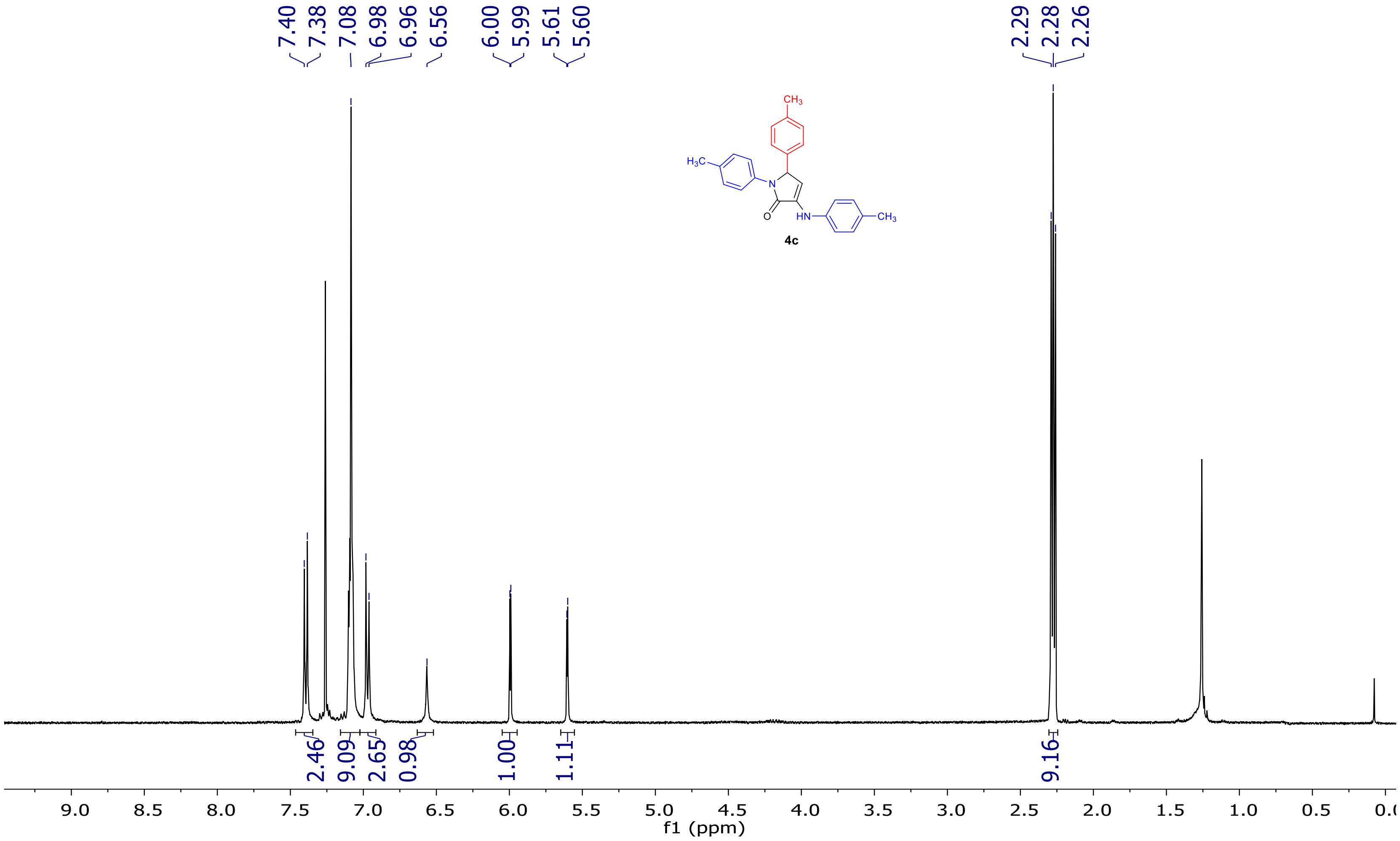

^{13}C NMR (101 MHz, $CDCl_3$)

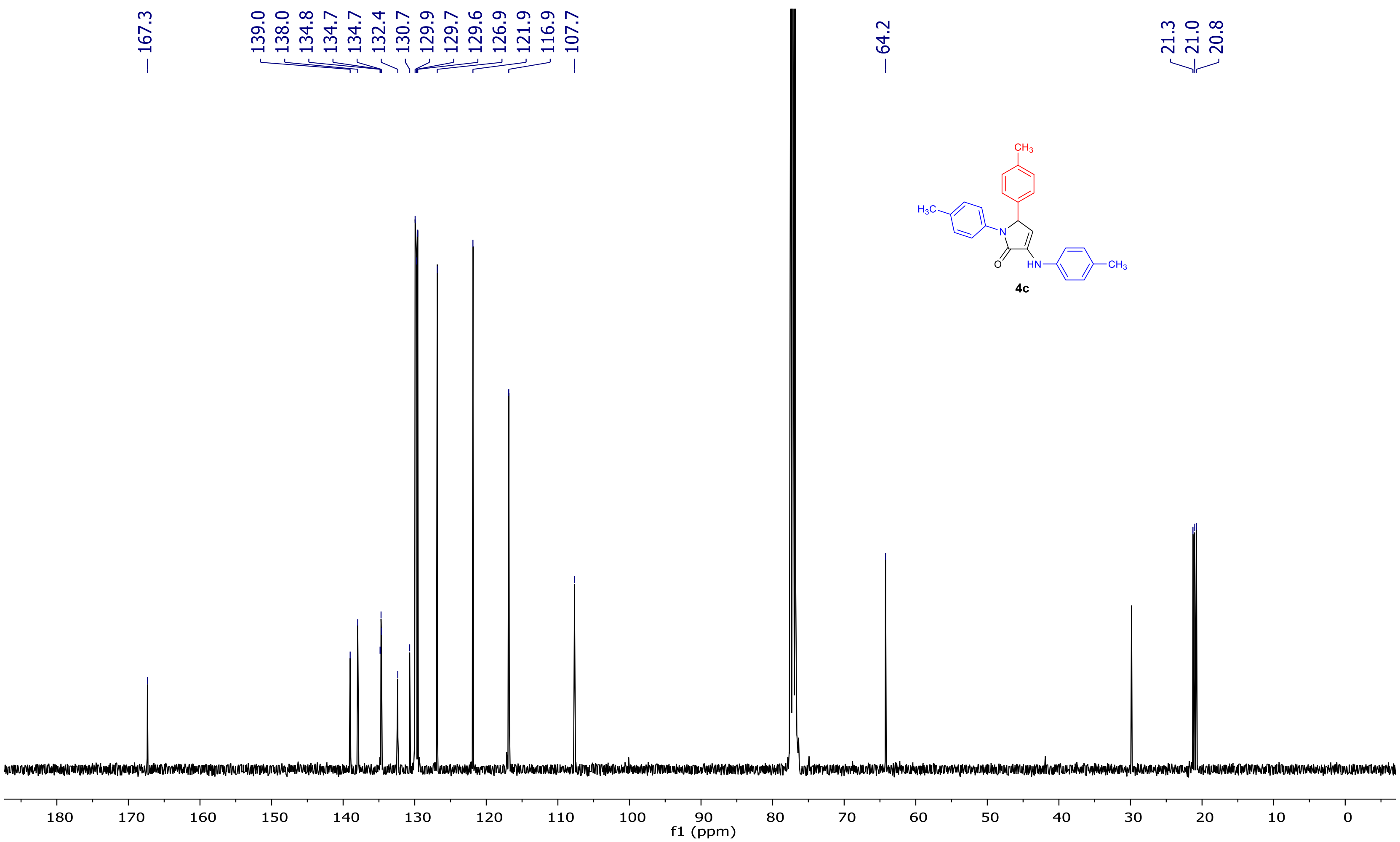

*5-(m-Tolyl)-1-(p-tolyl)-3-(p-tolylamino)-1,5-dihydro-2H-pyrrol-2-one (**4d**).*

^{1}H NMR (400 MHz, $CDCl_3$)

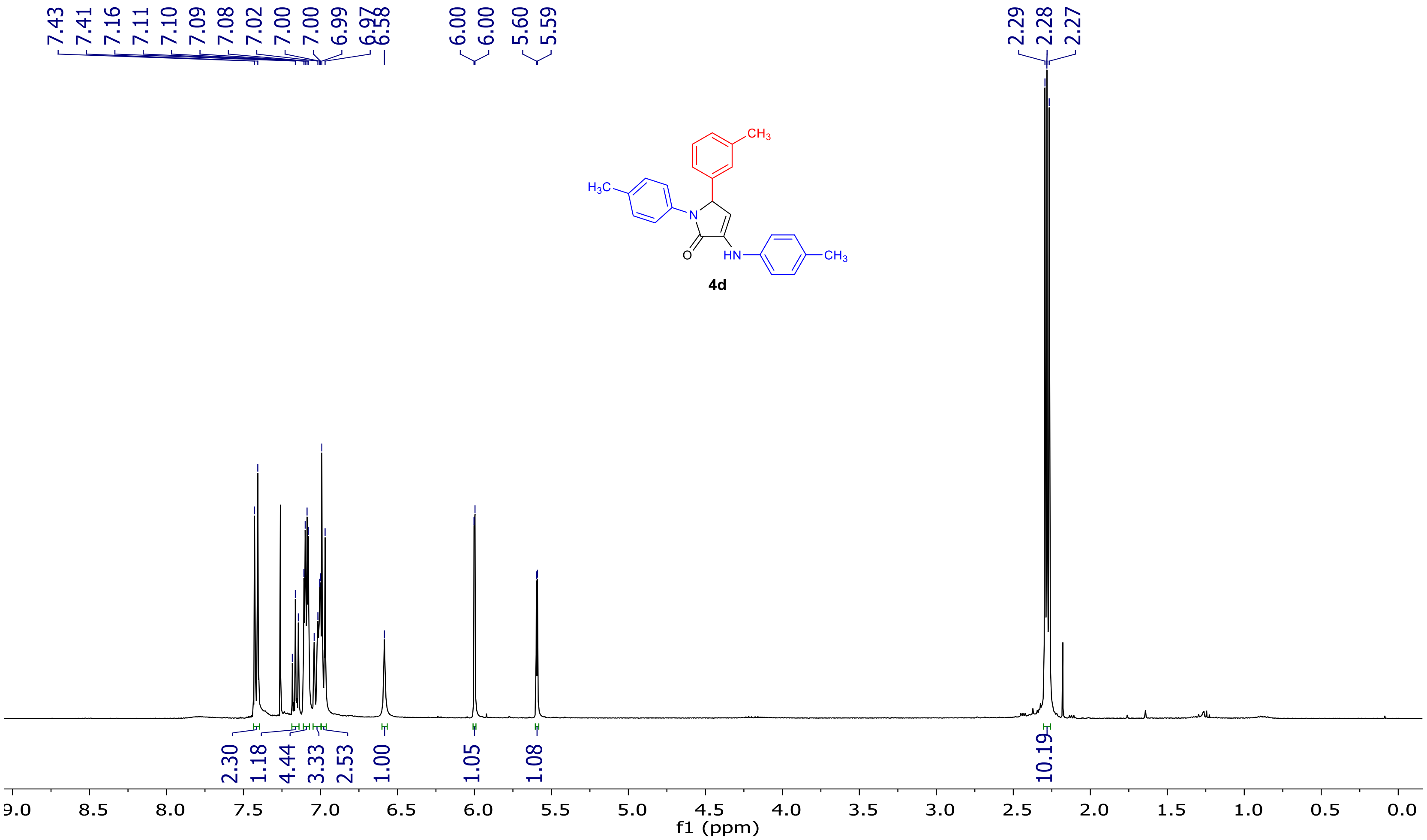

¹³C NMR (101 MHz, $CDCl_3$)

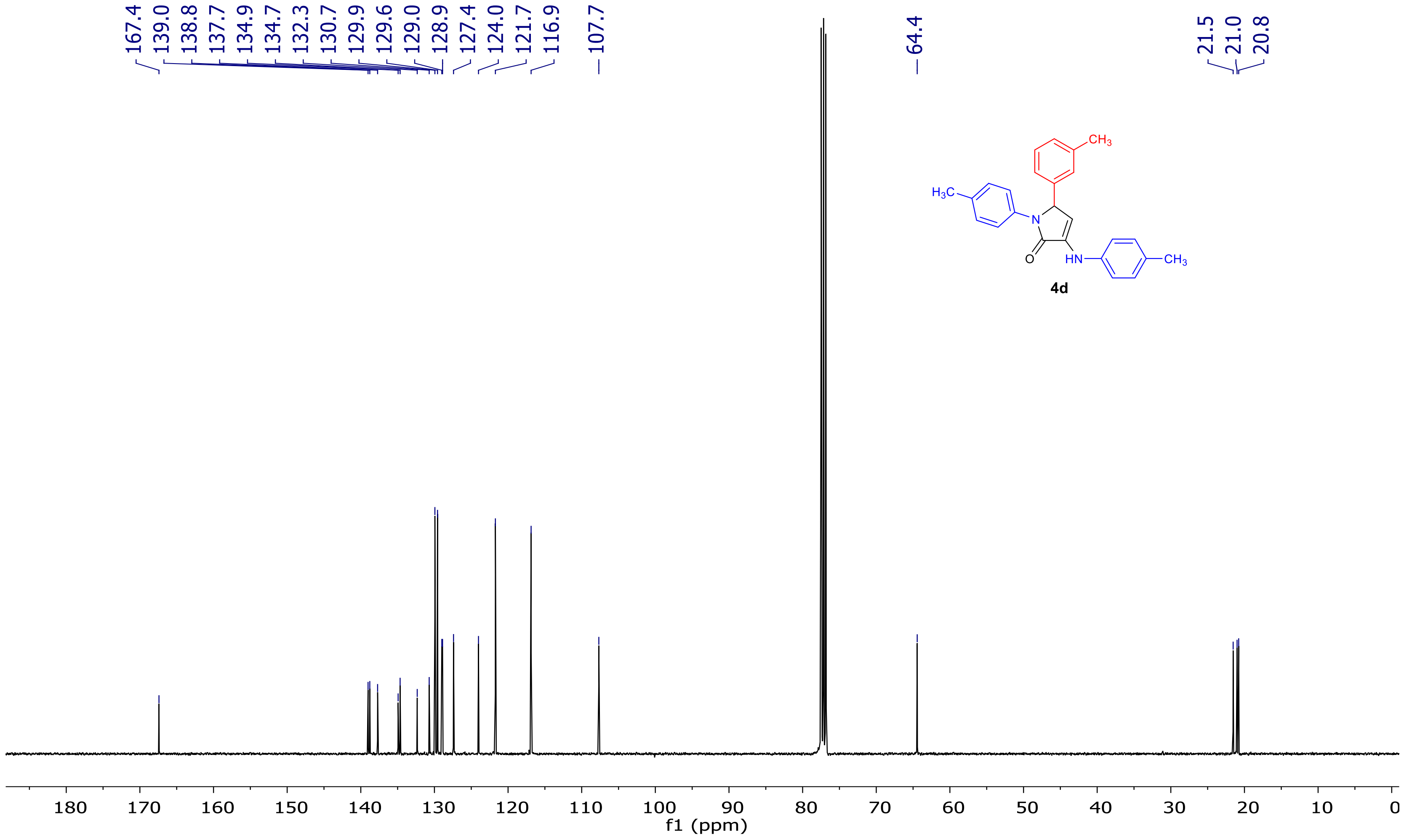

*5-(o-Tolyl)-1-(p-tolyl)-3-(p-tolylamino)-1,5-dihydro-2H-pyrrol-2-one (**4e**).*

^{1}H NMR (400 MHz, $CDCl_3$)

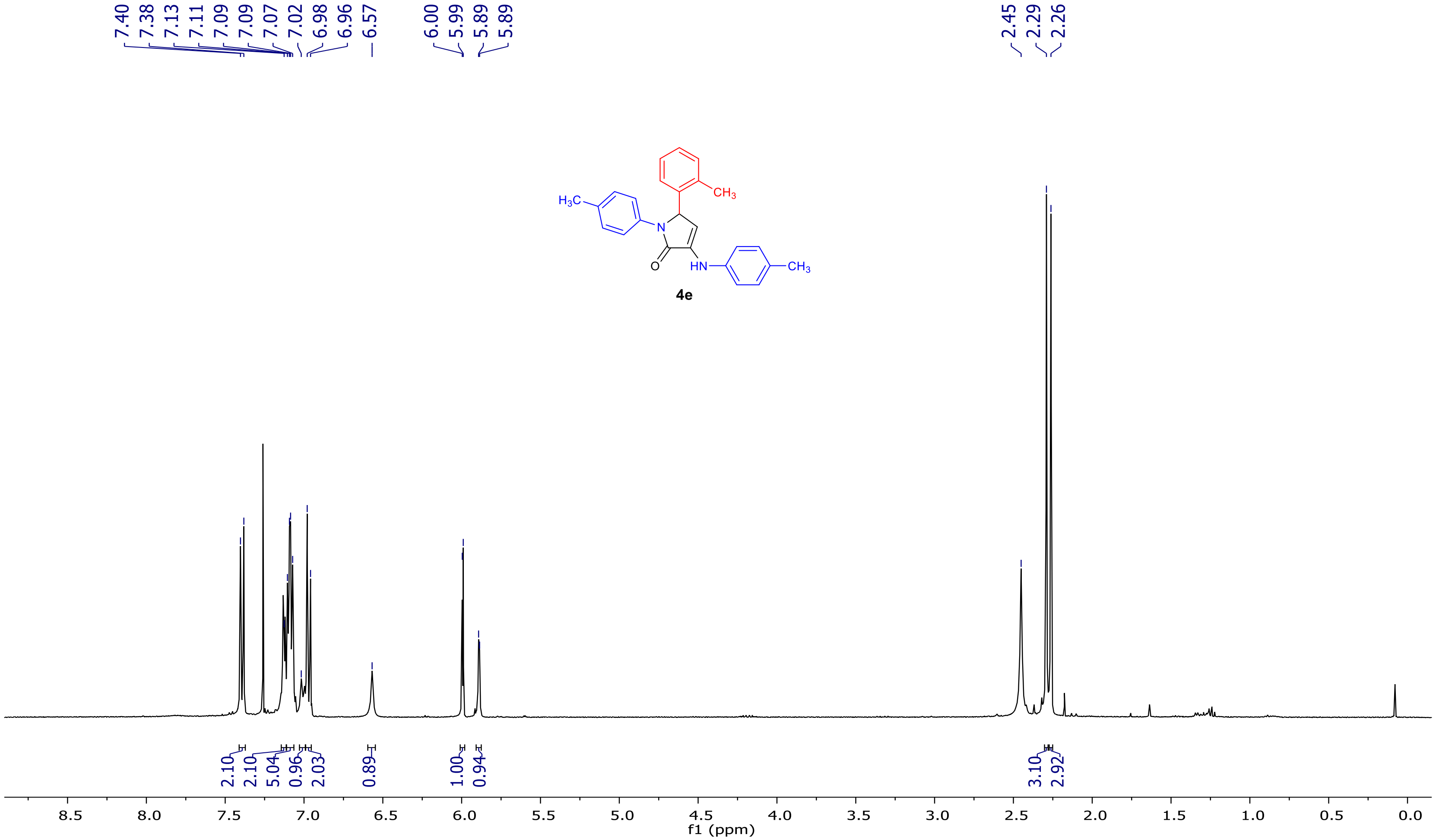

^{13}C NMR (101 MHz, $CDCl_3$)

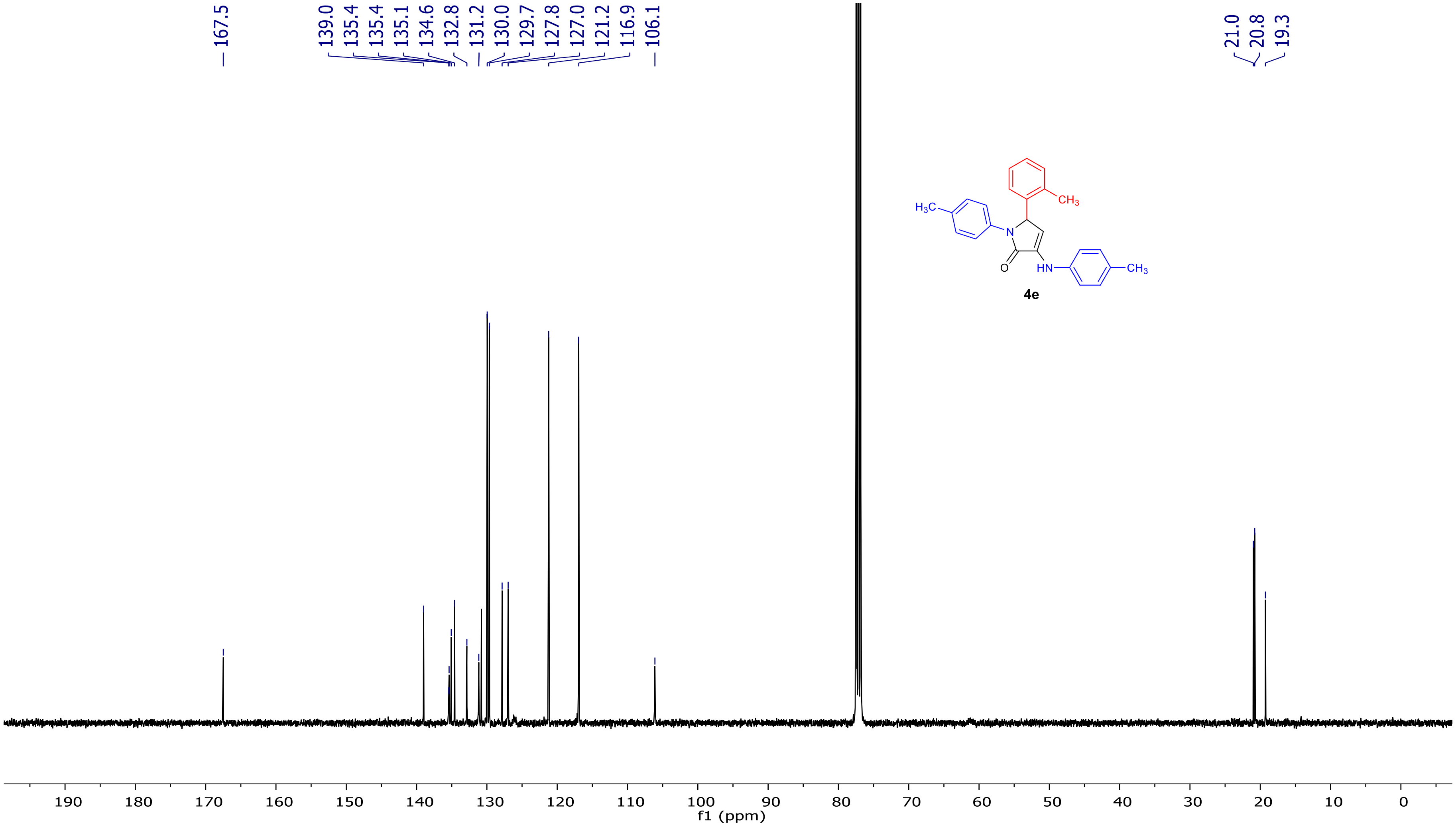

*5-(p-Fluorophenyl)-1-(p-tolyl)-3-(p-tolylamino)-1,5-dihydro-2H-pyrrol-2-one (**4f**).*

^{1}**H NMR** (400 MHz, $CDCl_3$)

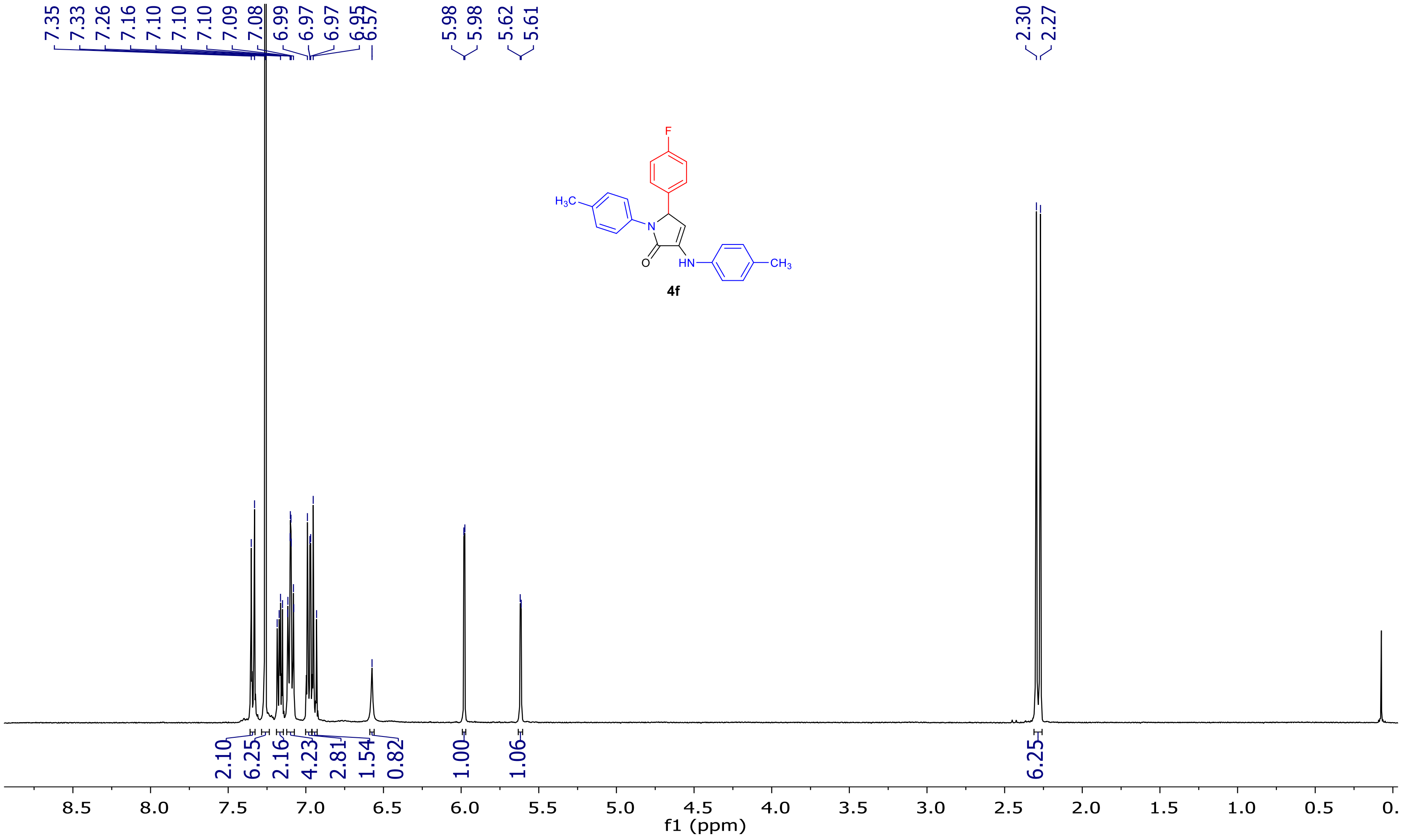

^{13}C NMR (100 MHz, $CDCl_3$)

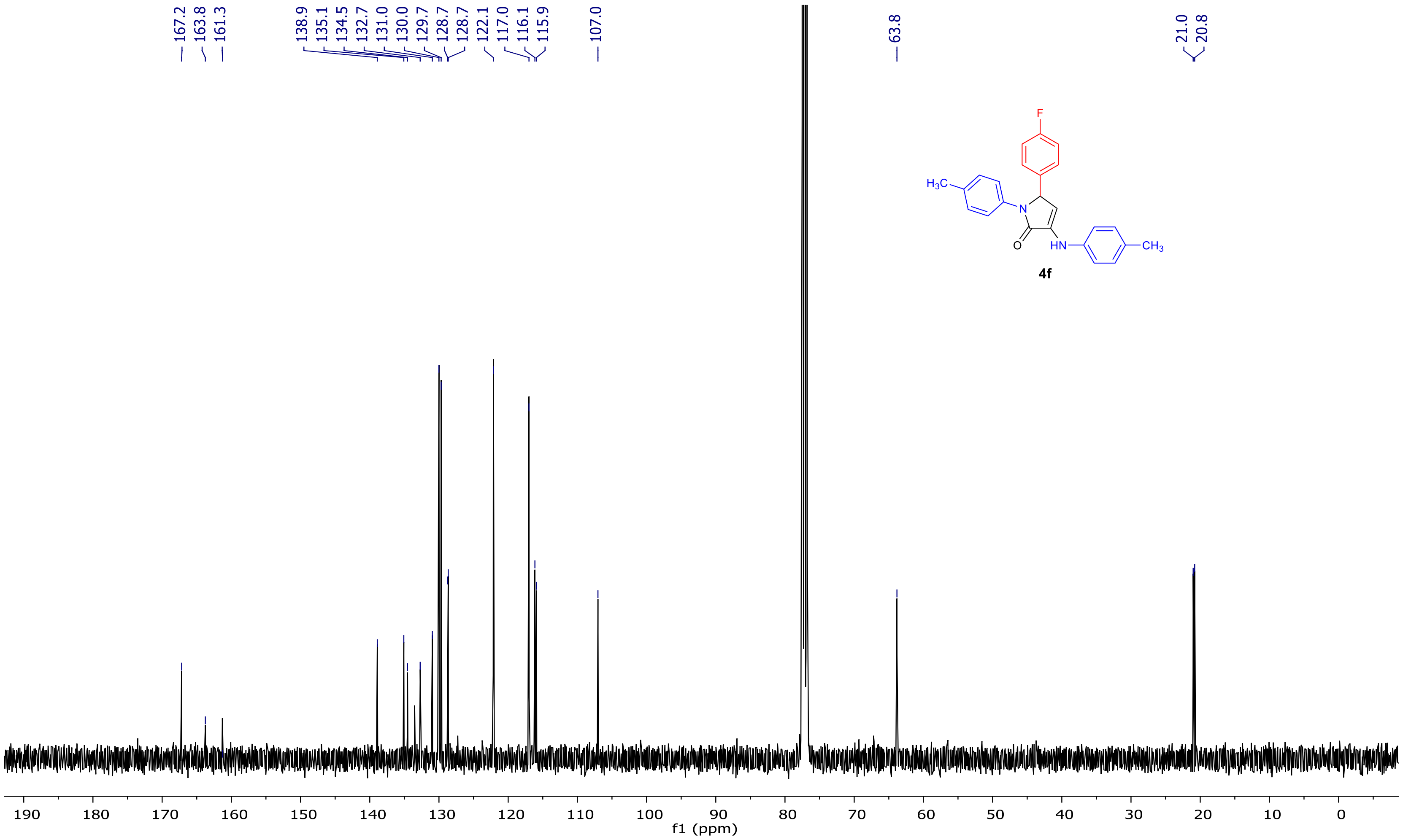

^{19}F NMR (282 MHz, $CDCl_3$)

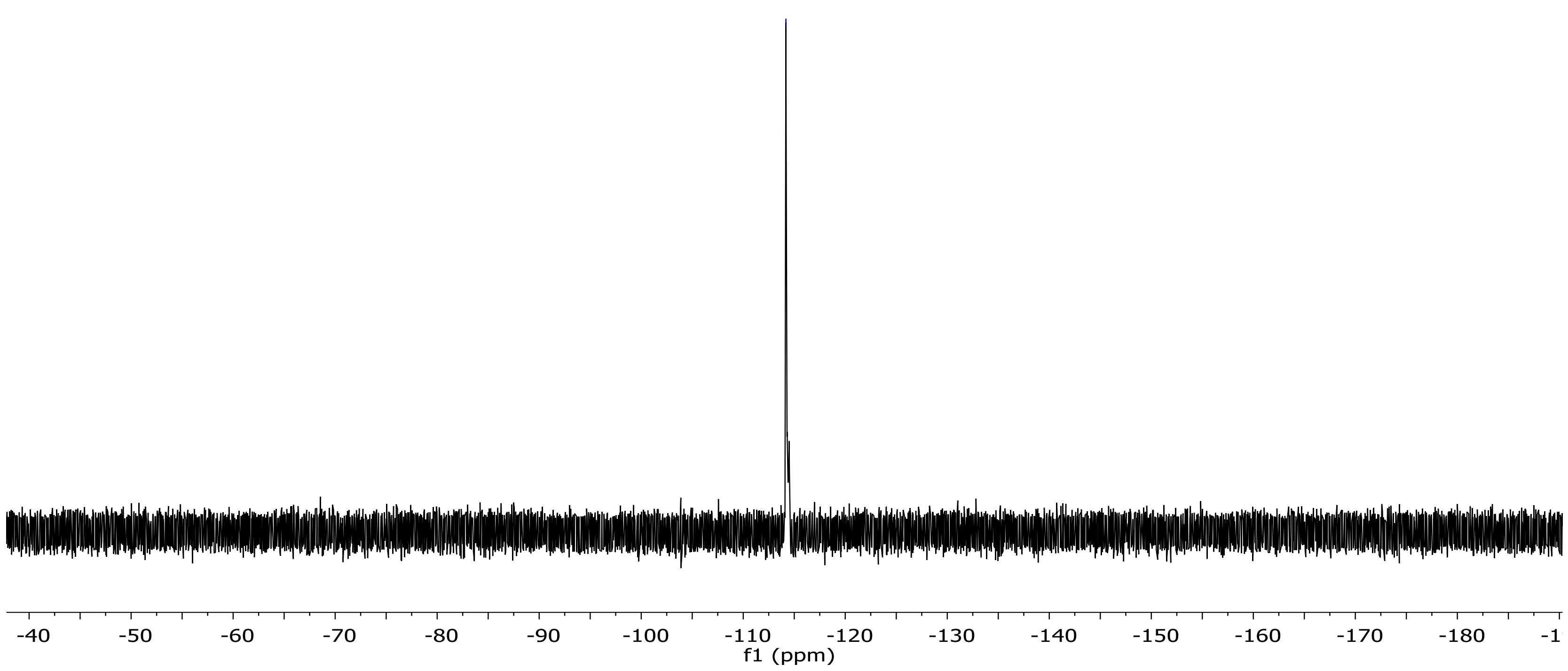

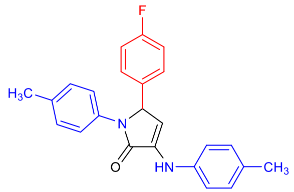

4f

*5-(p-Nitrophenyl)-1-(p-tolyl)-3-(p-tolylamino)-1H-pyrrol-2(5H)-one (**4g**).*

¹H NMR (400 MHz, $CDCl_3$).

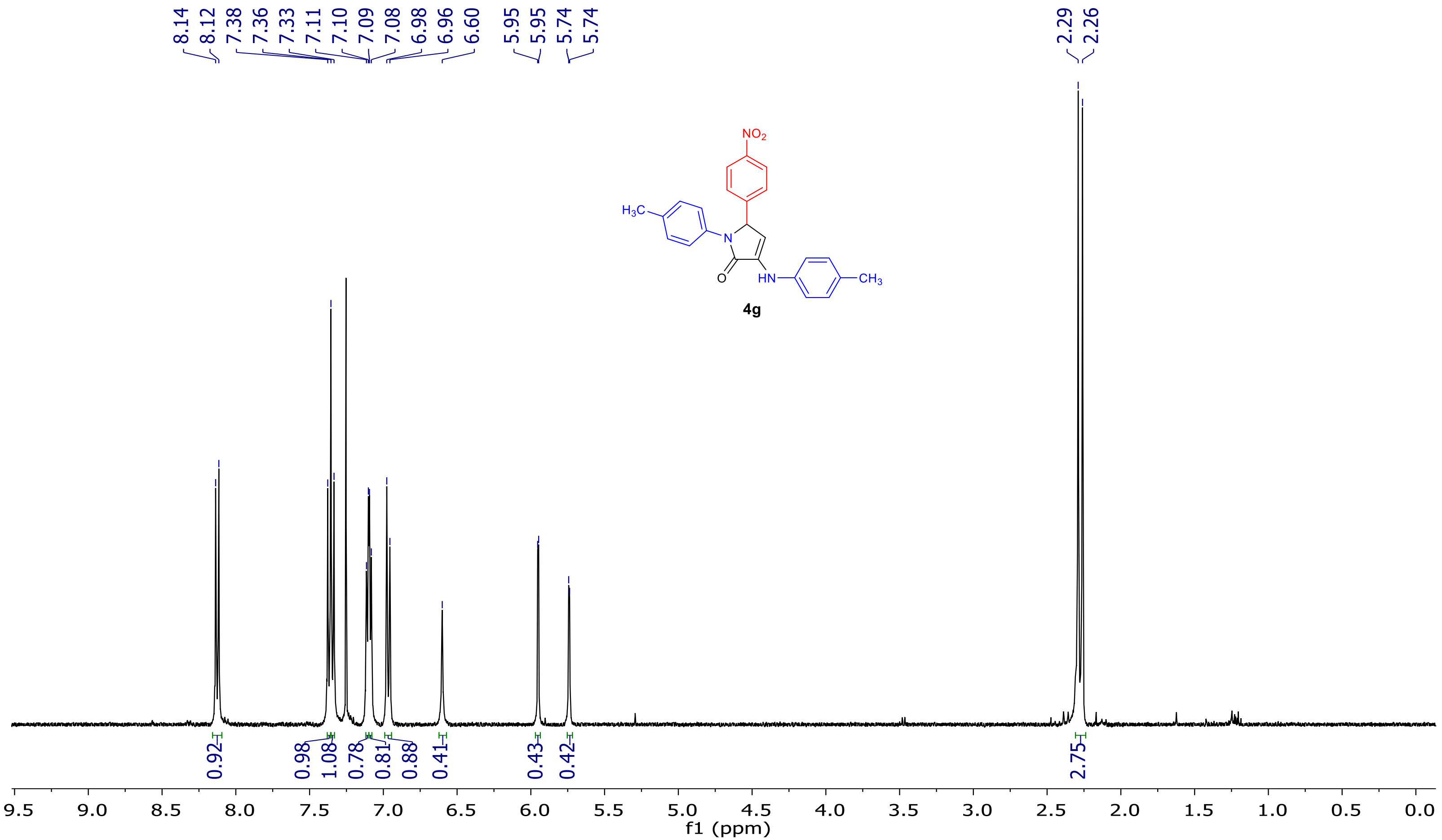

^{13}C NMR (100 MHz, CDCl3)

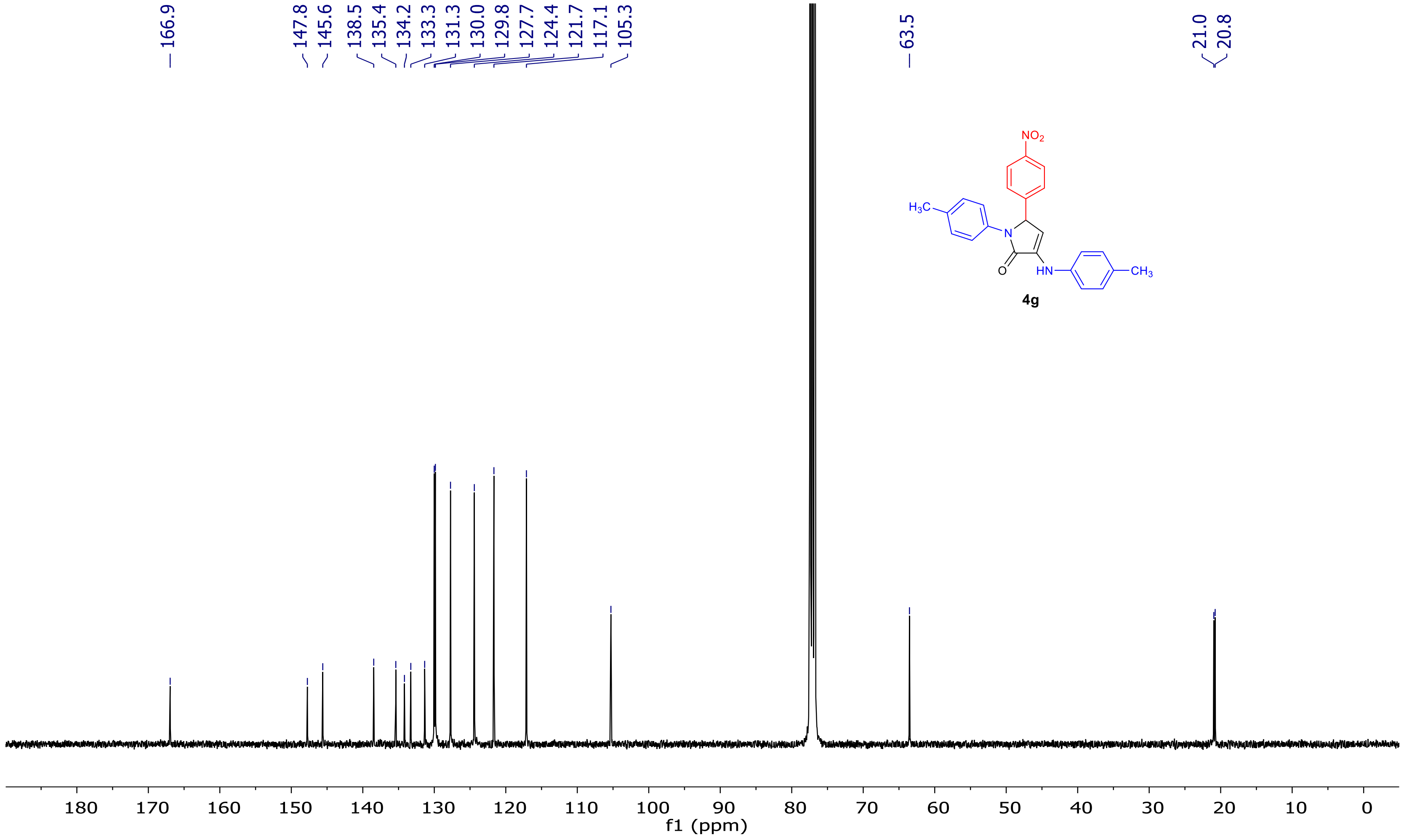

*5-(m-Nitrophenyl)-1-(p-tolyl)-3-(p-tolylamino)-1,5-dihydro-2H-pyrrol-2-one (**4h**).*

¹H NMR (400 MHz, $CDCl_3$)

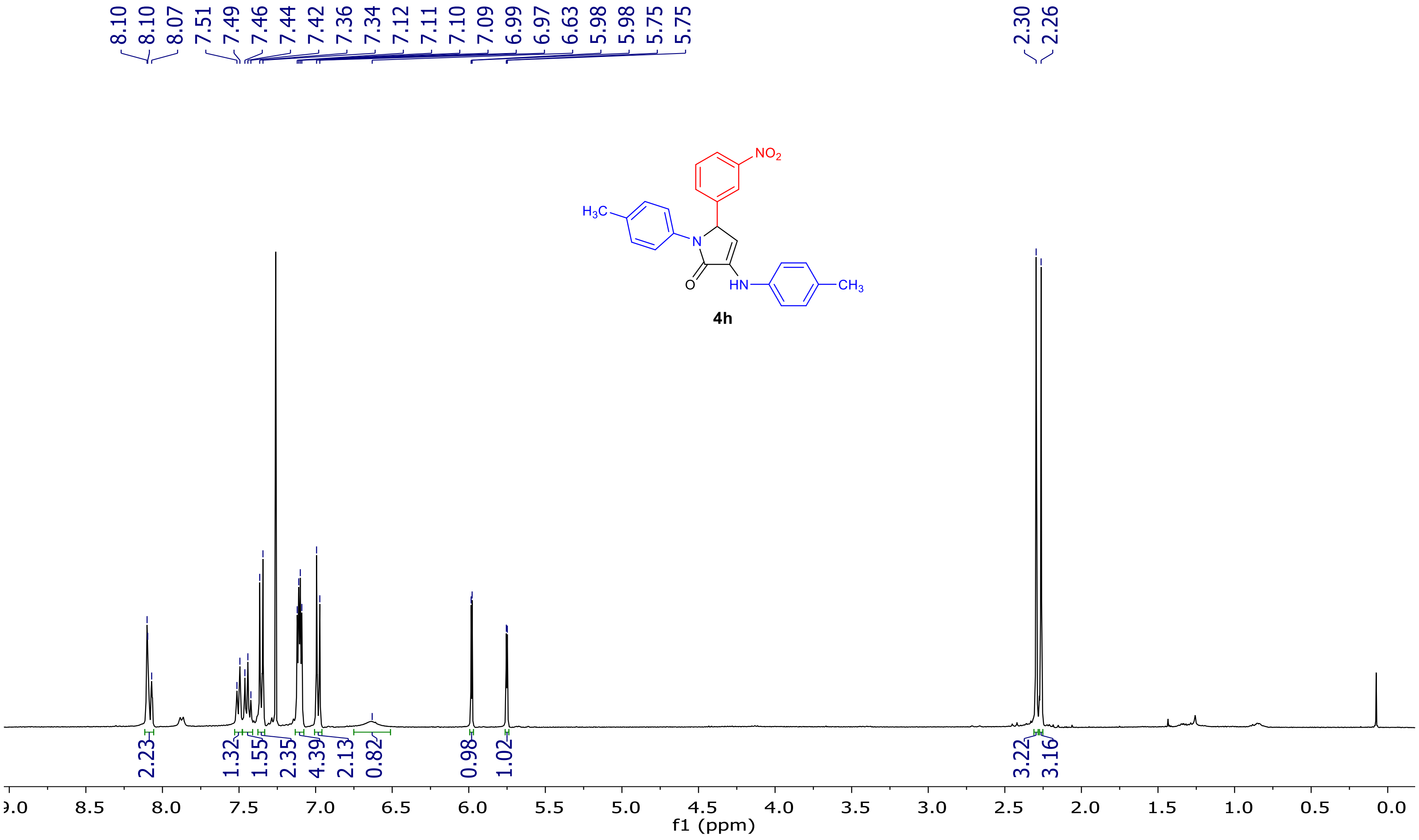

^{13}C NMR (100 MHz, $CDCl_3$)

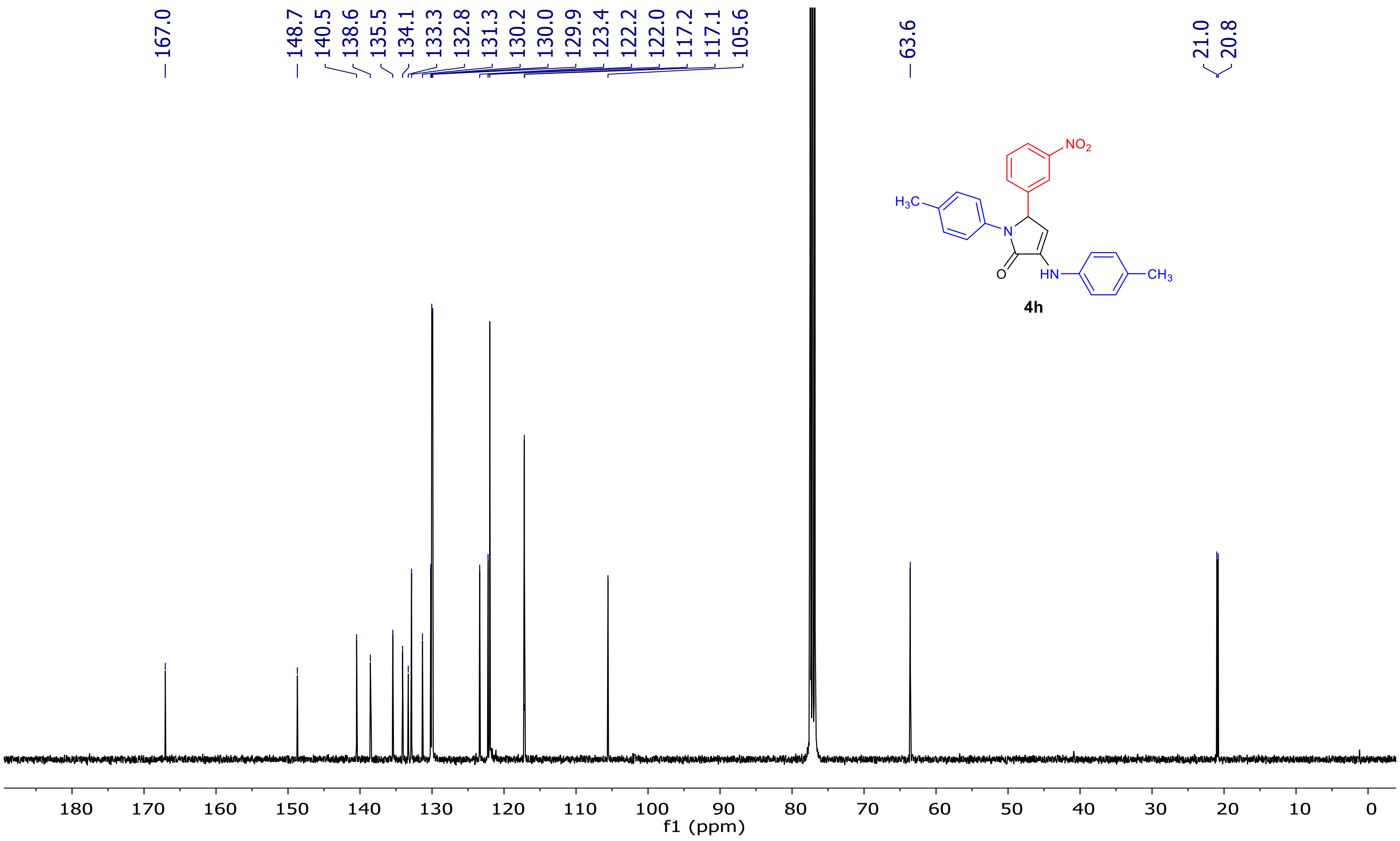

*1-(p-Tolyl)-3-(p-tolylamino)-5-(p-(trifluoromethyl)phenyl)-1,5-dihydro-2H-pyrrol-2-one (**4i**).*

¹H NMR (400 MHz, CDCl₃)

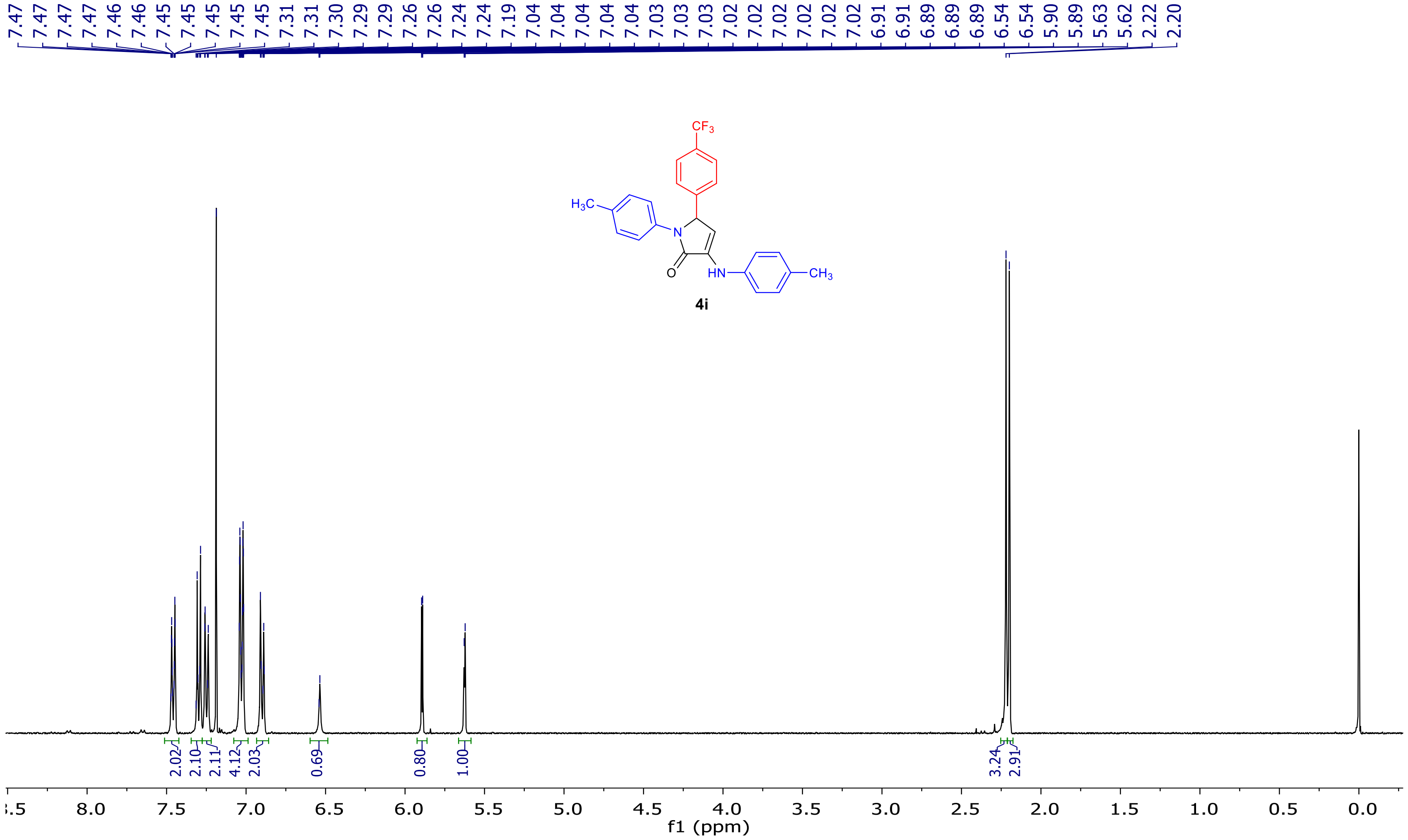

^{13}C NMR (100 MHz, $CDCl_3$)

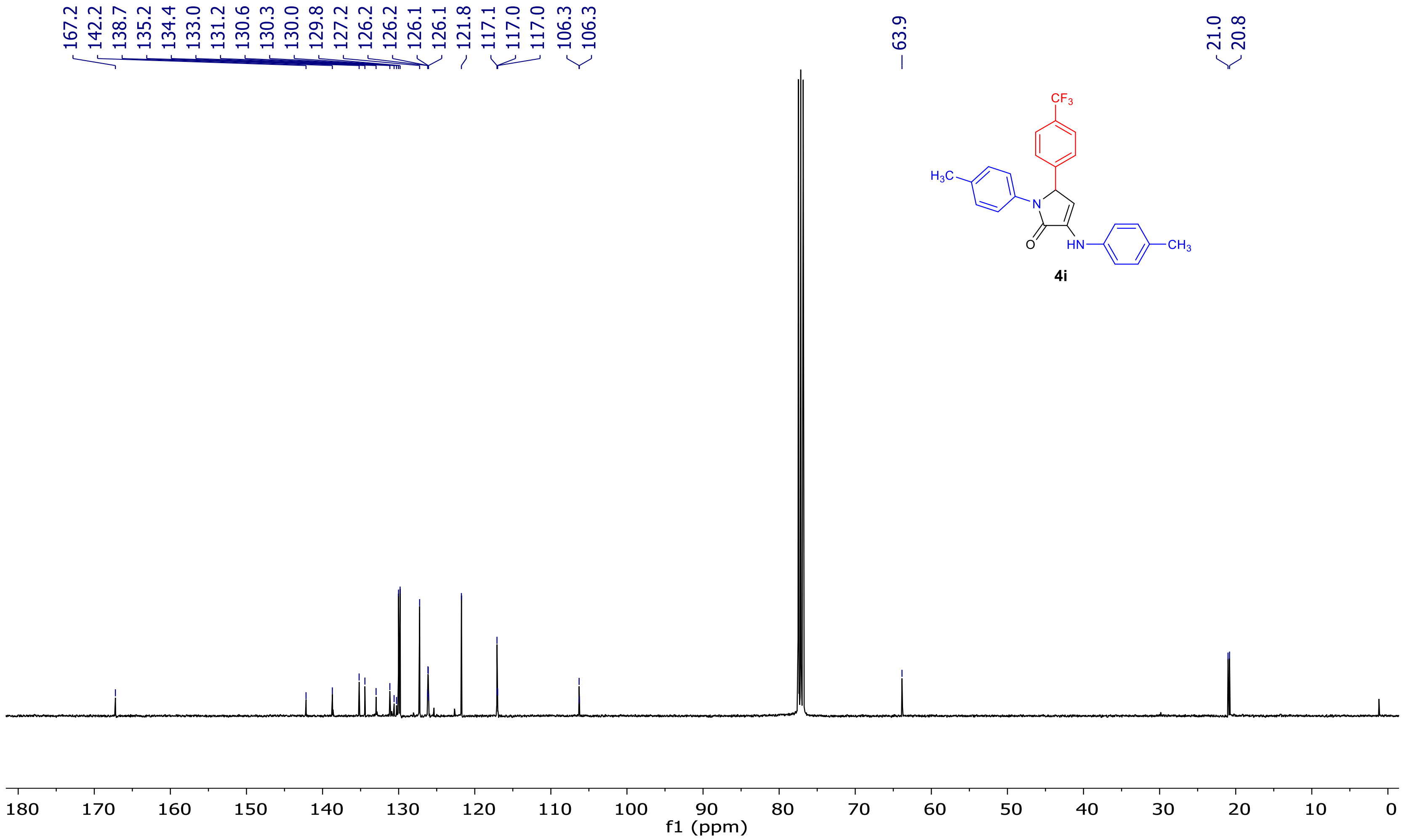

^{19}F NMR (282 MHz, $CDCl_3$)

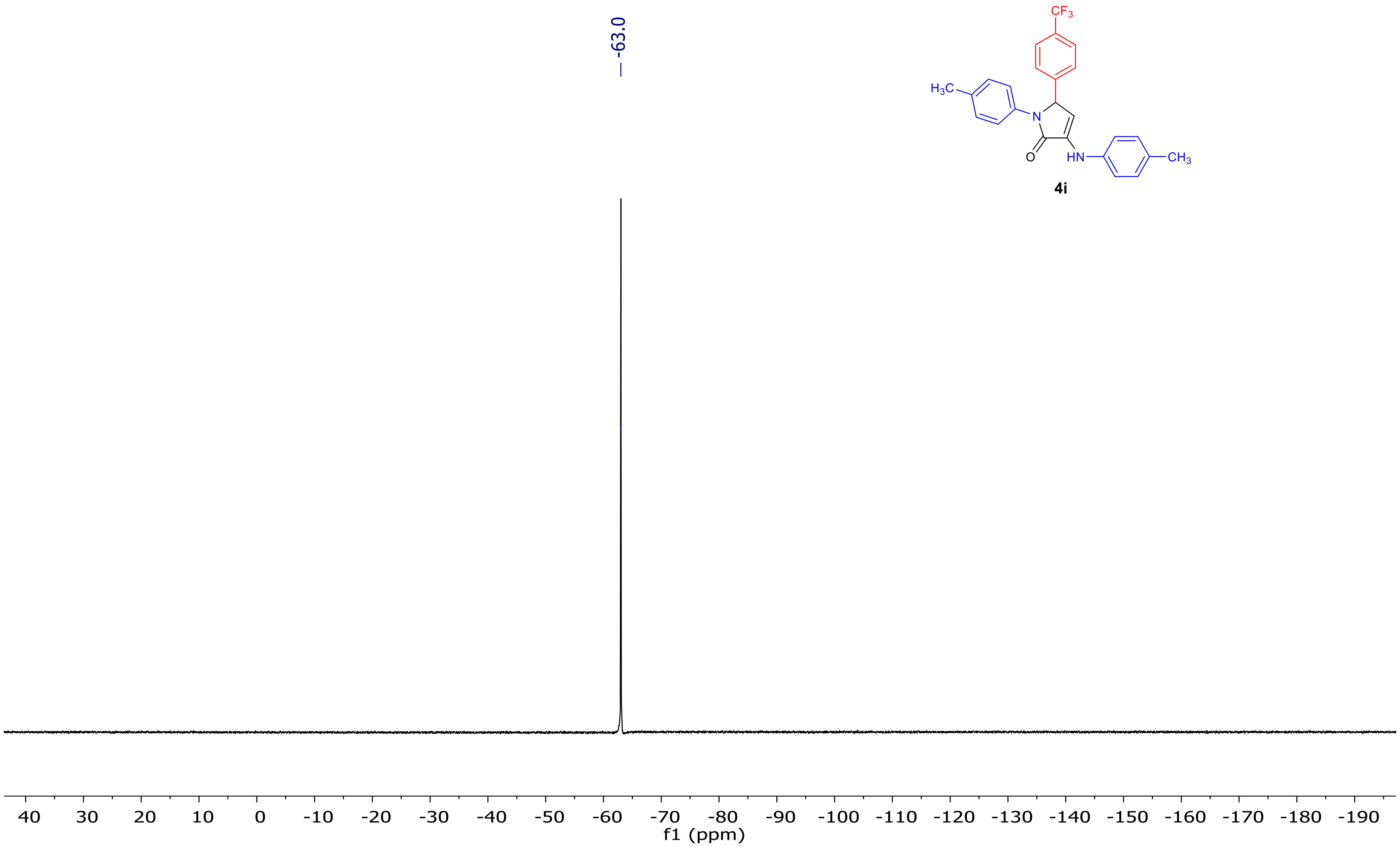

4i

*5-(1-Methyl-1H-indol-6-yl)-1-(p-tolyl)-3-(p-tolylamino)-1,5-dihydro-2H-pyrrol-2-one (**4j**).*

^{1}H NMR (400 MHz, $CDCl_3$)

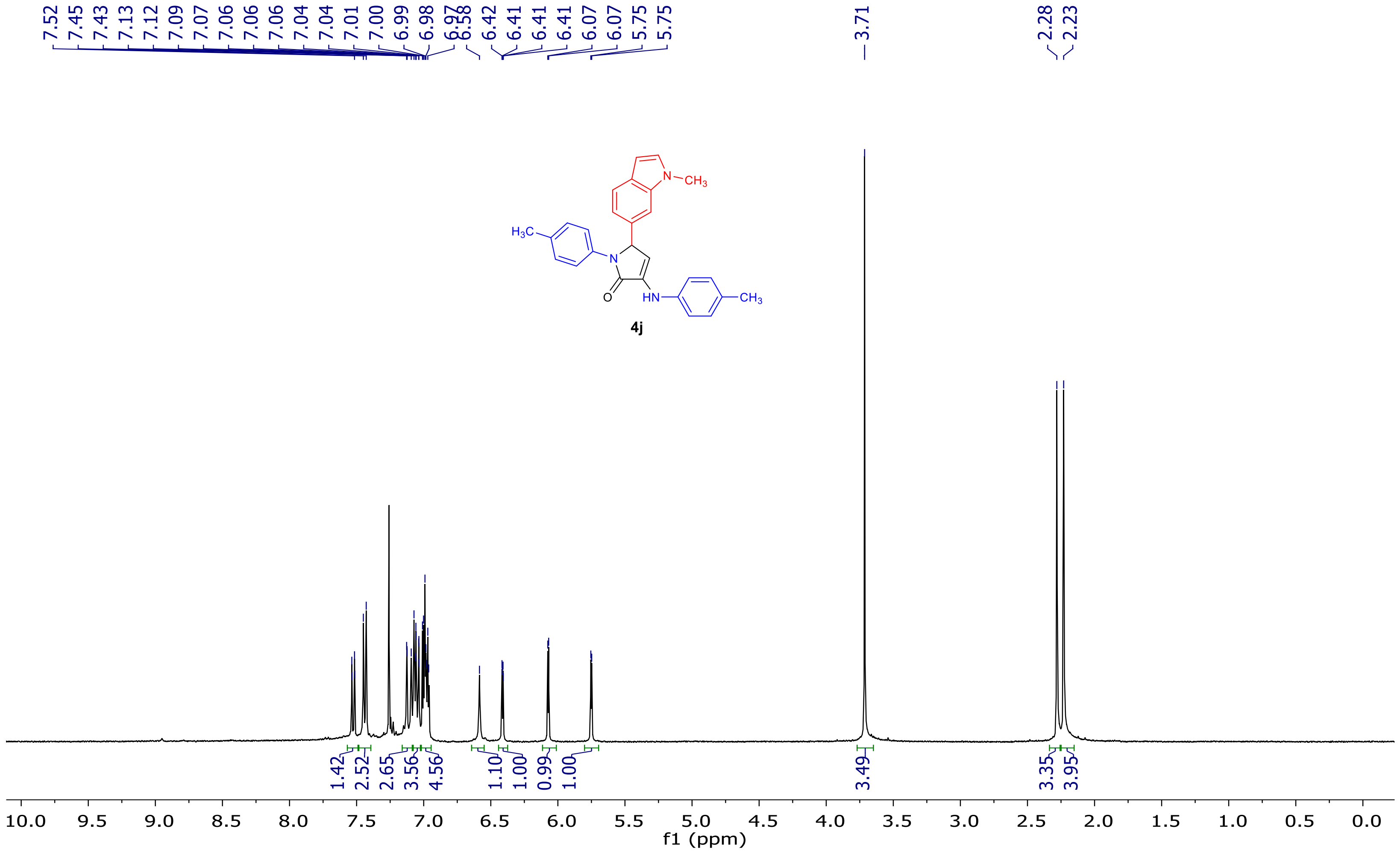

^{13}C NMR (100 MHz, $CDCl_3$)

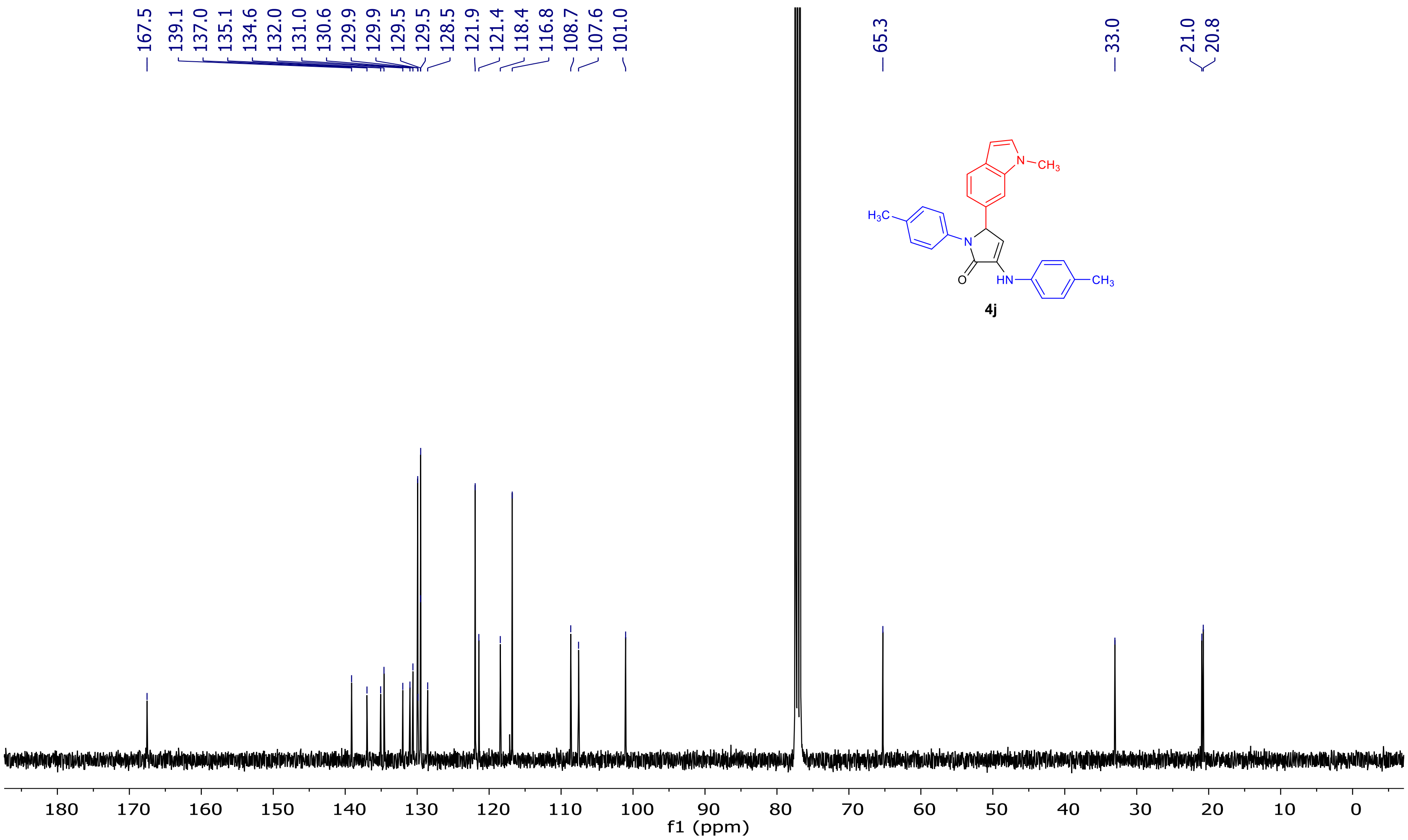

*5-(Furan-2-yl)-1-(p-tolyl)-3-(p-tolylamino)-1,5-dihydro-2H-pyrrol-2-one (**4k**).*

^{1}H NMR (400 MHz, $CDCl_3$)

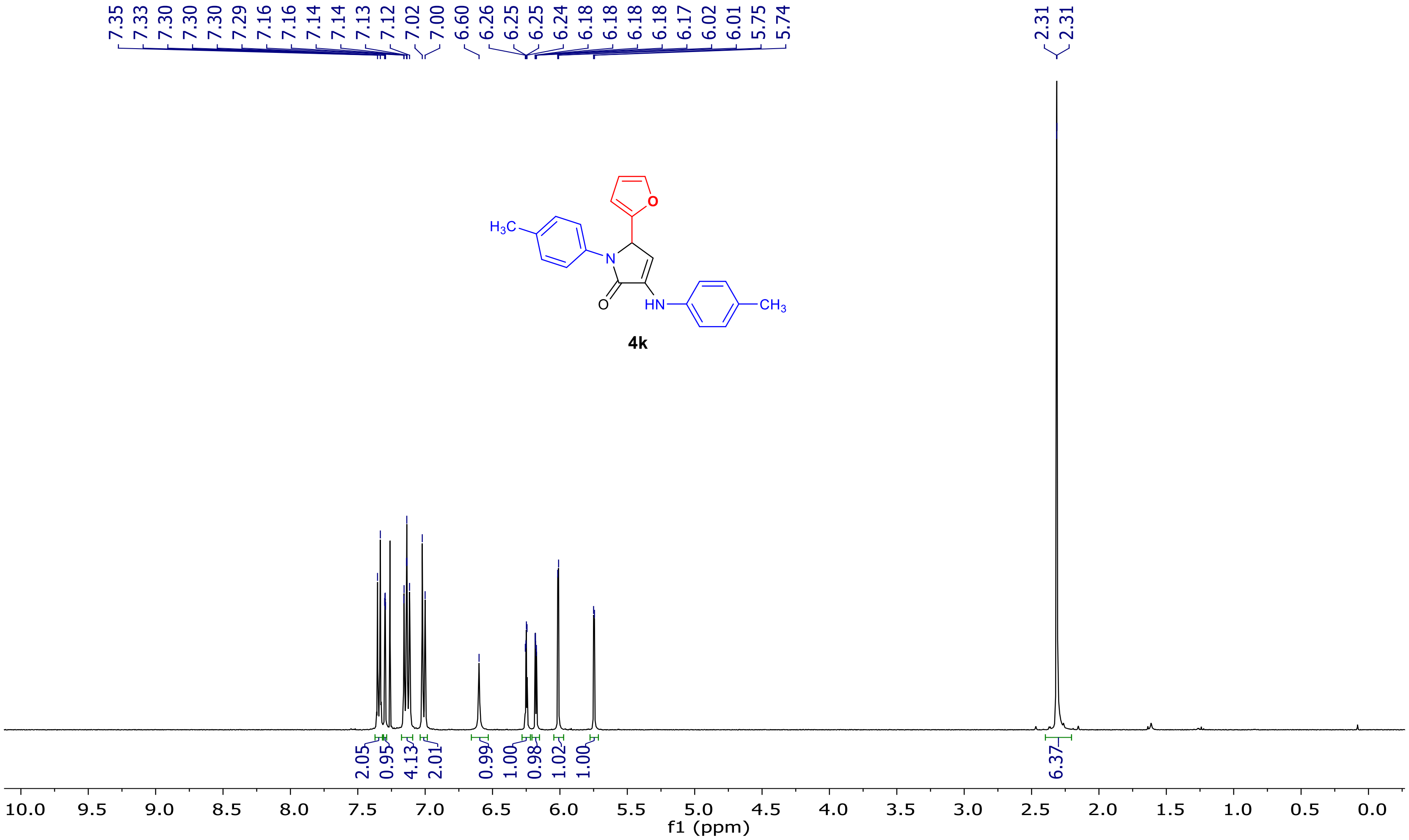

^{13}C NMR (101 MHz, $CDCl_3$)

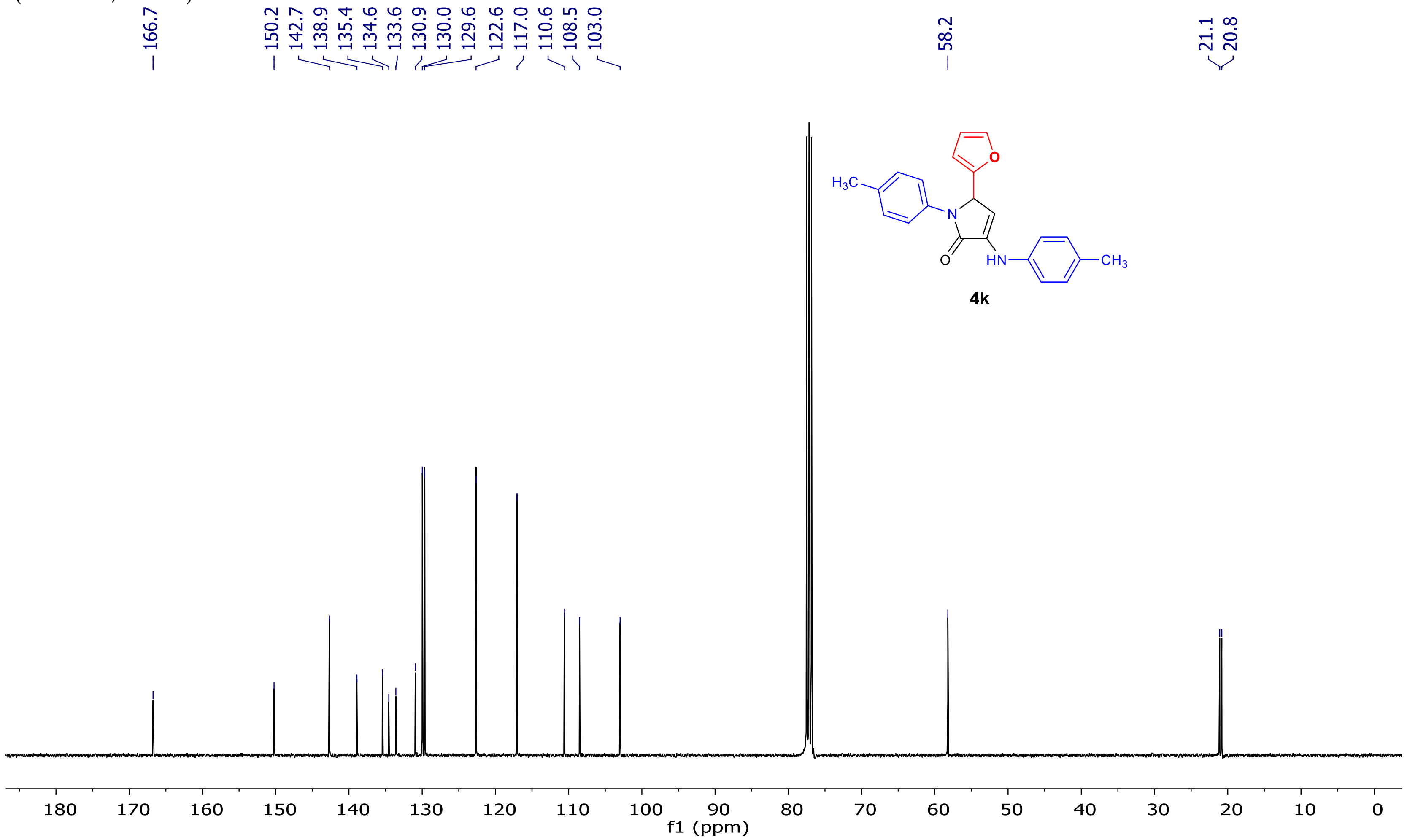

*5-(Thiophen-2-yl)-1-(p-tolyl)-3-(p-tolylamino)-1,5-dihydro-2H-pyrrol-2-one (**4l**).*

^{1}H NMR (300 MHz, $CDCl_3$)

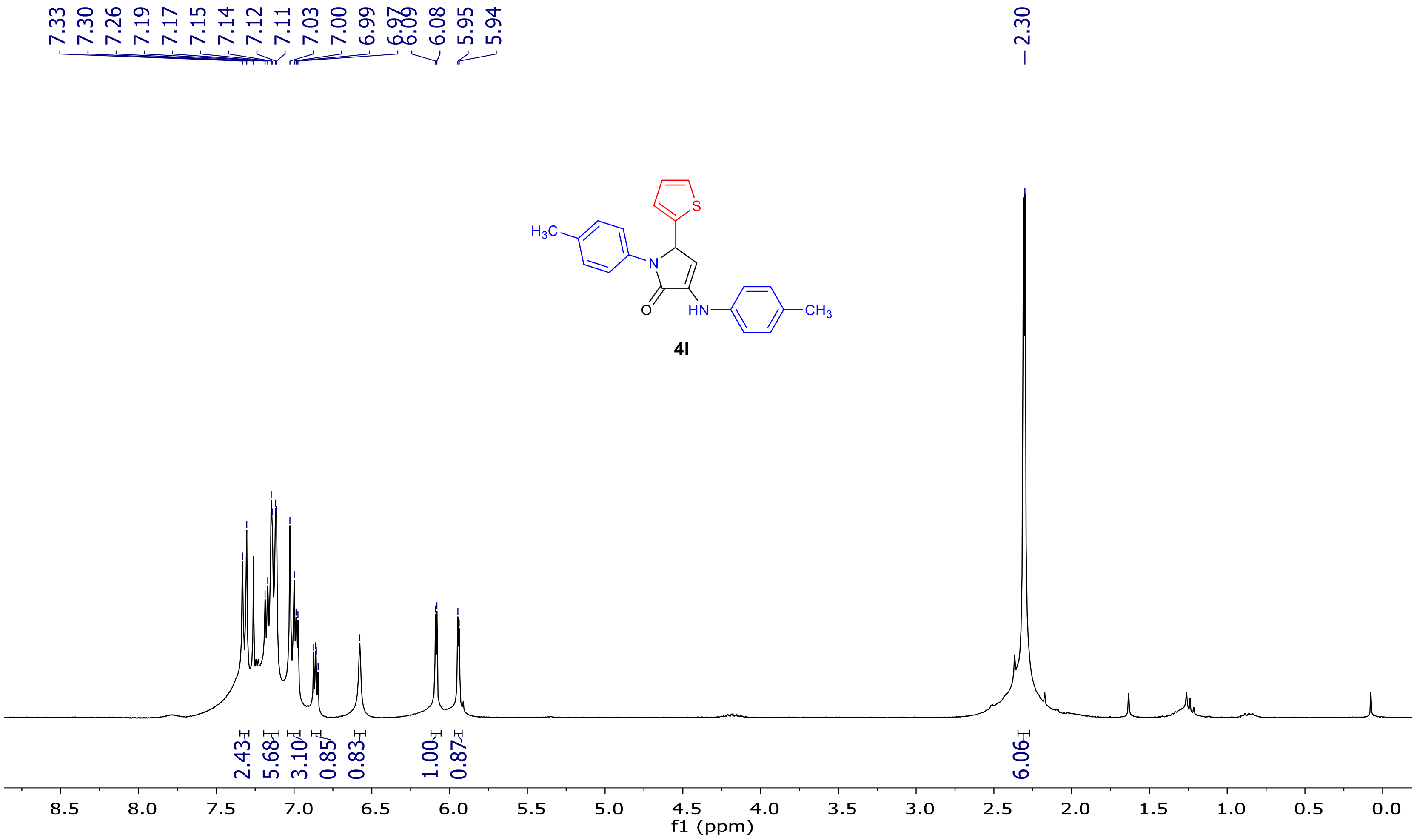

13C NMR (75 MHz, CDCl3)

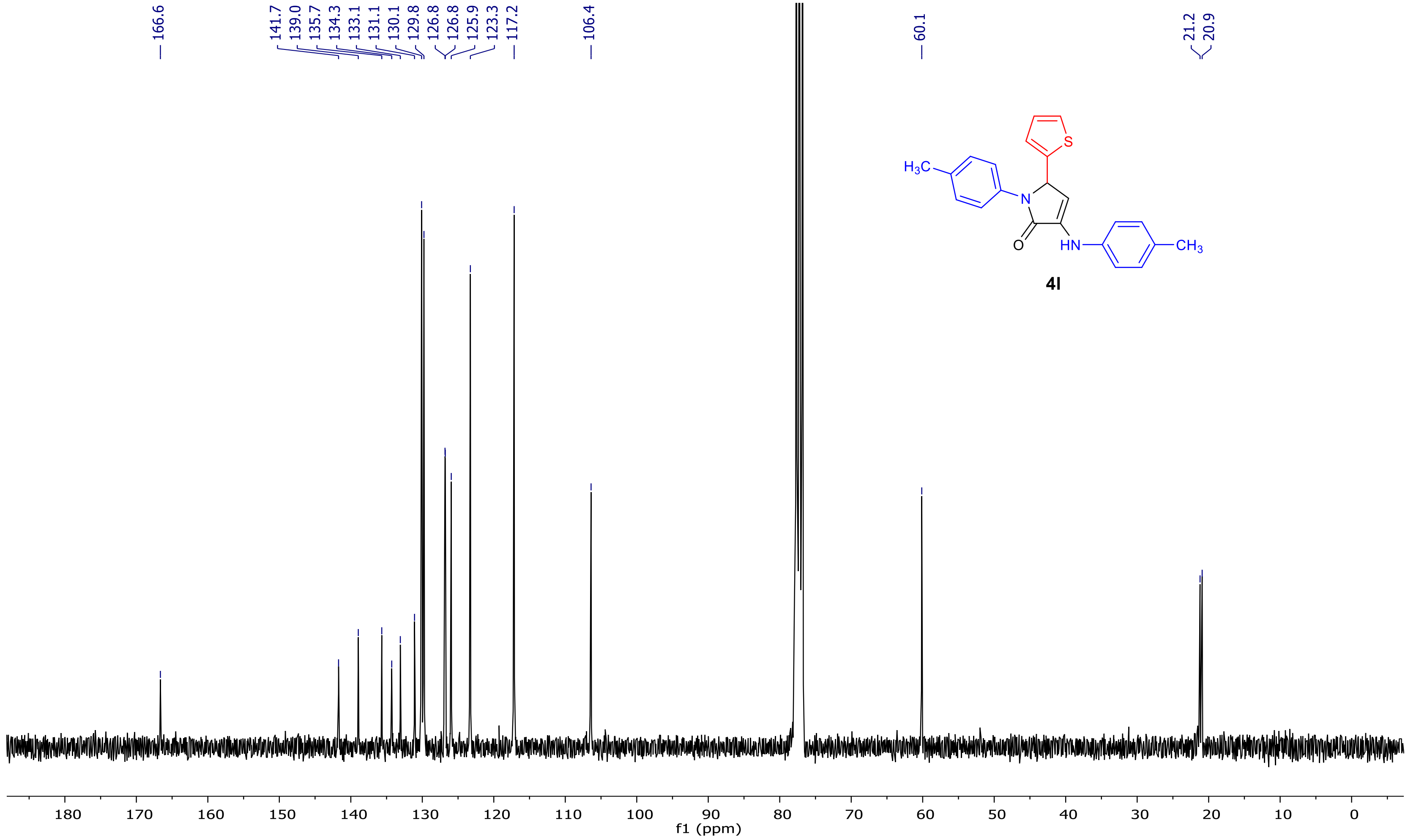

*5-(Naphthalen-2-yl)-1-(p-tolyl)-3-(p-tolylamino)-1,5-dihydro-2H-pyrrol-2-one (**4m**).*

¹H NMR (400 MHz, $CDCl_3$)

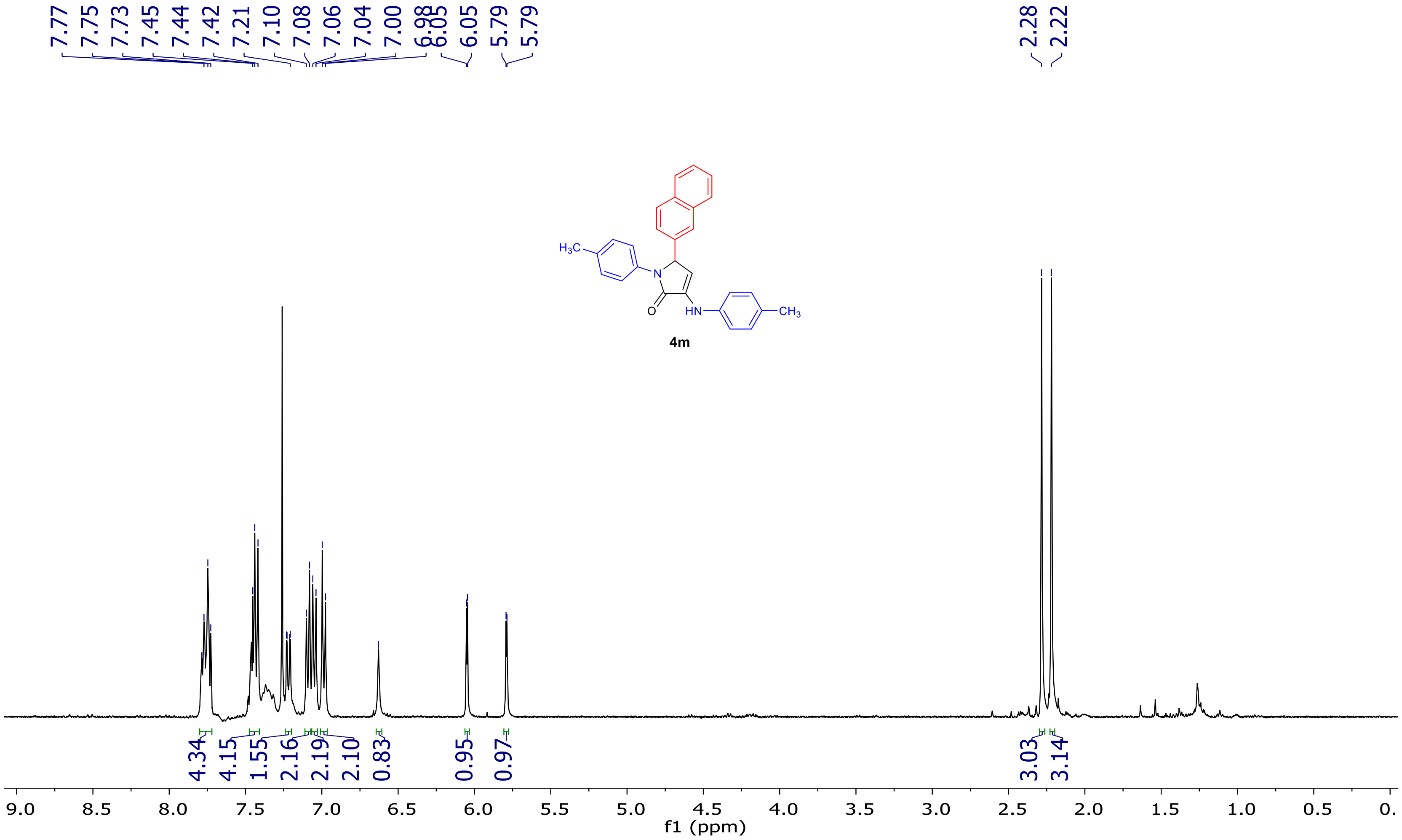

^{13}**C NMR** (101 MHz, $CDCl_3$)

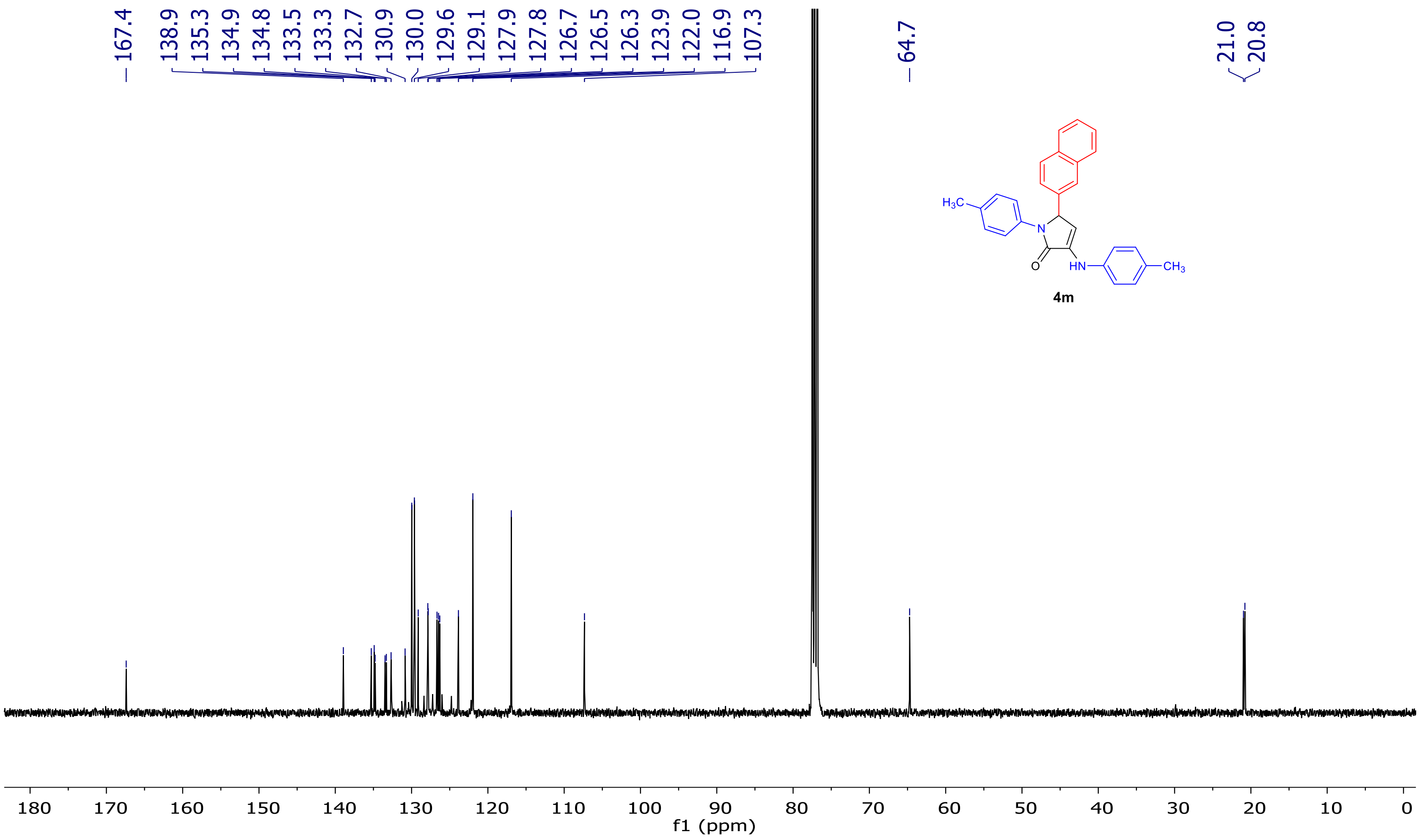

*5-Methyl-1-(p-tolyl)-3-(p-tolylamino)-1H-pyrrol-2(5H)-one (**4n**).*

^{1}H NMR (400 MHz, $CDCl_3$)

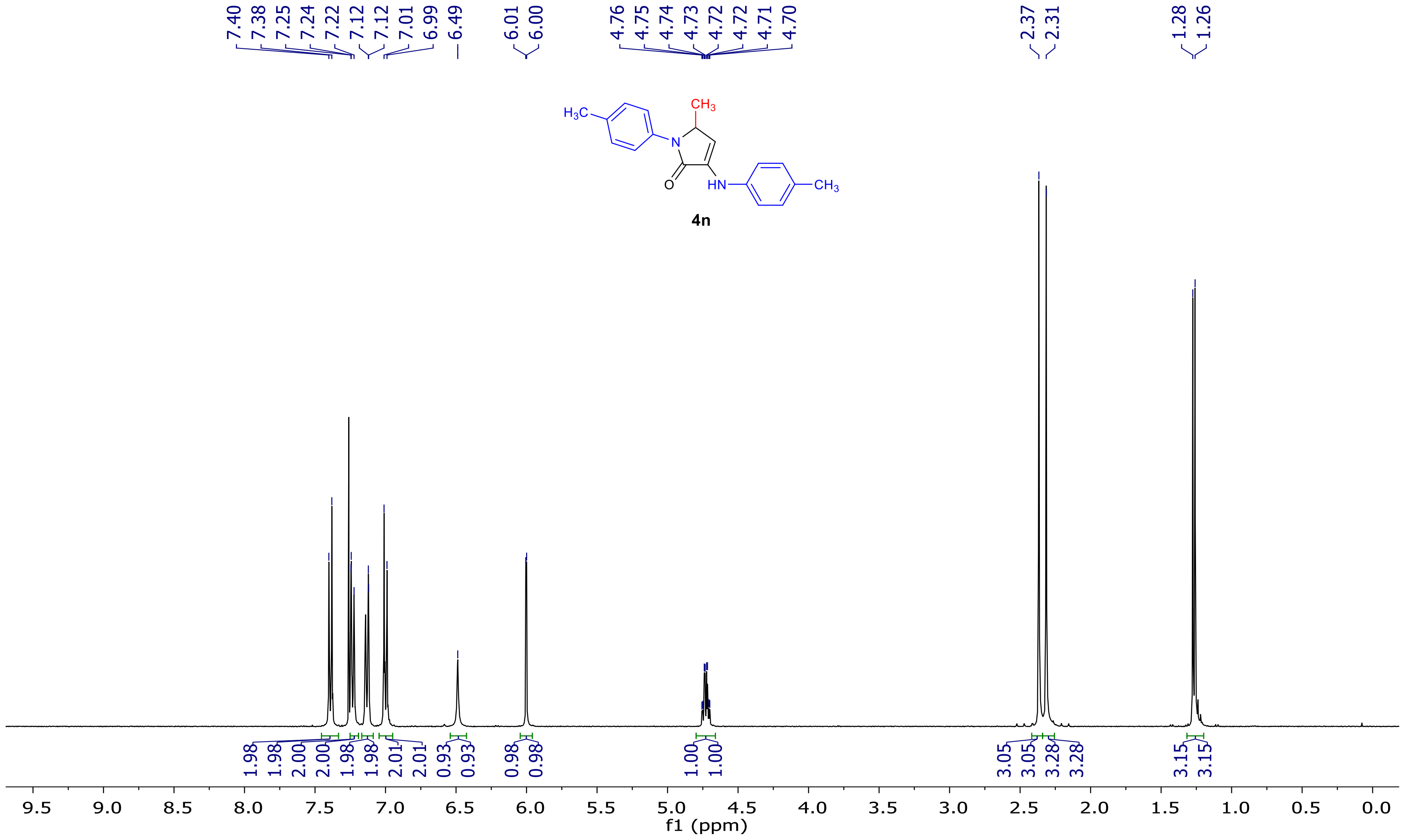

^{13}C NMR (101 MHz, $CDCl_3$)

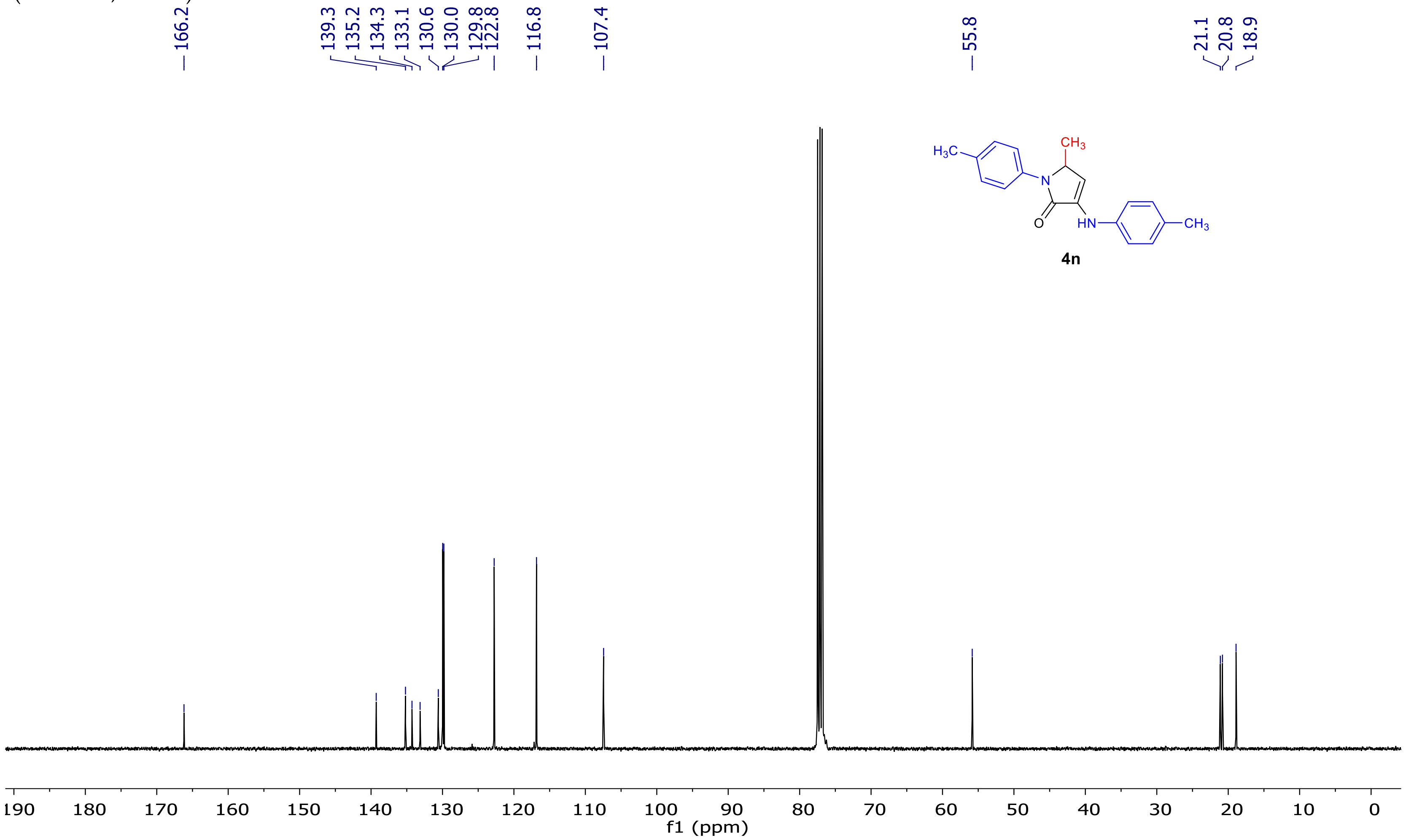

*5-Iso-propyl-1,3-di-p-tolyl-1H-pyrrol-2(5H)-one (**4o**).*

¹H NMR (400 MHz, CDCl3)

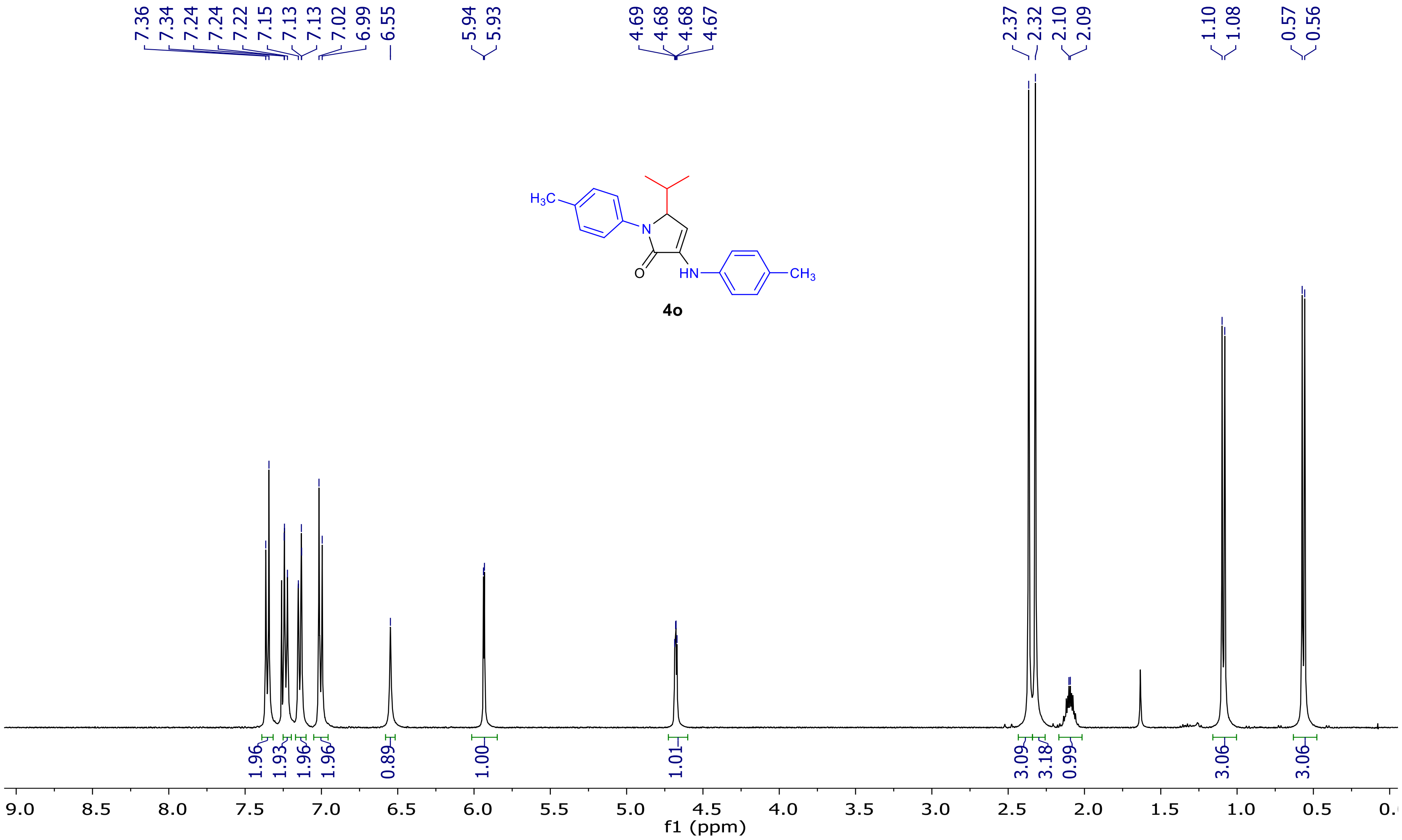

¹³C NMR (75 MHz, $CDCl_3$)

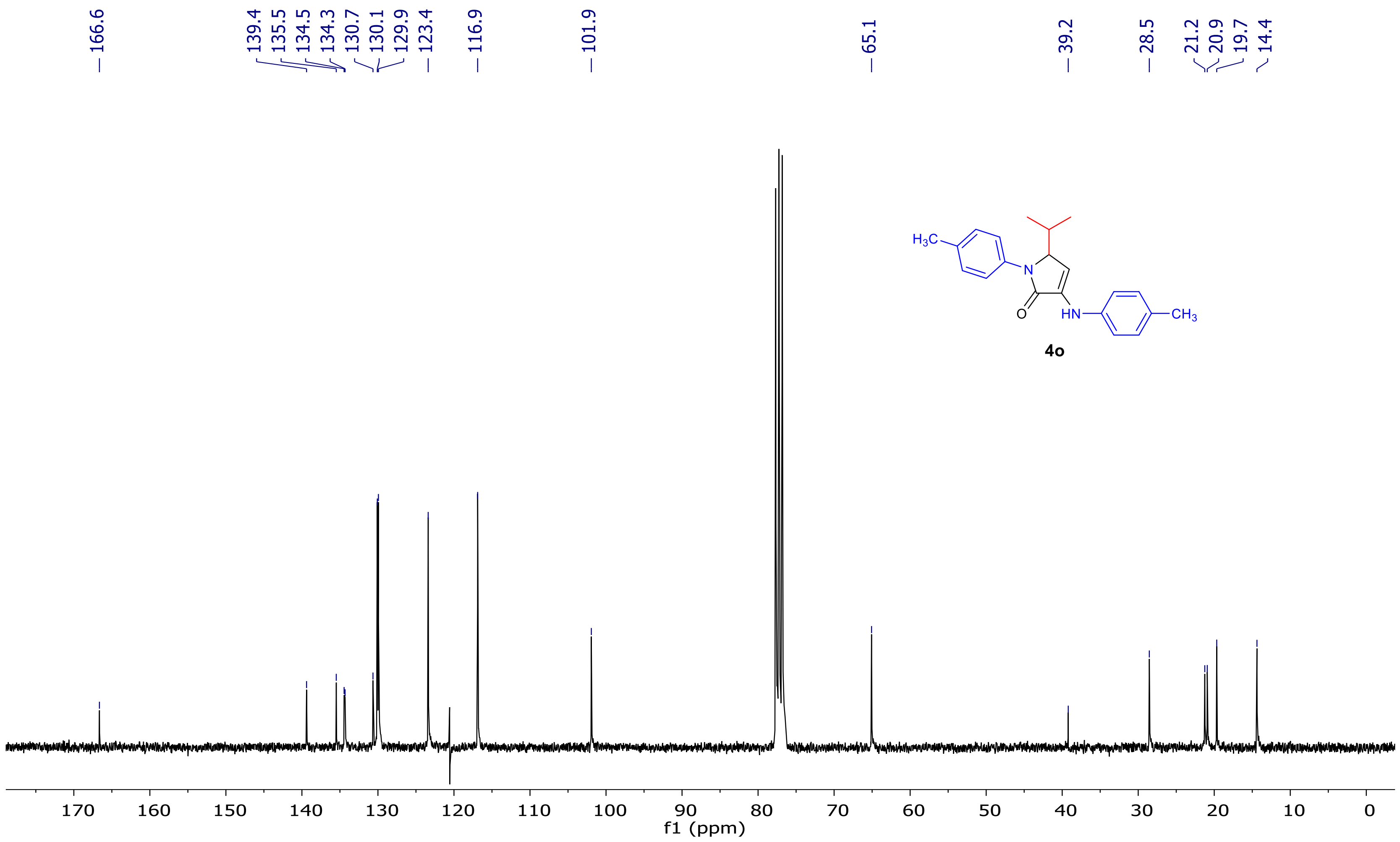

*5-Iso-butyl-1-(p-tolyl)-3-(p-tolylamino)-1,5-dihydro-2H-pyrrol-2-one (**4p**).*

^{1}H NMR (400 MHz, $CDCl_3$)

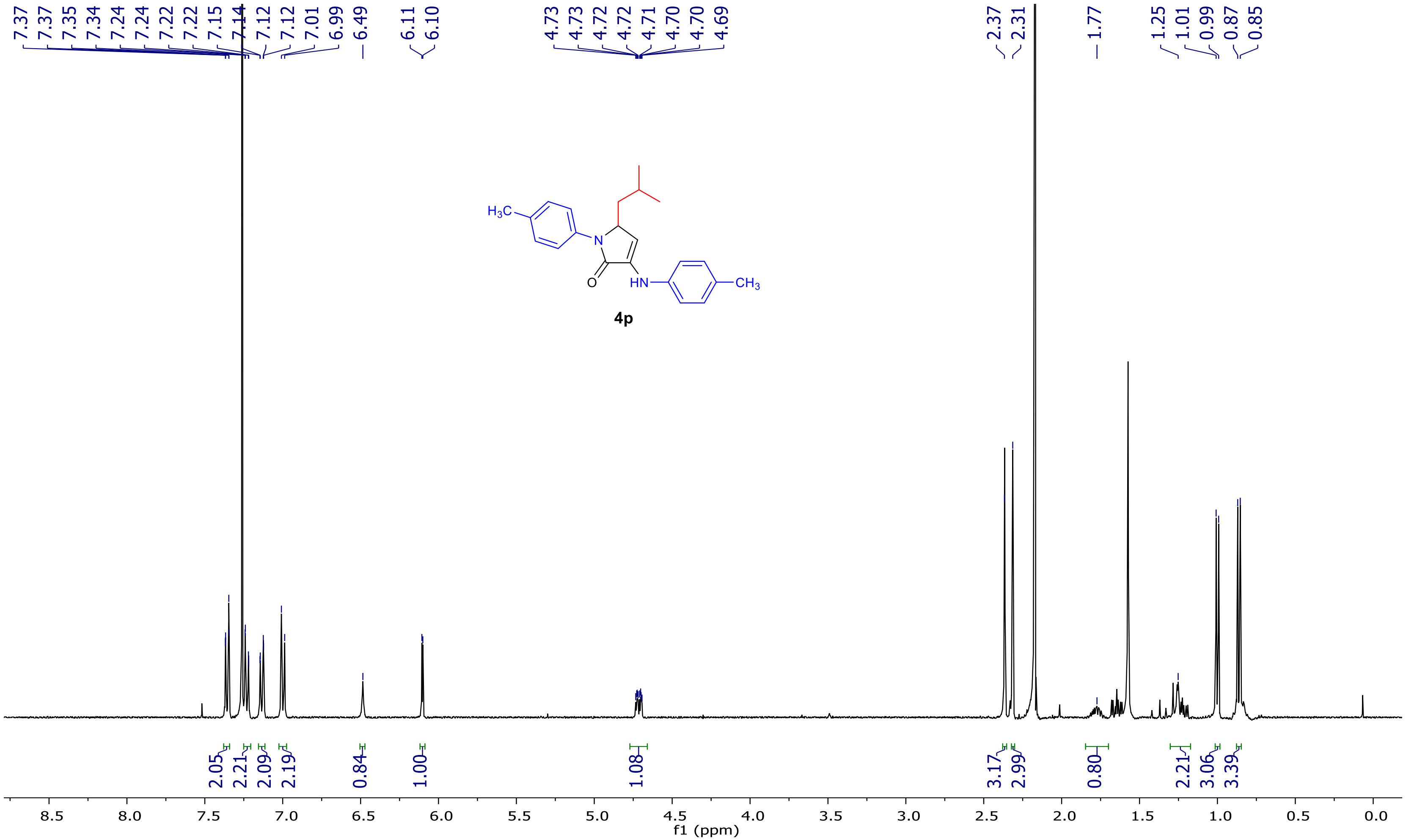

^{13}C NMR (101 MHz, $CDCl_3$)

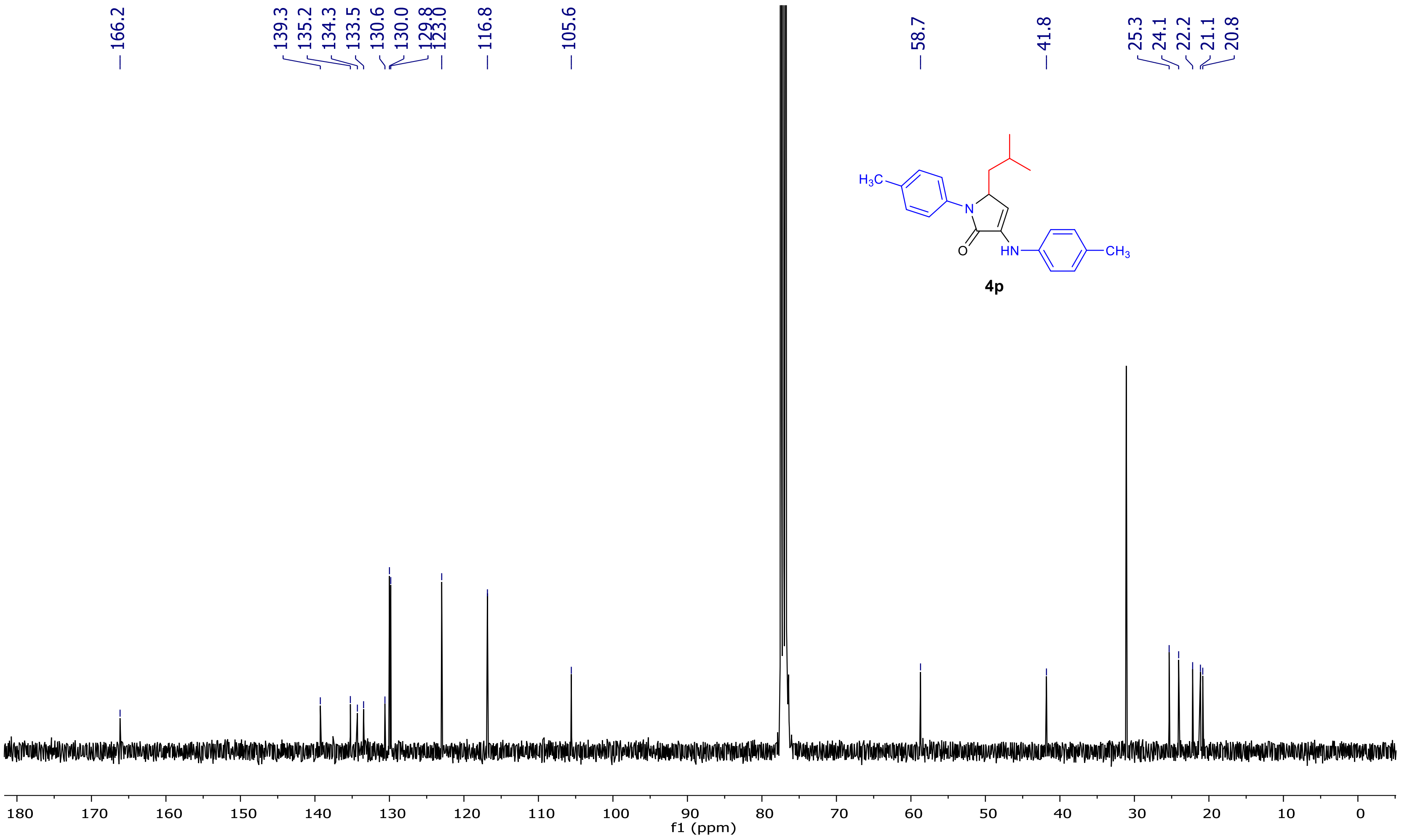

*1-Cyclohexyl-5-(p-tolyl)-3-(p-tolylamino)-1,5-dihydro-2H-pyrrol-2-one (**4q**).*

^{1}H NMR (400 MHz, $CDCl_3$)

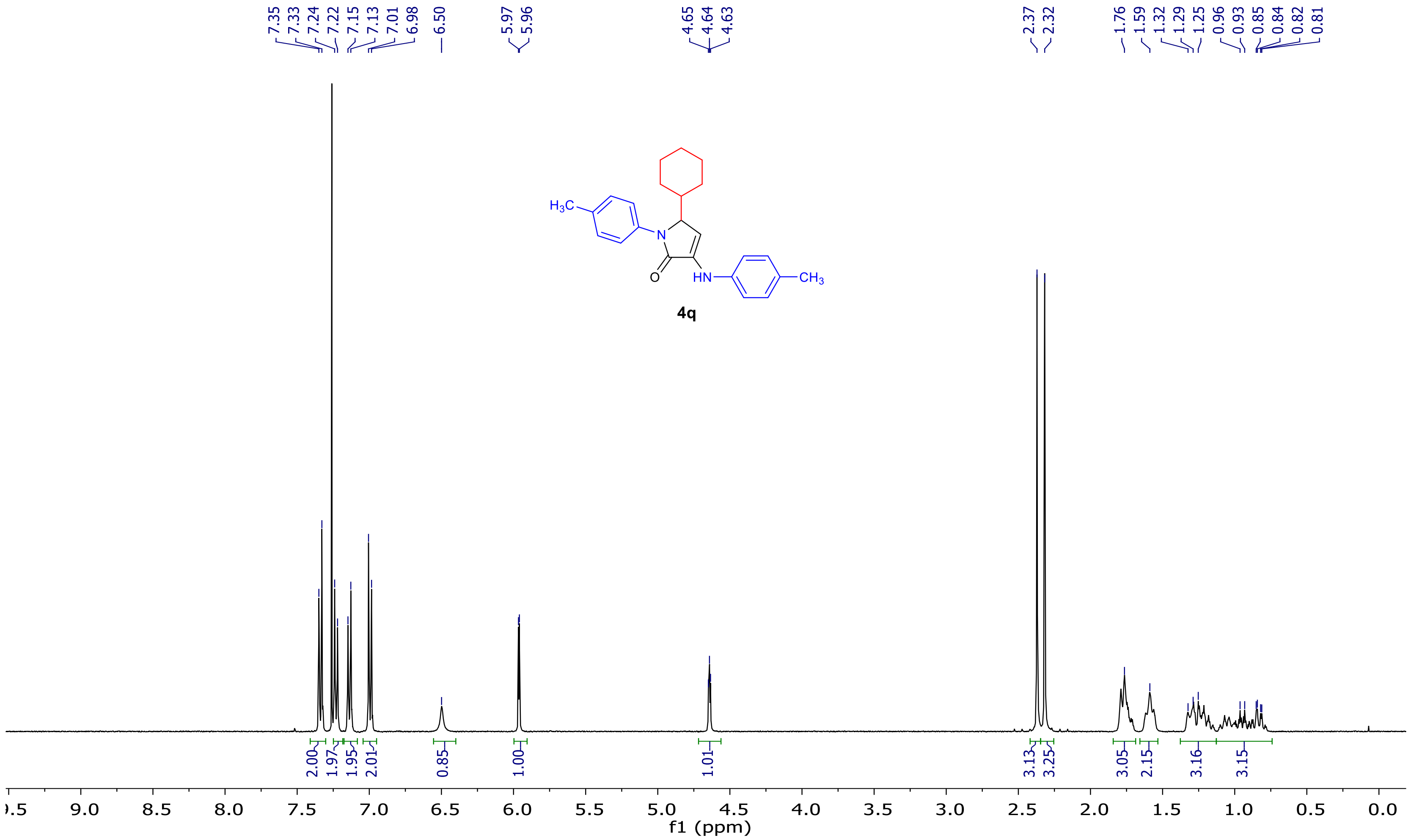

^{13}C NMR (101 MHz, $CDCl_3$)

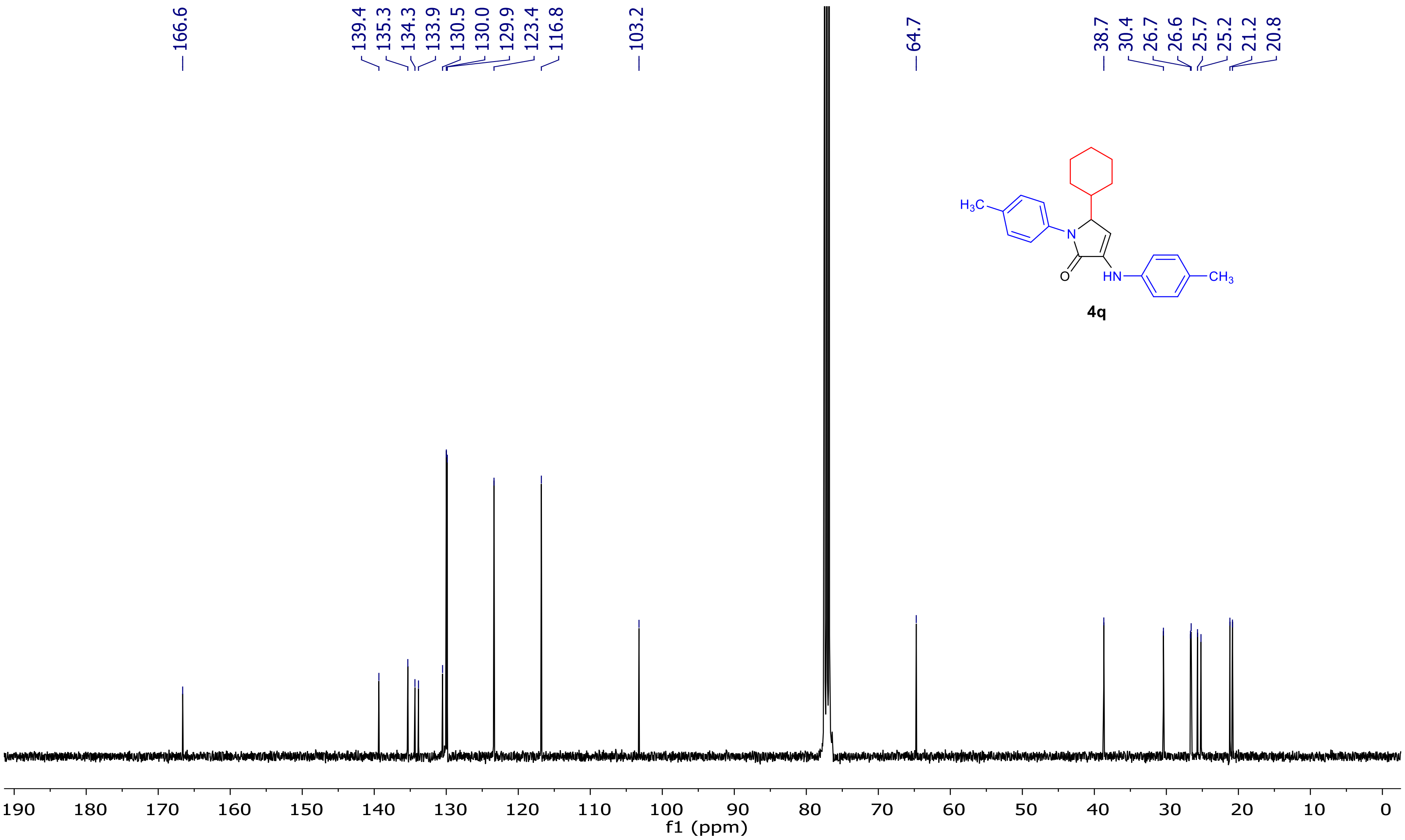

4q

(E)-5-Styryl-1-(p-tolyl)-3-(p-tolylamino)-1,5-dihydro-2H-pyrrol-2-one (**4r**).

^{1}H NMR (300 MHz, $CDCl_3$)

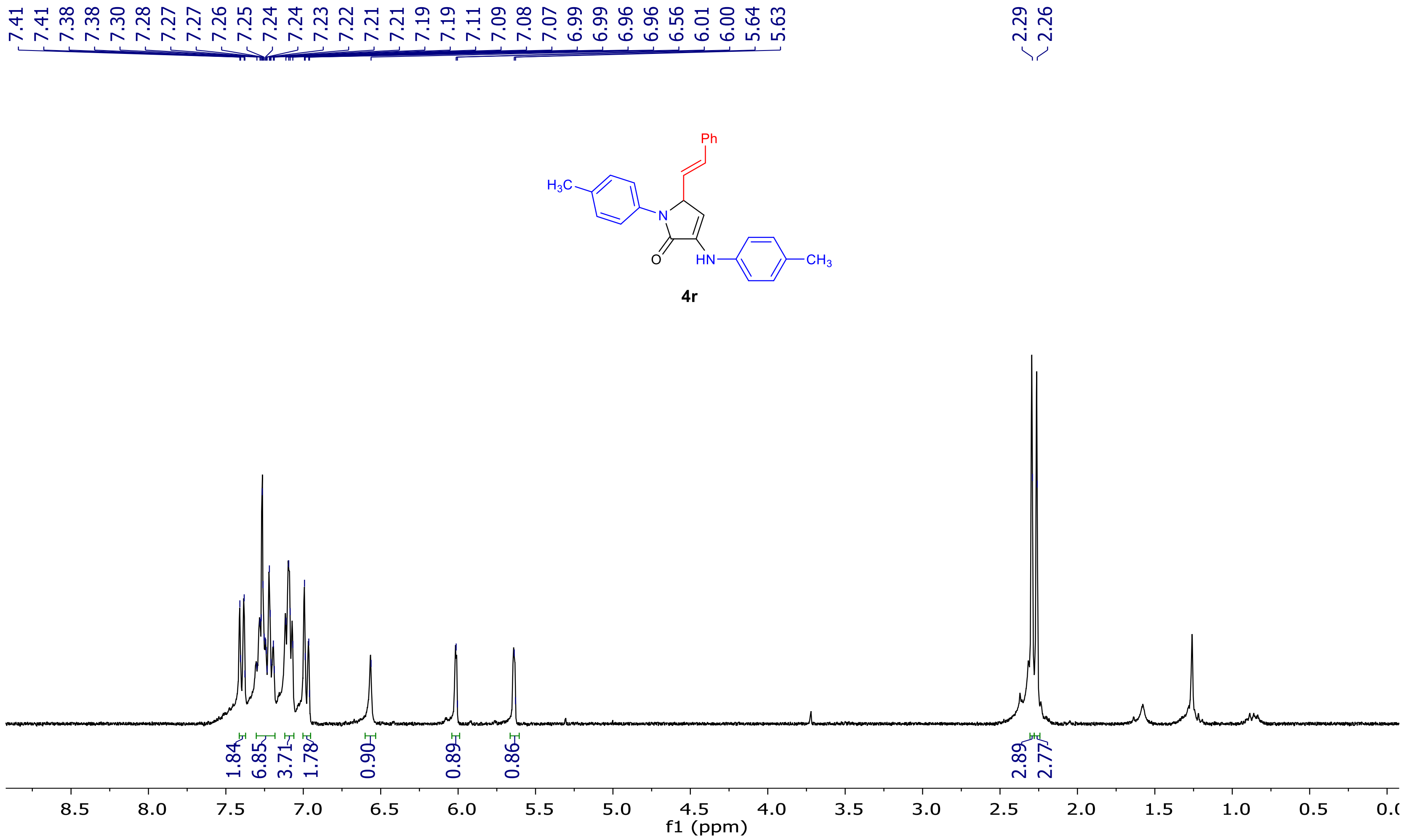

^{31}**C NMR** (75 MHz, $CDCl_3$)

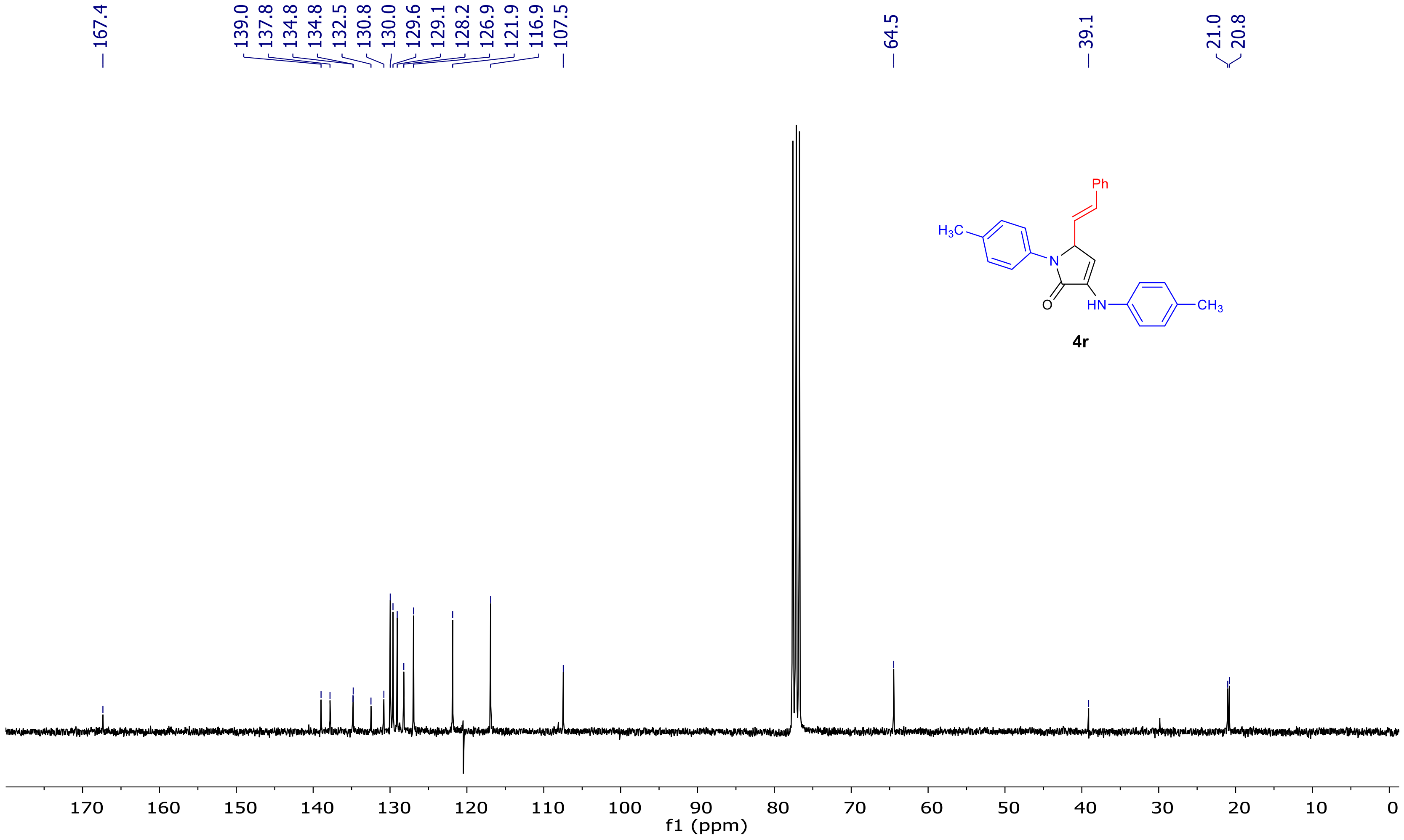

*Ethyl 5-oxo-1, 4-di-p-tolyl-2,5-dihydro-1H-pyrrol-2-carboxylate (**4s**).*

^{1}H NMR (400 MHz, $CDCl_3$)

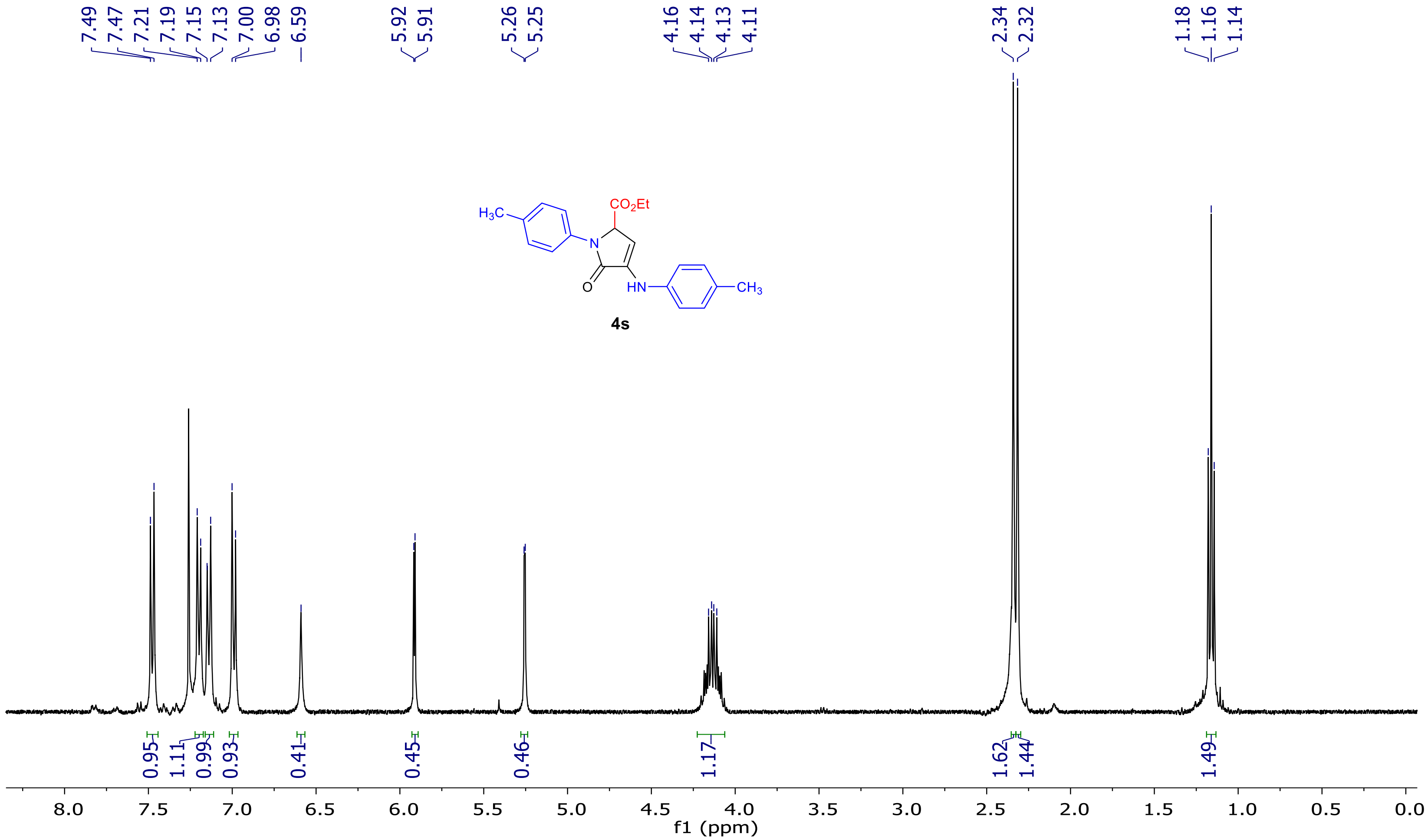

^{13}C NMR (100 MHz, $CDCl_3$)

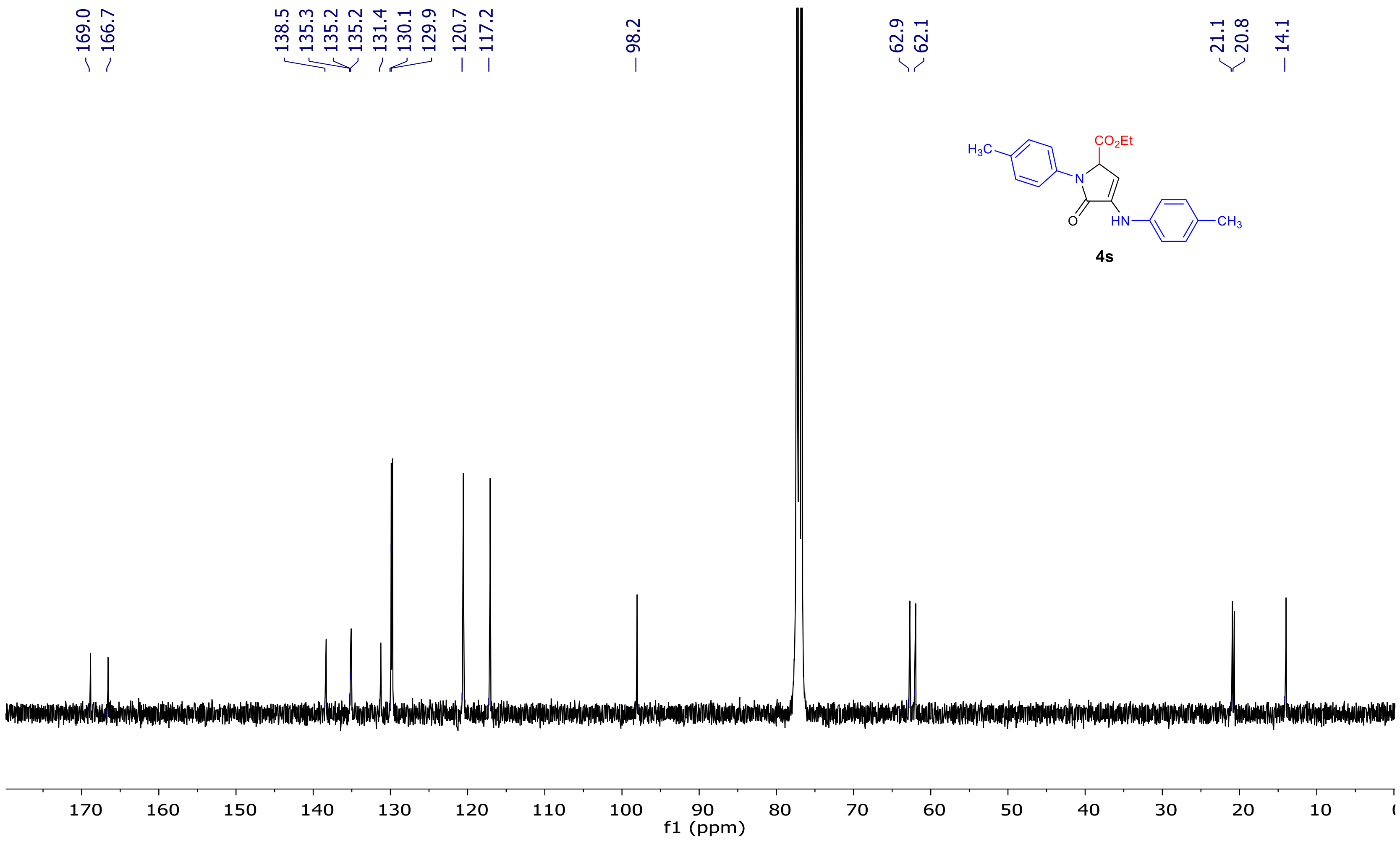

*5-(Perfluorophenyl)-1-(p-tolyl-3-(p-tolylamino)-1H-pyrrol-2(5H)-one (**4t**).*

[1]**H NMR** (400 MHz, $CDCl_3$)

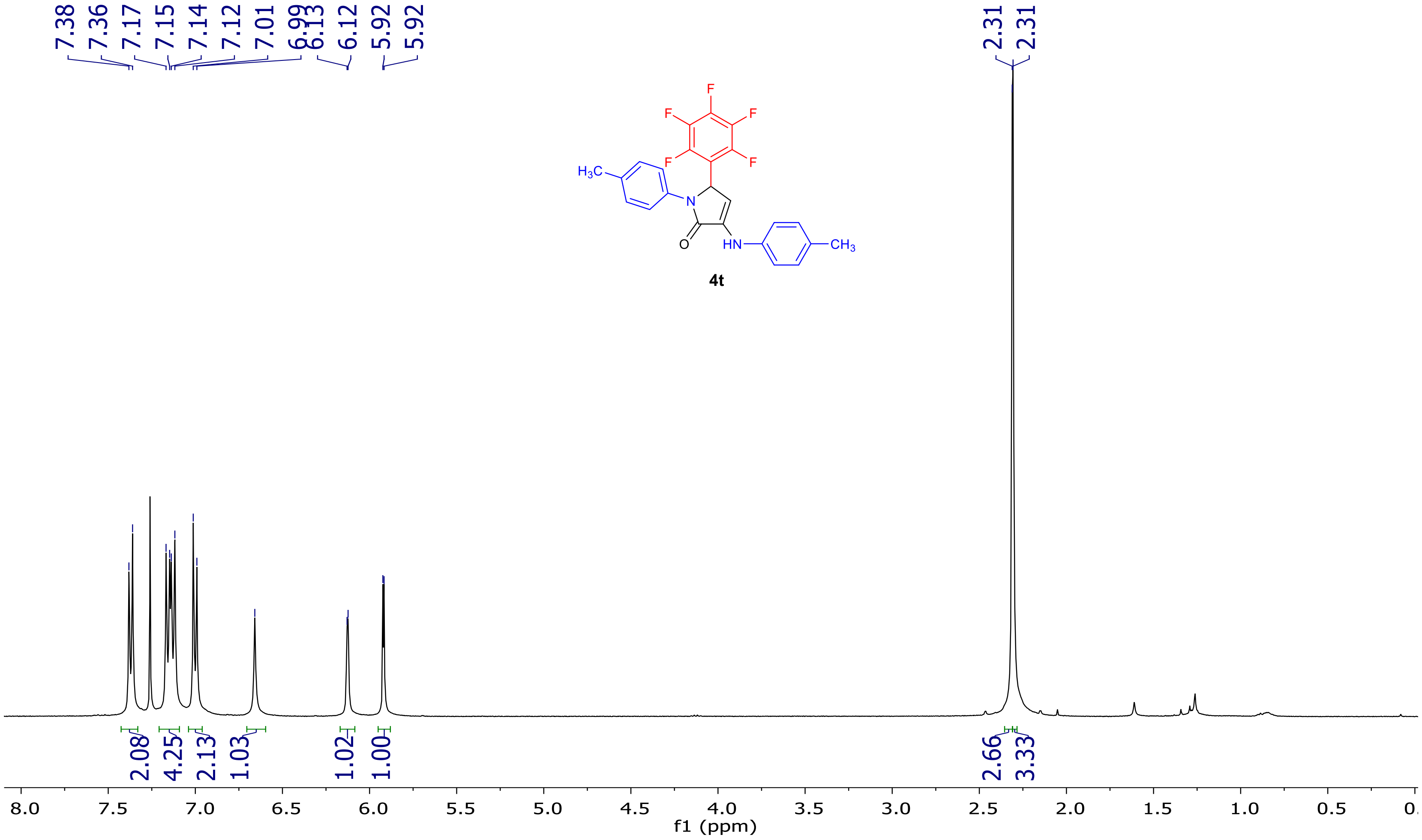

^{13}**C NMR** (75 MHz, $CDCl_3$)

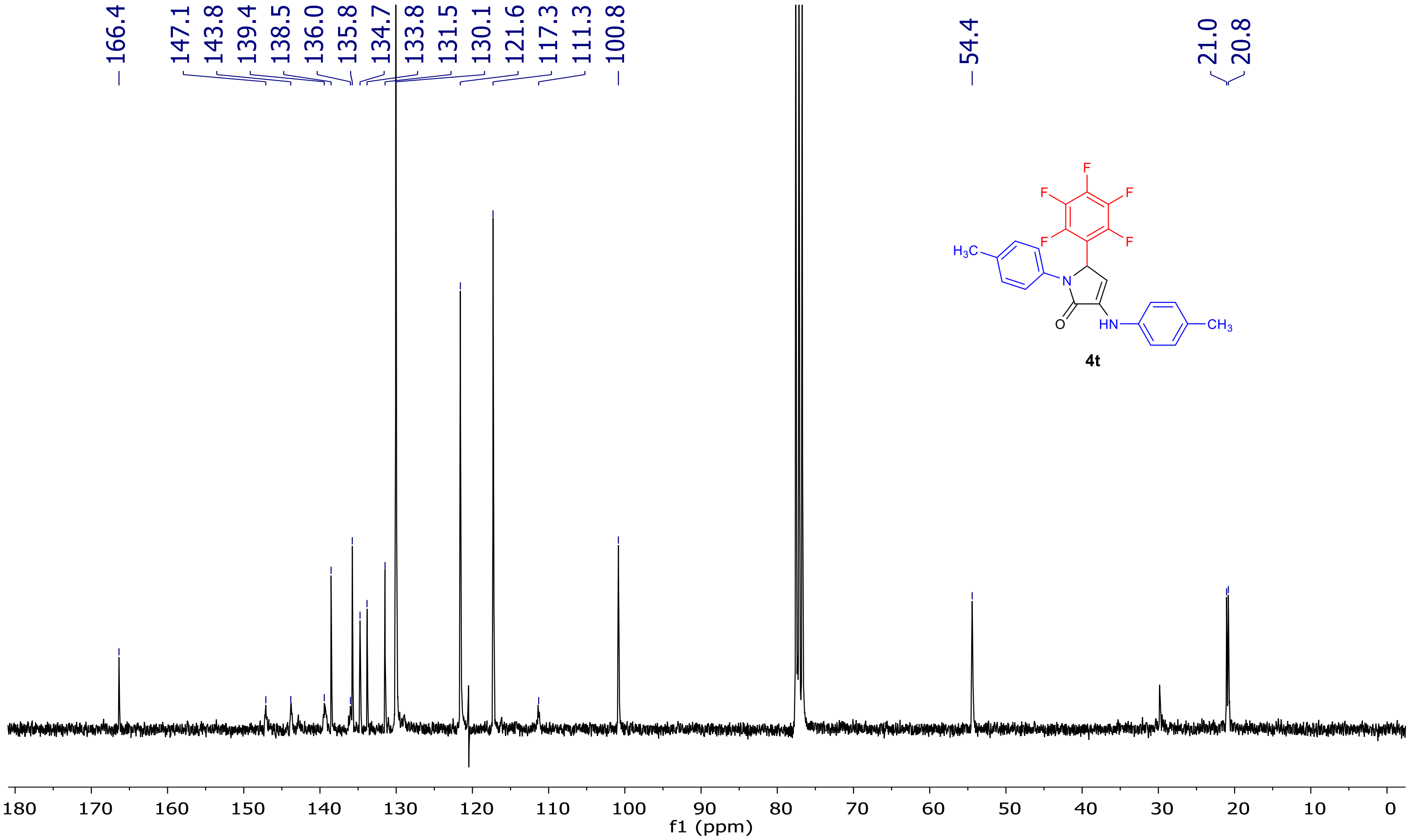

^{19}F NMR (282 MHz, $CDCl_3$)

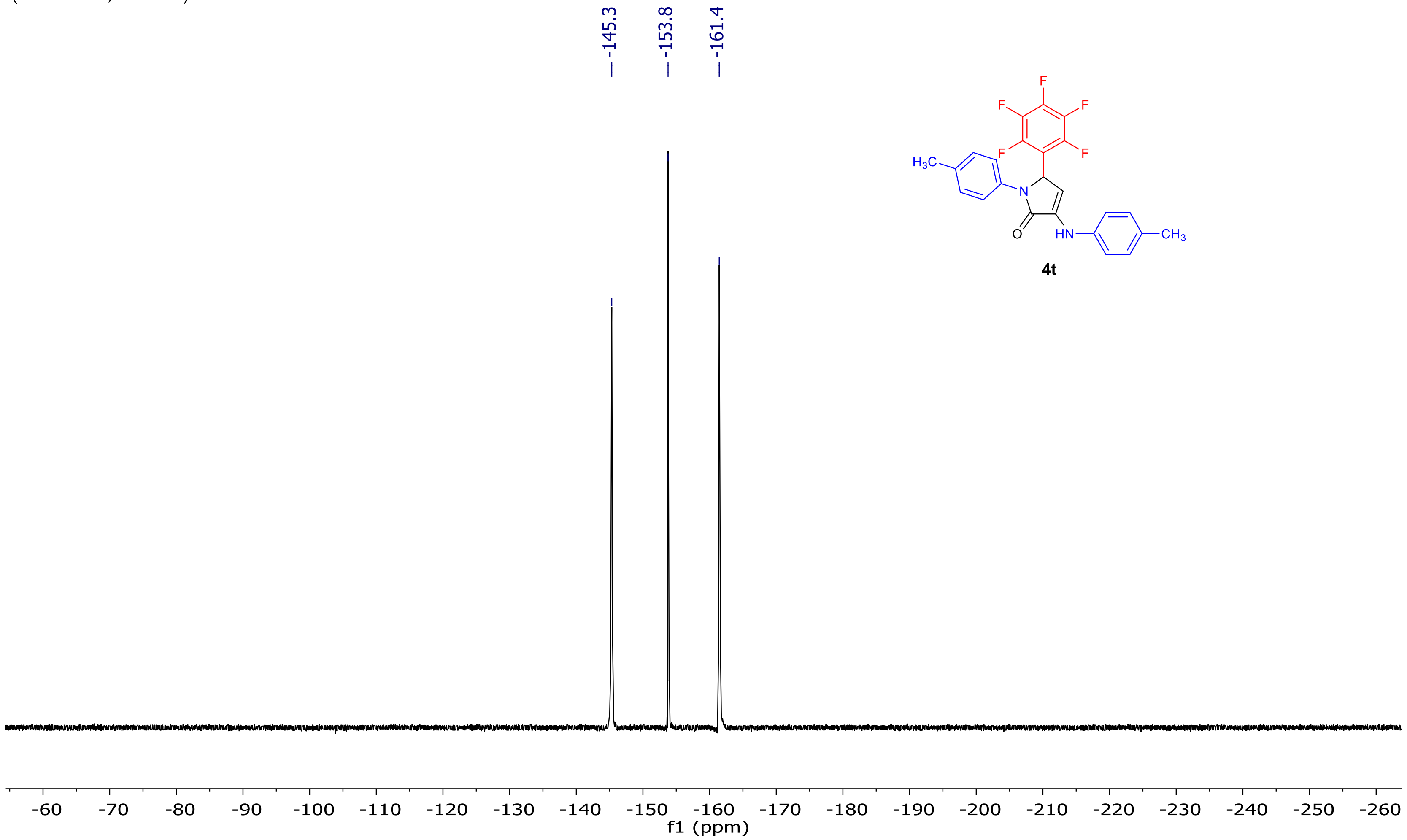

4t

*1-(p-Tolyl)-3-(p-tolylamino)-5-(trifluoromethyl)-1,5-dihydro-2H-pyrrol-2-one (**4u**).*

^{1}H NMR (400 MHz, $CDCl_3$)

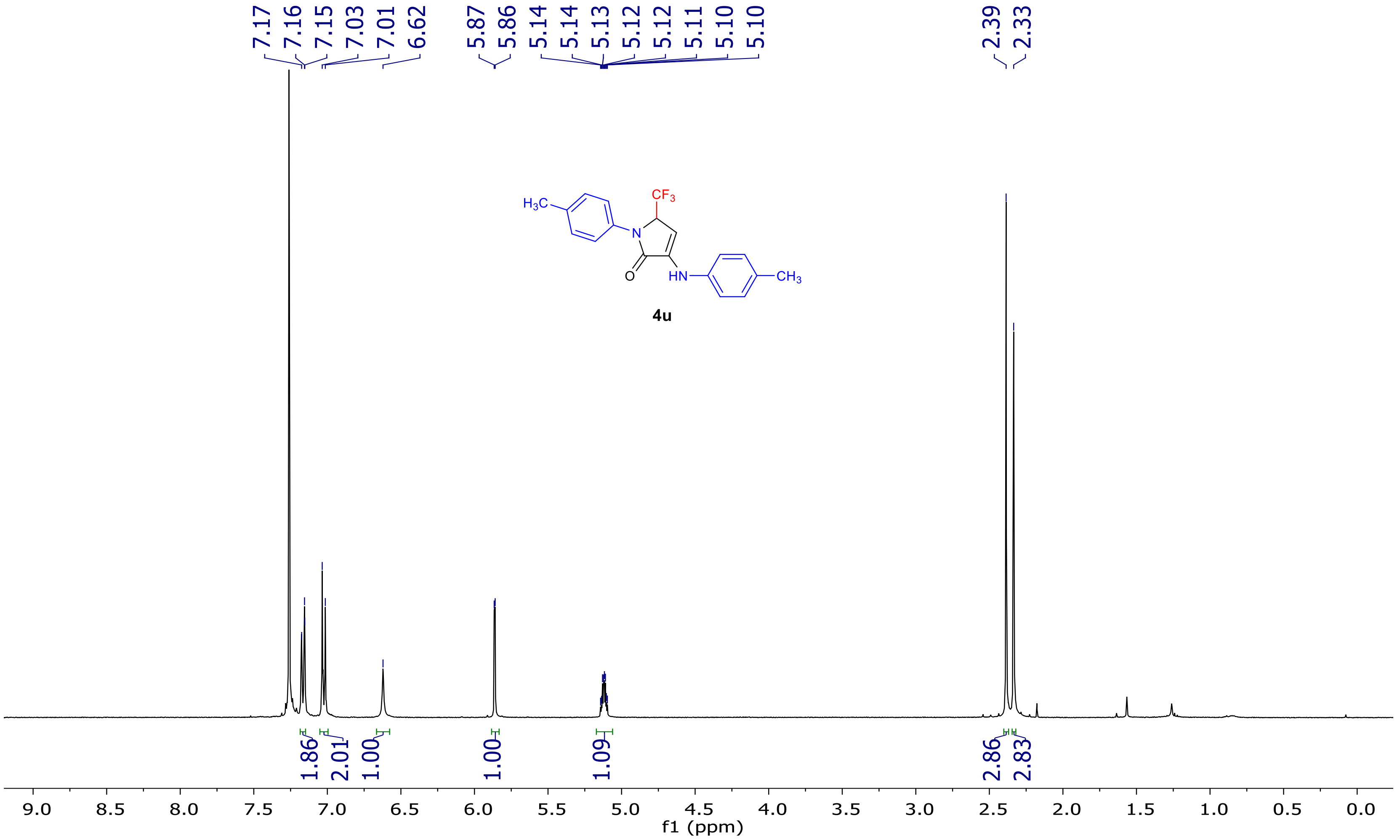

^{13}**C NMR** (101 MHz, $CDCl_3$)

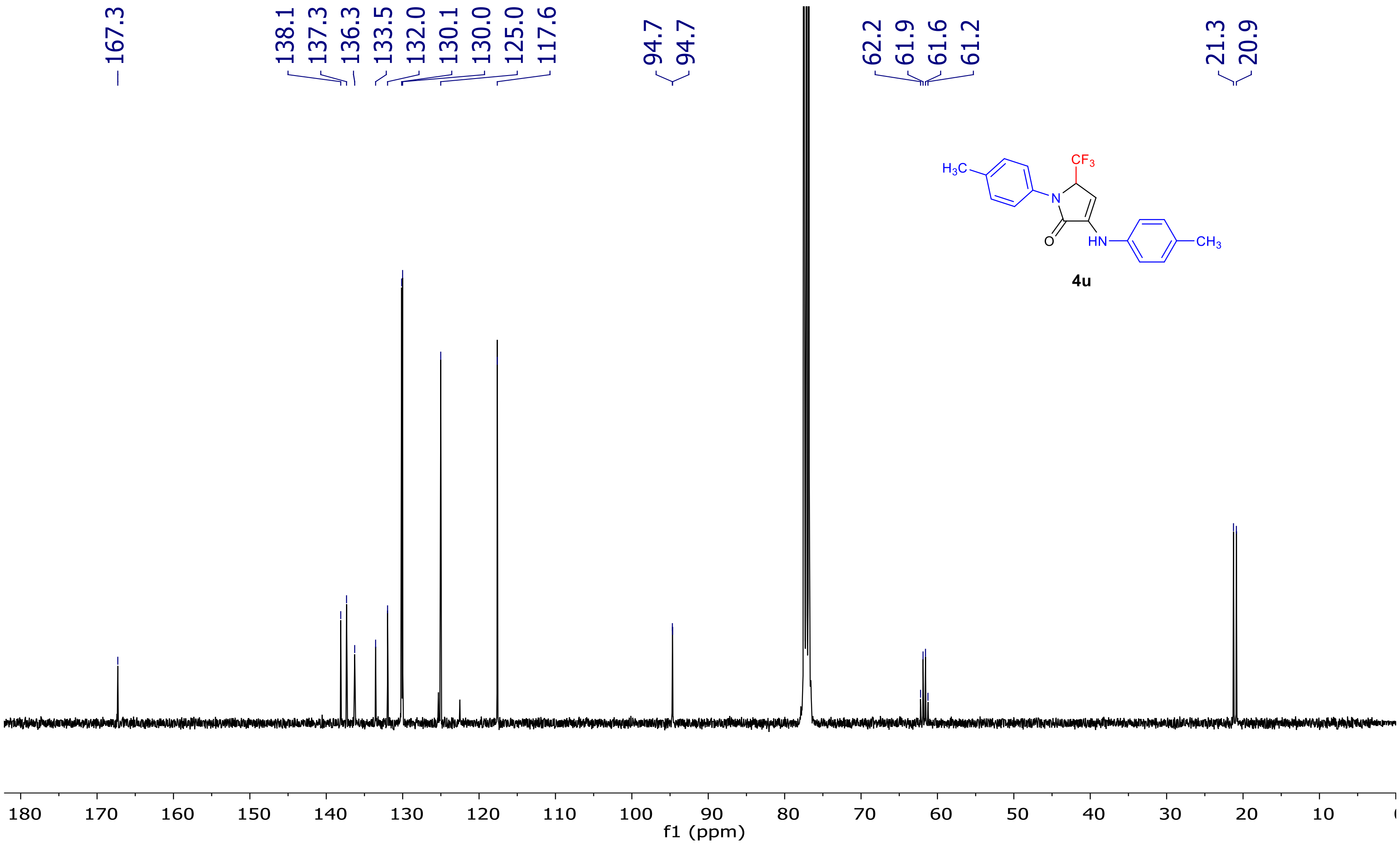

^{19}F NMR (282 MHz, CDCl3)

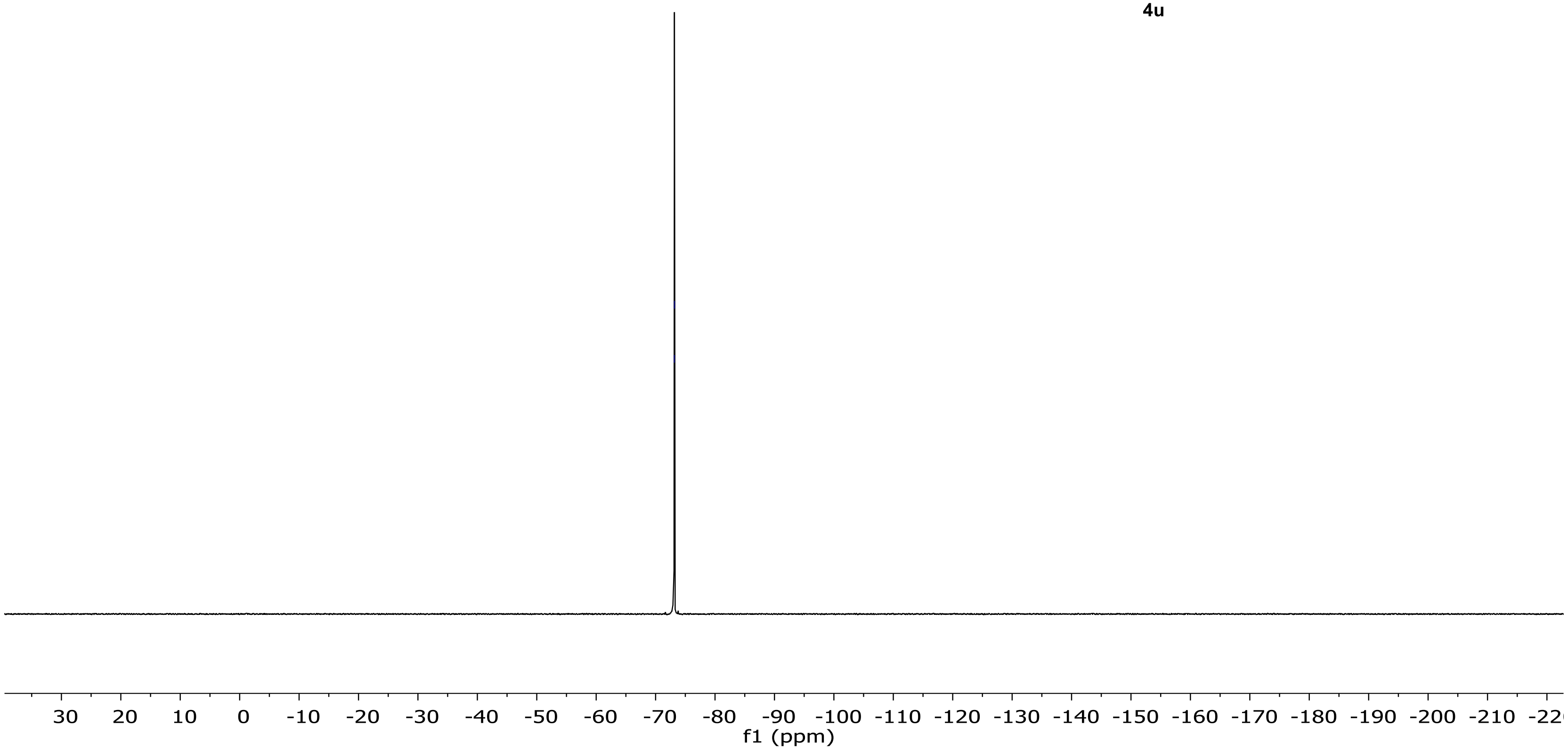

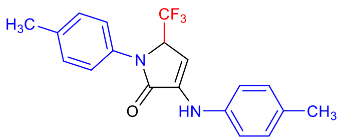

4u

*Diethyl ((5-oxo-1-(p-tolyl)-4-(p-tolylamino)-2,5-dihydro-1H-pyrrol-2-yl)methyl)-phosphonate (**4v**).*

^{1}H NMR (300 MHz, $CDCl_3$)

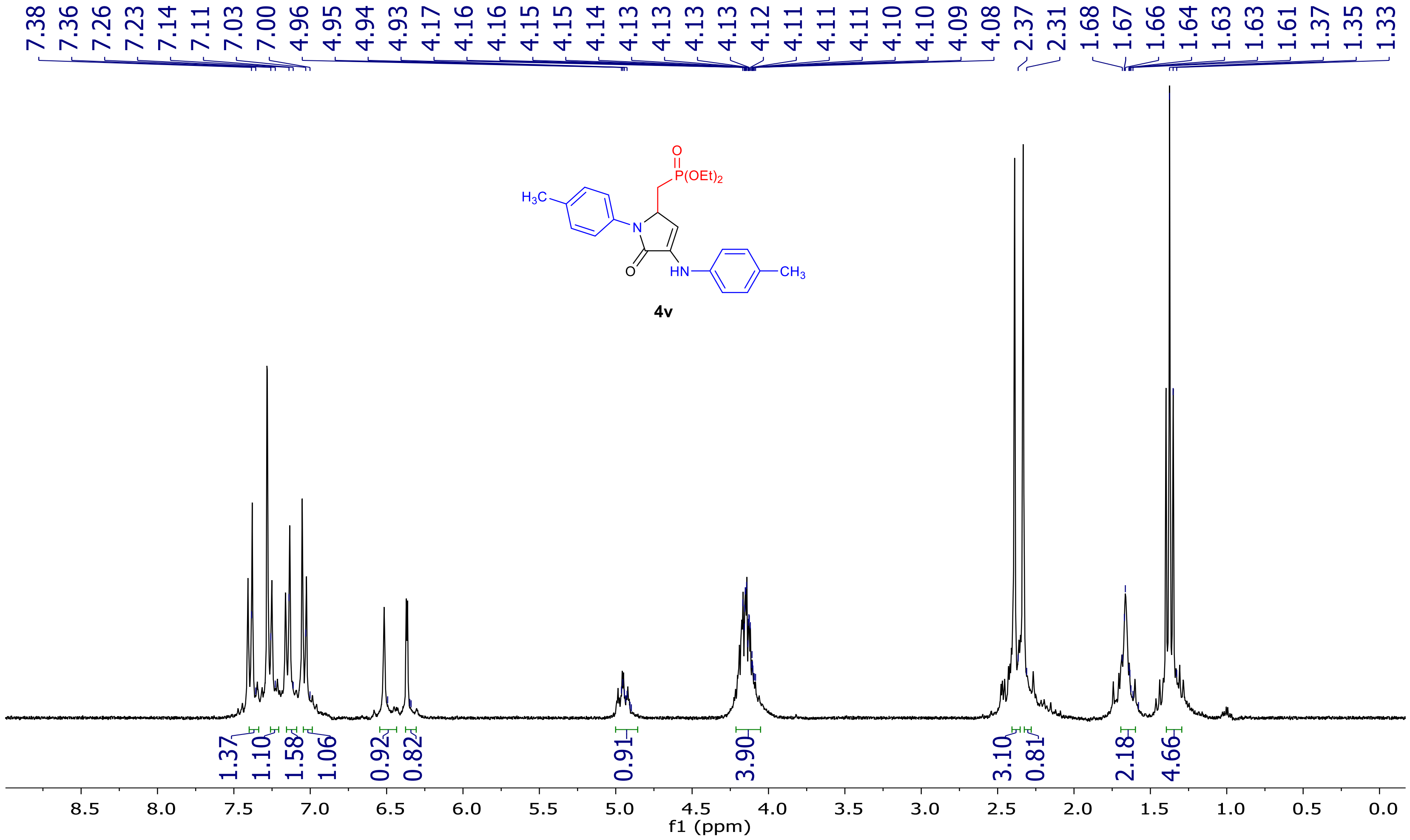

^{13}C NMR (75 MHz, $CDCl_3$)

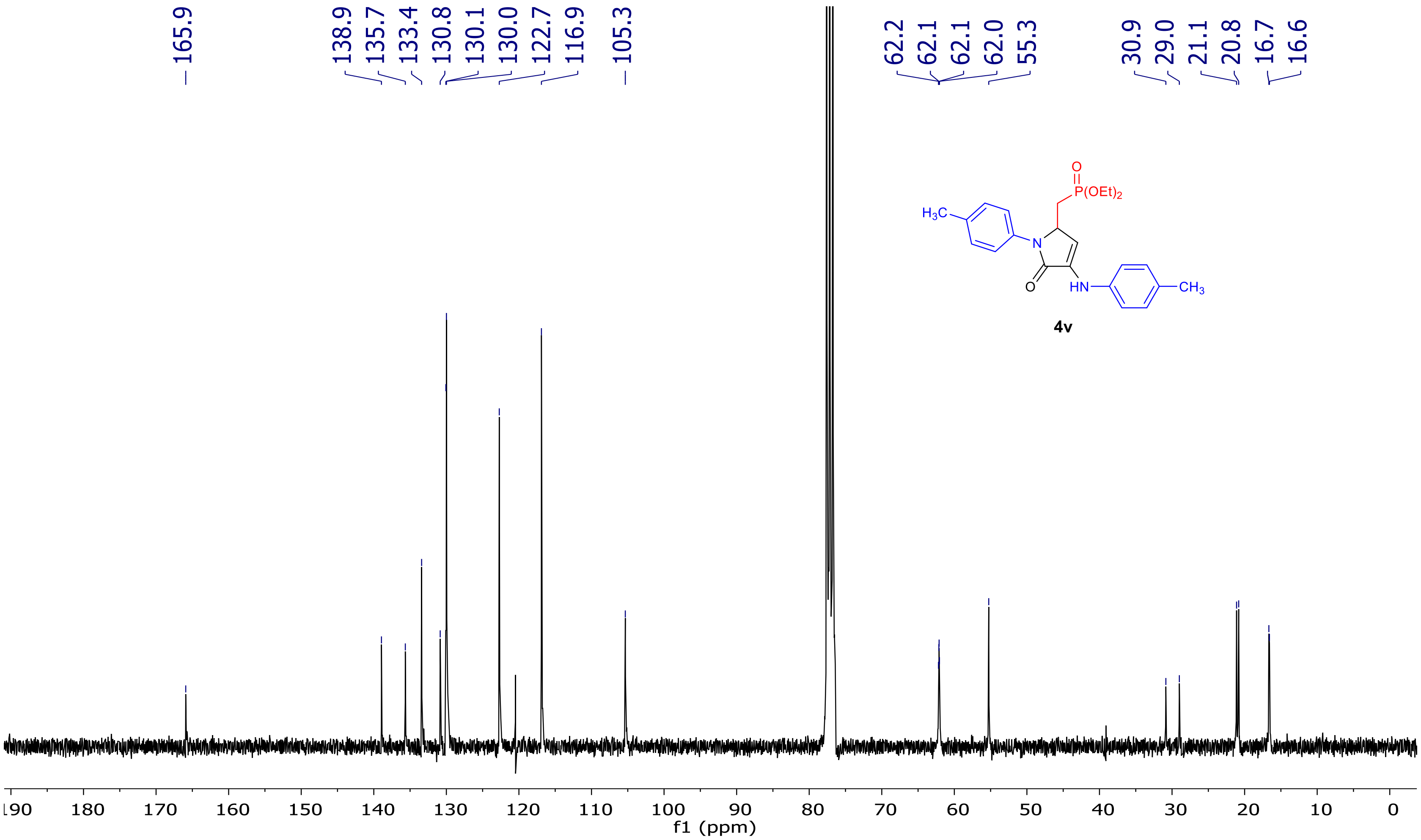

^{31}P NMR (121 MHz, $CDCl_3$)

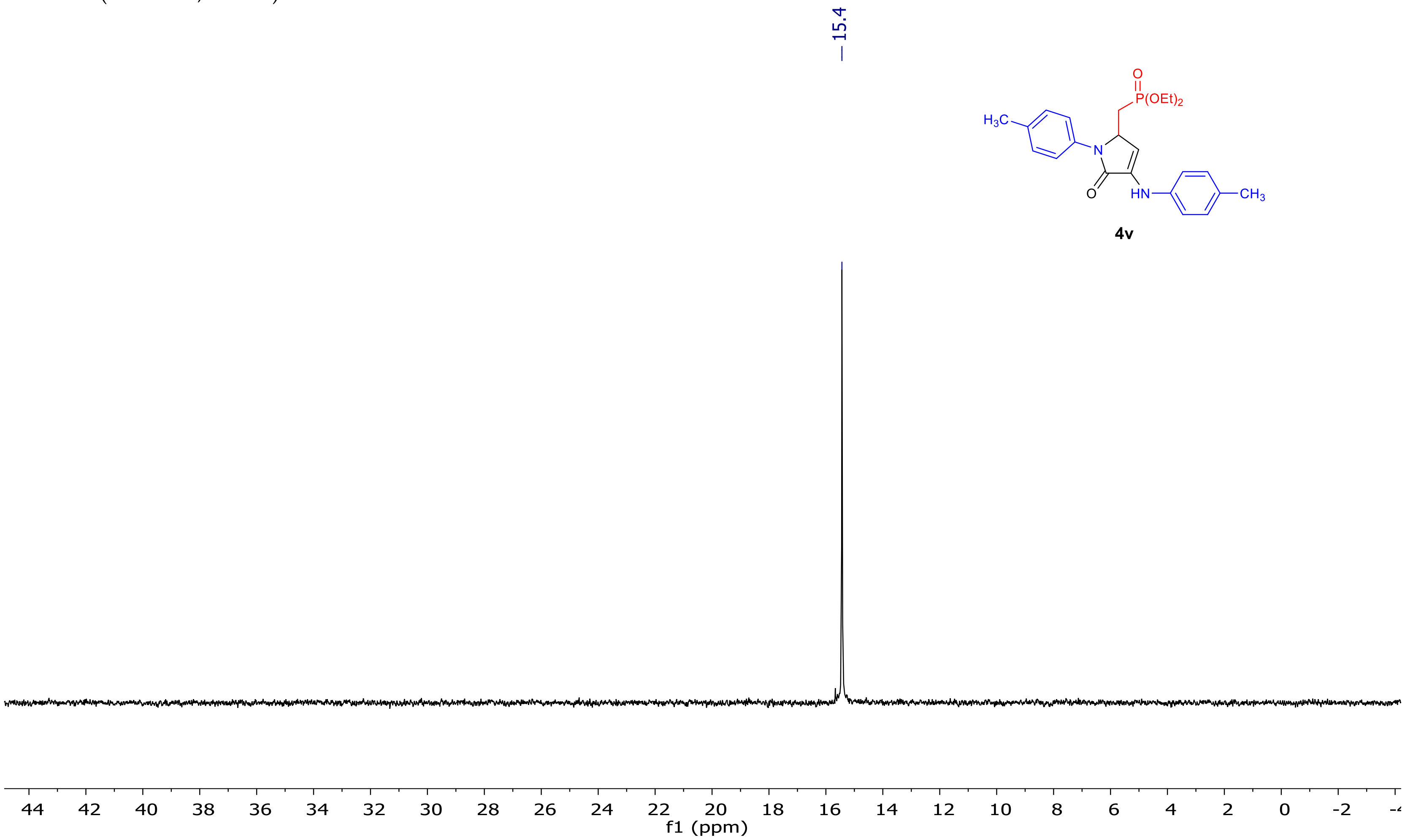

*5-((Diphenylphosphoryl)methyl)-1-(p-tolyl)-3-(p-tolylamino)-1H-pyrrol-2(5H)-one (**4w**).*

^{1}H NMR (400 MHz, $CDCl_3$)

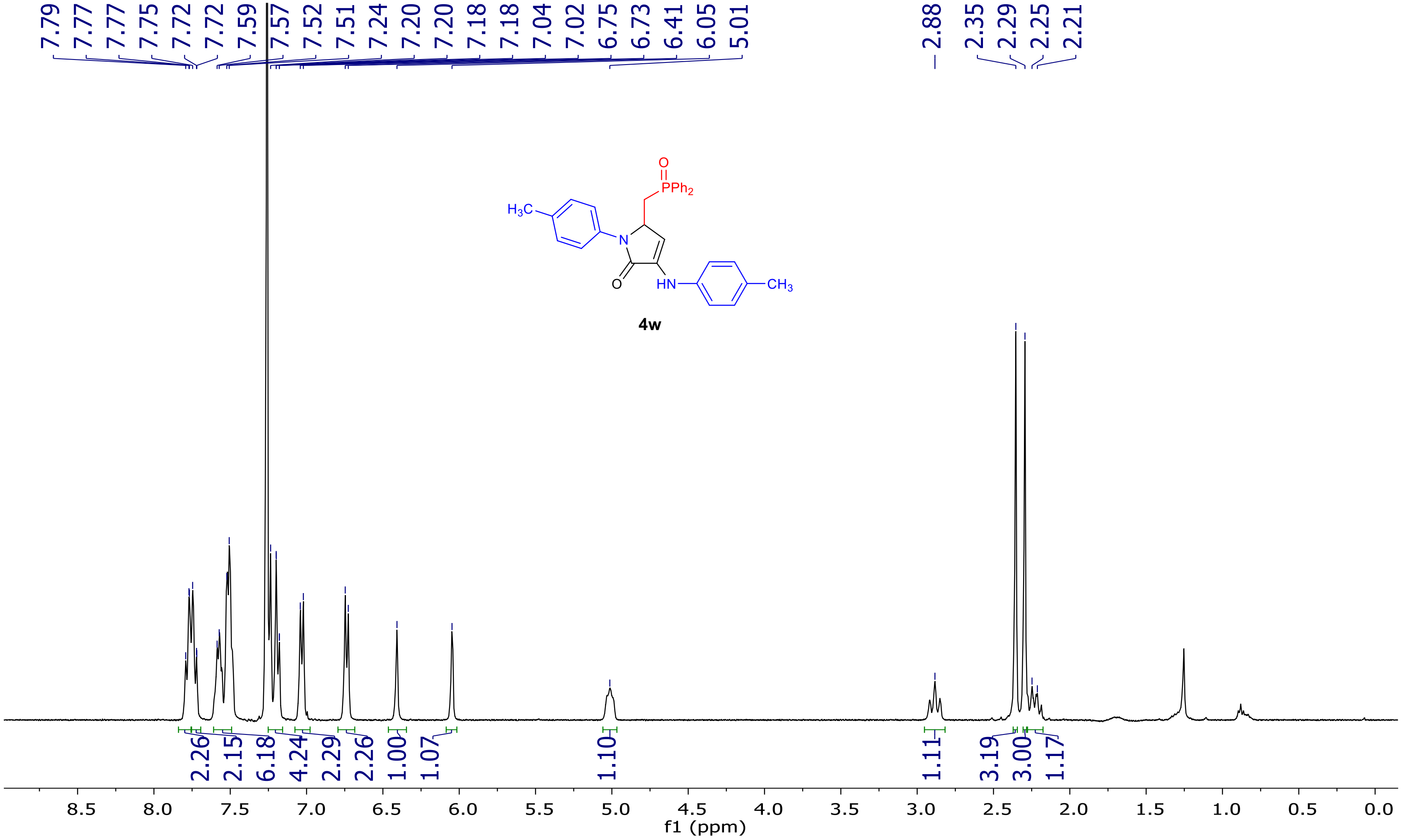

13C NMR (101 MHz, CDCl3)

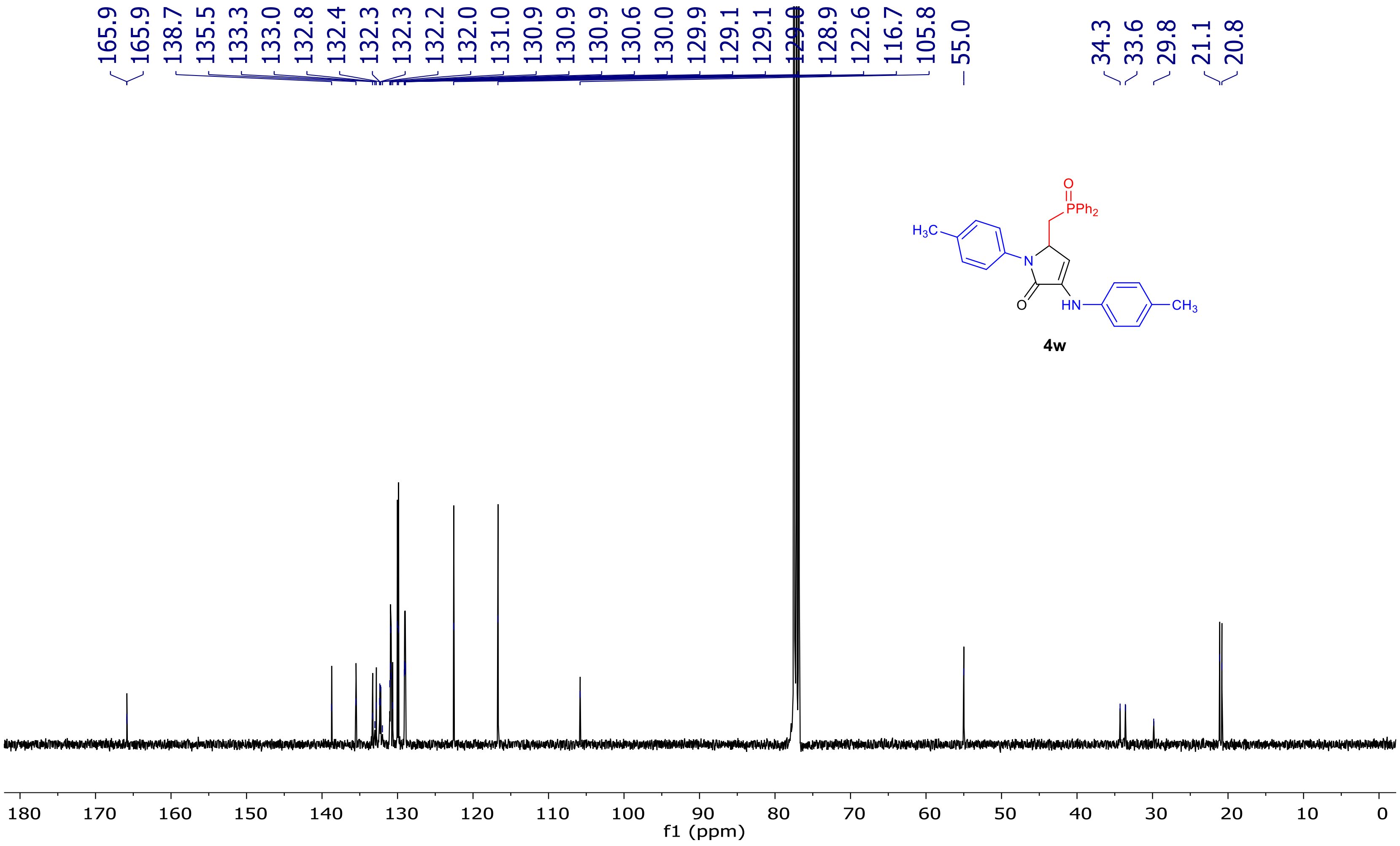

^{31}P NMR (121 MHz, $CDCl_3$)

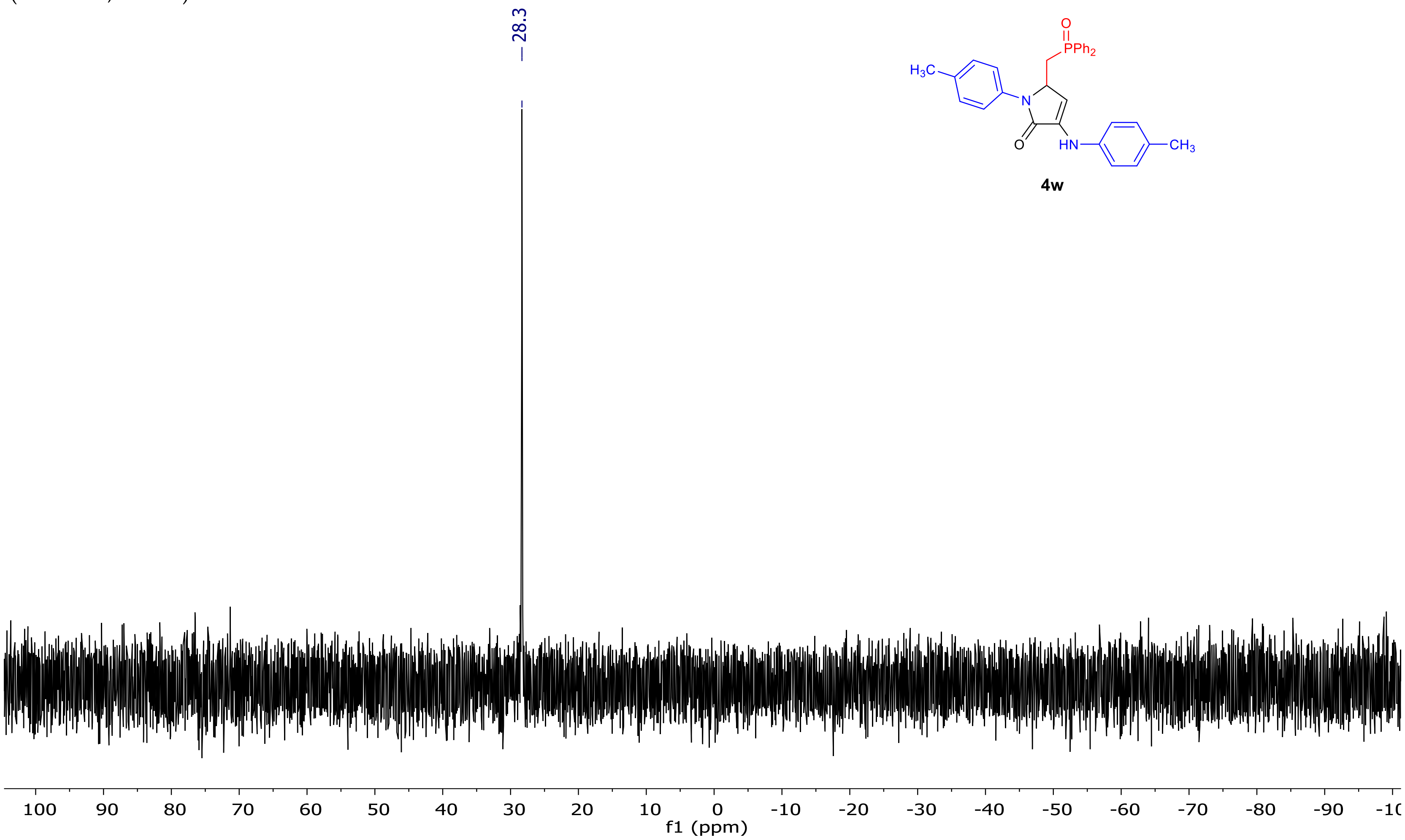

*4-Methyl-5-(p-nitrophenyl)-1-(p-tolyl)-3-(p-tolylamino)-1,5-dihydro-2H-pyrrol-2-one (**5a**).*

^{1}H NMR (400 MHz, $CDCl_3$)

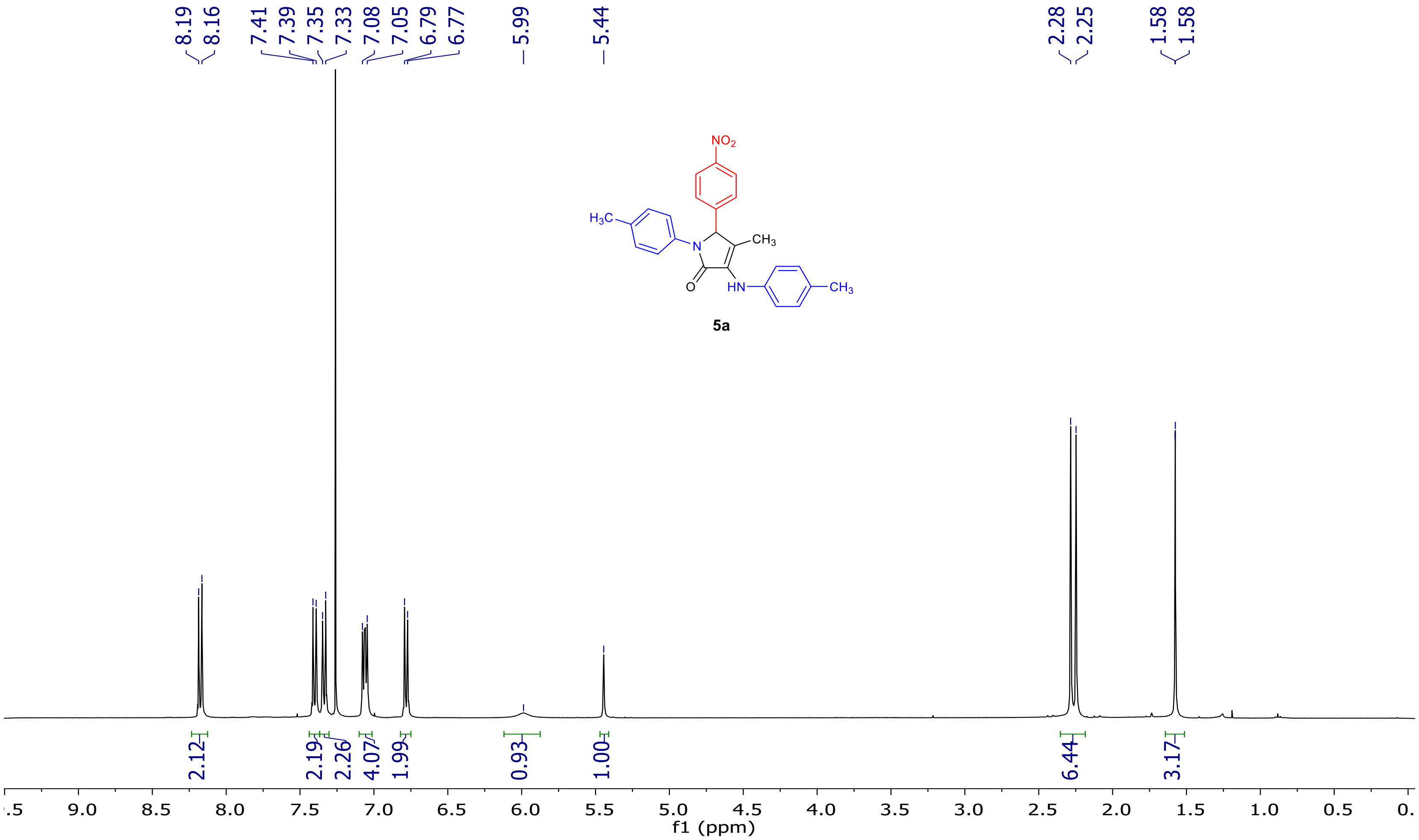

13C NMR (75 MHz, CDCl3)

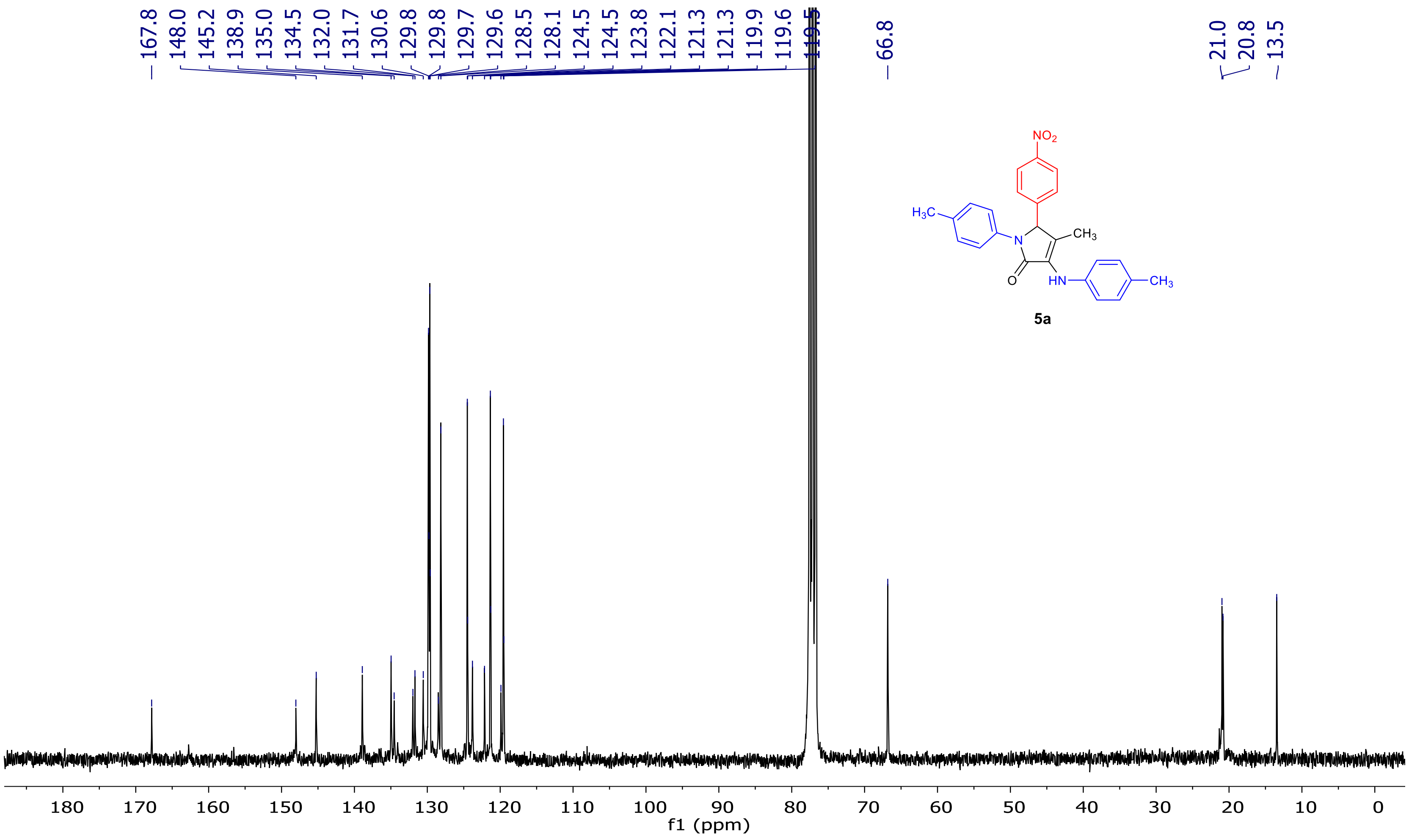

*4-Benzyl-5-(p-nitrophenyl)-1-(p-tolyl)-3-(p-tolylamino)-1,5-dihydro-2H-pyrrol-2-one (**5b**).*

^{1}**H NMR** (300 MHz, $CDCl_3$)

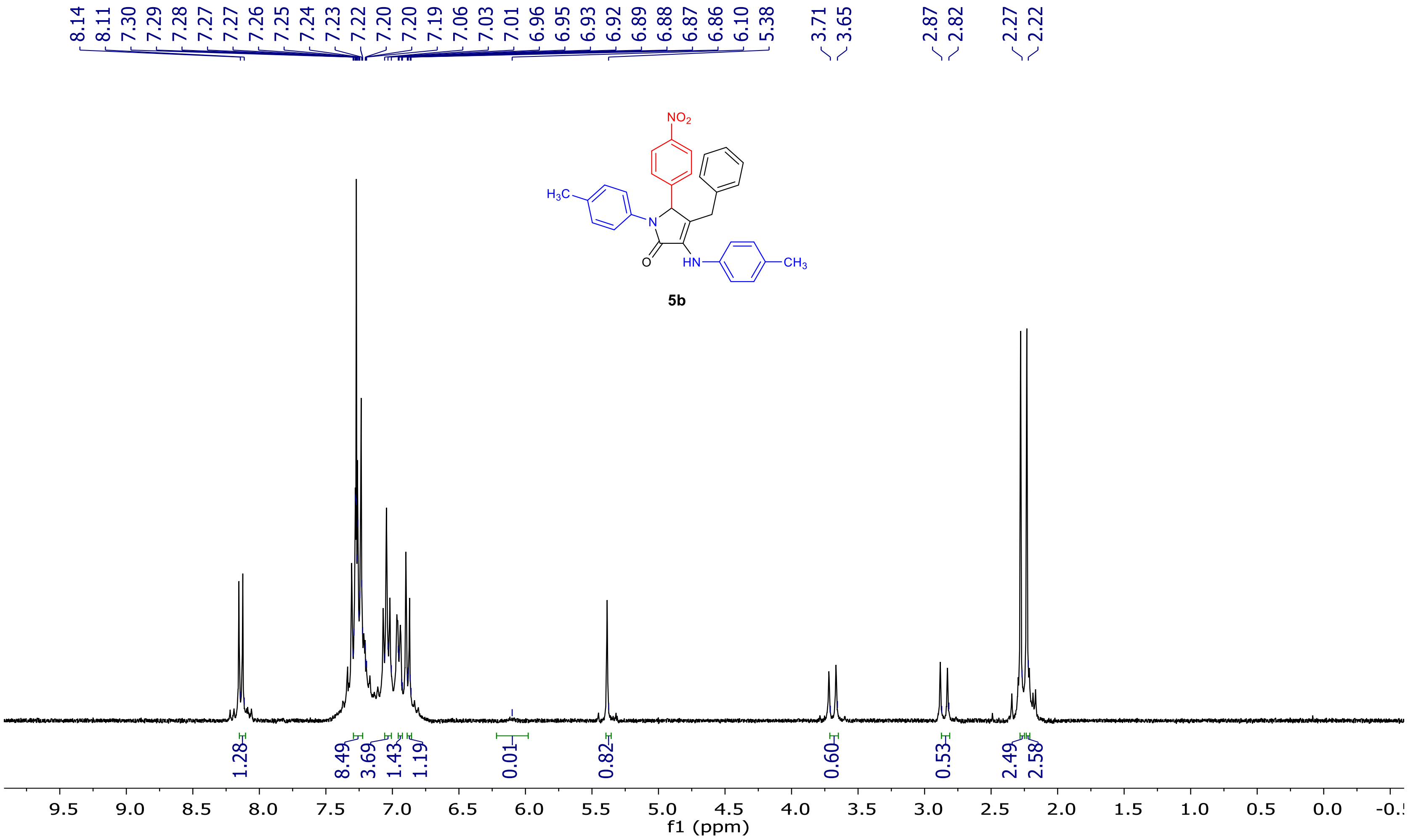

^{13}**C NMR** (75 MHz, $CDCl_3$)

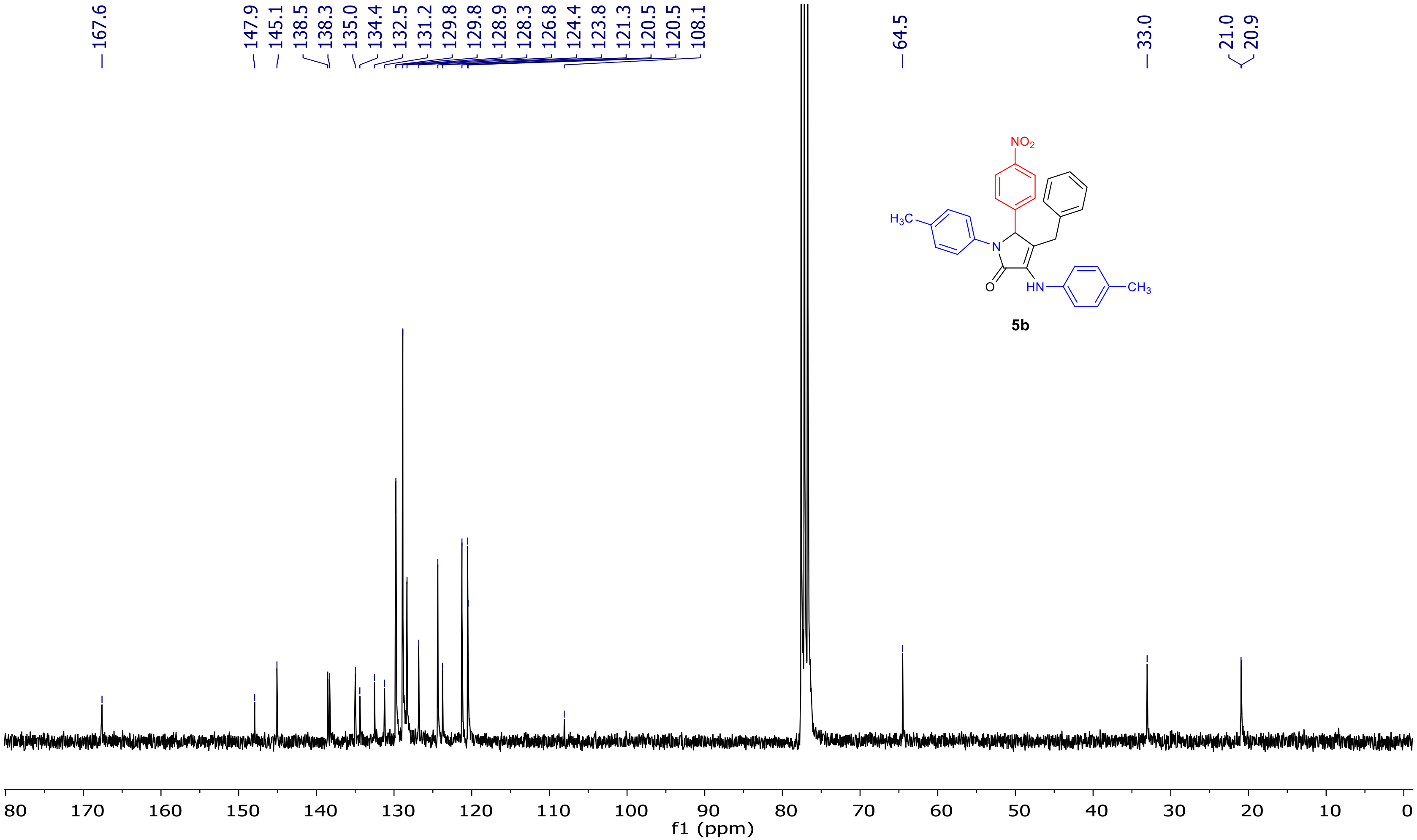

*Methyl 5-oxo-2-phenyl-1-(p-tolyl)-4-(p-tolylamino)-2,5-dihydro-1H-pyrrole-3-carboxylate (**5c**).*
^{1}H NMR (400 MHz, $CDCl_3$)

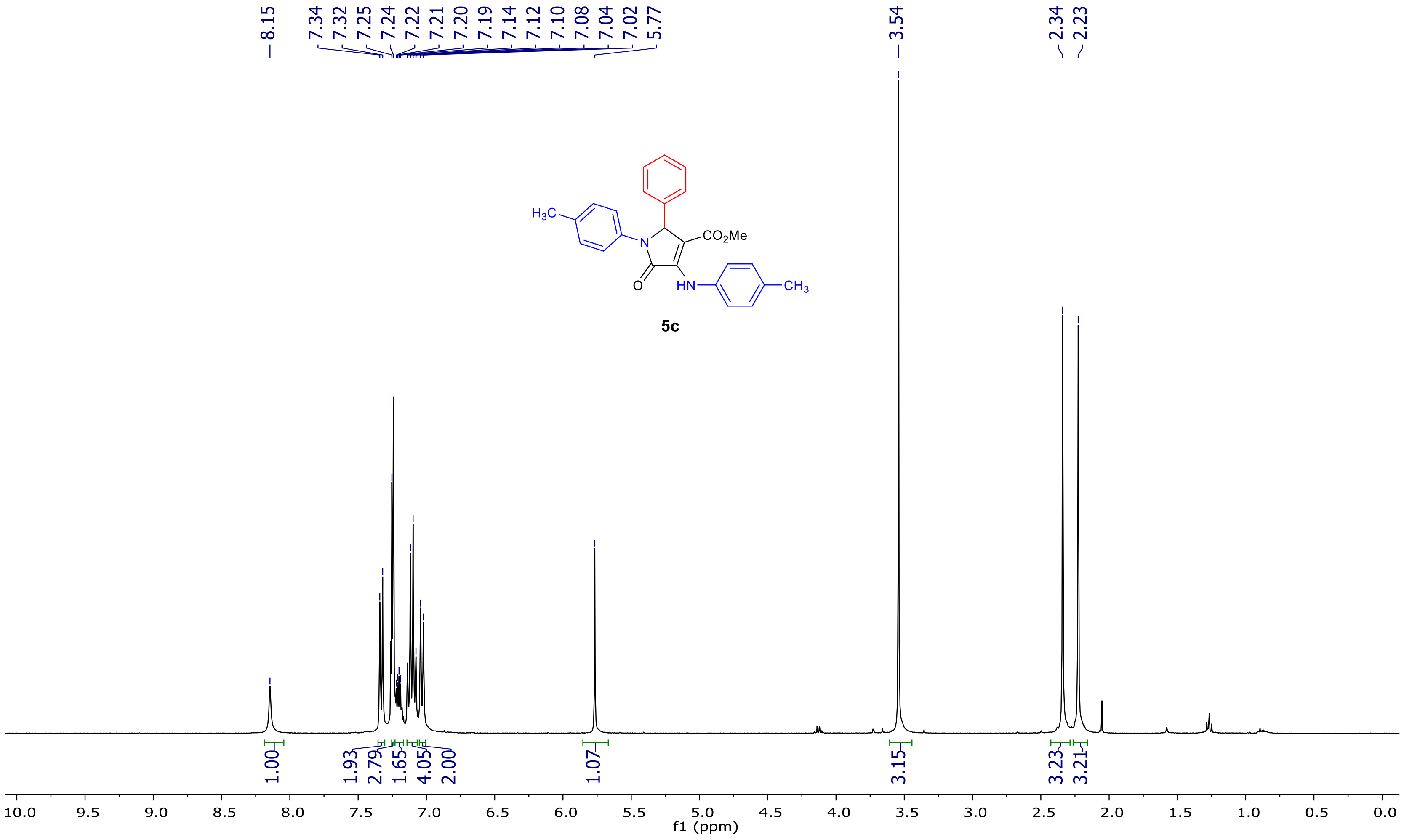

13C NMR (101 MHz, $CDCl_3$)

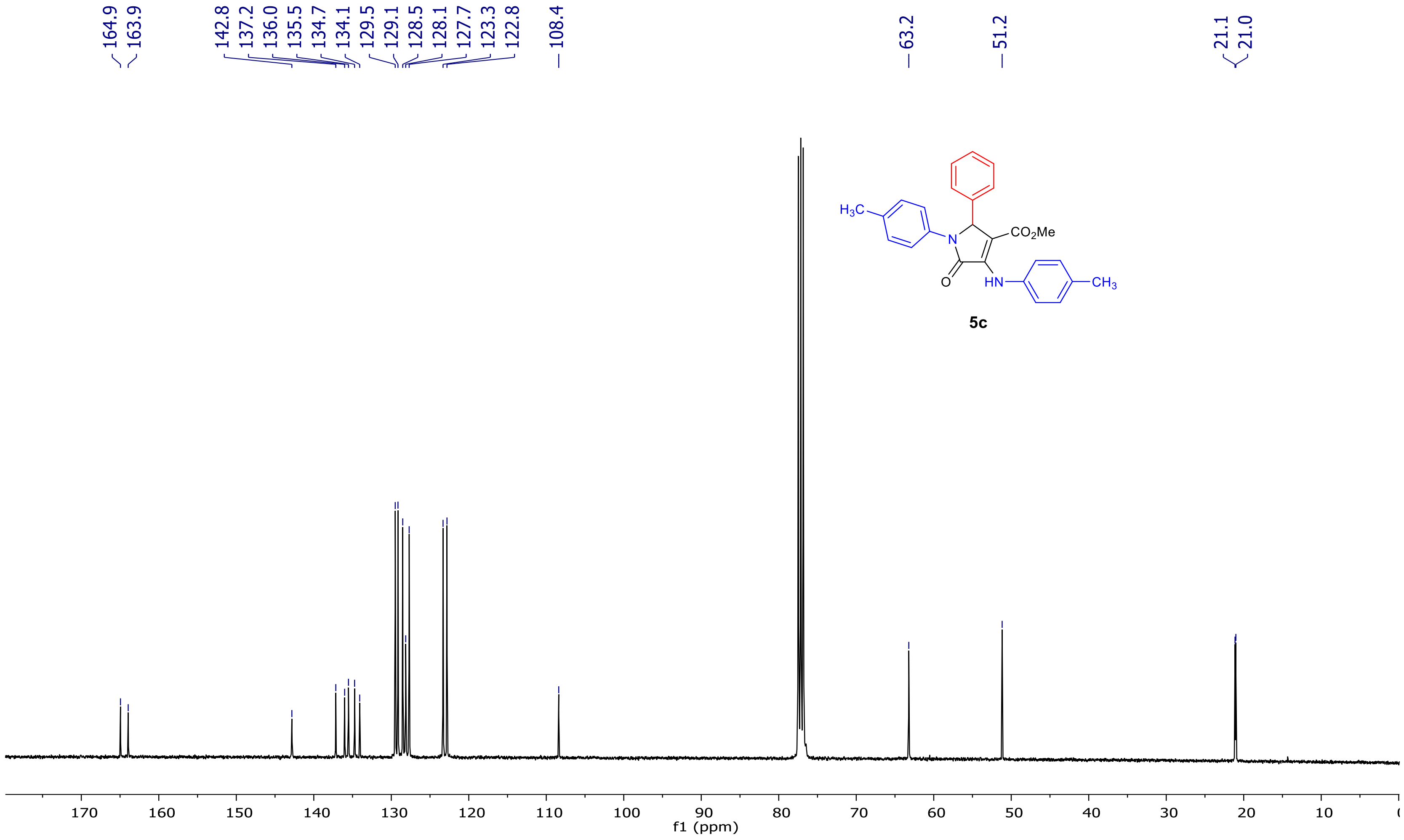

*Diethyl (5-oxo-1-(p-tolyl)-4-(p-tolylamino)-2,5-dihydro-1H-pyrrol-3-yl)phosphonate (**5d**).*
[1]**H NMR** (300 MHz, $CDCl_3$)

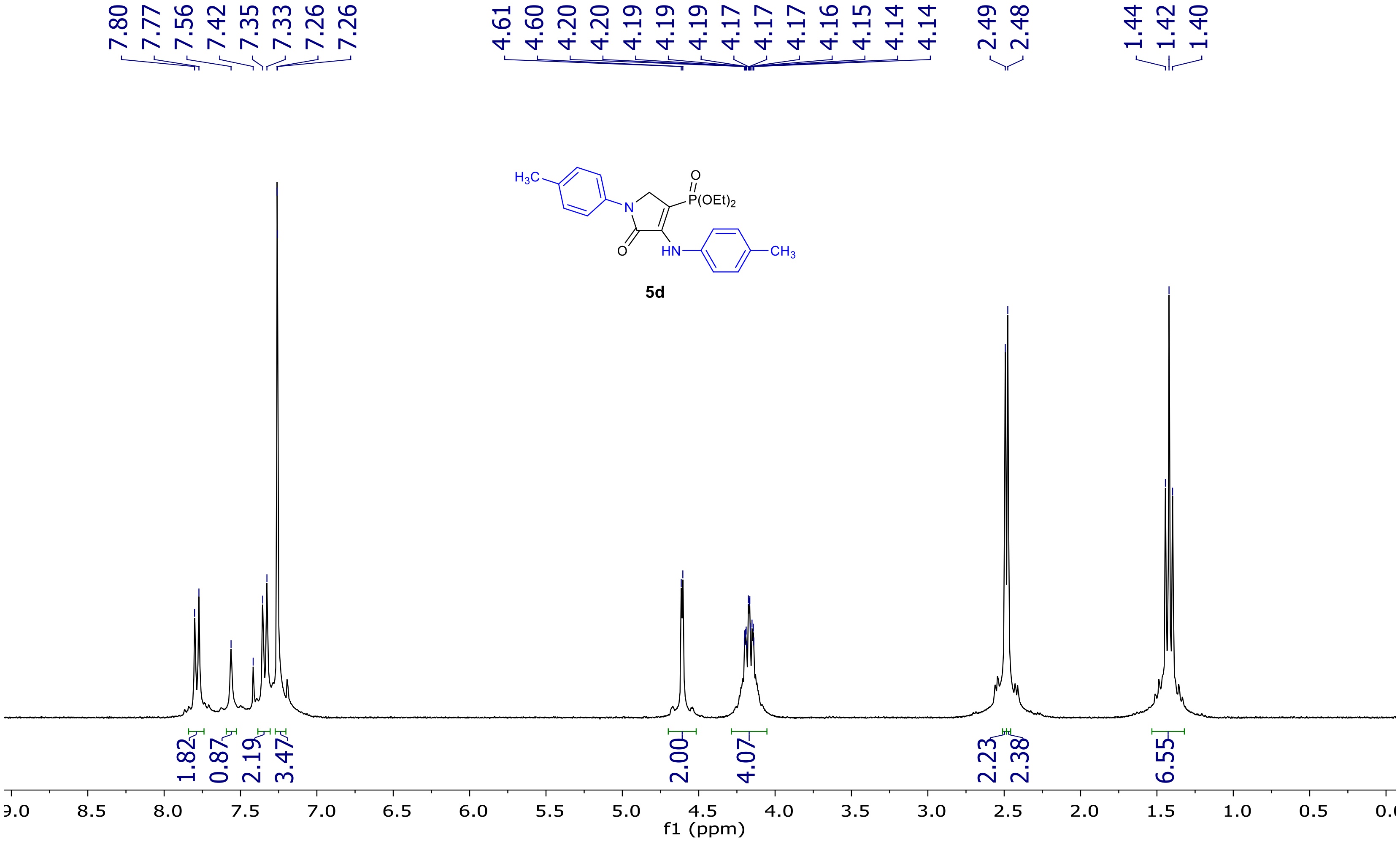

¹³C NMR (75 MHz, $CDCl_3$)

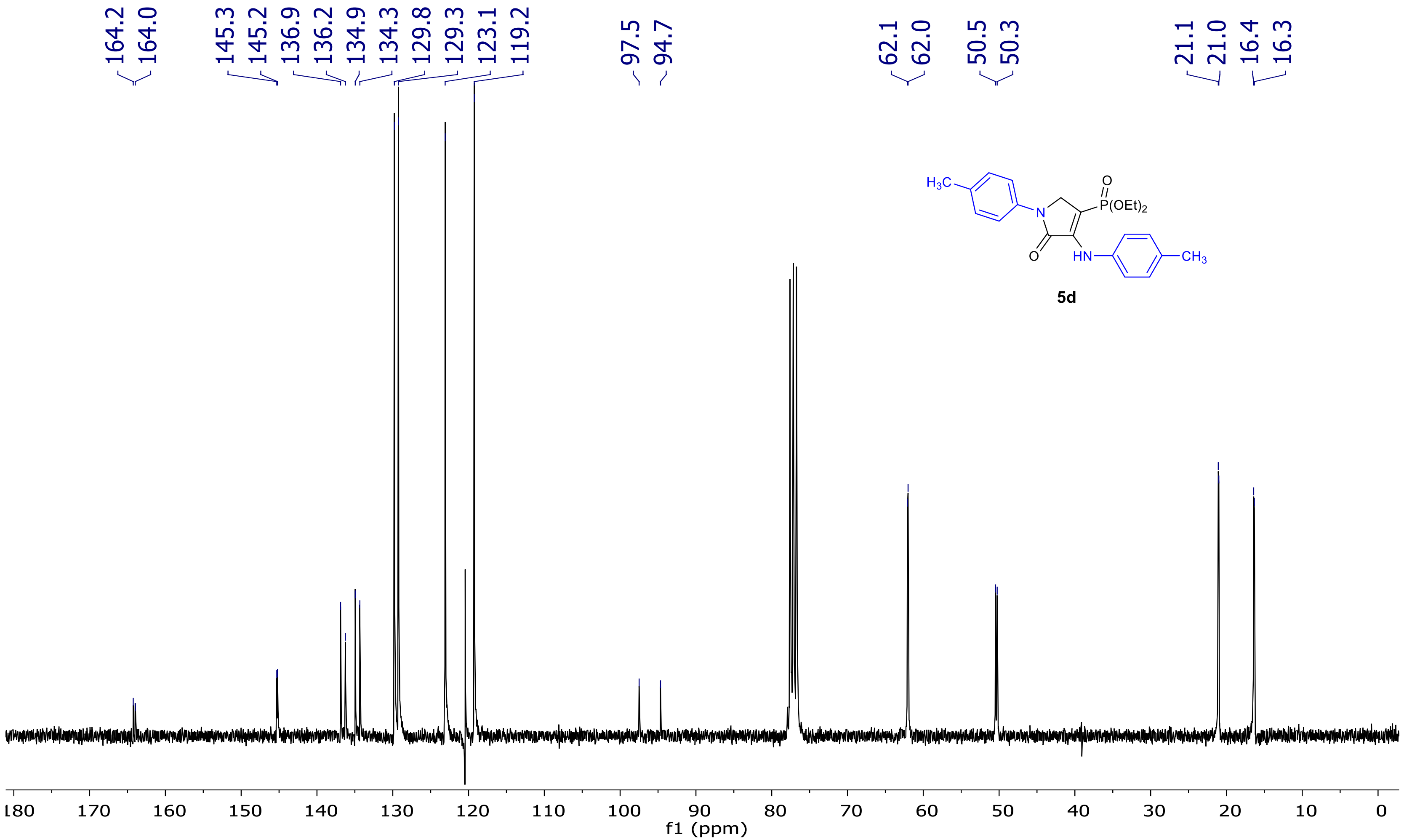

^{31}P NMR (121 MHz, $CDCl_3$)

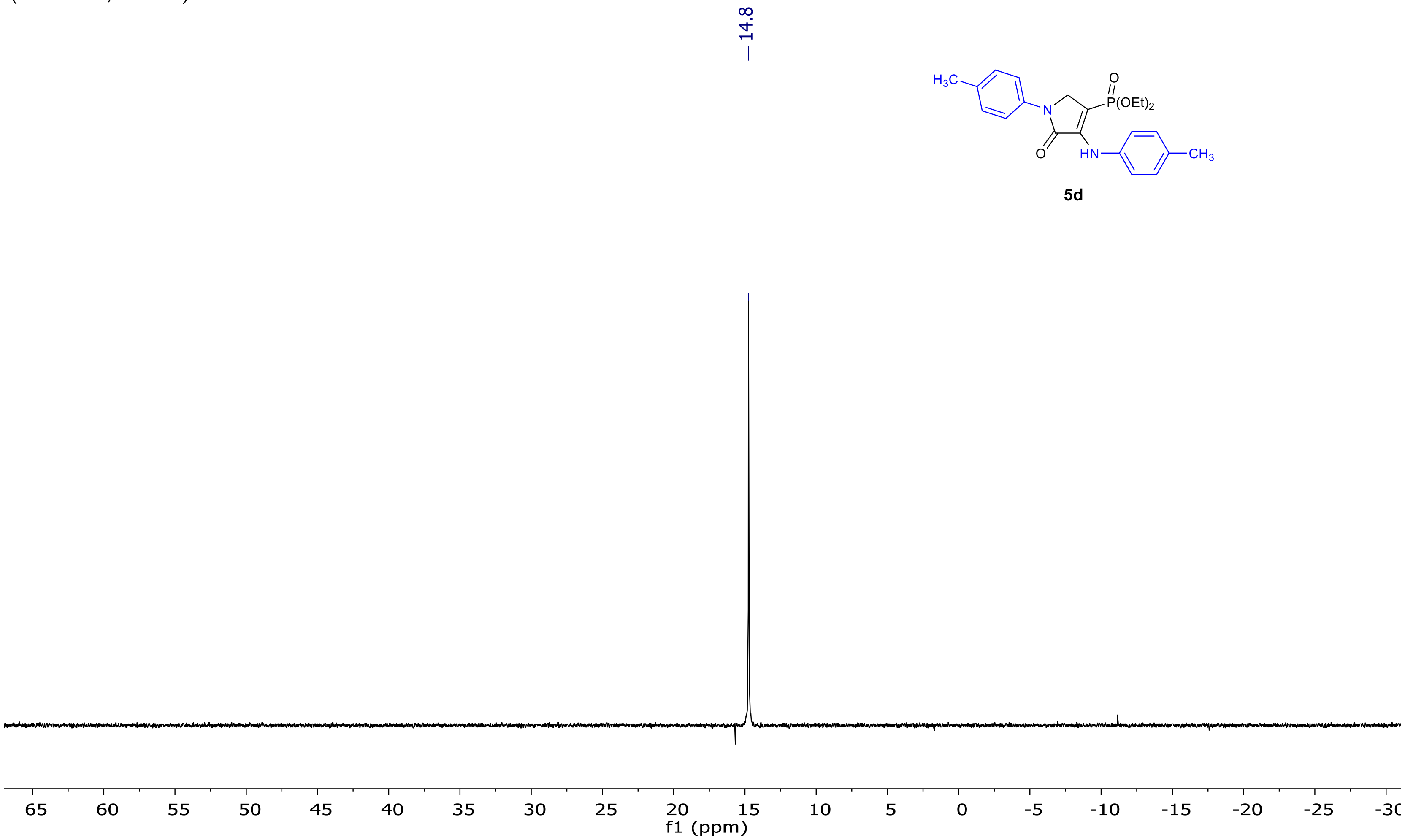

*4-(Diphenylphosphoryl)-1-(p-tolyl)-3-(p-tolylamino)-1,5-dihydro-2H-pyrrol-2-one. (**5e**)*

^{1}H NMR (300 MHz, $CDCl_3$)

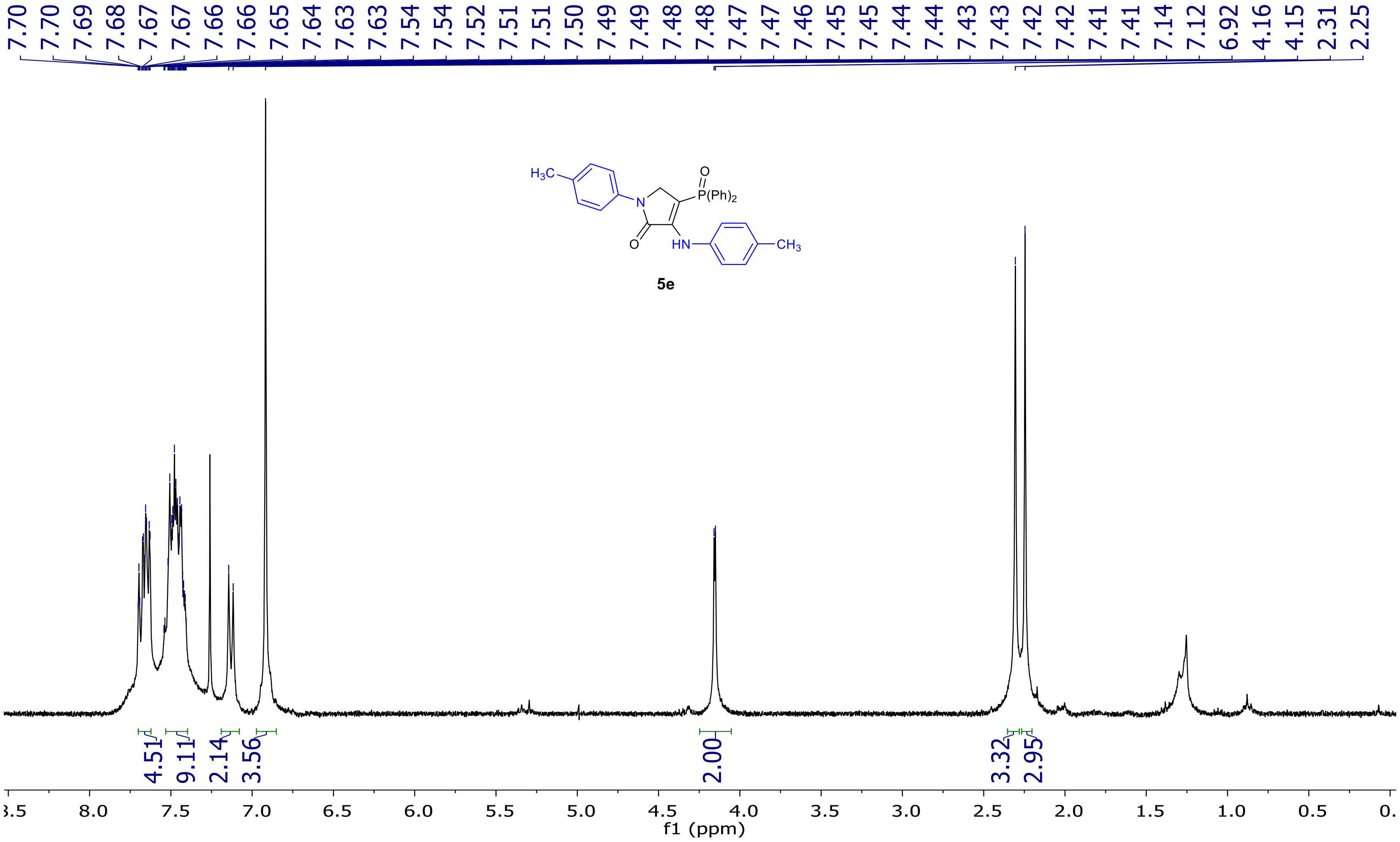

^{13}C NMR (75 MHz, $CDCl_3$)

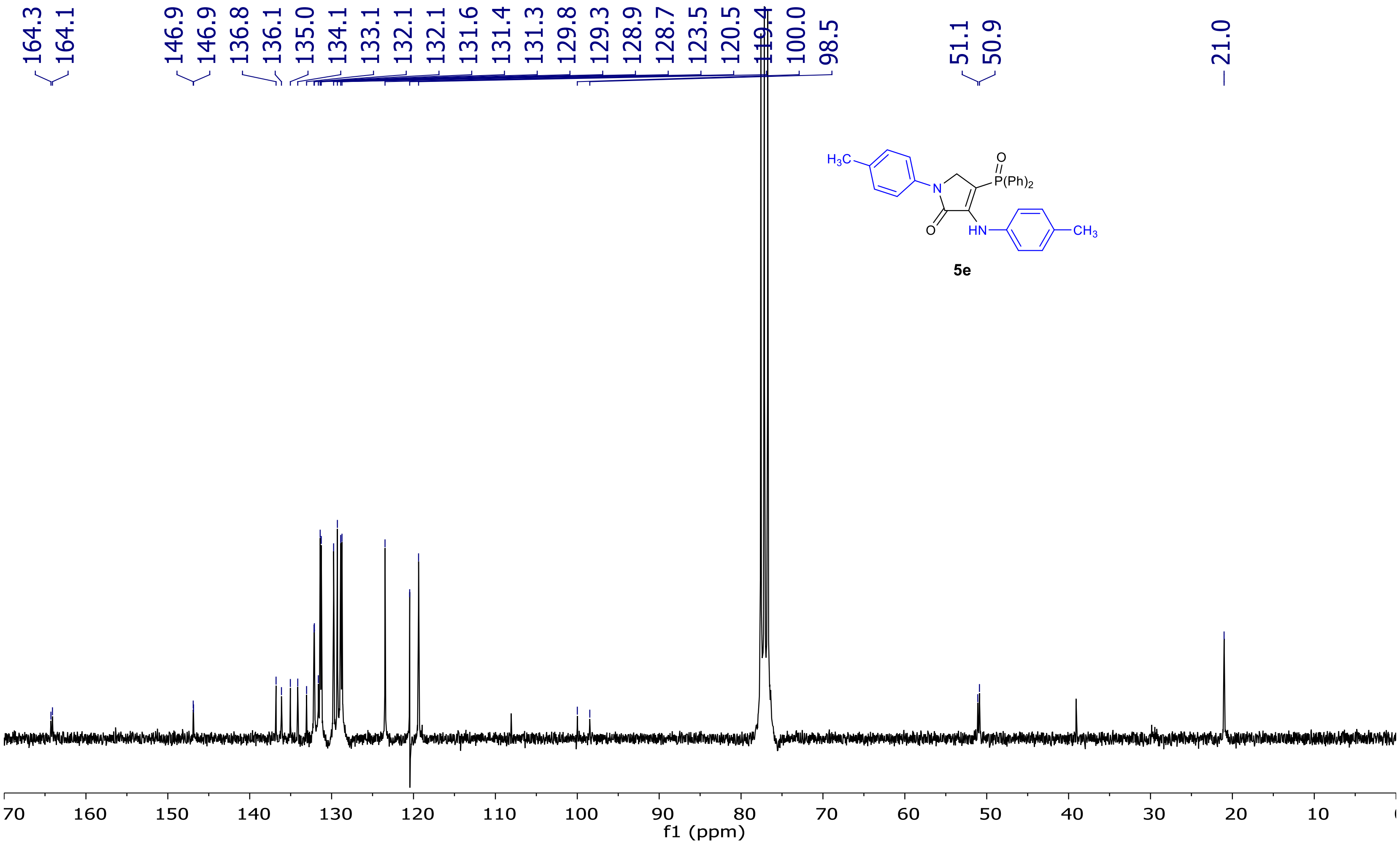

5e

^{31}P NMR (121 MHz, $CDCl_3$)

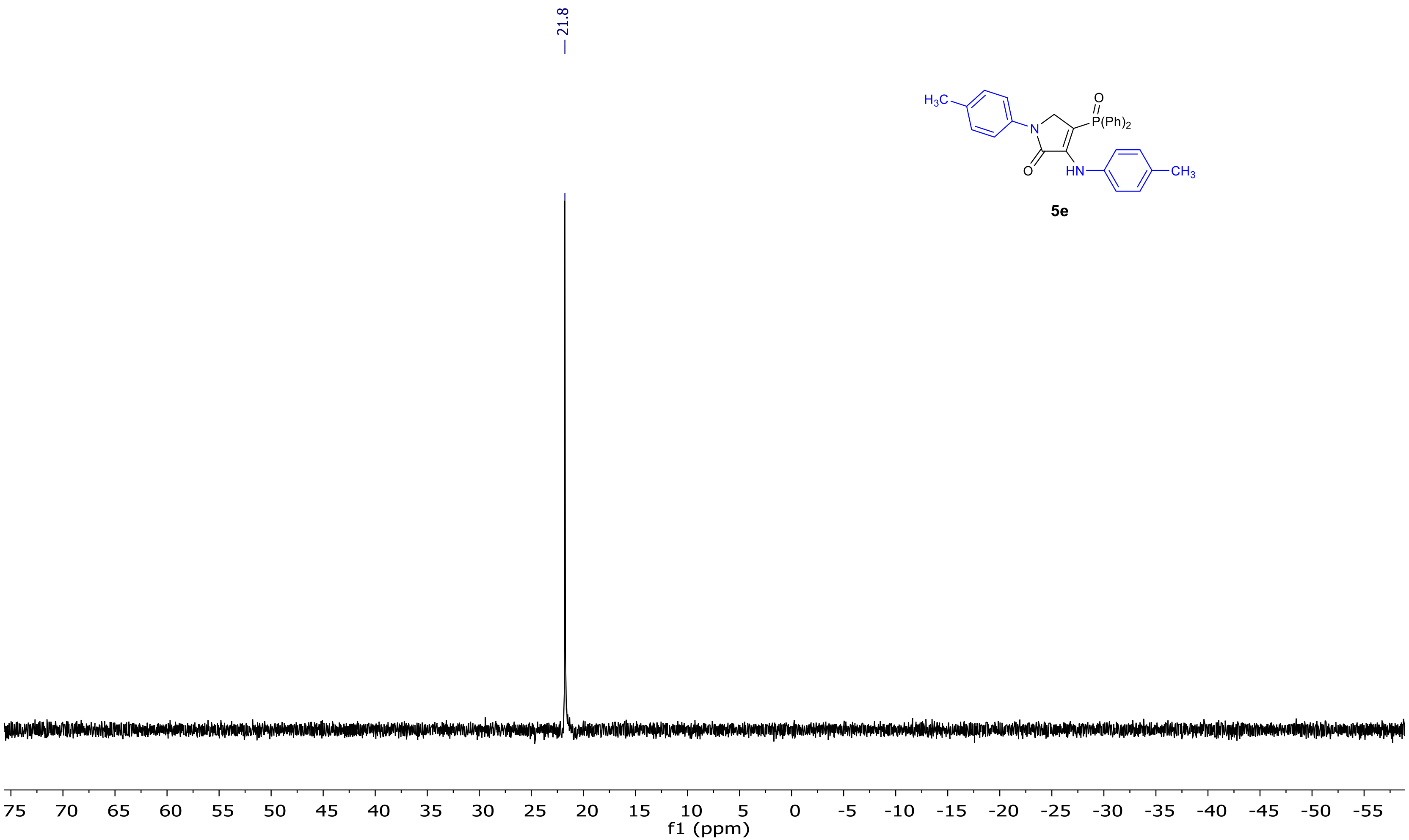

5e

*1-(p-Methoxyphenyl)-3-((p-methoxyphenyl)amino)-5-phenyl-1,5-dihydro-2H-pyrrol-2-one (**6a**).*

^{1}H NMR (400 MHz, $CDCl_3$)

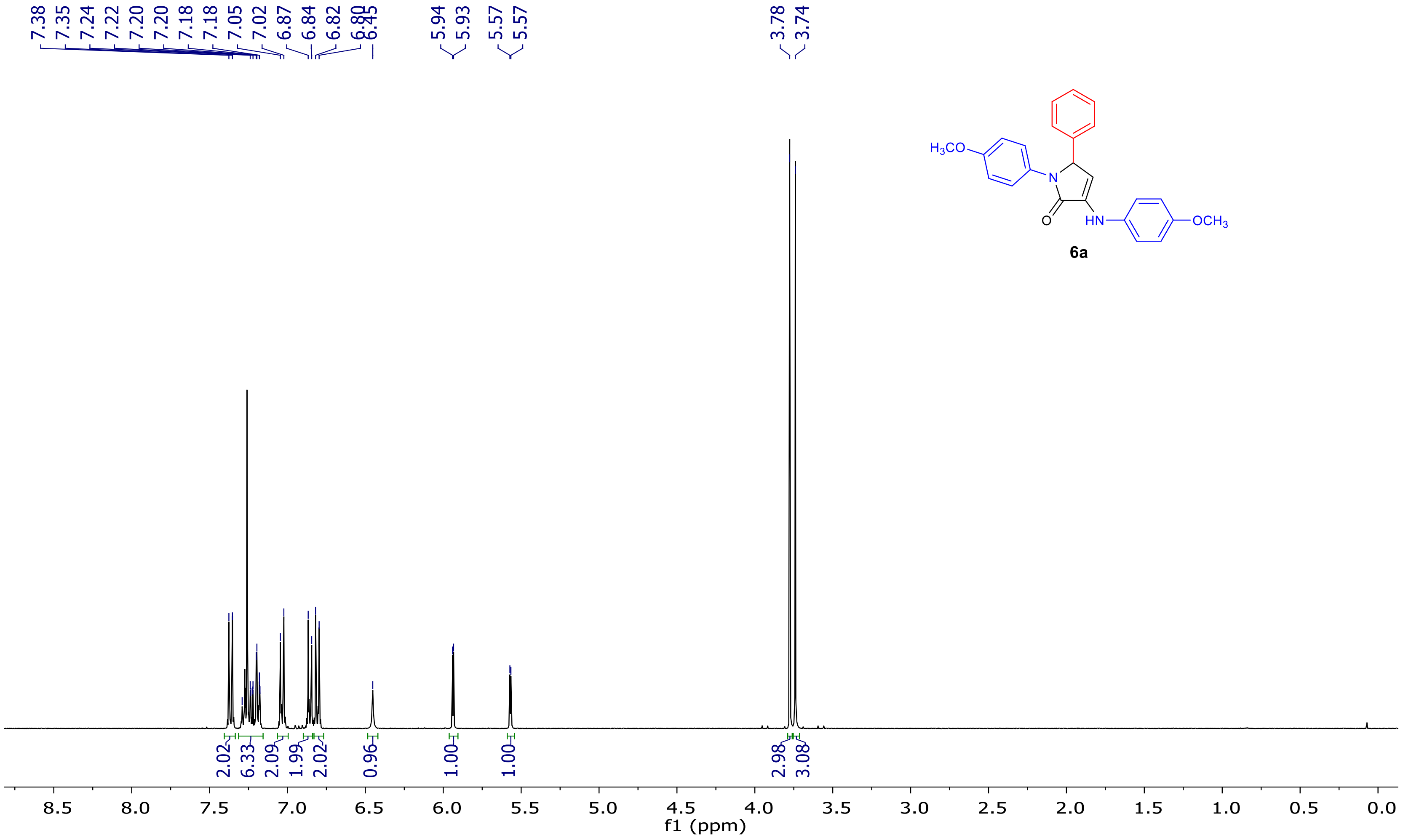

^{13}**C NMR** (101 MHz, $CDCl_3$)

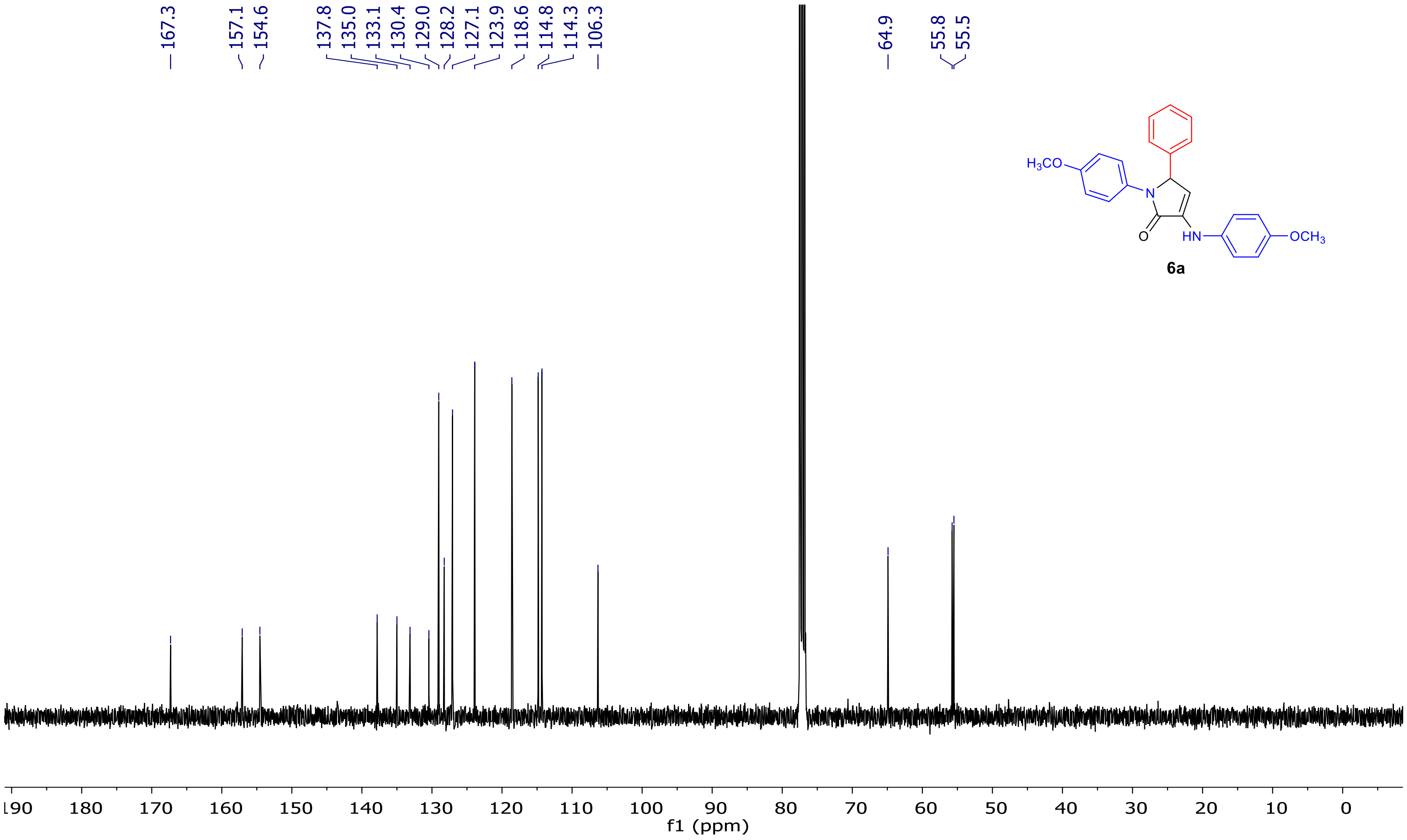

6a

*1-(p-Methoxyphenyl)-3-((p-methoxyphenyl)amino)-5-(p-nitrophenyl)-1H-pyrrol-2(5H)-one (**6b**).*

^{1}H NMR (400 MHz, $CDCl_3$)

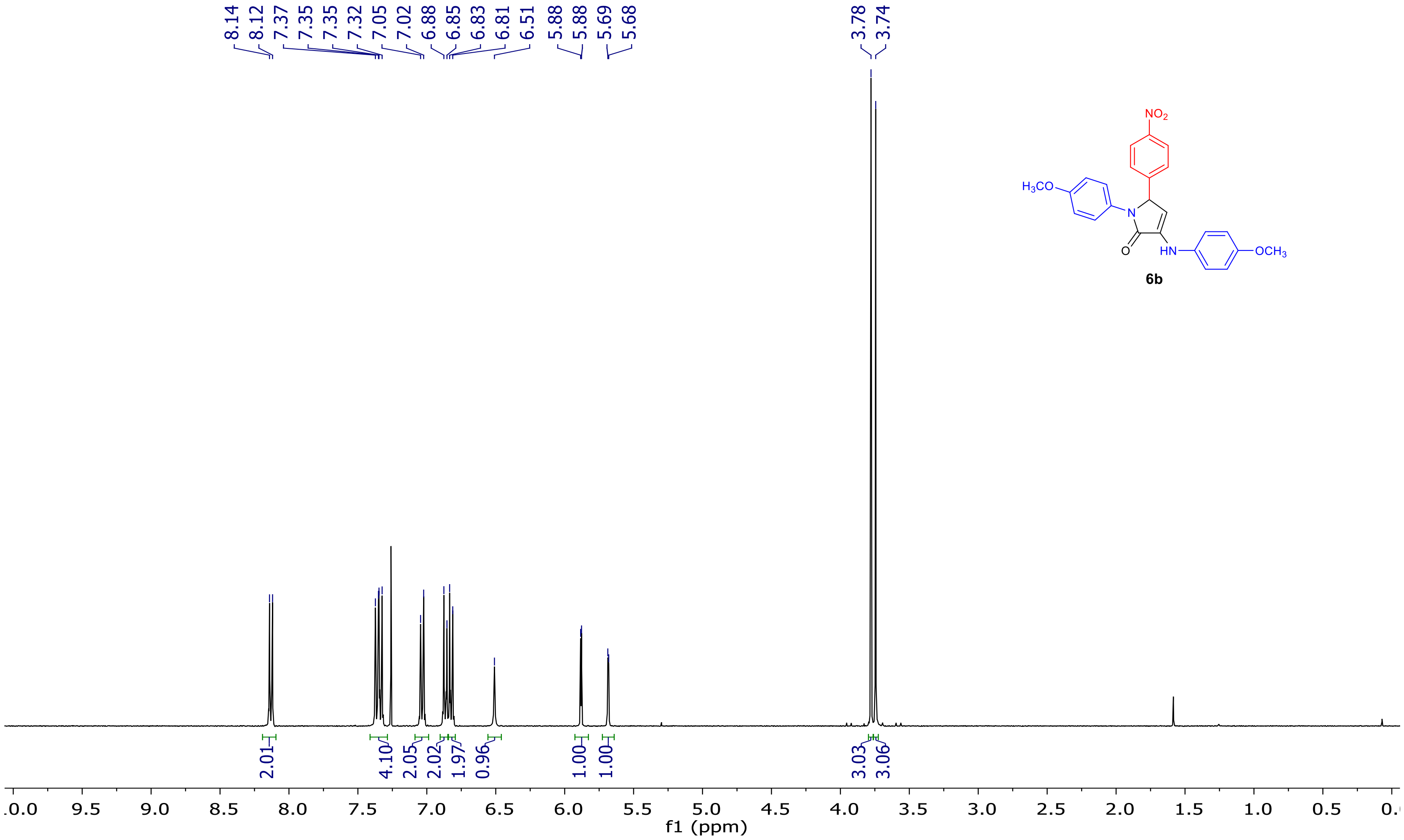

6b

^{13}**C NMR** (101 MHz, $CDCl_3$)

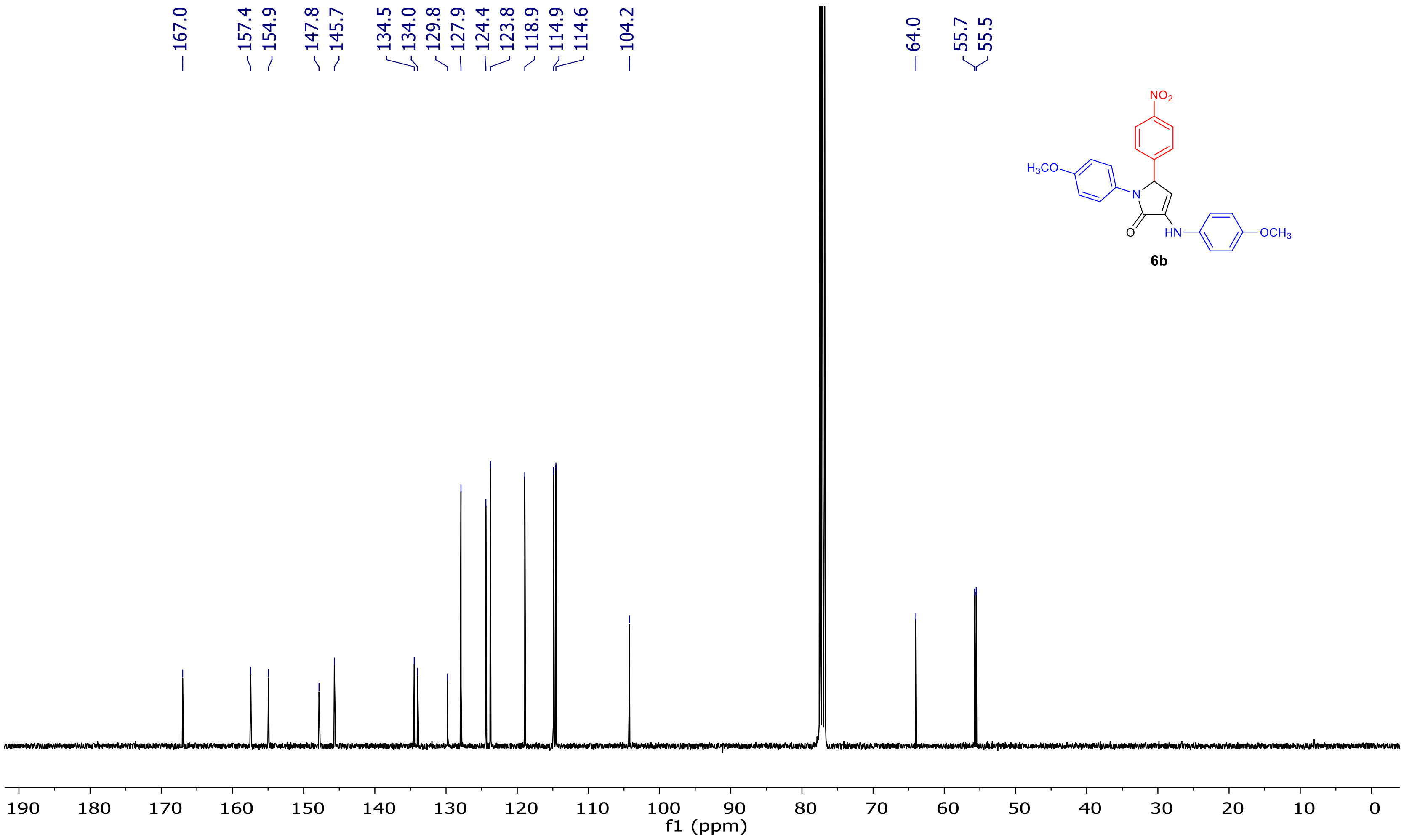

*Diethyl ((1-(p-methoxyphenyl)-4-((p-methoxyphenyl)amino)-5-oxo-2,5-dihydro-1H-pyrrol-2-yl)methyl)phosphonate (**6c**).*

^{1}H NMR (300 MHz, $CDCl_3$)

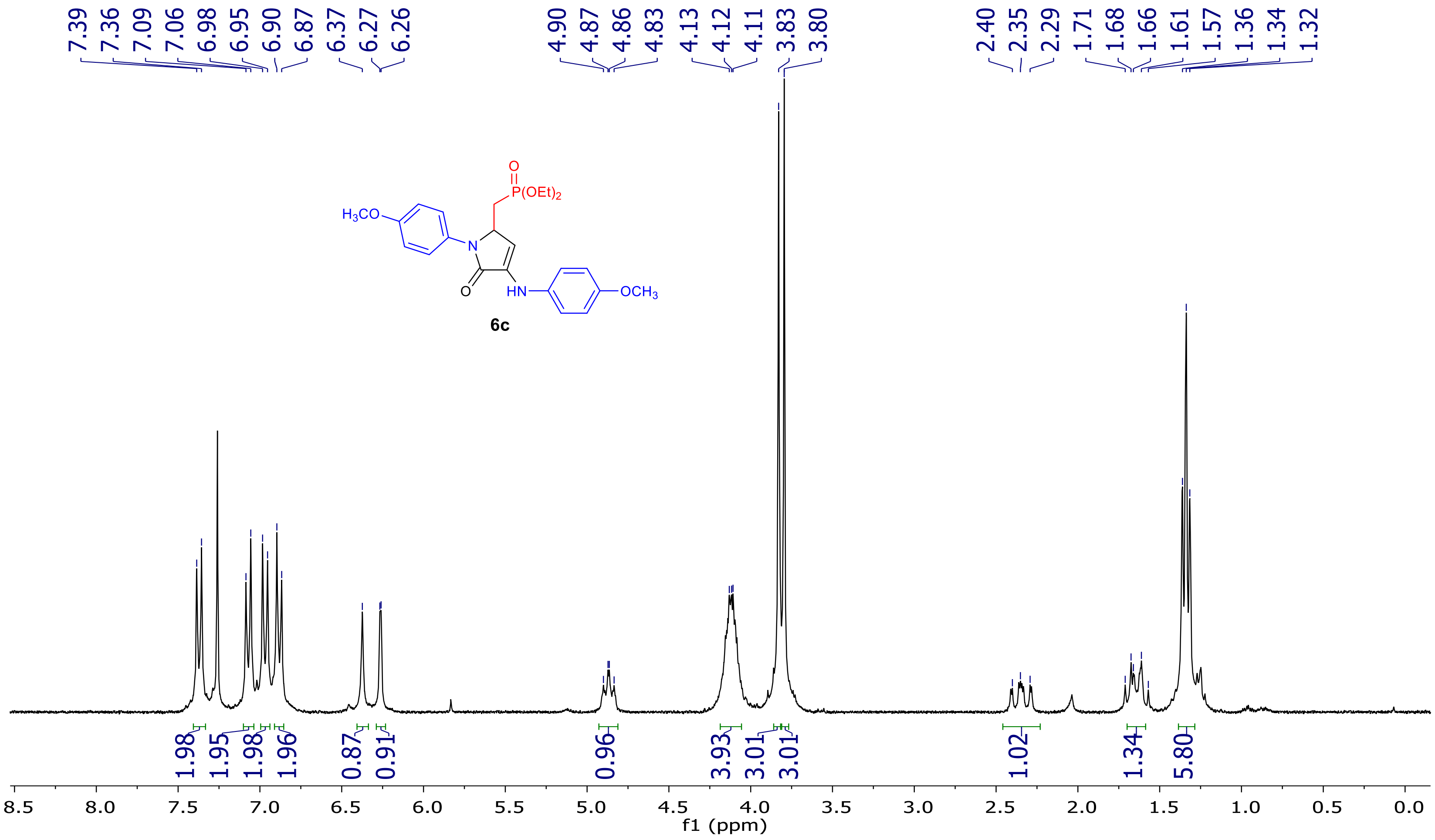

^{13}C NMR (75 MHz, CDCl3)

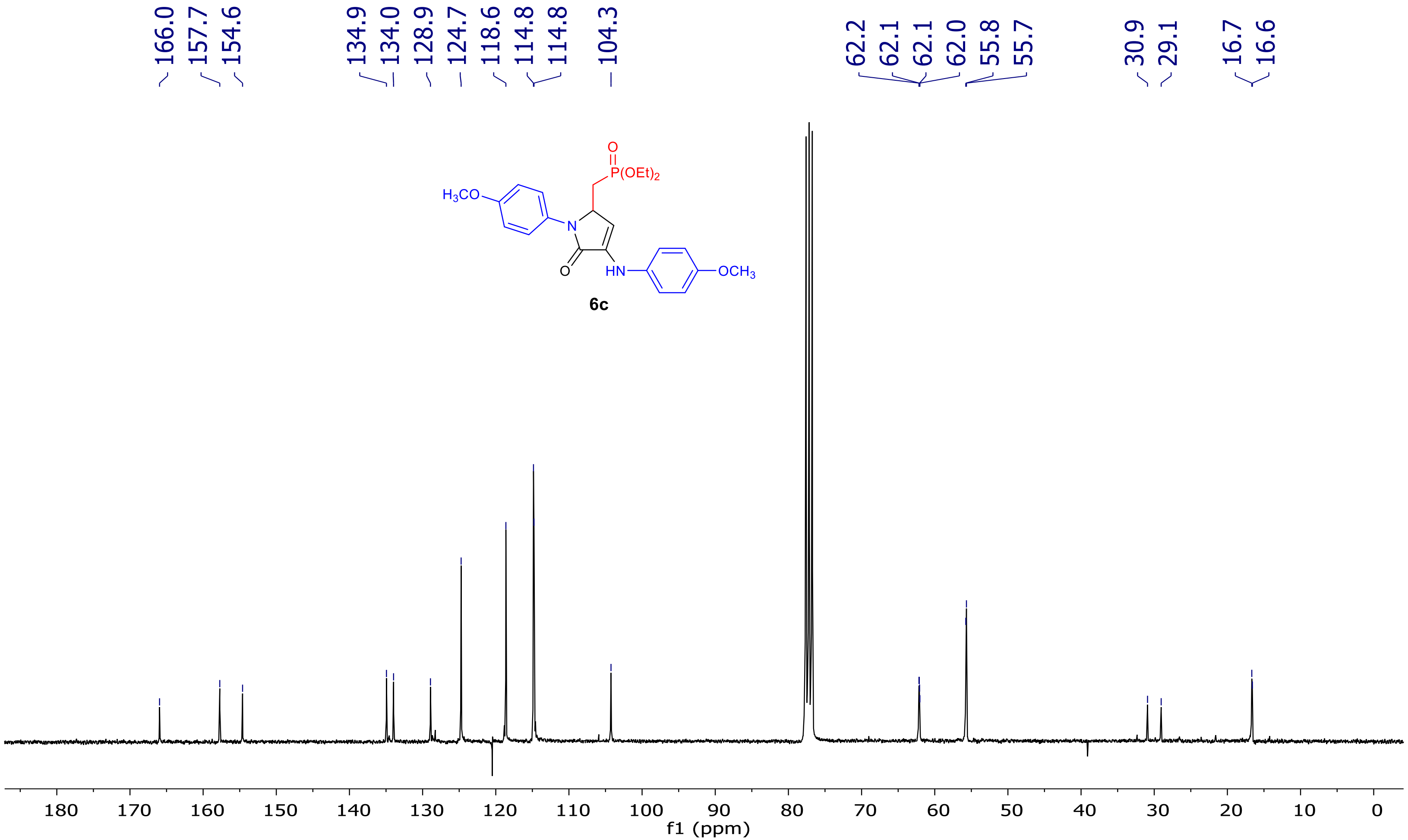

³¹P NMR (121 MHz, CDCl₃)

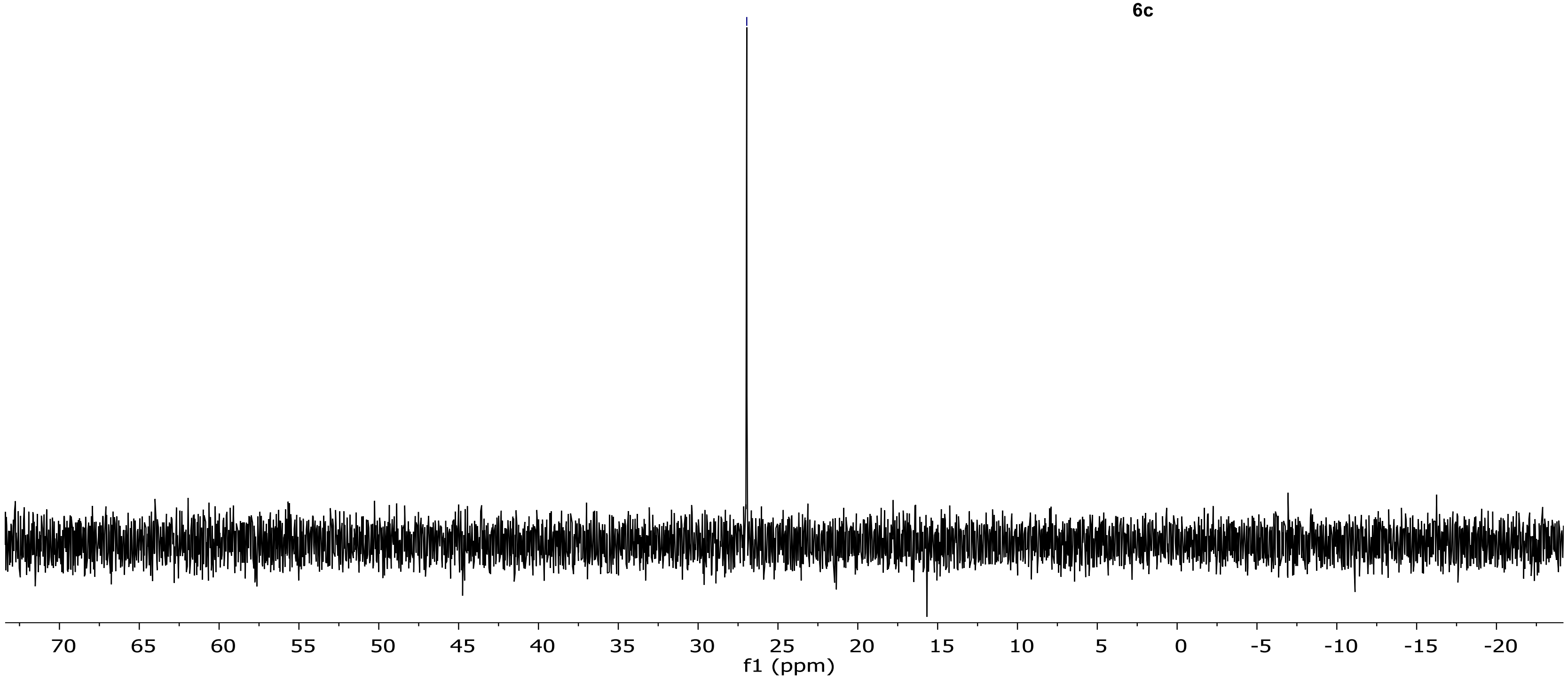

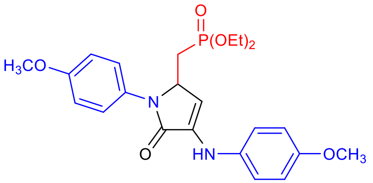

6c

*1-(p-Chlorophenyl)-3-((p-chlorophenyl)amino)-5-(p-nitrophenyl)-1,5-dihydro-2H-pyrrol-2-one (**7**).*
^{1}H NMR (400 MHz, DMSO-d_6)

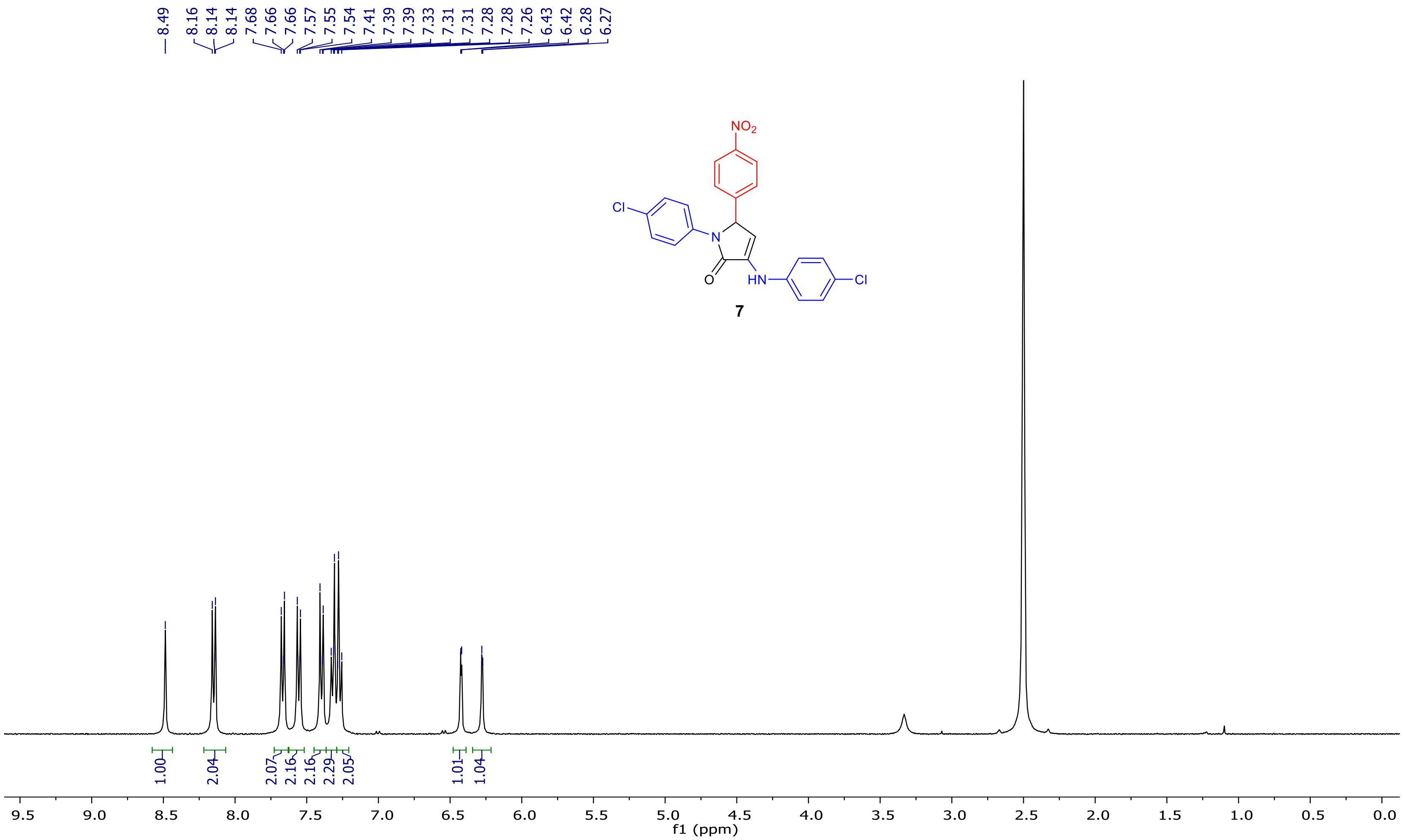

^{13}CNMR (101 MHz, DMSO-*d*$_6$)

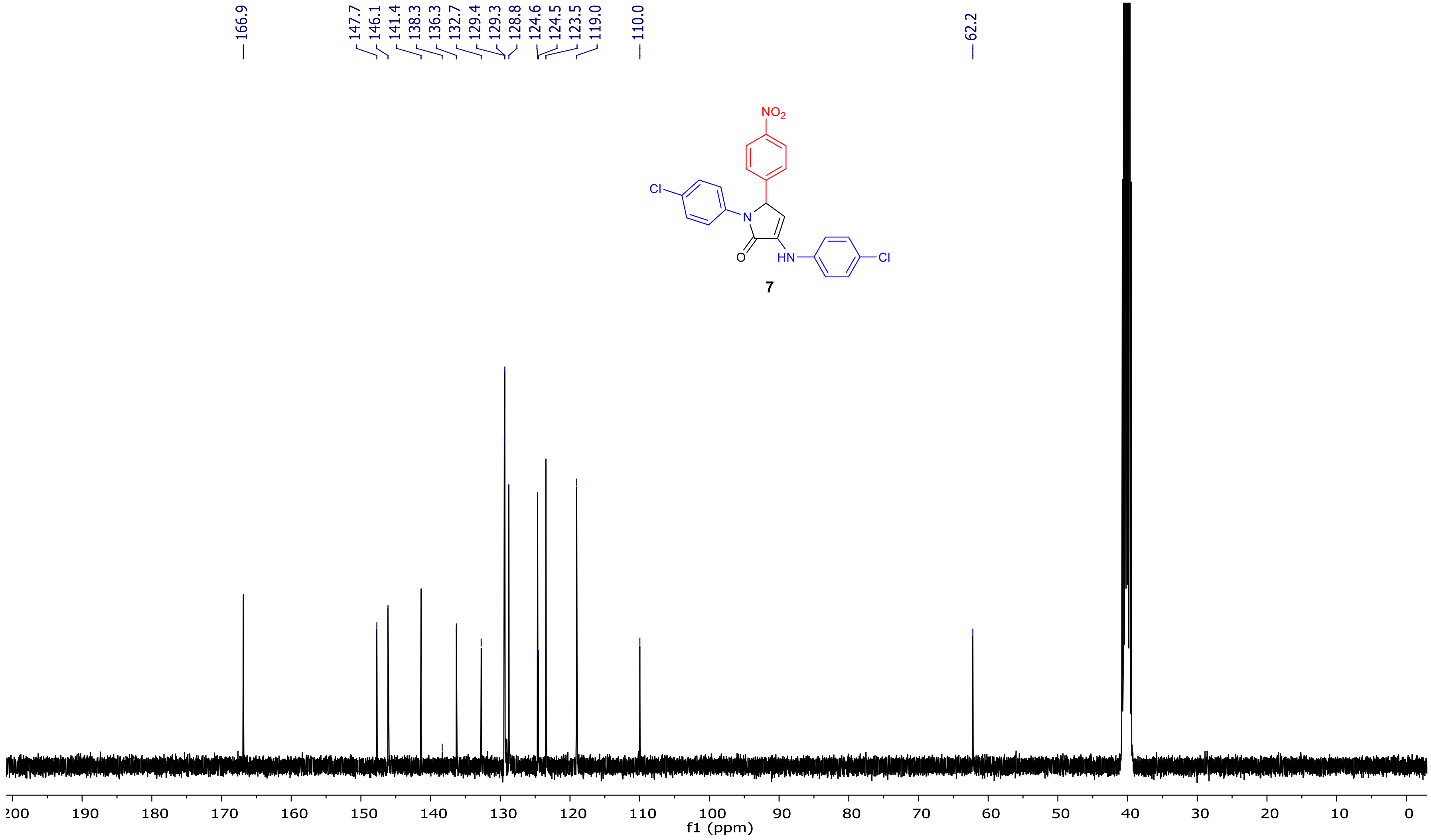

*1,3-bis(p-Bromophenyl)-5-(p-nitrophenyl)-1H-pyrrol-2(5H)-one (**8a**).*

^{1}H NMR (300 MHz, DMSO-*d*6)

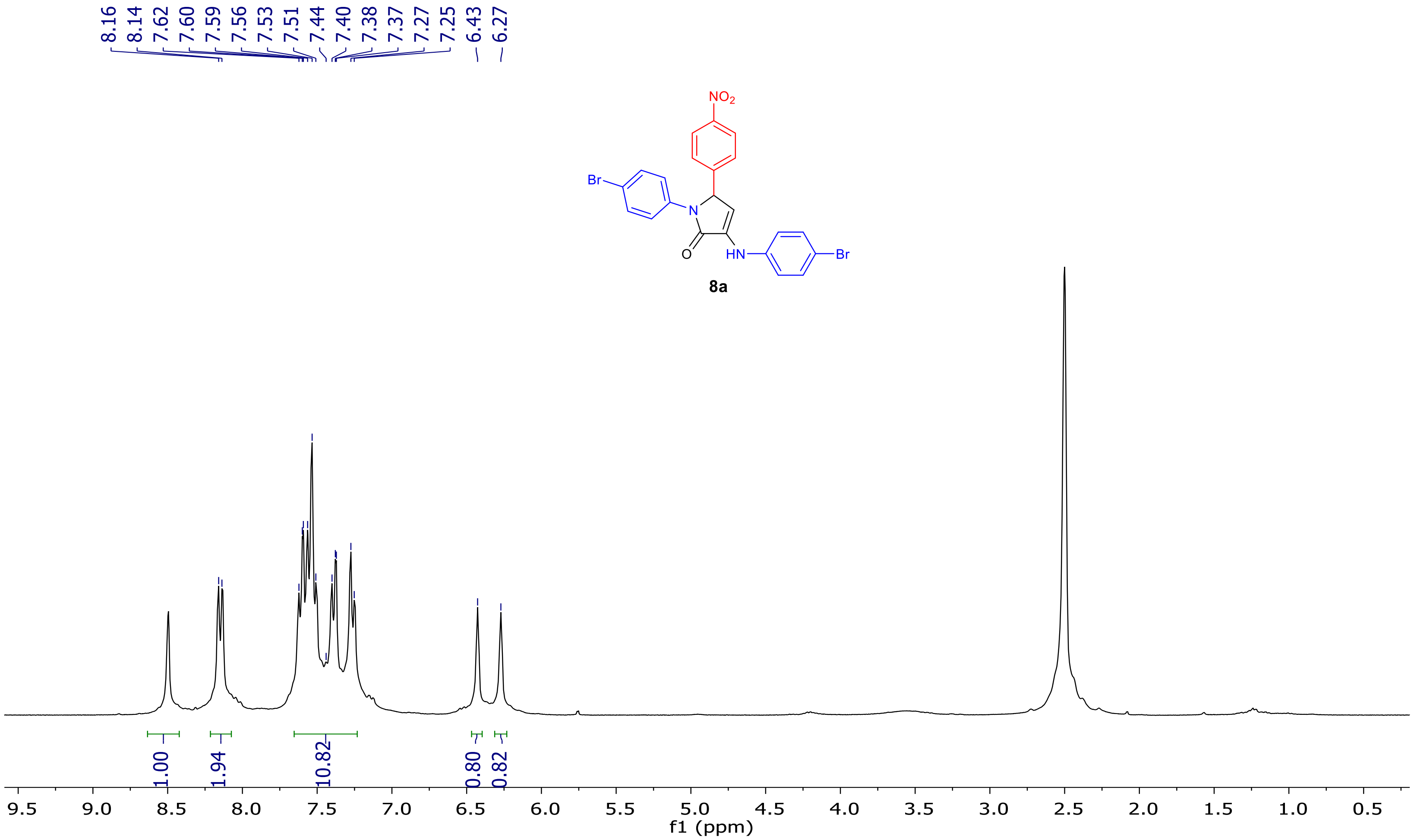

^{13}**C NMR** (75 MHz, DMSO-*d*$_6$)

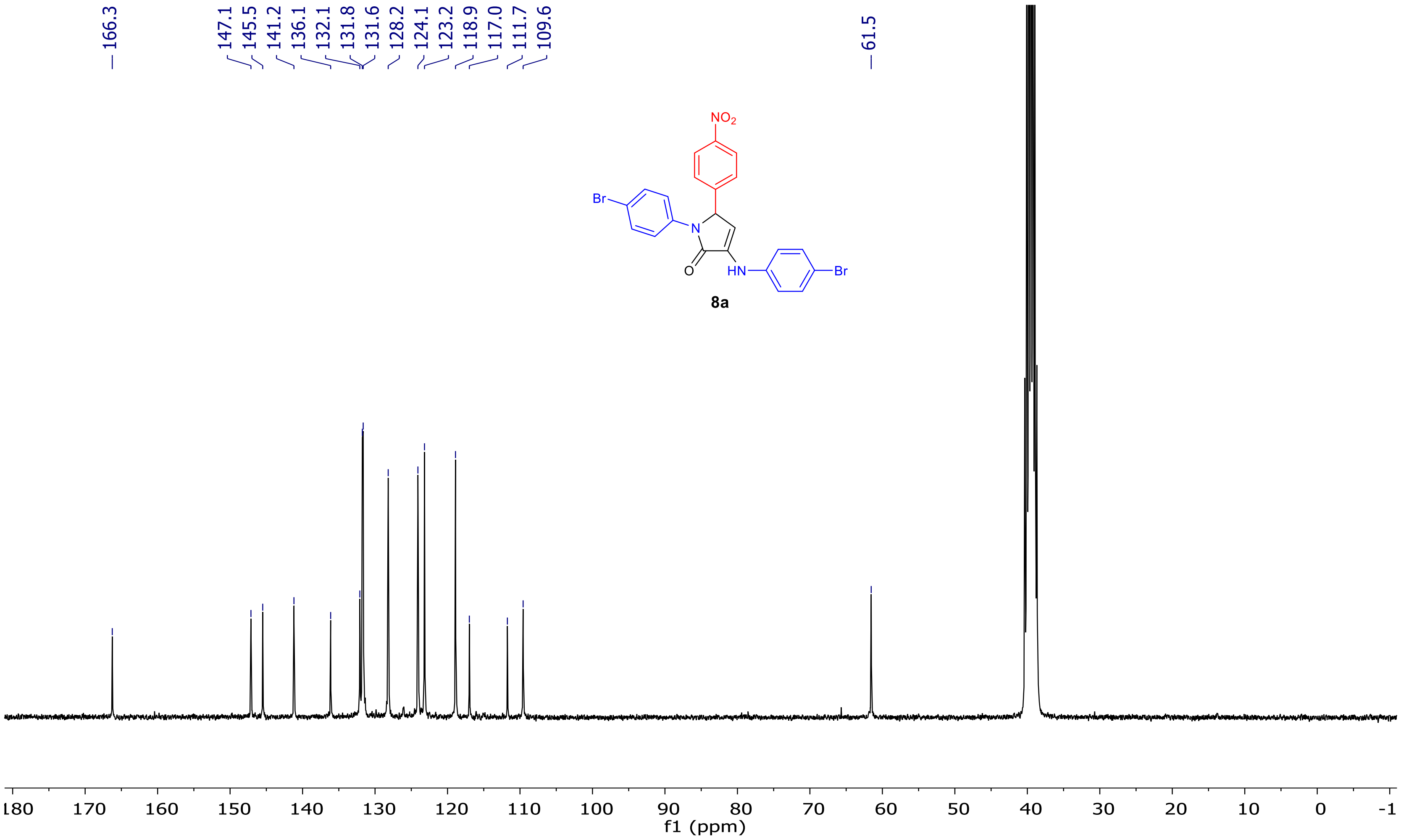

*1-(p-Bromophenyl)-3-((p-bromophenyl)amino)-5-(p-(trifluoromethyl)phenyl)-1,5-dihydro-2H-pyrrol-2-one (**8b**).*

^{1}H NMR (400 MHz, $CDCl_3$)

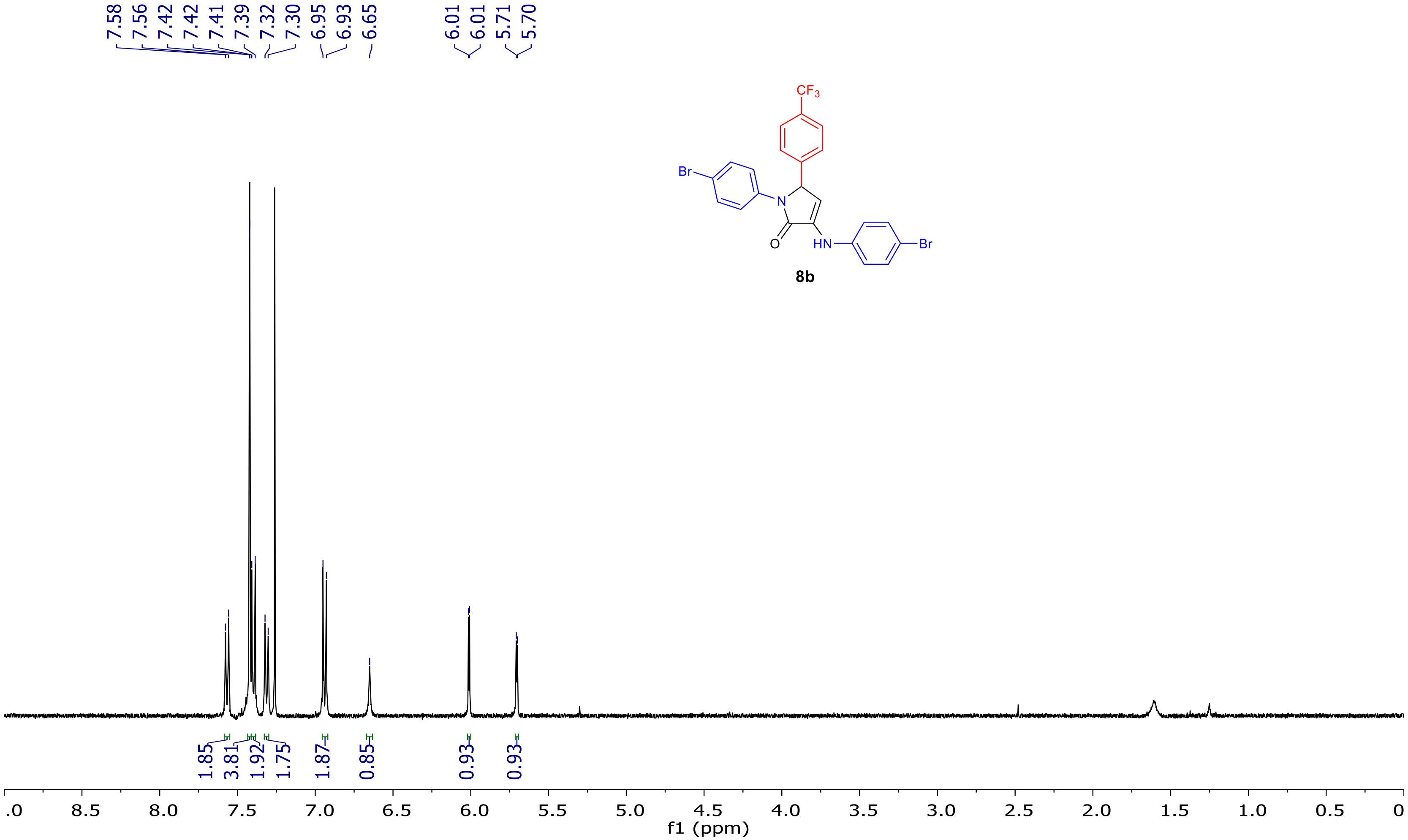

^{13}C NMR (100 MHz, $CDCl_3$)

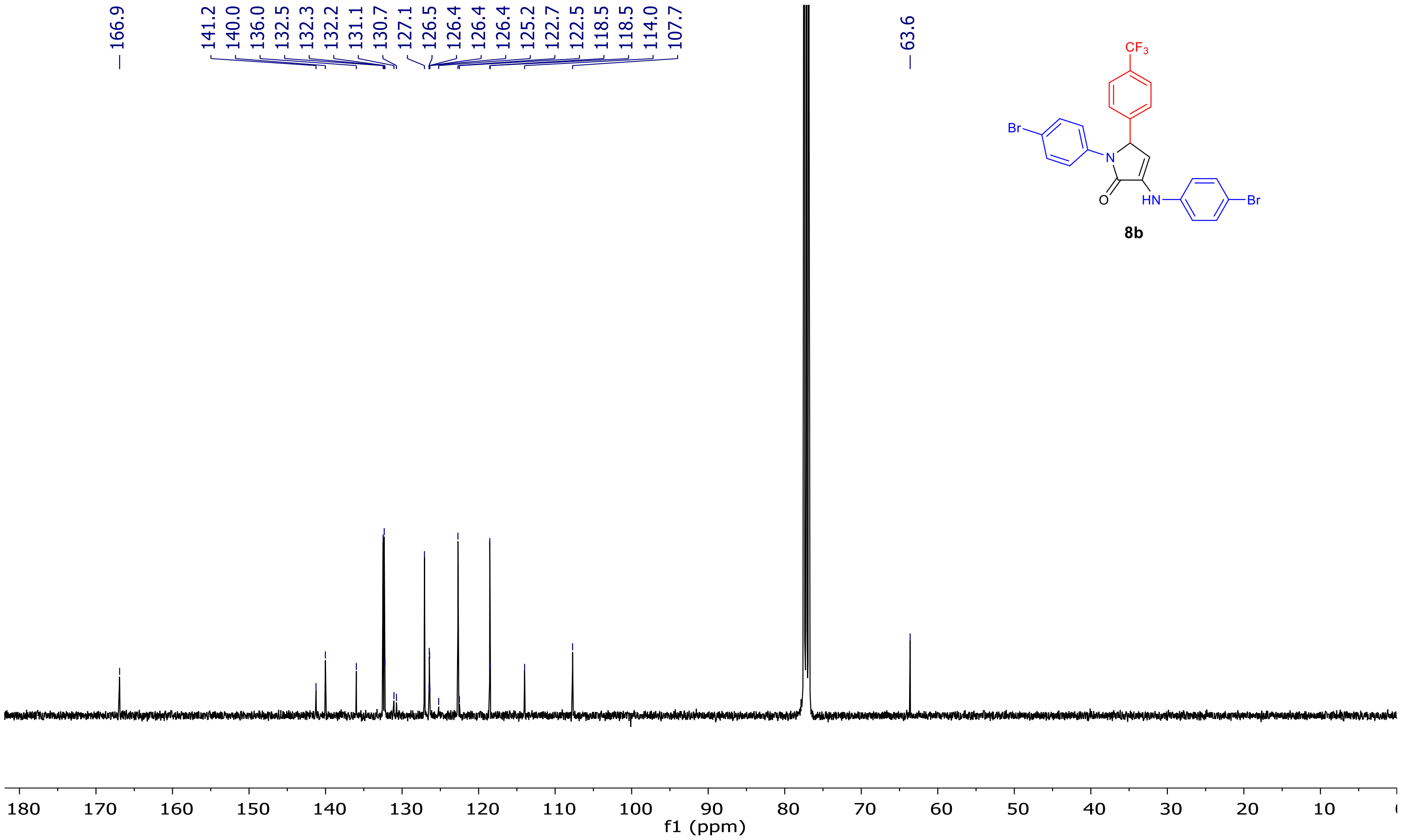

8b

^{19}F NMR (282 MHz, $CDCl_3$)

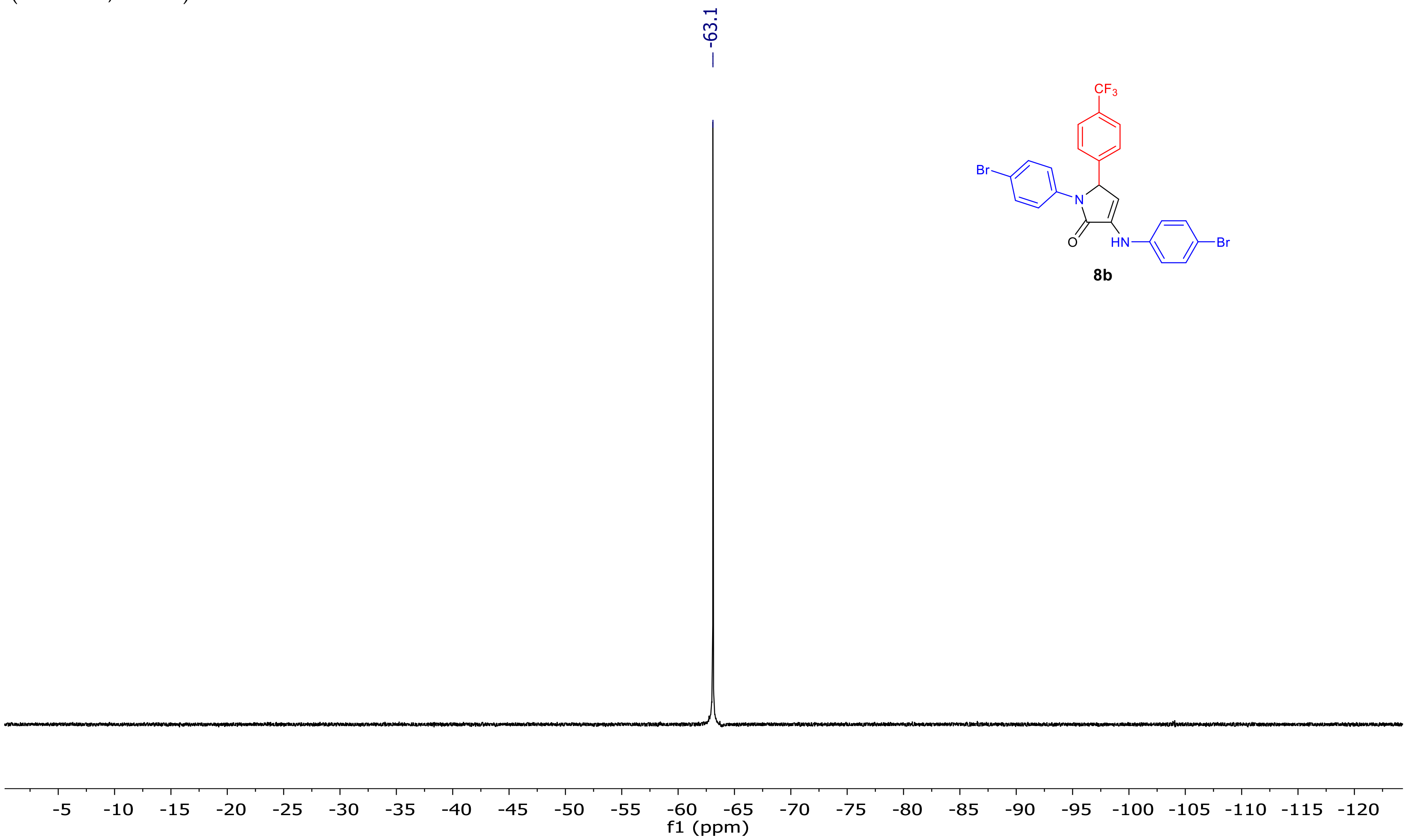

1-(m-Chlorophenyl)-3-((m-chlorophenyl)amino)-5-(p-nitrophenyl)-1,5-dihydro-2H-pyrrol-2-one (**9**).

^{1}H NMR (400 MHz, $CDCl_3$)

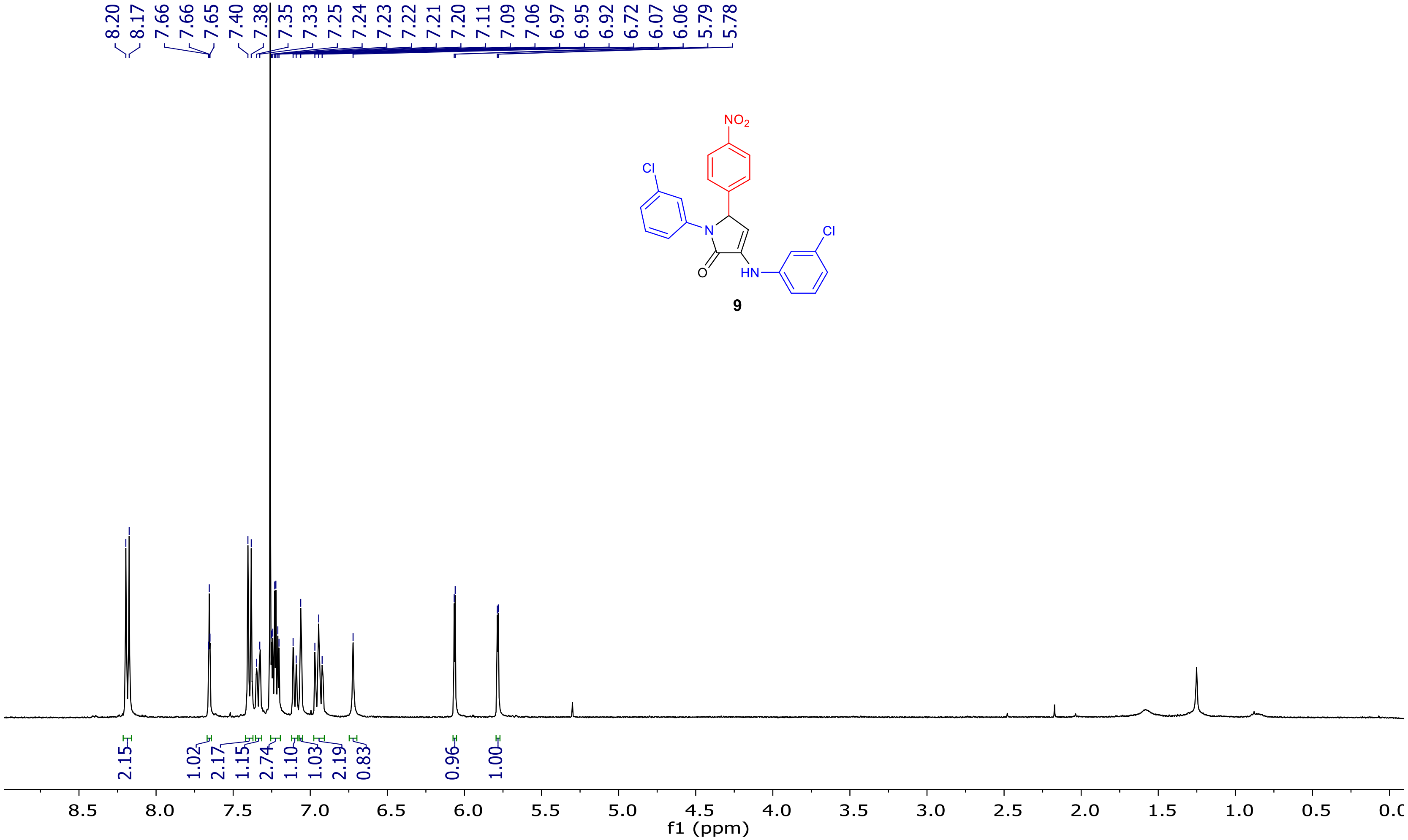

9

^{13}C NMR (100 MHz, $CDCl_3$)

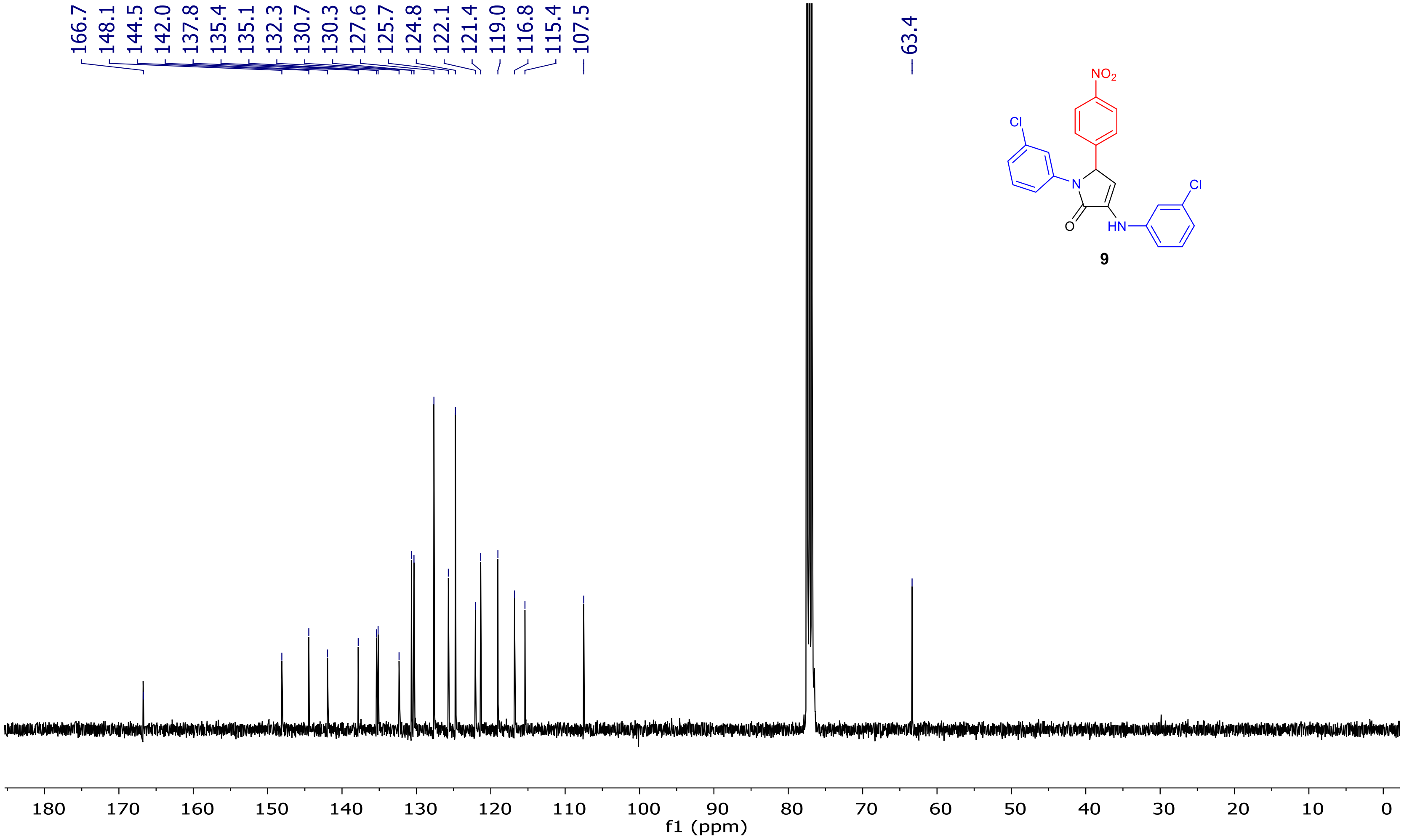

*1-(o-Fluorophenyl)-3-((o-fluorophenyl)amino)-5-(p-nitrophenyl)-1H-pyrrol-2(5H)-one (**10**).*

^{1}H NMR (300 MHz, $CDCl_3$)

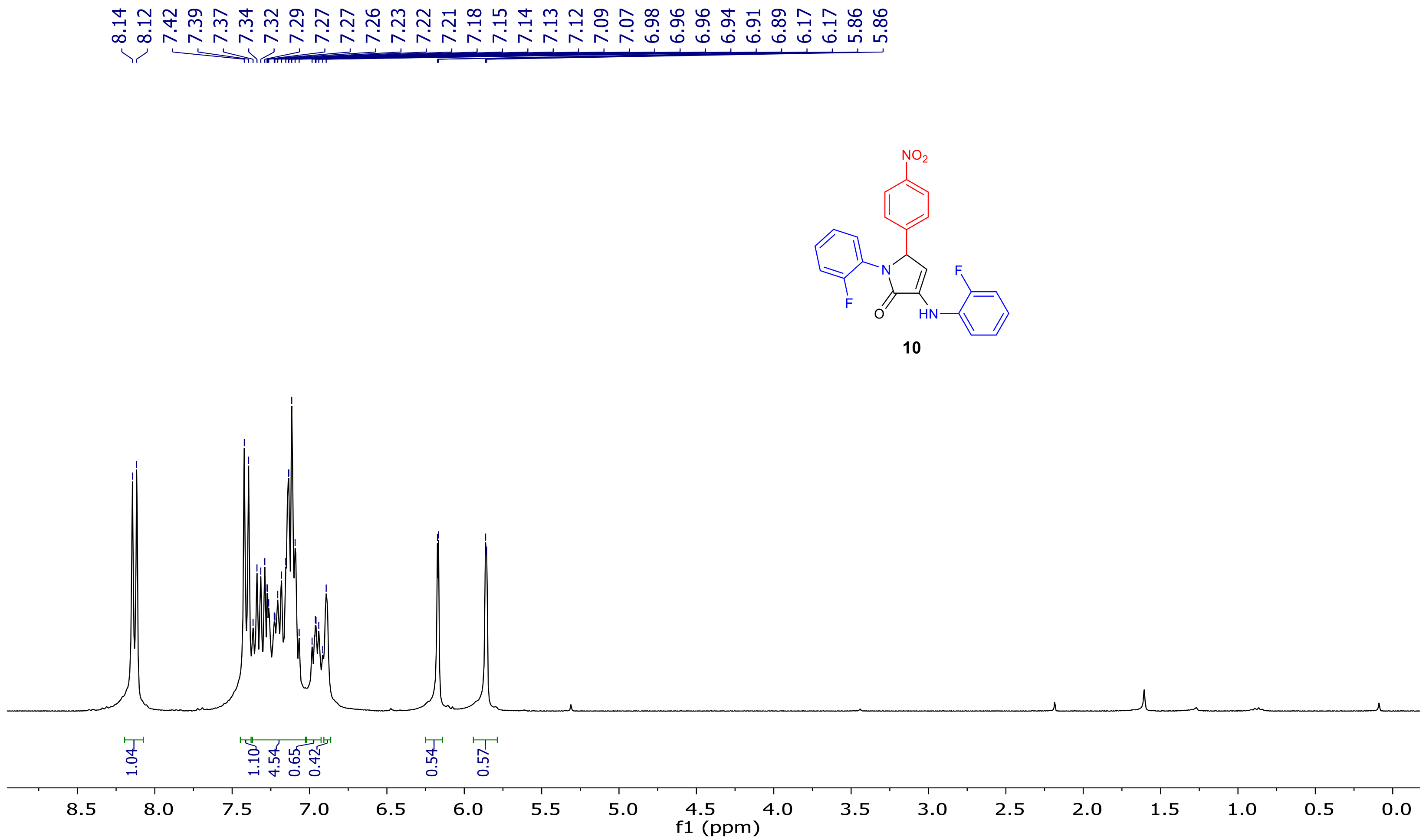

^{13}C NMR (75 MHz, $CDCl_3$)

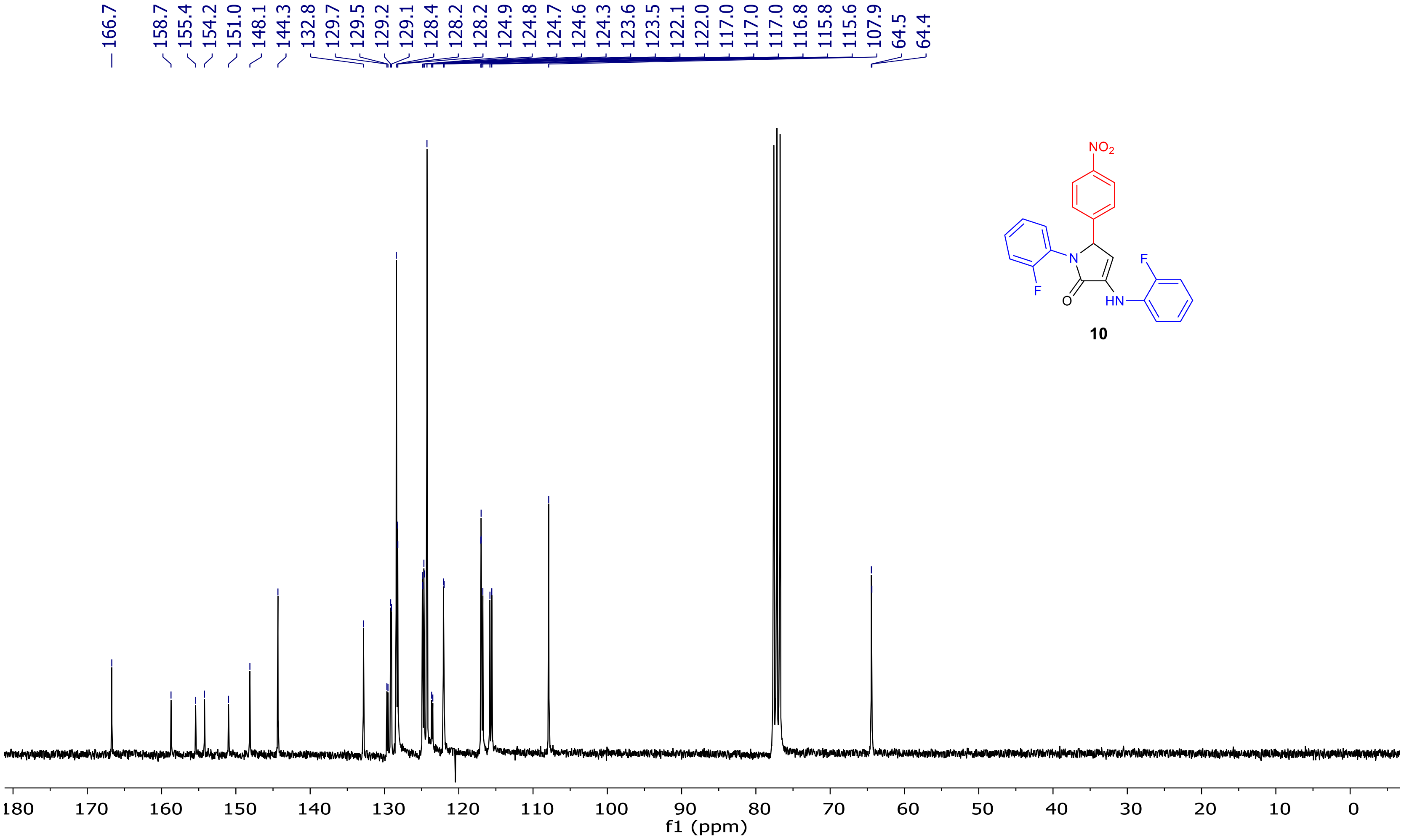

10

^{19}F NMR (282 MHz, $CDCl_3$)

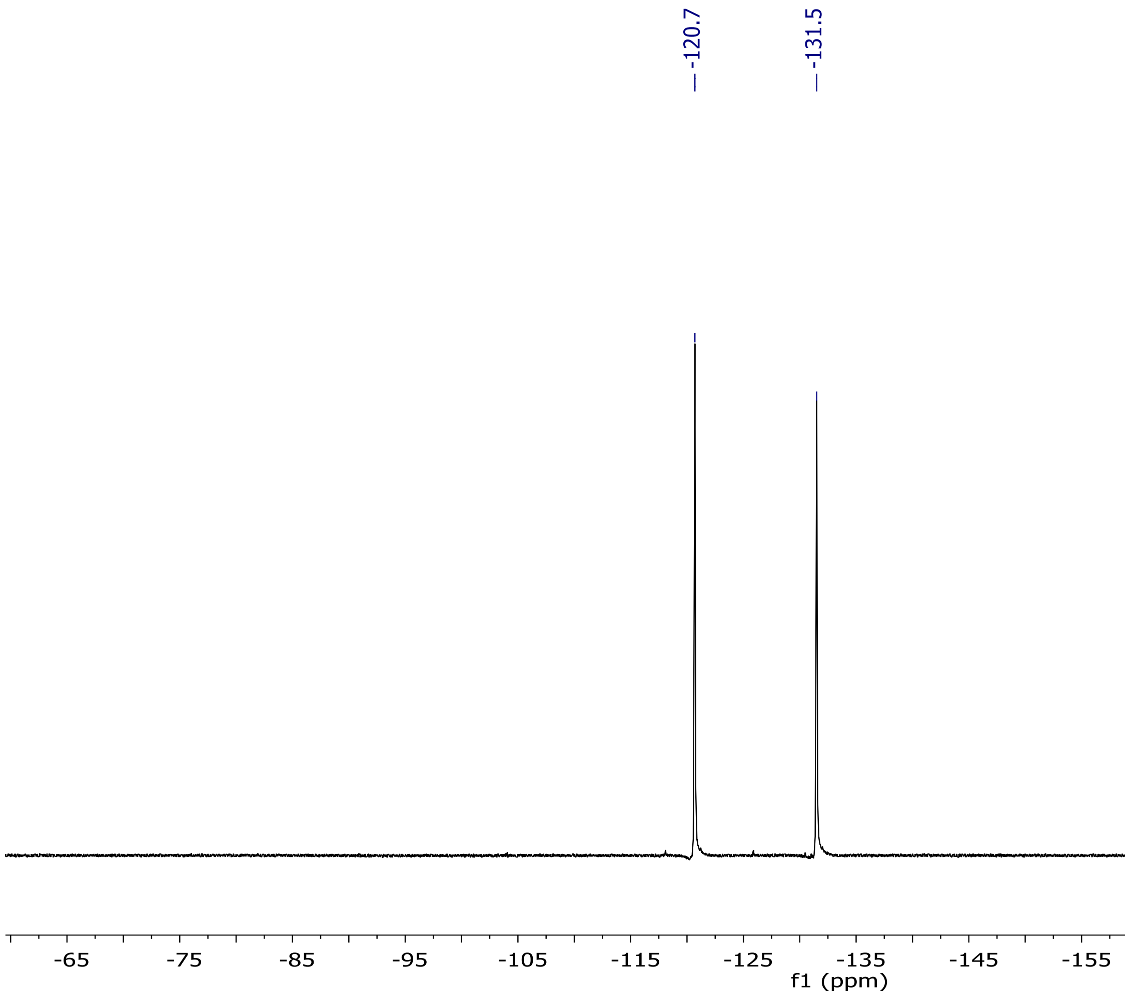

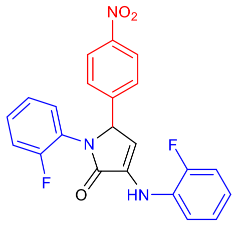

10

*1-(m-(Trifluoromethyl)phenyl)-3-((m-(trifluoromethyl)phenyl)amino)-1,5-dihydro-2H-pyrrol-2-one (**11a**).*

^{1}H NMR (400 MHz, $CDCl_3$)

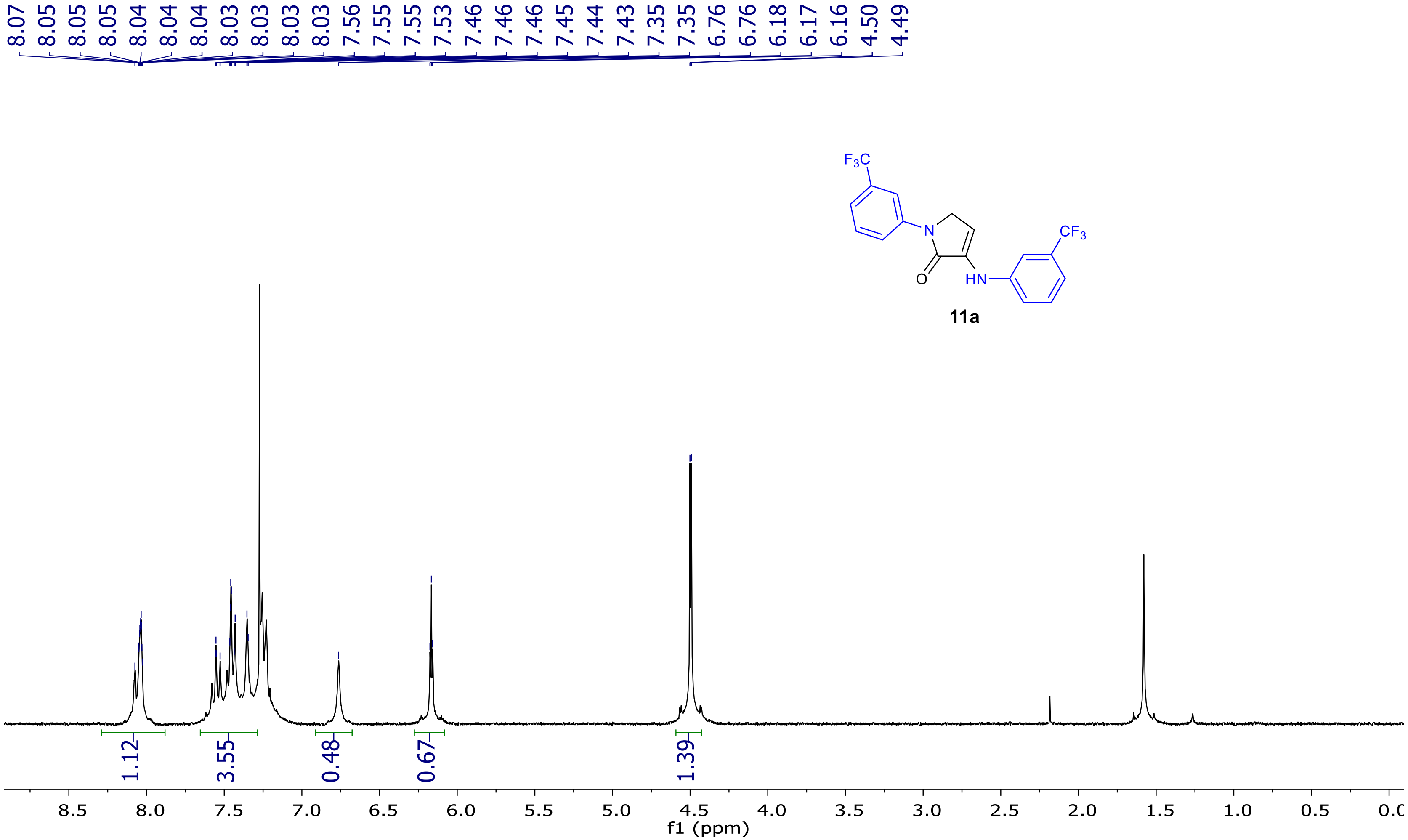

^{13}C NMR (101 MHz, $CDCl_3$)

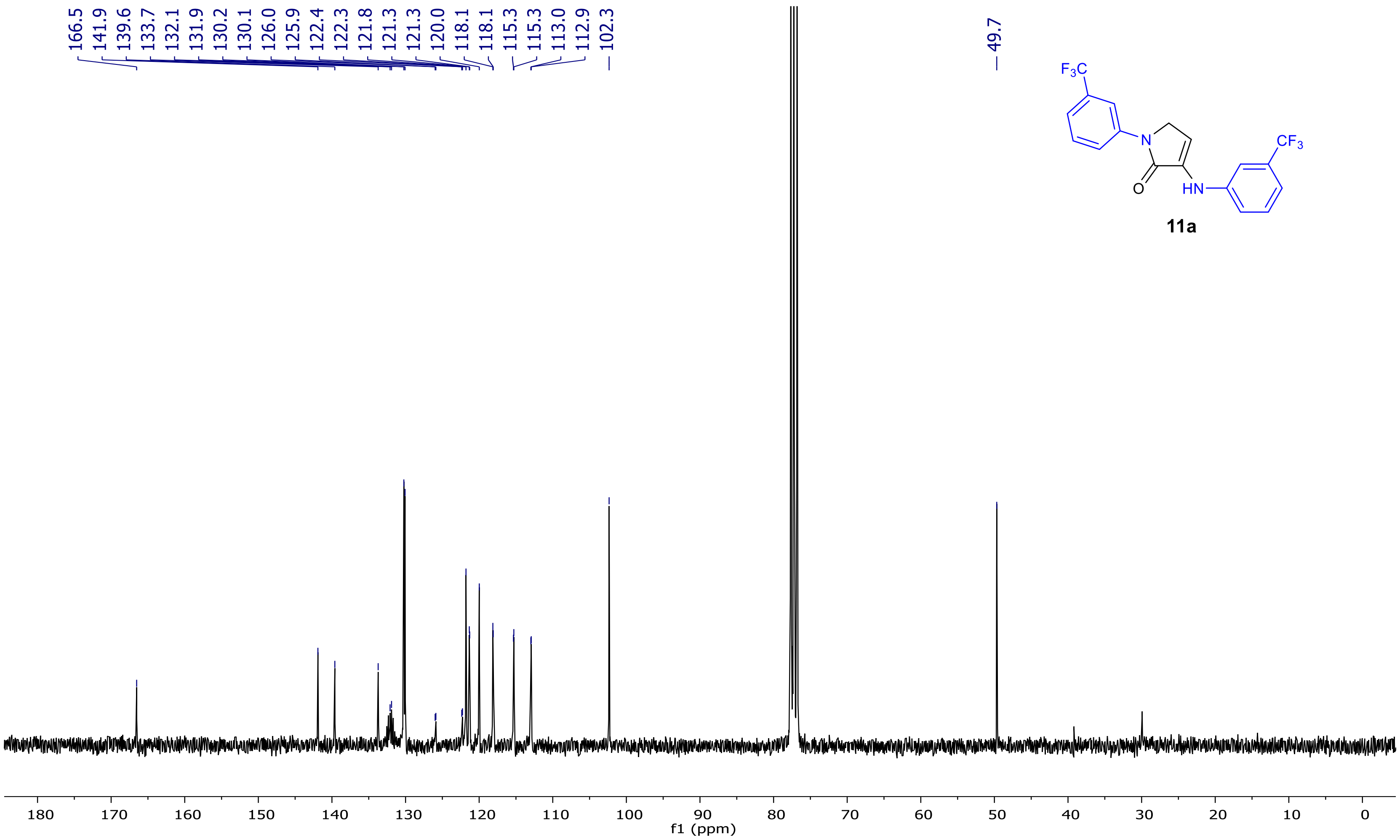

11a

^{19}F NMR (282 MHz, $CDCl_3$)

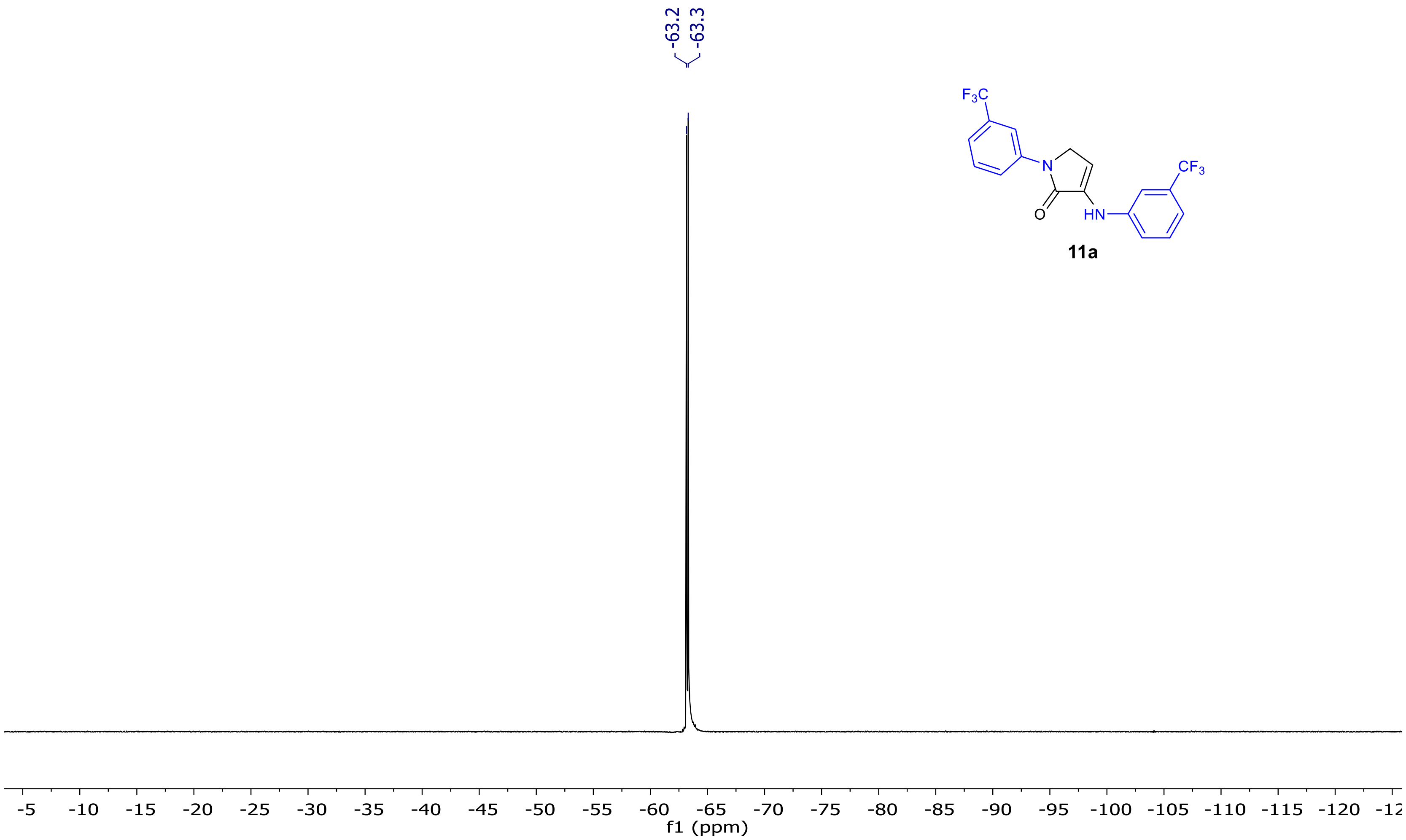

11a

*5-(p-Nitrophenyl)-1-(m-(trifluoromethyl)phenyl)-3-((m-(trifluoromethyl)phenyl)amino)-1H-pyrrol-2(5H)-one (**11b**).*
^{1}H NMR (400 MHz, $CDCl_3$)

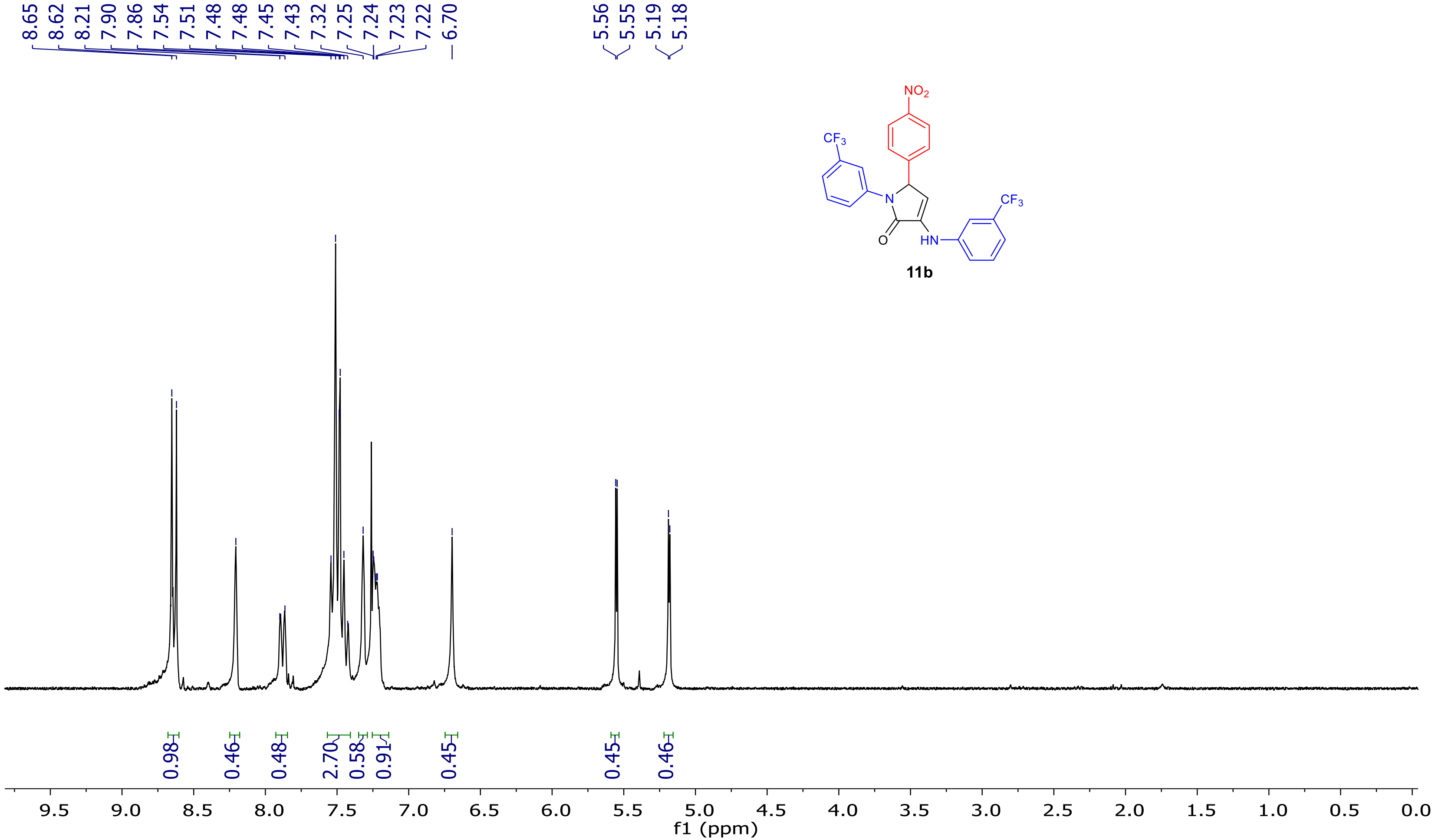

^{13}C NMR (100 MHz, $CDCl_3$)

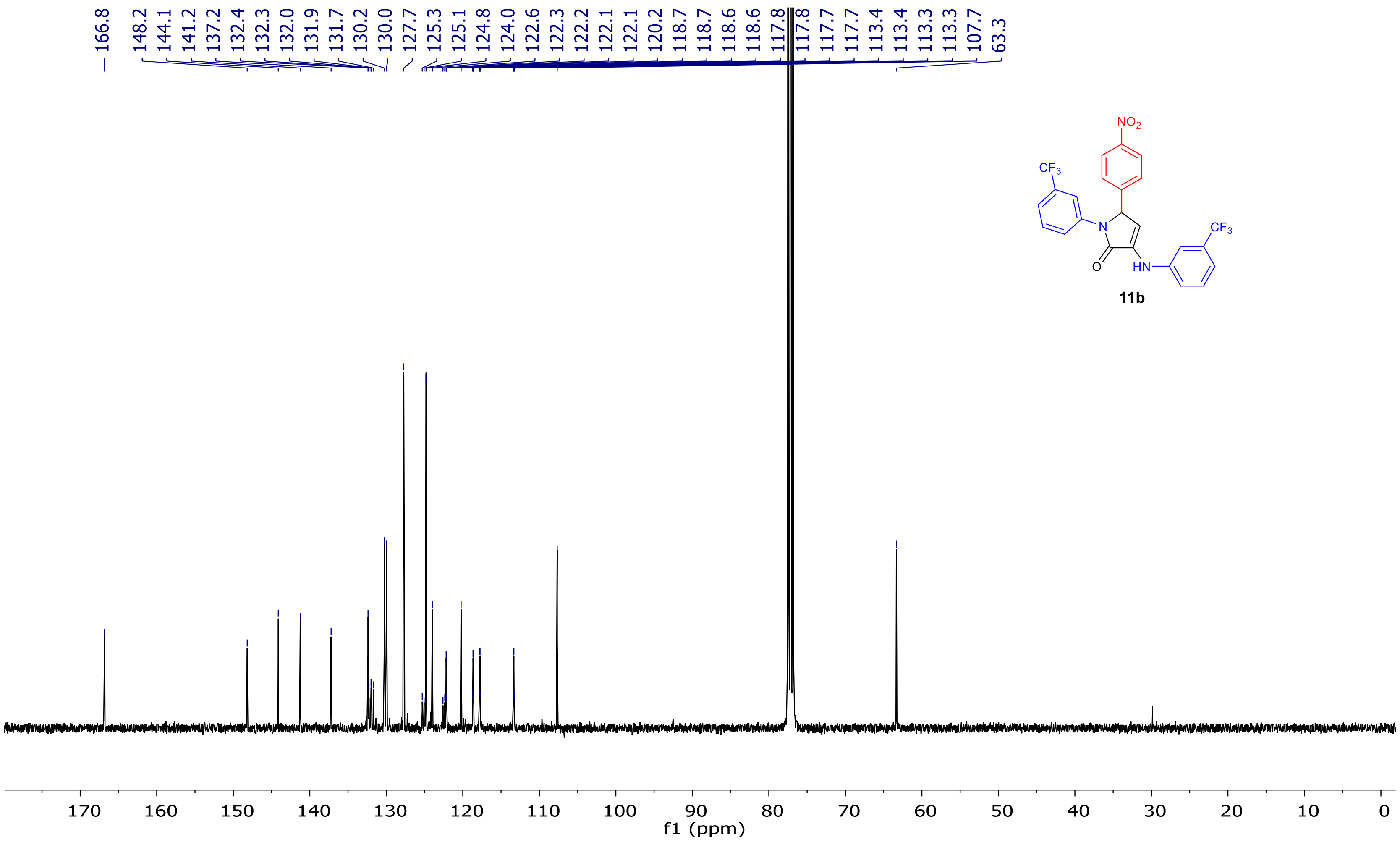

^{19}F NMR (282 MHz, $CDCl_3$)

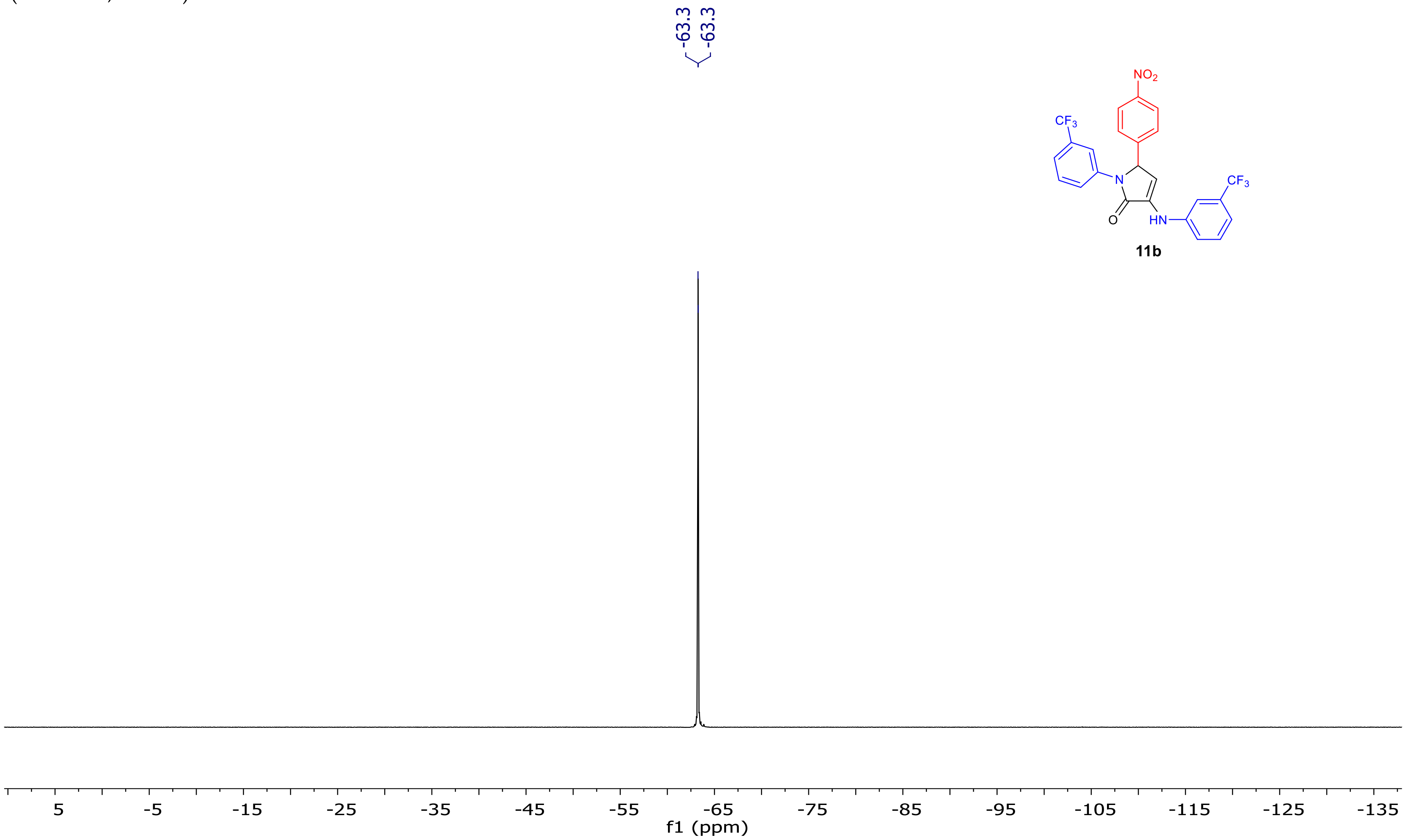

*5-(p-Nitrophenyl)-1-(quinolin-3-yl)-3-(quinolin-3-ylamino)-1H-pyrrol-2(5H)-one (**12**).*

^{1}H NMR (400 MHz, $CDCl_3$)

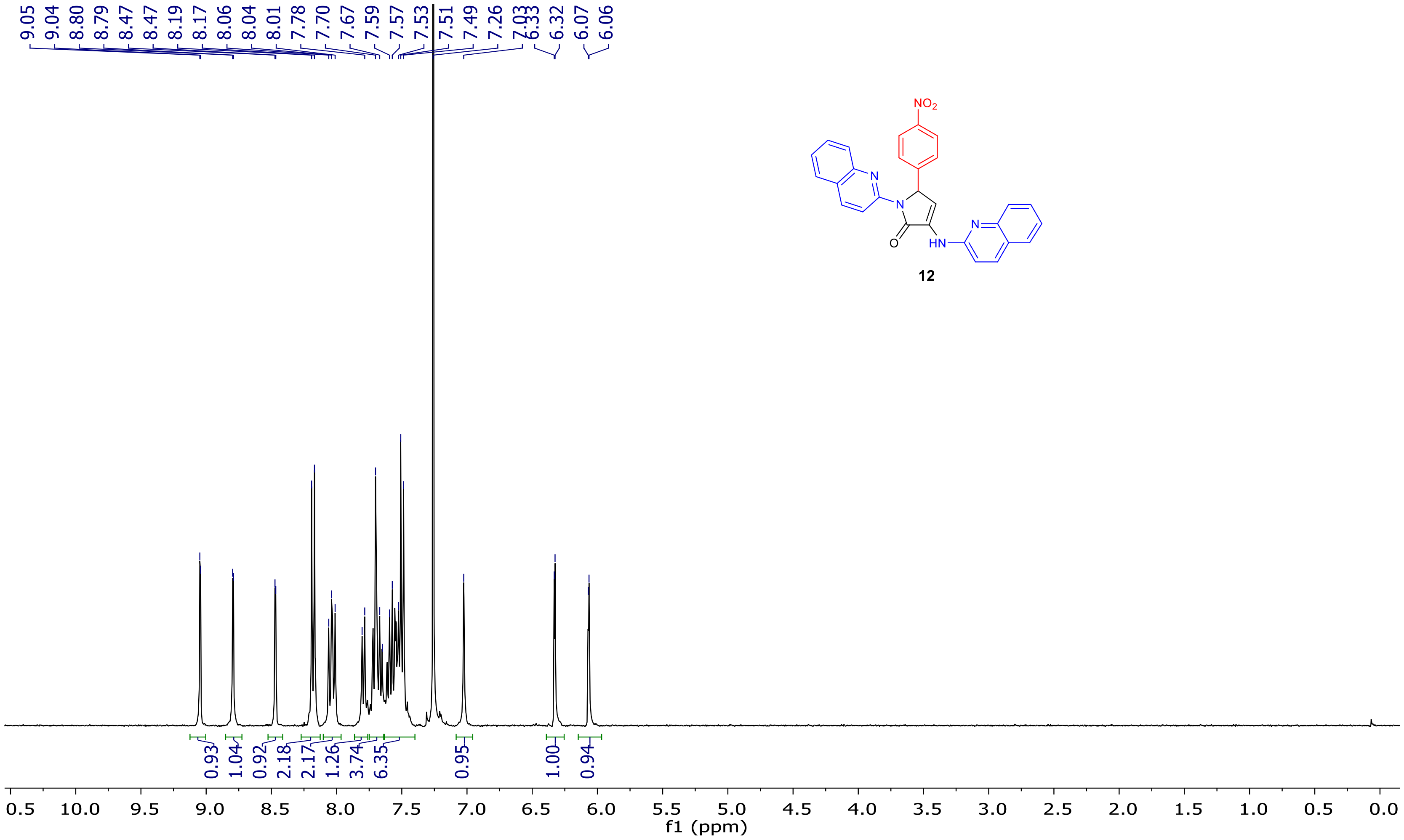

12

^{13}C NMR (101 MHz, $CDCl_3$)

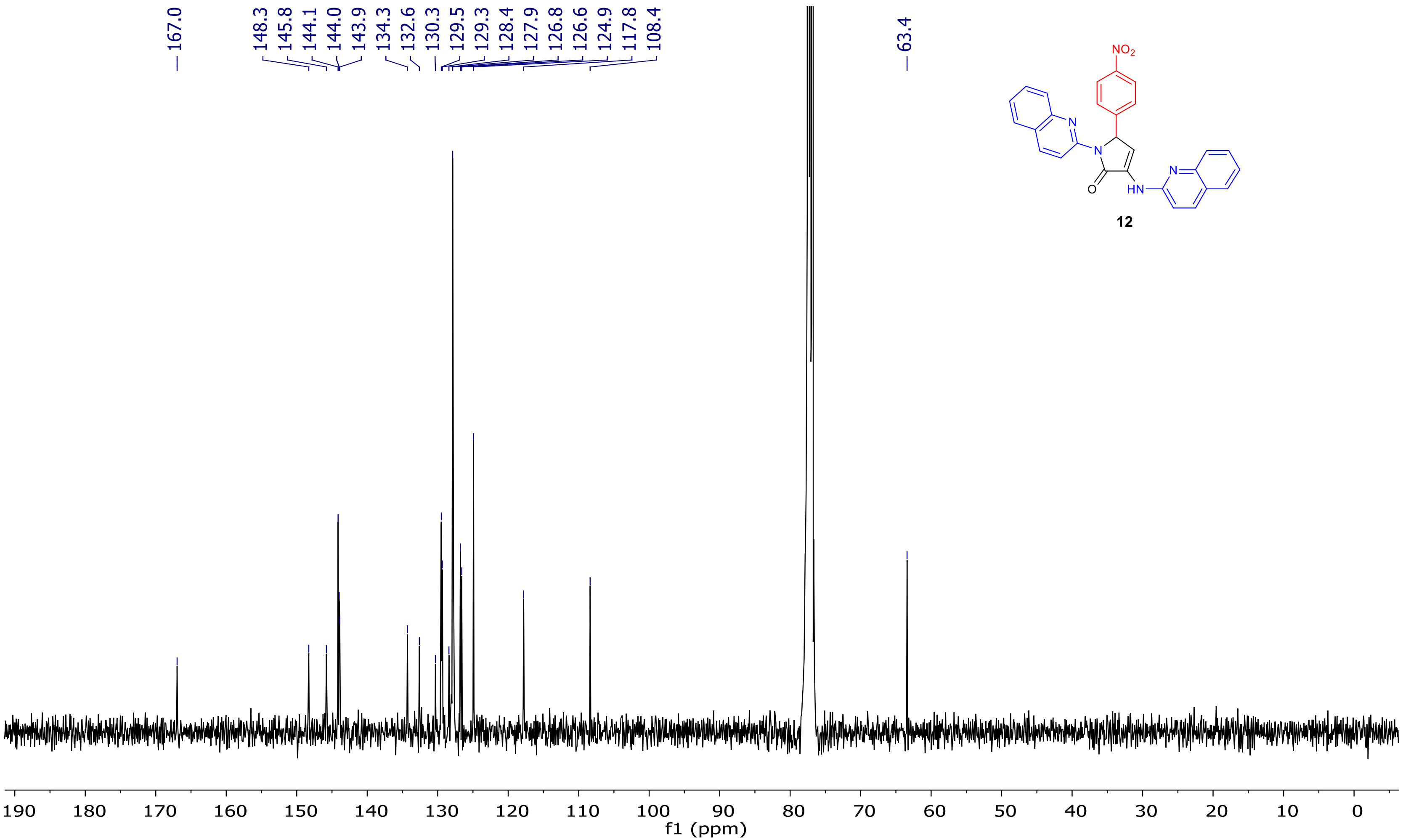

*Ethyl 2-methyl-5-oxo-1-(p-tolyl)-4-(p-tolylamino)-2,5-dihydro-1H-pyrrole-2-carboxylate (**16**).*

^{1}H NMR (300 MHz, $CDCl_3$)

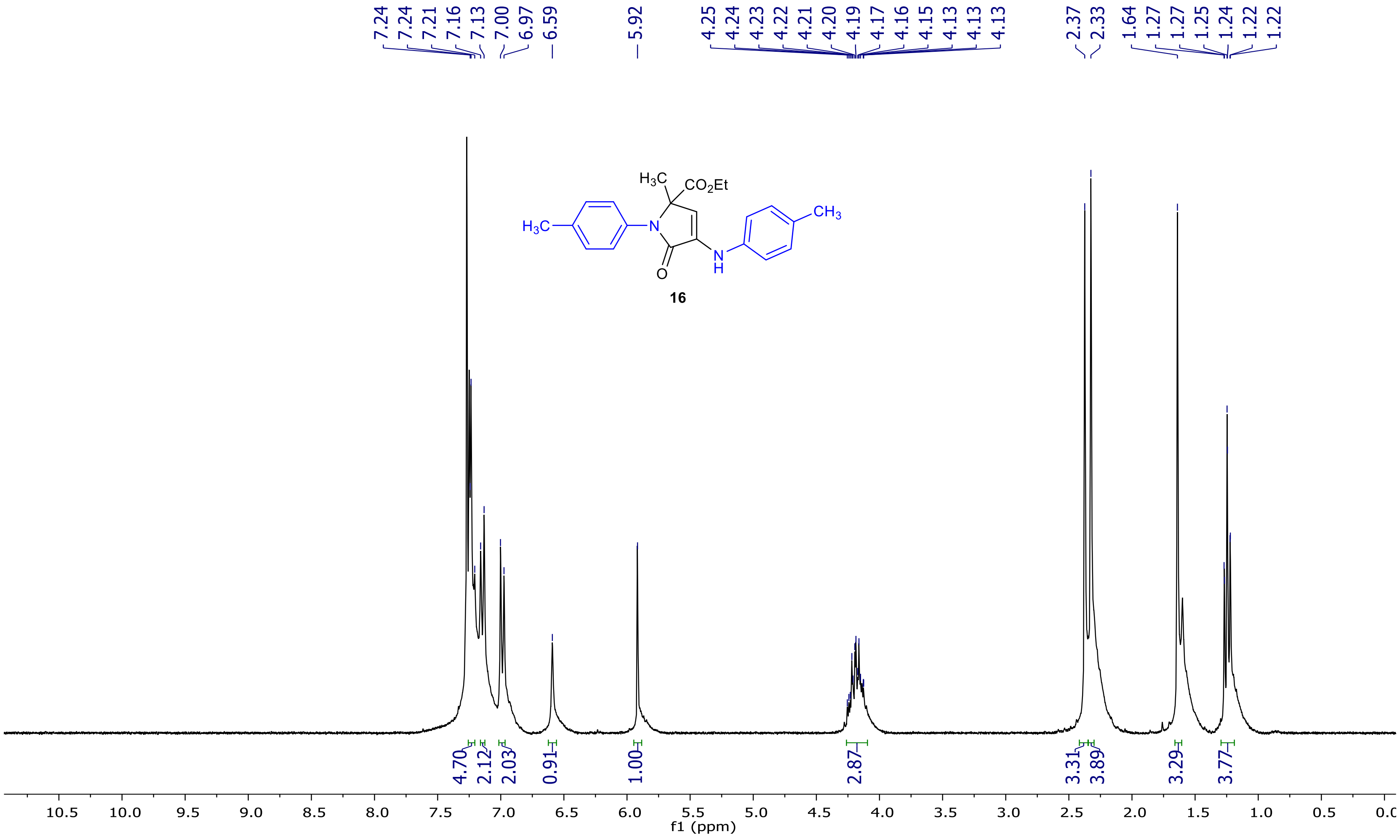

^{13}C NMR (75 MHz, $CDCl_3$)

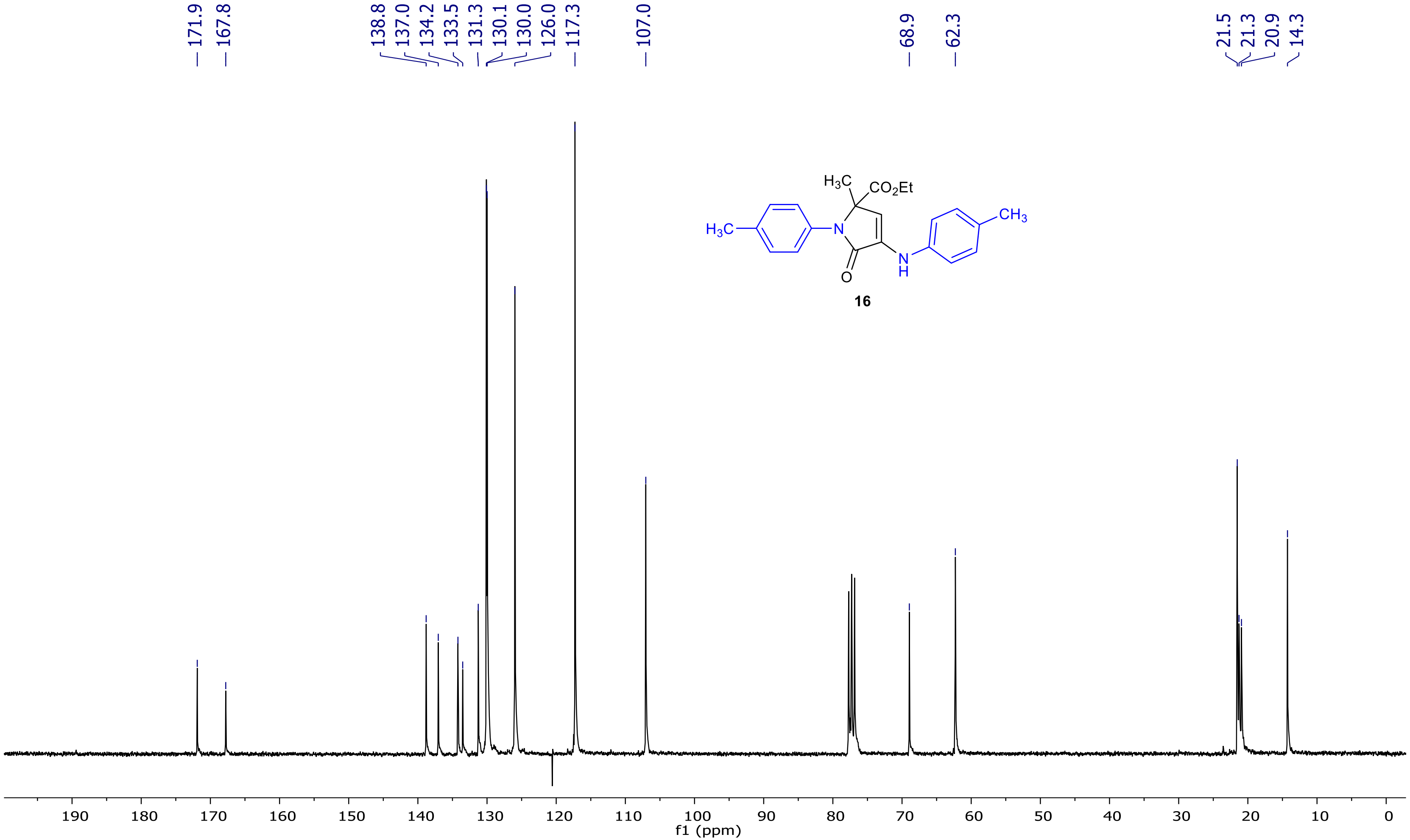

Ethyl (2S, 4R*)-2-methyl-5-oxo-1-(p-tolyl)-4-(p-tolylamino)pyrrolidine-2-carboxylate (**17**).*

¹H NMR (400 MHz, $CDCl_3$)

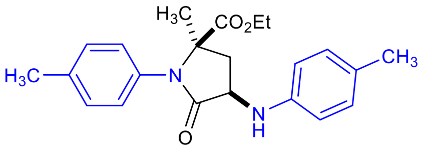

17

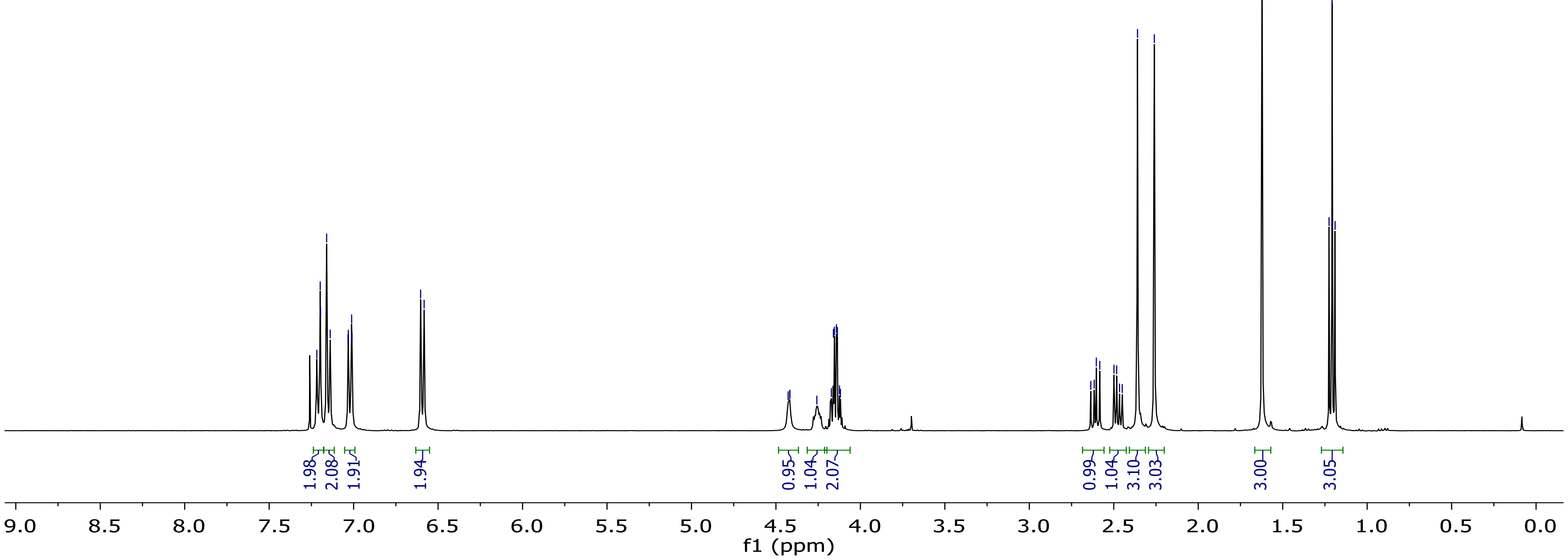

^{13}C NMR (100 MHz, $CDCl_3$)

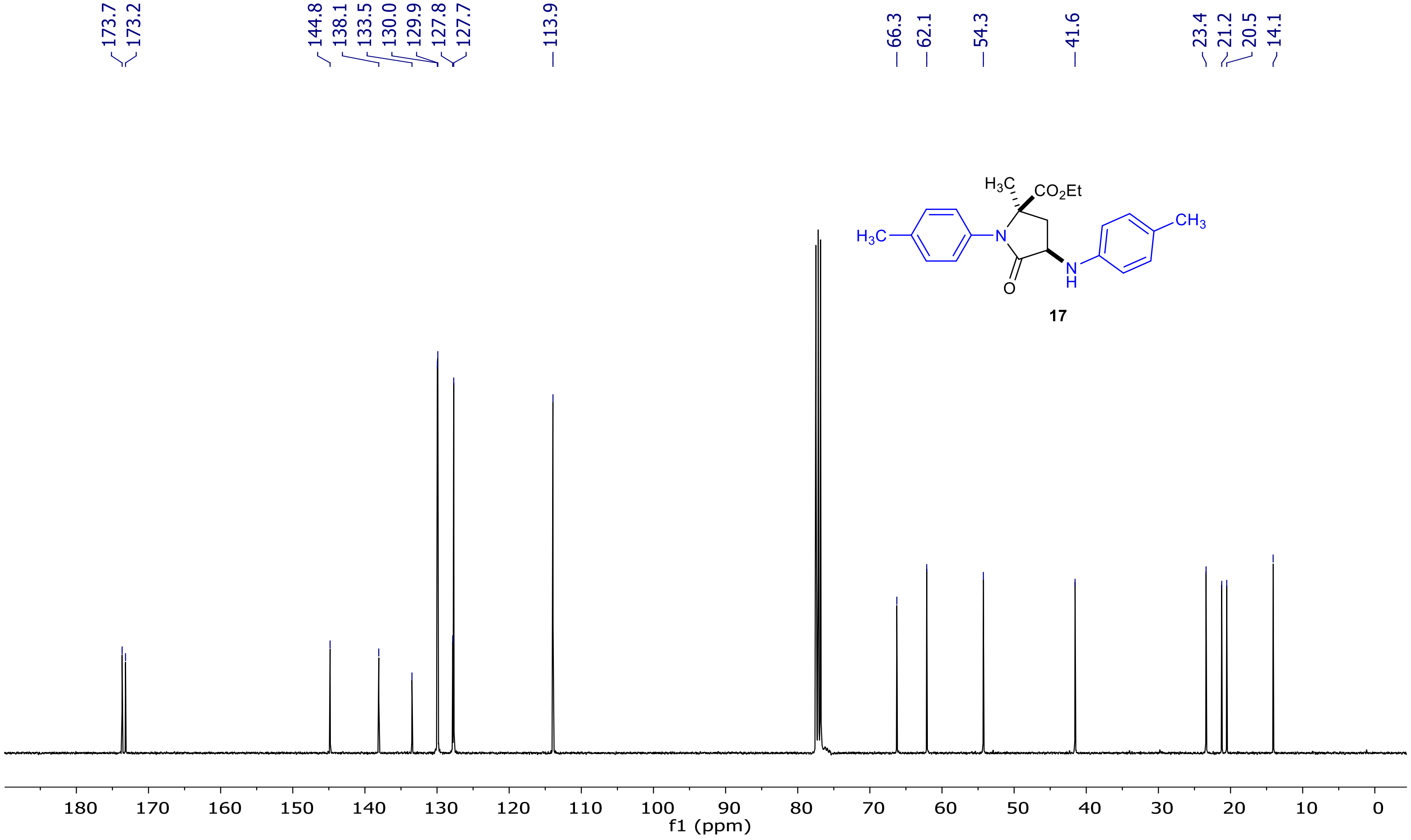

((3R, 5R*)-1-(p-Tolyl)-3-(p-tolylamino)-5-(trifluoromethyl)pyrrolidin-2-one (**18a**).*

¹H NMR (400 MHz, $CDCl_3$)

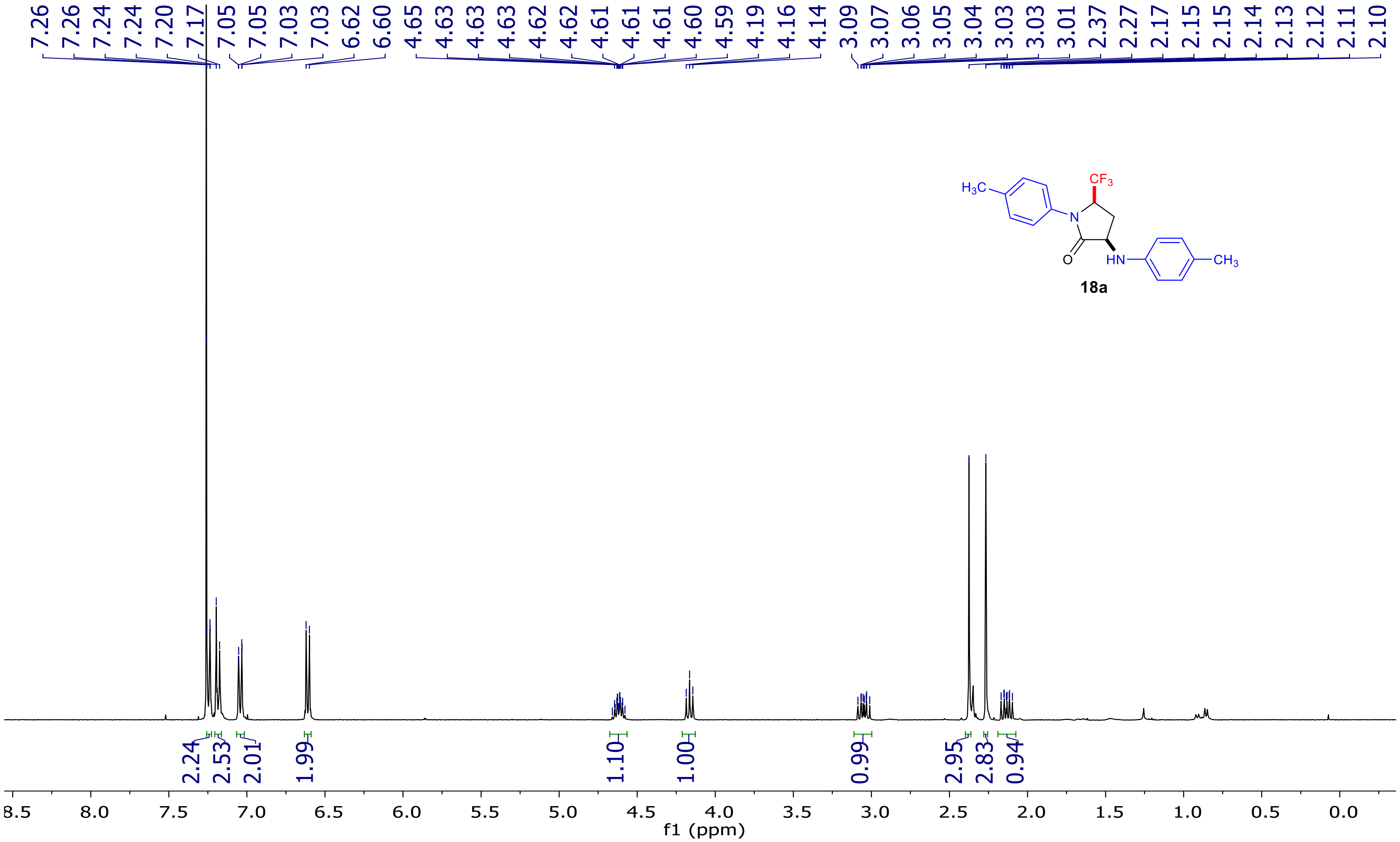

^{13}C NMR (75 MHz, CDCl3)

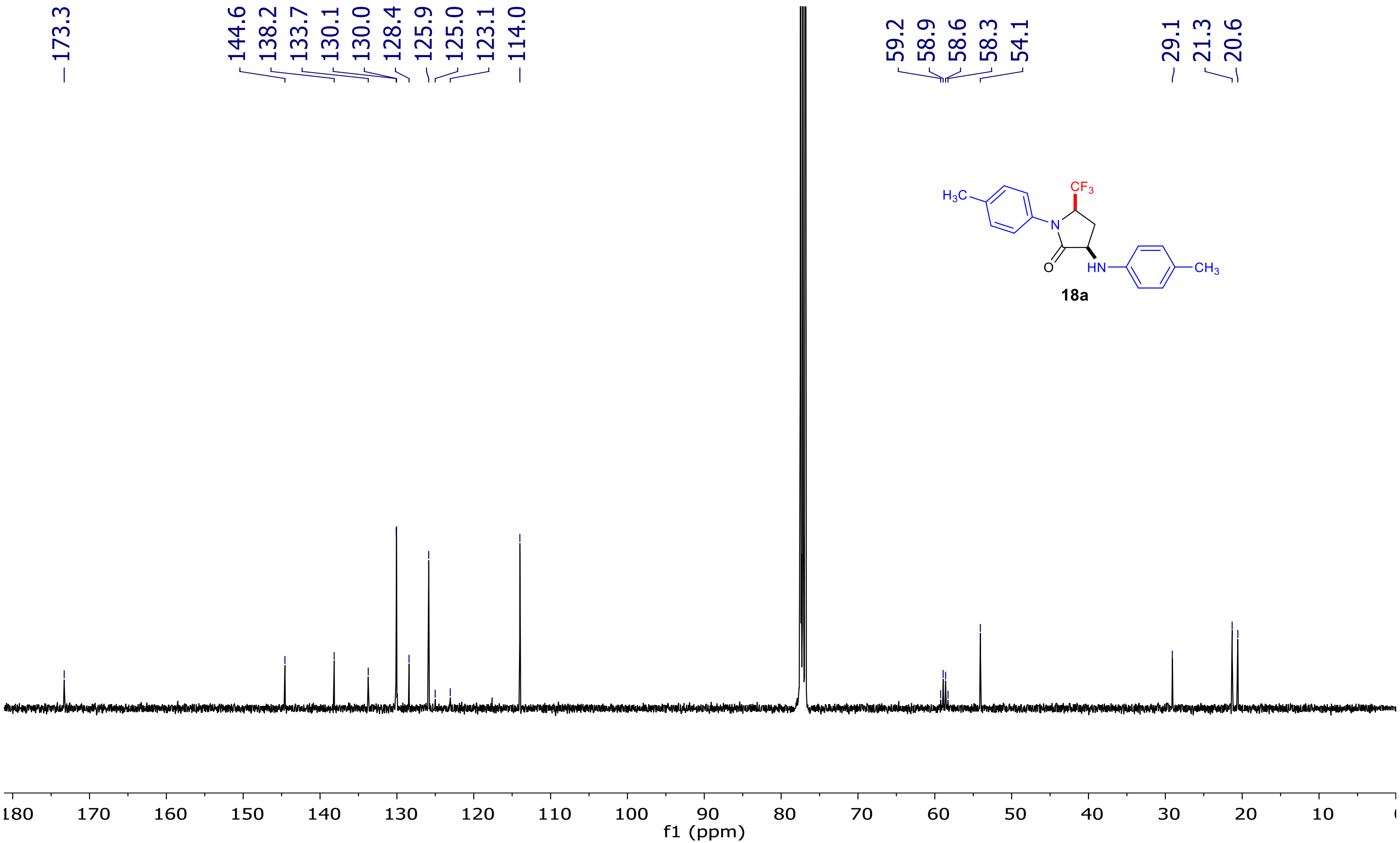

¹⁹F NMR (282 MHz, $CDCl_3$)

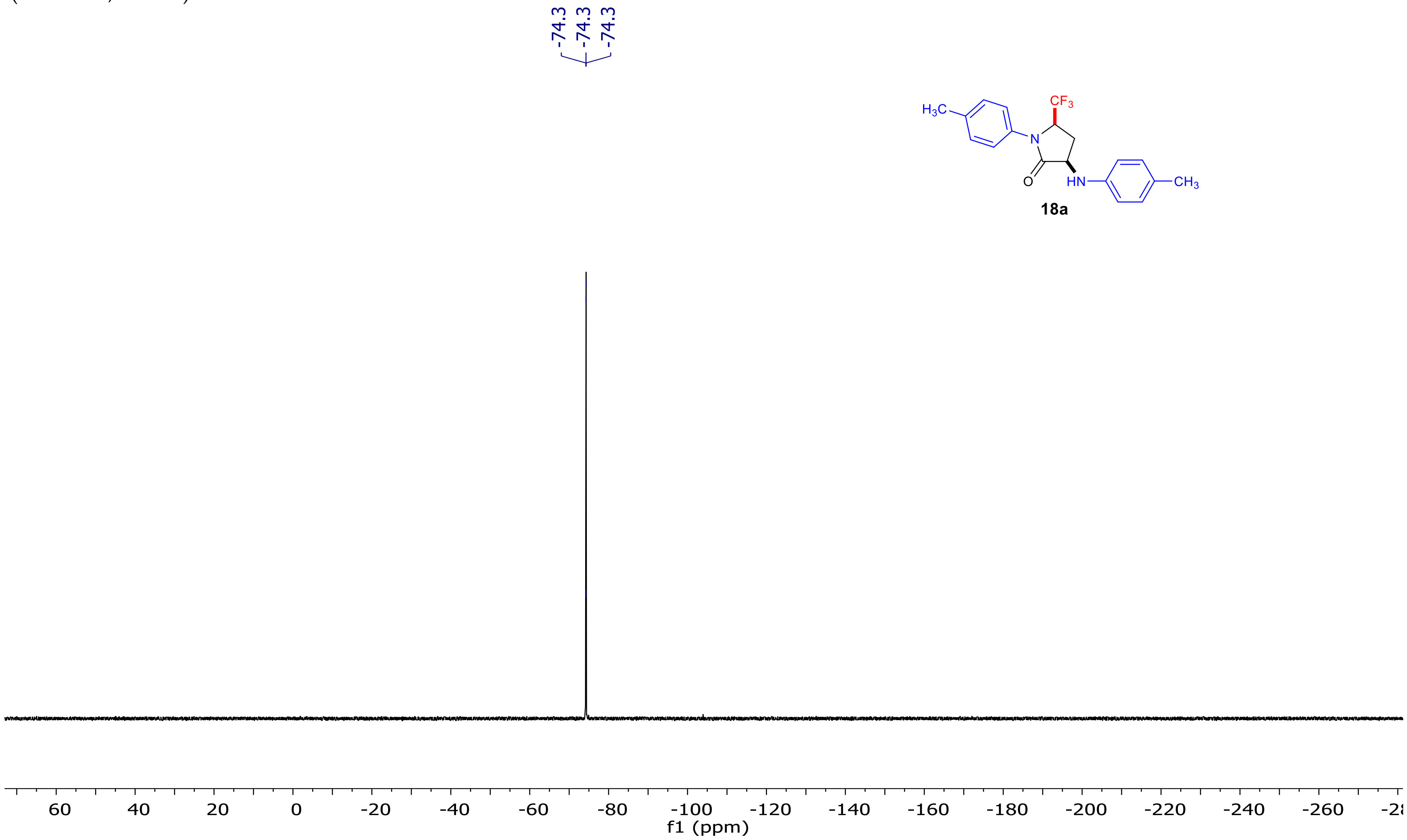

18a

Diethyl (((2R, 4R*)-5-oxo-1-(p-tolyl)-4-(p-tolylamino)pyrrolidin-2-yl)methyl)phosphonate (**18b**).*
¹H NMR (400 MHz, $CDCl_3$)

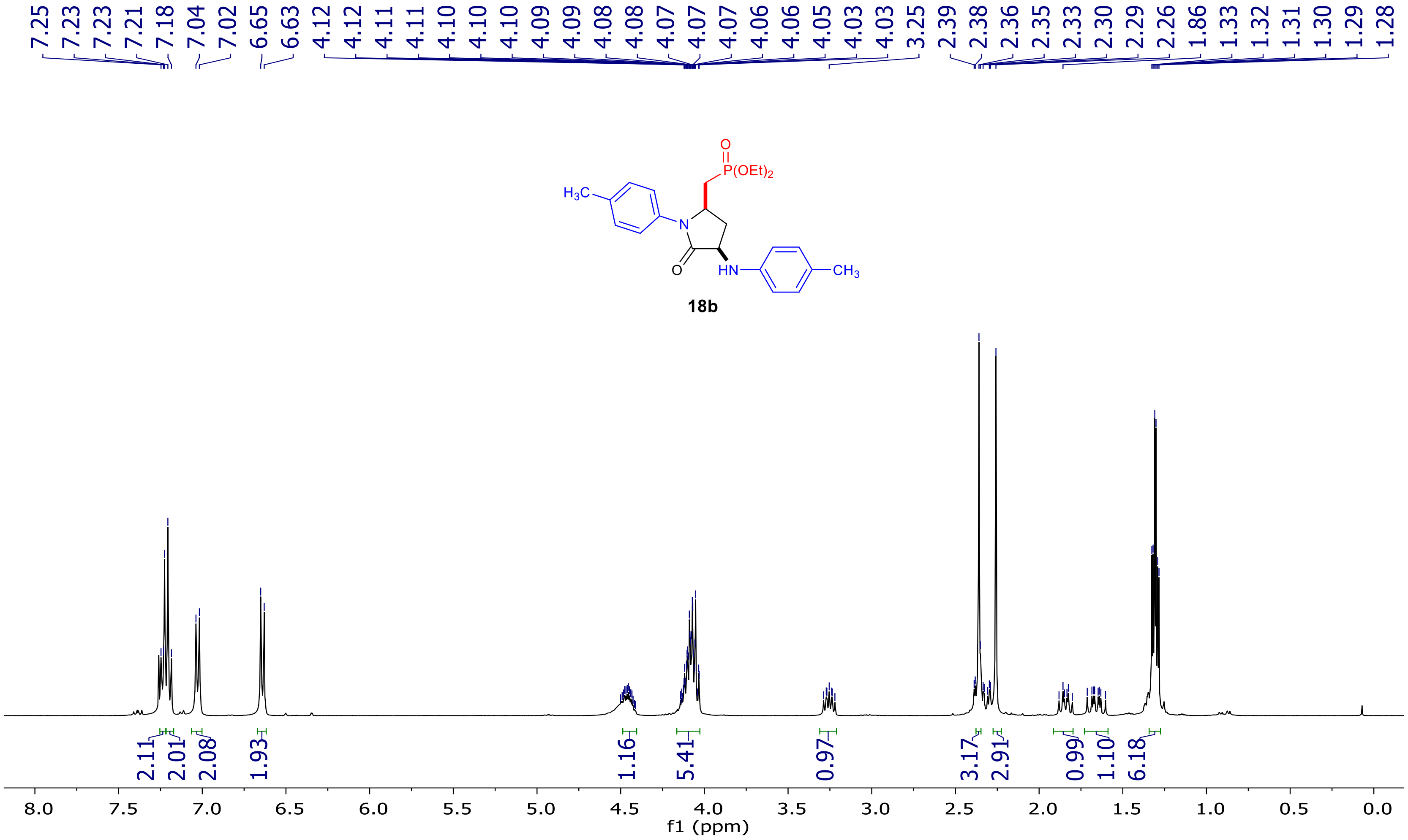

^{13}C NMR (101 MHz, $CDCl_3$)

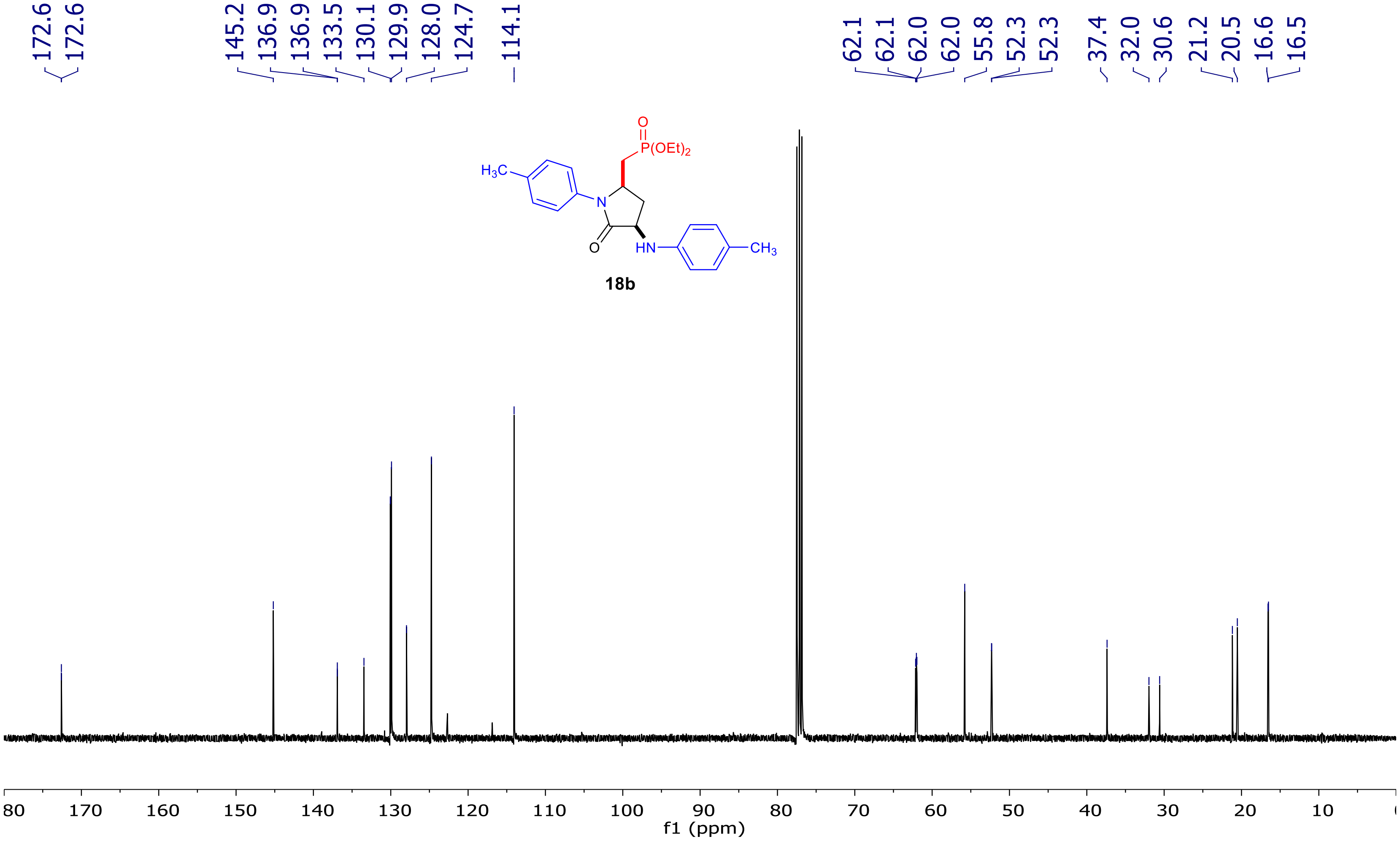

^{31}P NMR (162 MHz, $CDCl_3$)

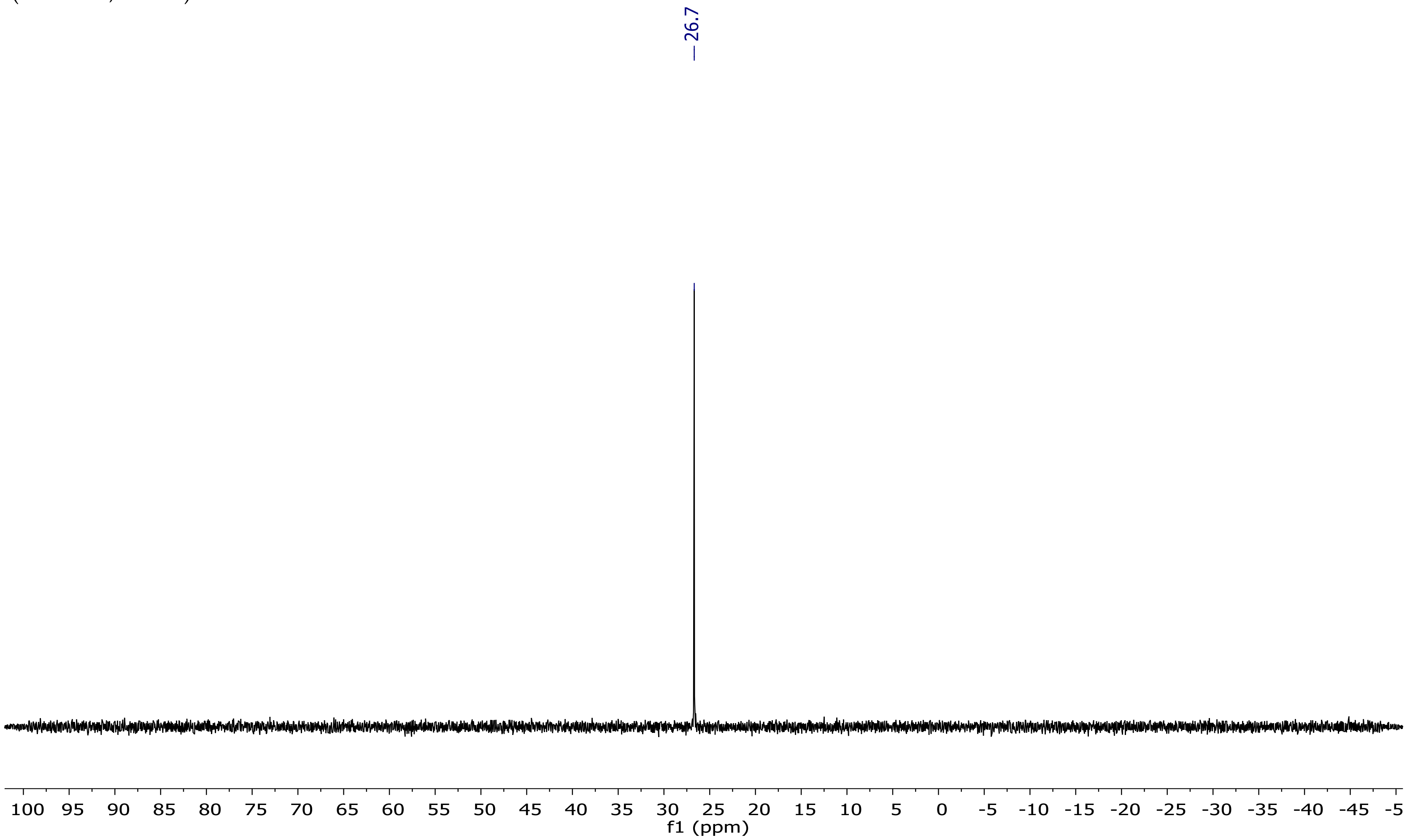

(3R, 5R*)-1-(p-tolyl)-3-((p-methoxyphenyl)amino)-5-phenylpyrrolidin-2-one (**18c**).*

¹H NMR (400 MHz, $CDCl_3$)

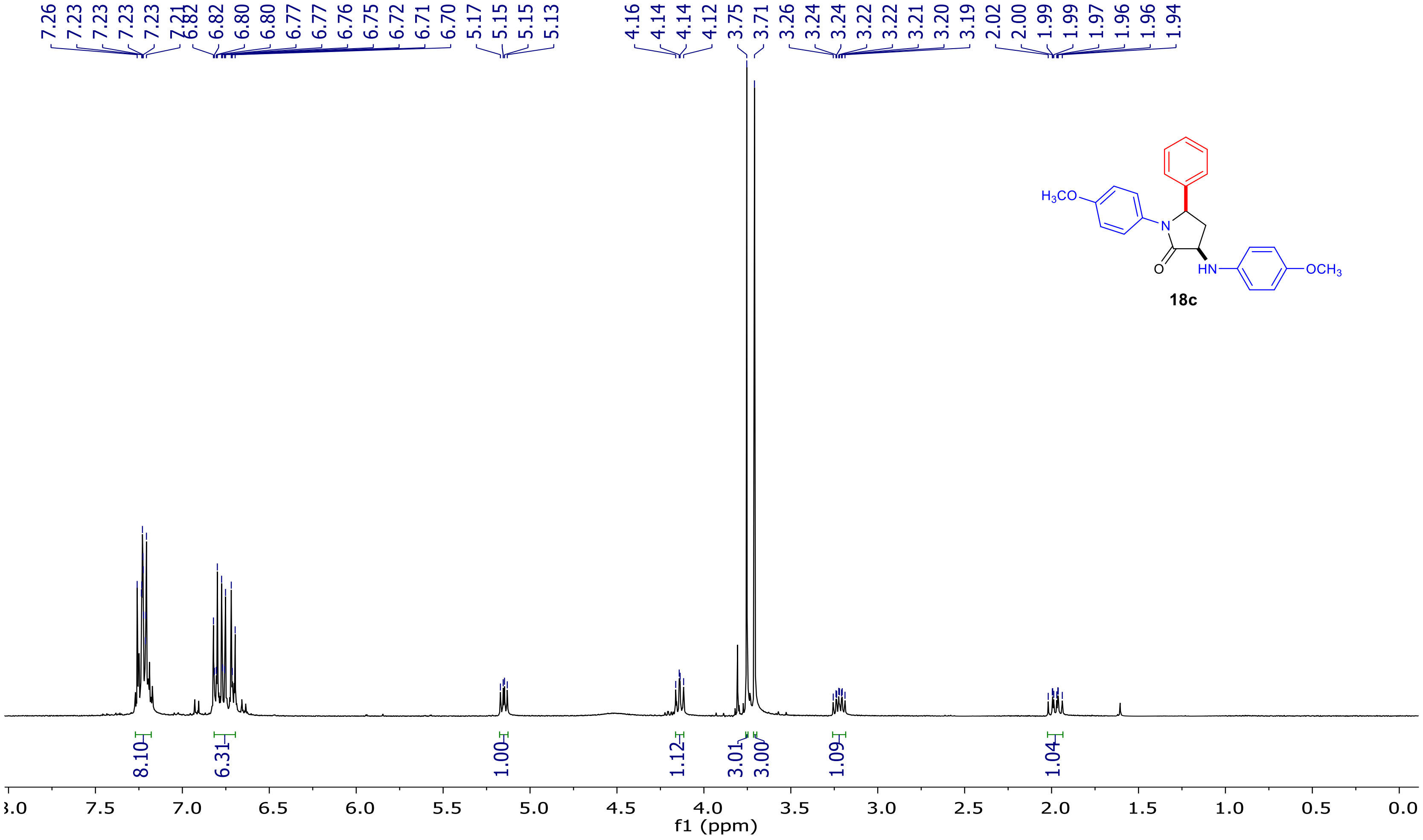

^{13}C NMR (100 MHz, $CDCl_3$)

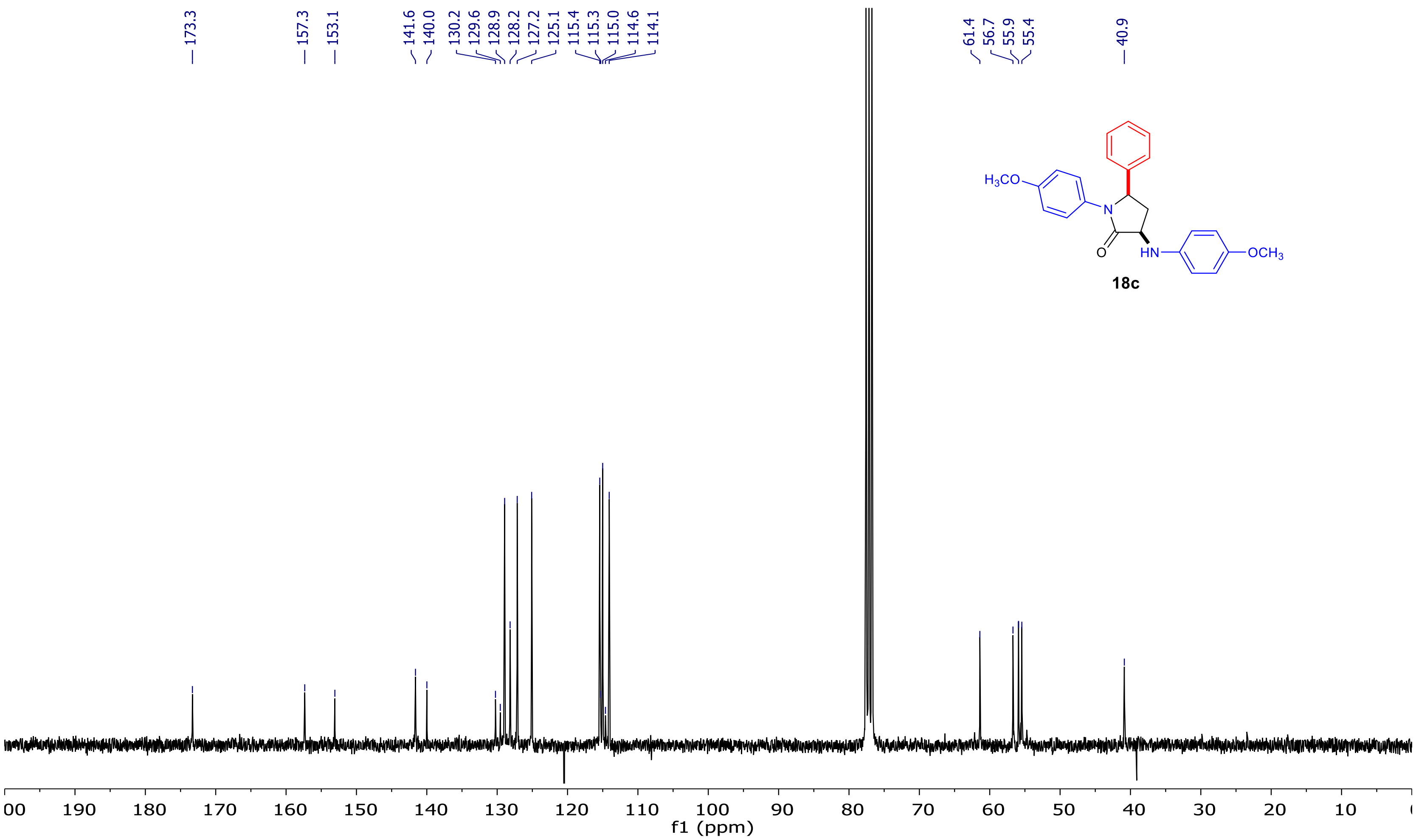

*Diethyl ((3,4-dimethyl-5-oxo-1-(p-tolyl)-4-(p-tolylamino)-4,5-dihydro-1H-pyrrol-2-yl)methyl)phosphonate (**19**).*
[1]**H NMR** (400 MHz, $CDCl_3$)

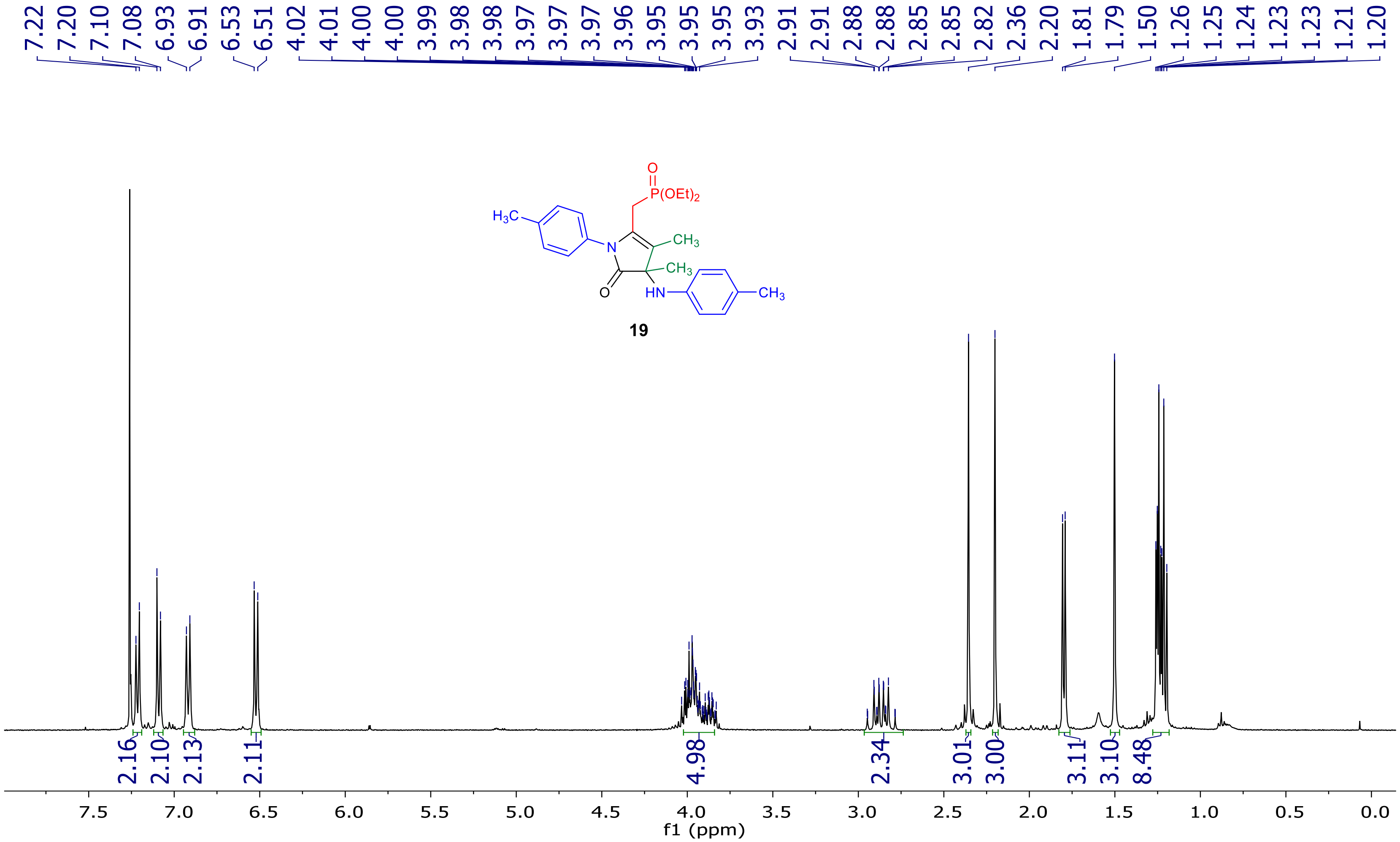

^{13}C NMR (101 MHz, $CDCl_3$)

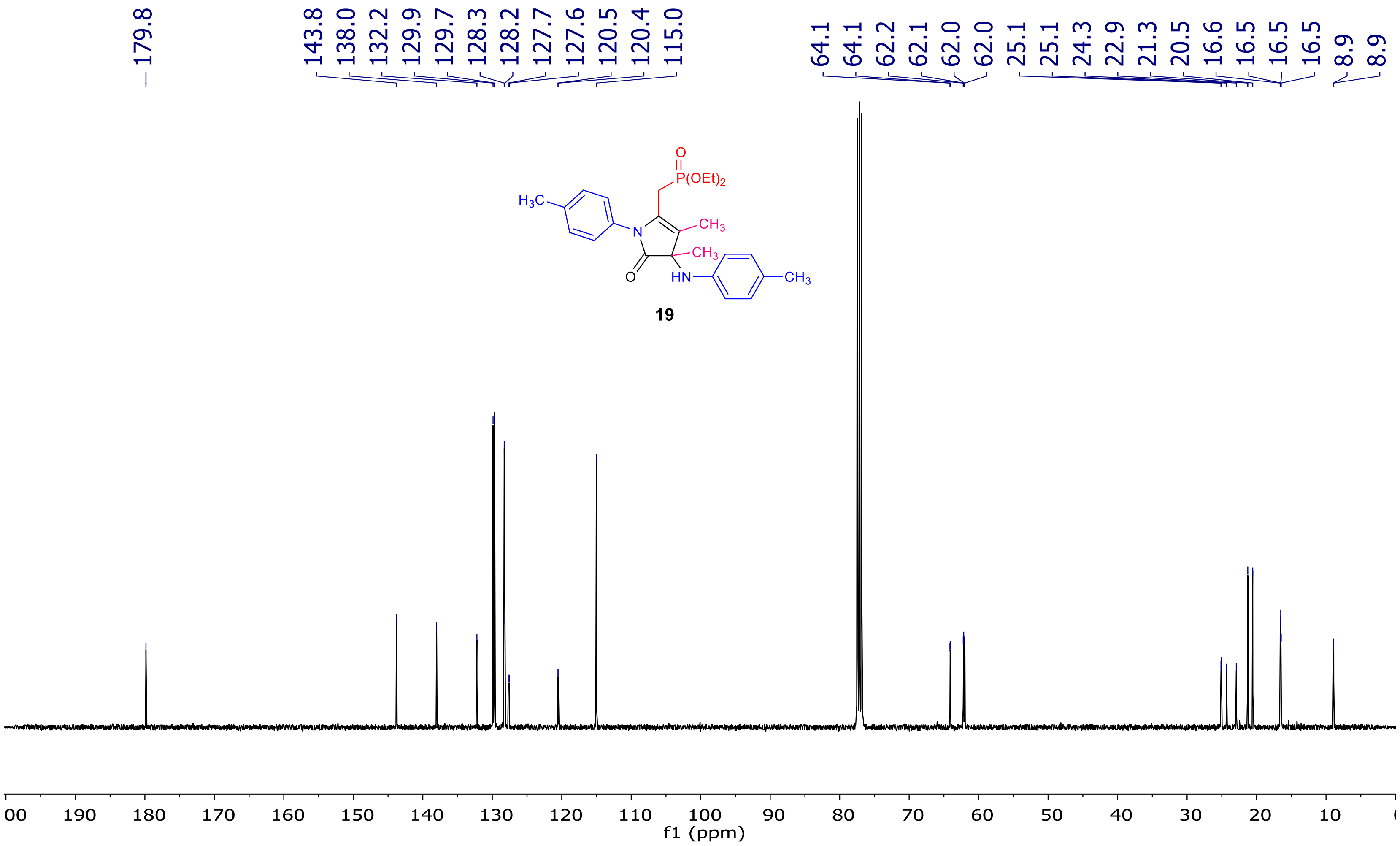

^{31}P NMR (162 MHz, $CDCl_3$)

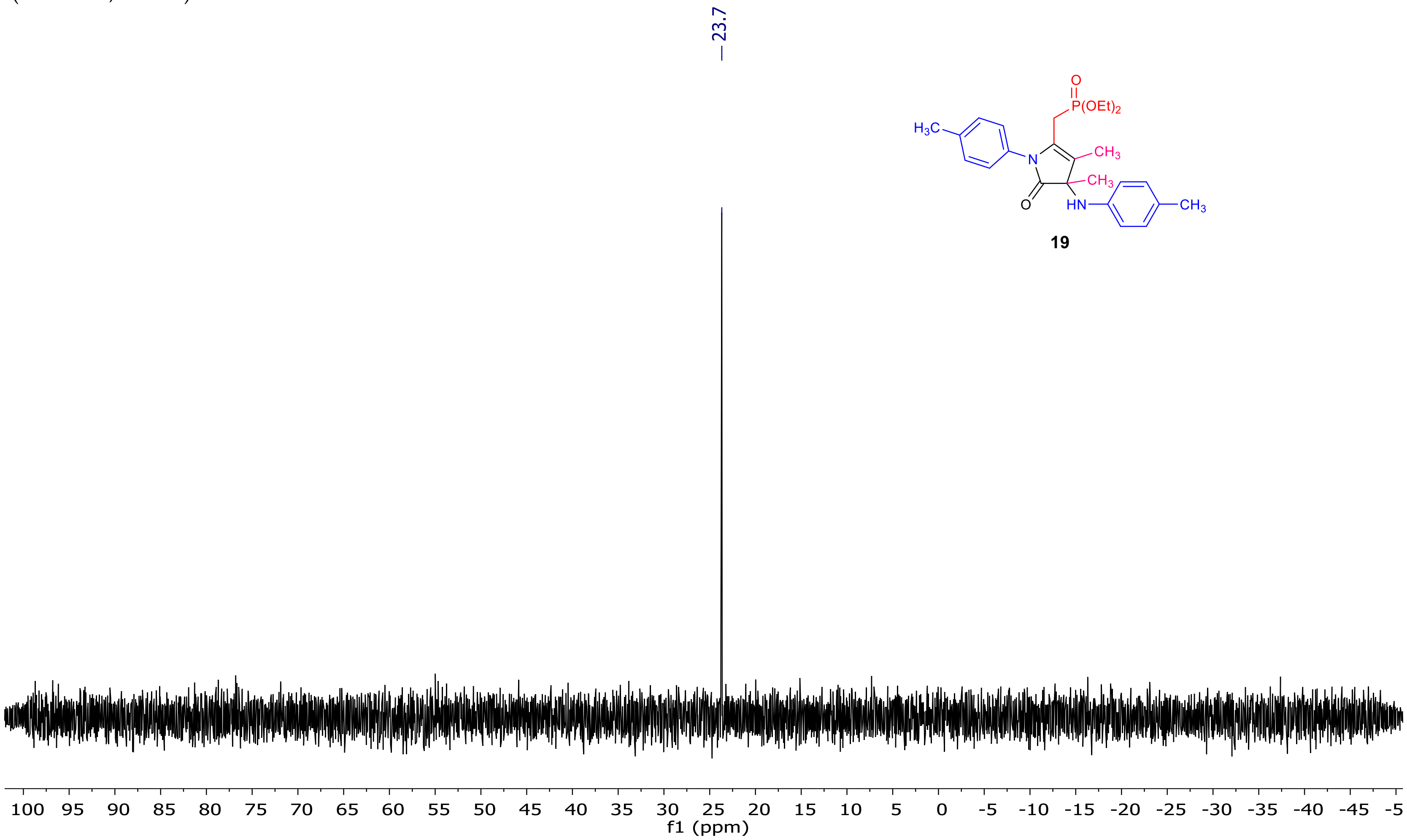

*(2Z, 3E, 5E)-6-Phenyl-N-(p-tolyl)-2-(p-tolyl imino)hexa-3,5-dienamide (**20a**).*

¹H NMR (400 MHz, $CDCl_3$)

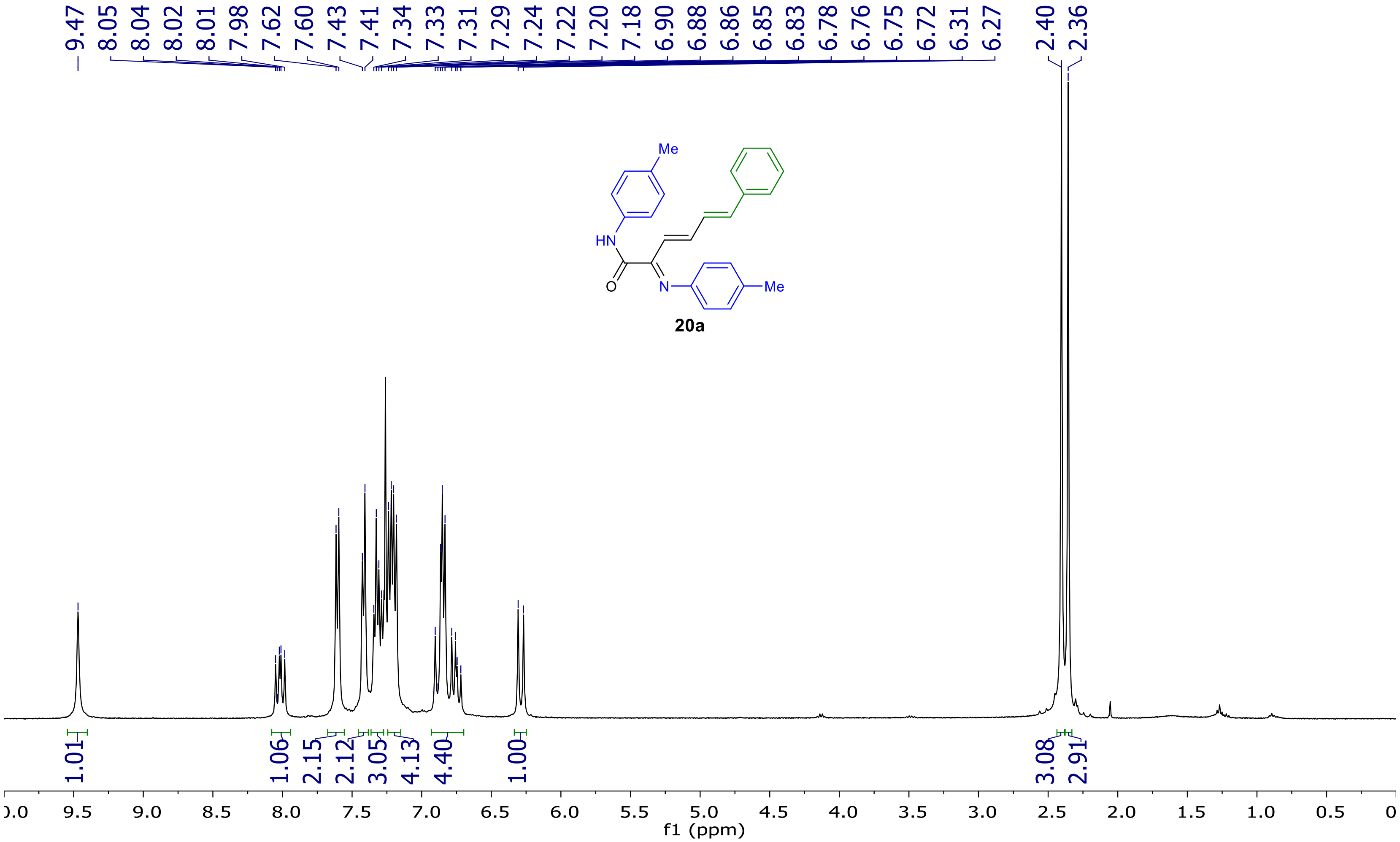

^{13}**C NMR** (101 MHz, $CDCl_3$)

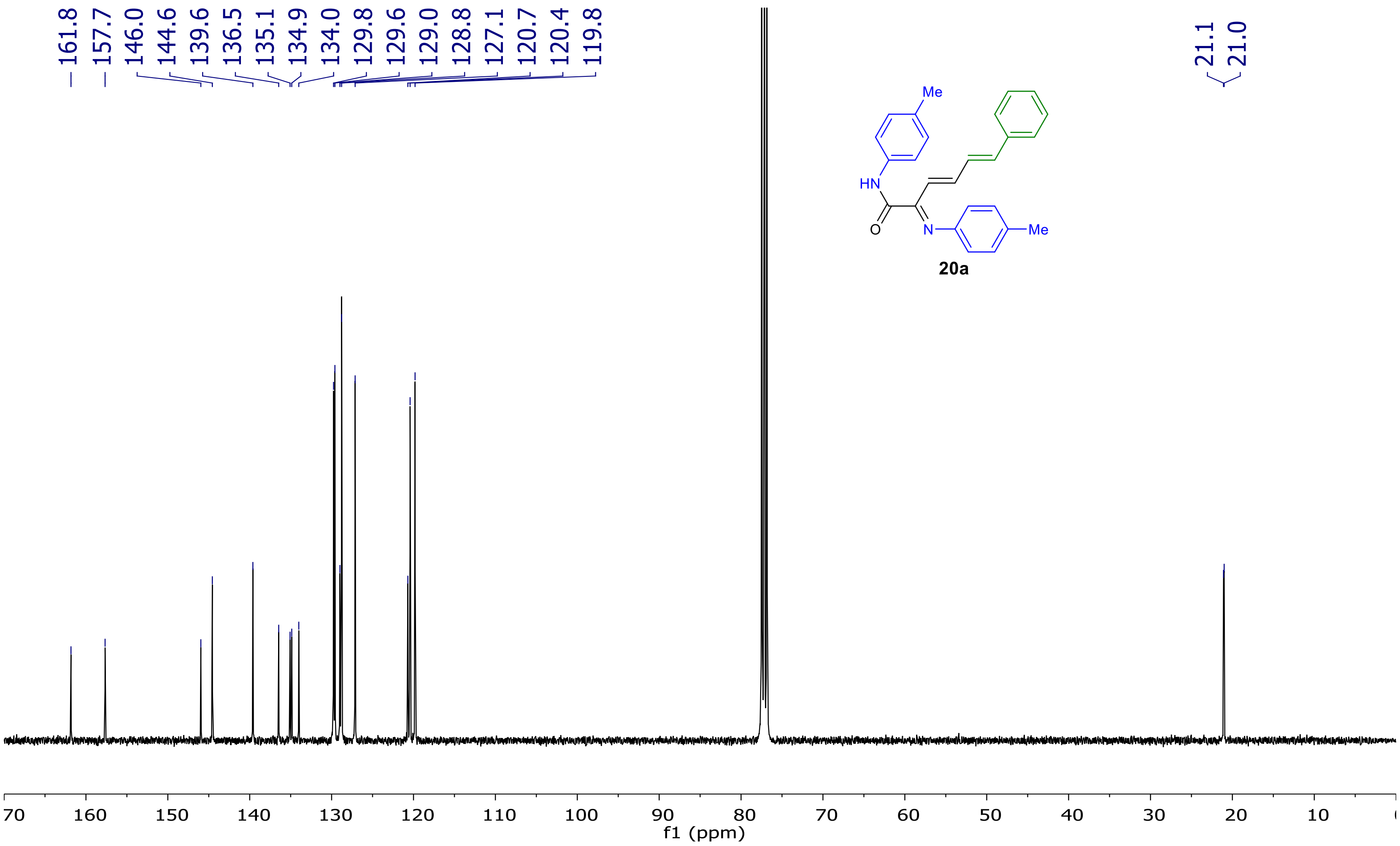

*(2Z, 3E, 5E)-N-(p-Tolyl)-2-(p-tolylimino)-6-(p-(trifluoromethyl)phenyl)hexa-3,5-dienamide (**20b**).*
^{1}H NMR (400 MHz, $CDCl_3$)

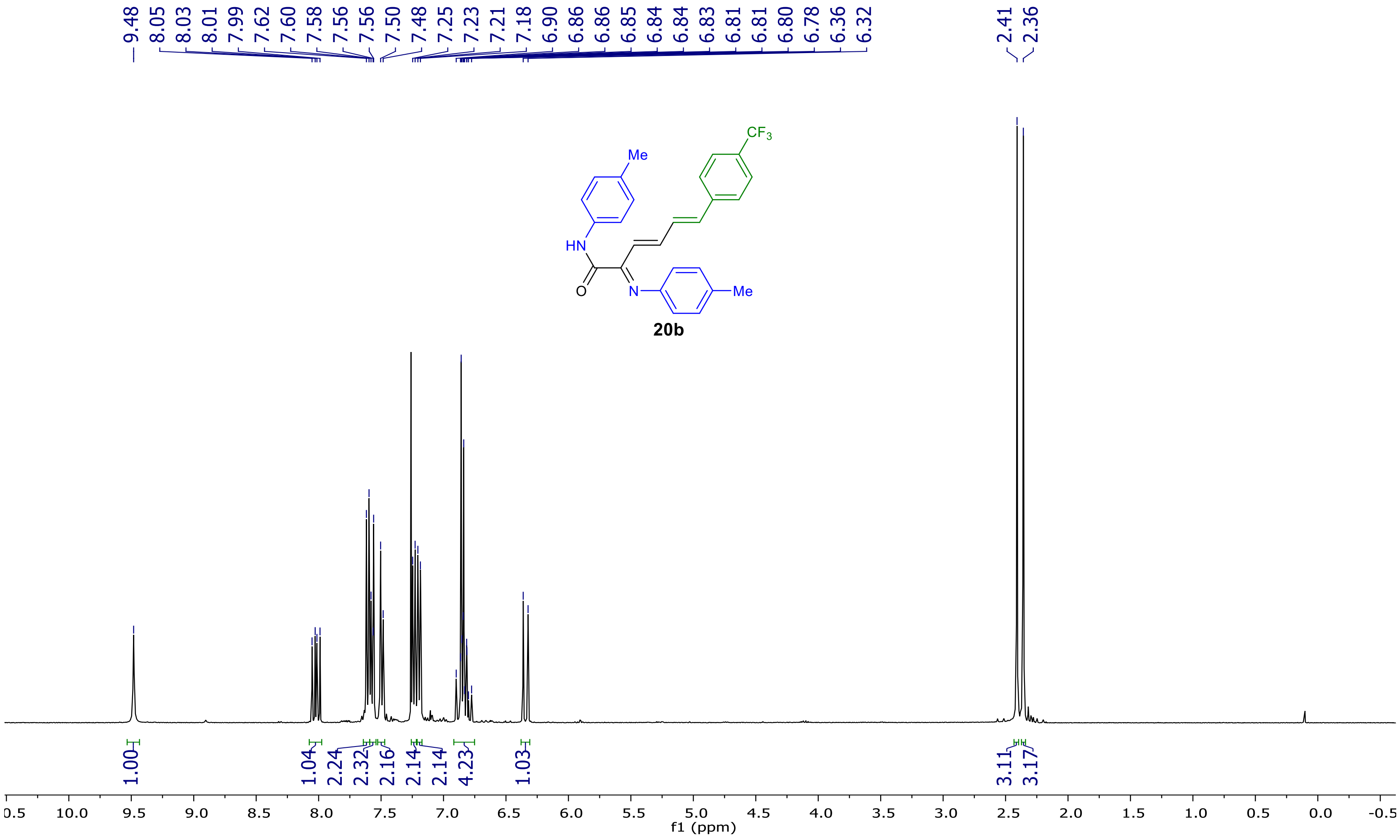

^{13}**C NMR** (101 MHz, $CDCl_3$)

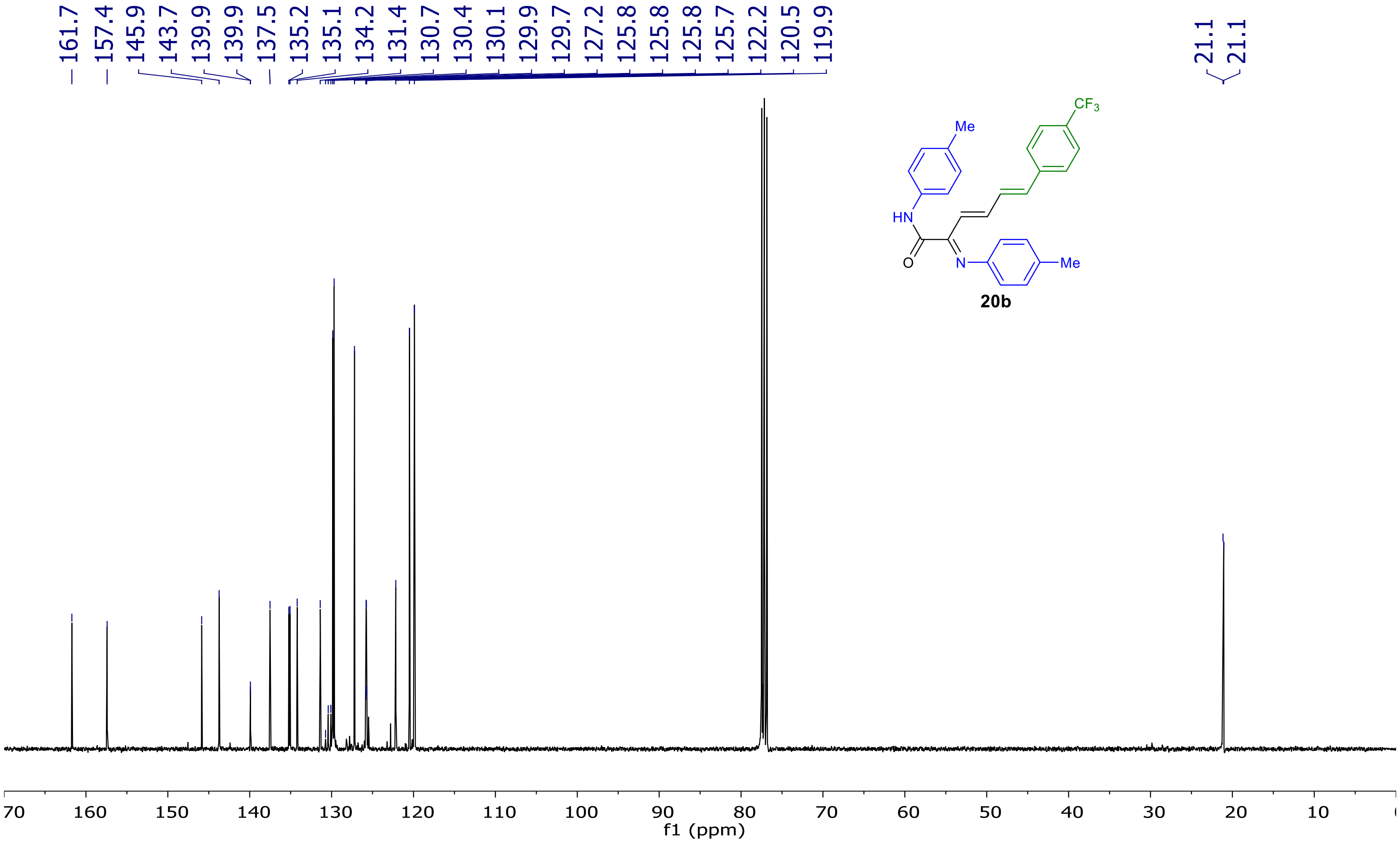

(Z)-5-Phenyl-1-(p-tolyl)-3-(p-tolylimino)-1,3-dihydro-2H-pyrrol-2-one (**21**).

^{1}H NMR (400 MHz, $CDCl_3$)

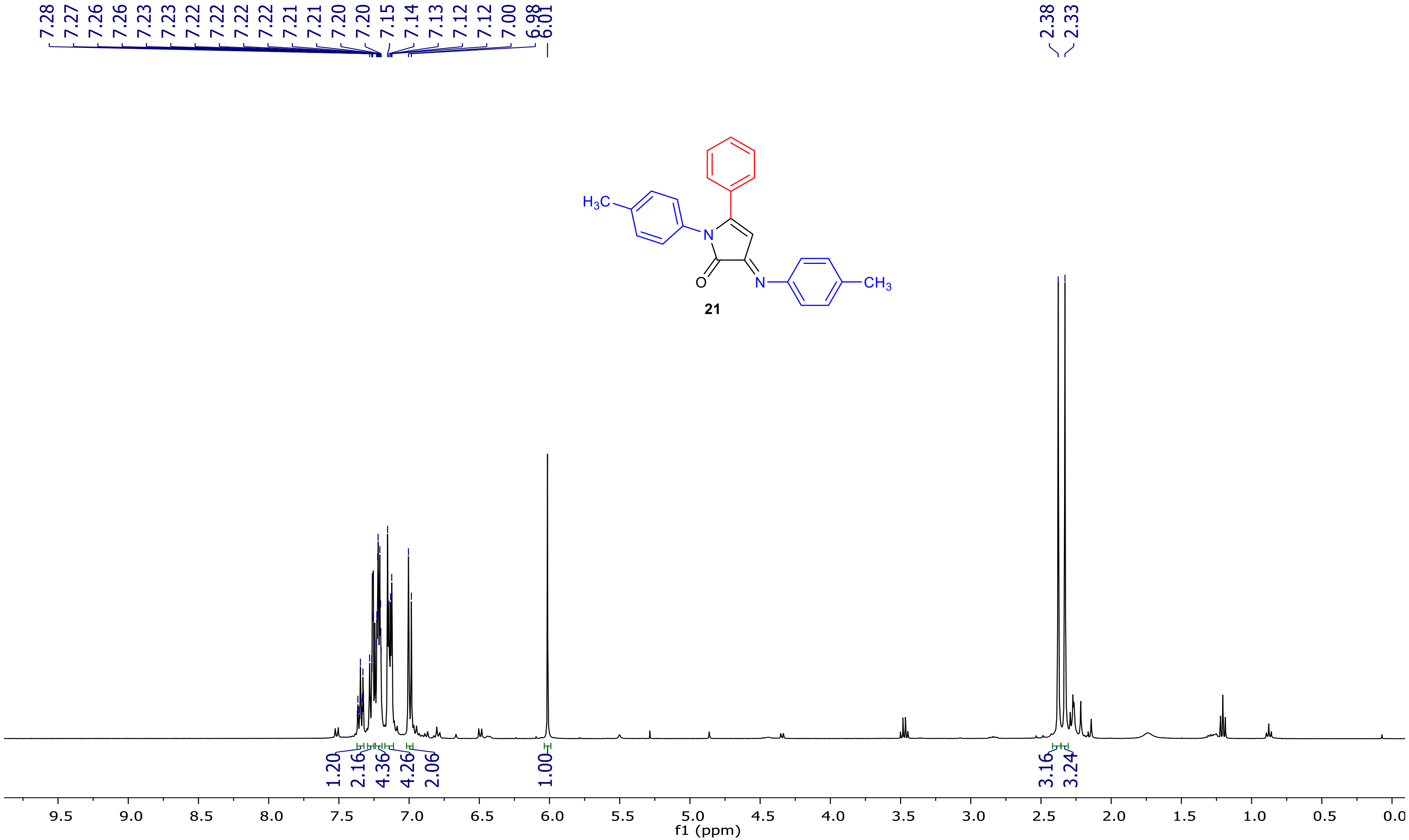

^{13}**C NMR** (101 MHz, $CDCl_3$)

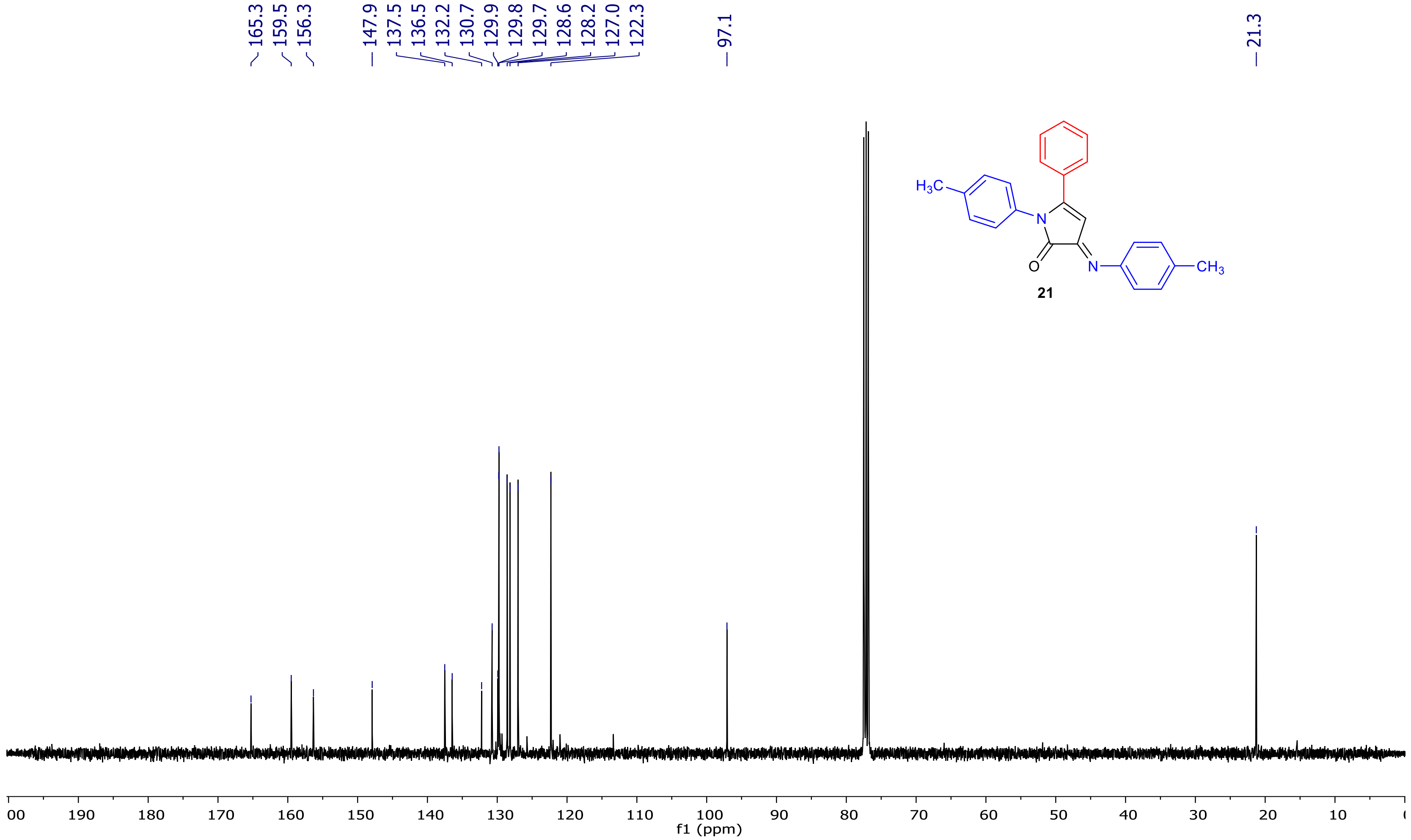

3. HPLC chromatograms of compounds 4-12 and 16-21.

1-(p-Tolyl)-3-(p-tolylamino)-1,5-dihydro-2H-pyrrol-2-one (***4a***).
Purity: **99.9%**

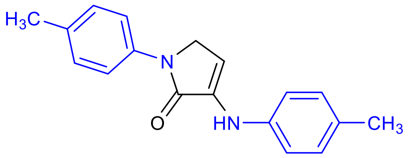

4a

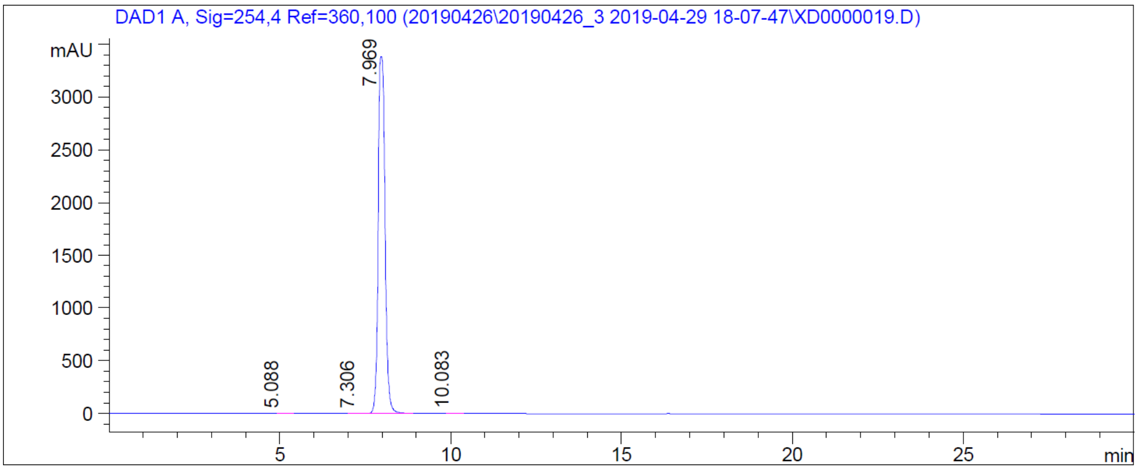

#	Meas. R	Respons	Respons	Height	Symmetr	Area %
###	5.088	16.204	0.034	0.037	0	0.034
###	7.306	11.217	0.024	0.030	2	0.024
###	7.969	4.701e4	99.902	99.887	1	99.902

5-Phenyl-1-(p-tolyl)-3-(p-tolylamino)-1H-pyrrol-2(5H)-one (***4b***).
Purity: **99.4%**

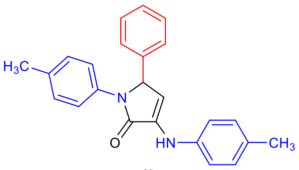

4b

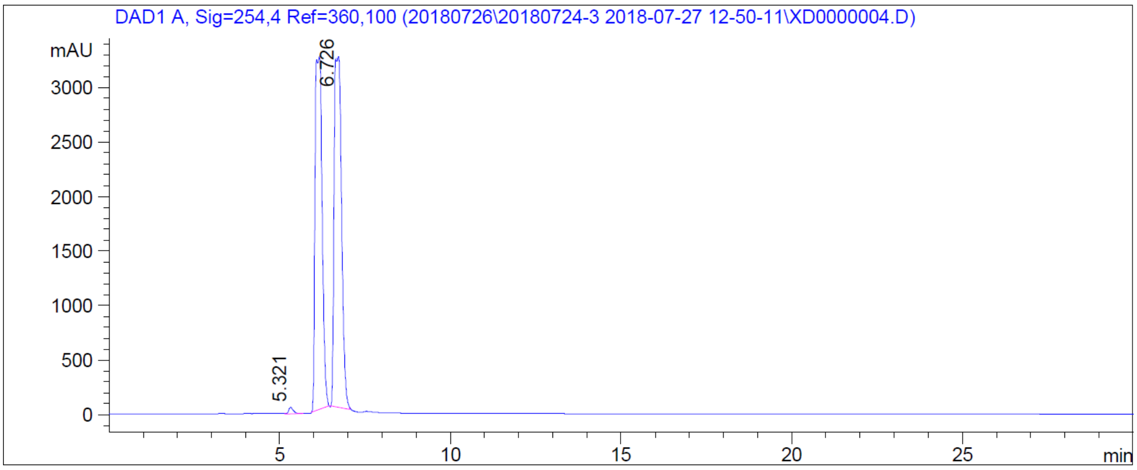

#	Meas. R	Respons	Respons	Height	Symmetr	Area %
###	5.321	554.588	0.581	0.915	1	0.581
###	6.164	4.638e4	48.565	49.706	1	48.565
###	6.726	4.856e4	50.854	49.378	1	50.854

1,5-Di-p-tolyl-3-(p-tolylamino)-1,5-dihydro-2H-pyrrol-2-one (**4c**).
Purity: **98.5%**

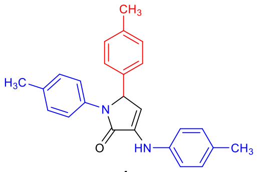

4c

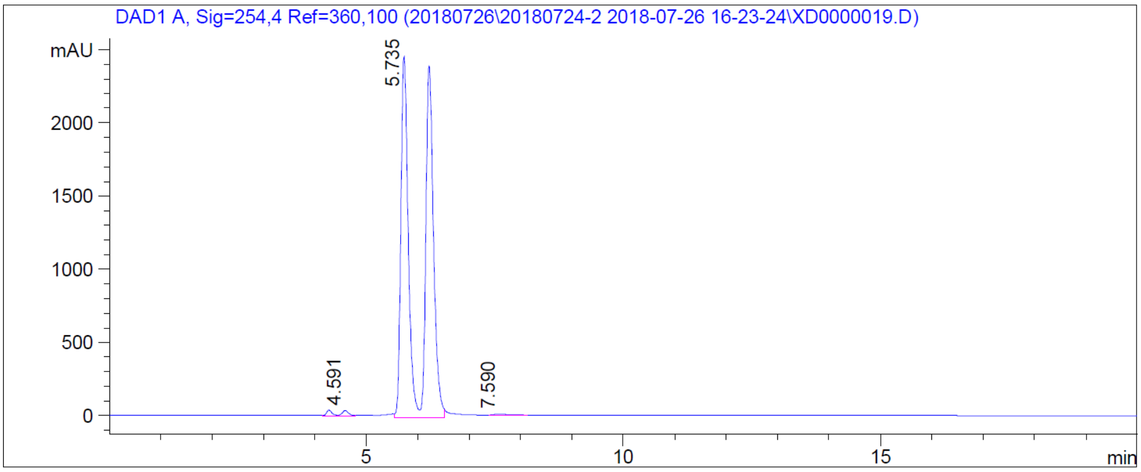

#	Meas. R	Respons	Respons	Height	Symmetr	Area %
###	4.275	307.332	0.615	1.570	1	0.615
###	4.591	317.113	0.634	1.433	1	0.634
###	5.735	4.924e4	98.476	96.690	0	98.476
###	7.590	137.674	0.275	0.307	1	0.275

5-(m-Tolyl)-1-(p-tolyl)-3-(p-tolylamino)-1,5-dihydro-2H-pyrrol-2-one (**4d**).
Purity: **98.6%**

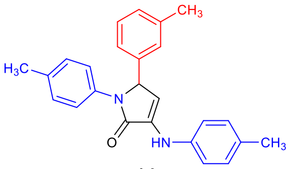

4d

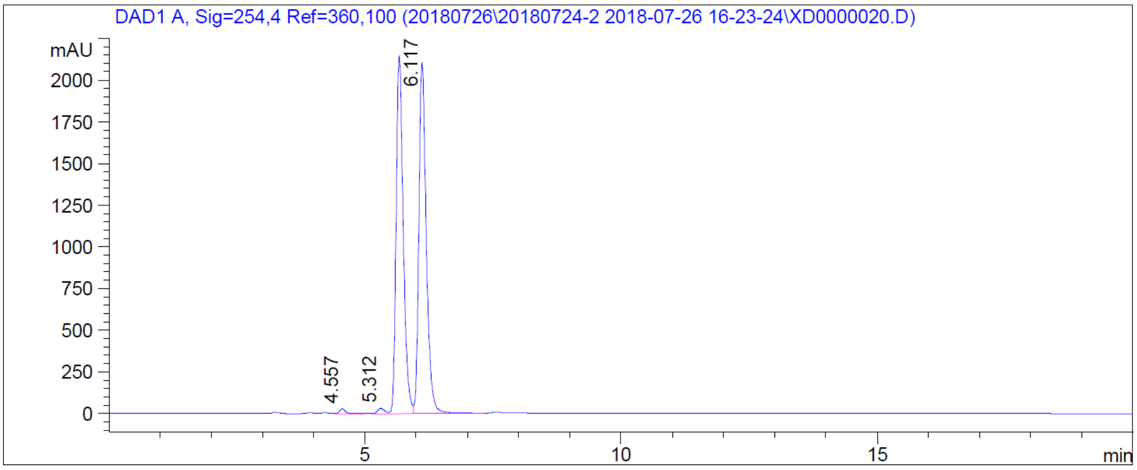

#	Meas. R	Respons	Respons	Height	Symmetr	Area %
###	4.557	282.469	0.663	0.700	1	0.663
###	5.312	320.768	0.753	0.751	1	0.753
###	5.671	2.072e4	48.608	49.713	1	48.608
###	6.117	2.130e4	49.977	48.836	1	49.977

*5-(o-Tolyl)-1-(p-tolyl)-3-(p-tolylamino)-1,5-dihydro-2H-pyrrol-2-one (**4e**).*
Purity: **98.1%**

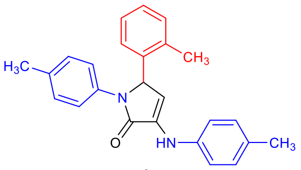

4e

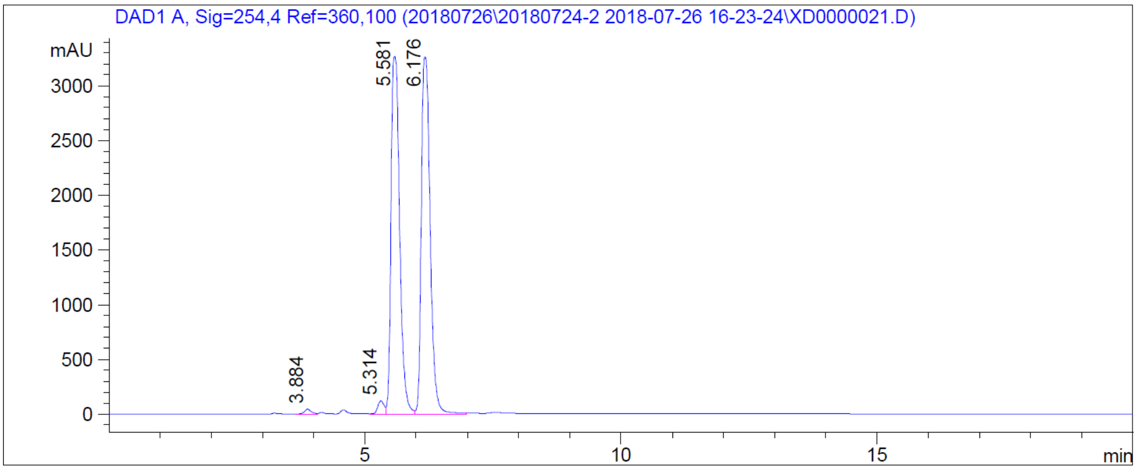

#	Meas. R	Respons	Respons	Height	Symmetr	Area %
###	3.884	438.656	0.557	0.687	1	0.557
###	5.314	1.047e3	1.329	1.802	1	1.329
###	5.581	3.821e4	48.502	48.796	1	48.502
###	6.176	3.909e4	49.613	48.715	1	49.613

*5-(p-Fluorophenyl)-1-(p-tolyl)-3-(p-tolylamino)-1,5-dihydro-2H-pyrrol-2-one (**4f**).*
Purity: **99.3%**

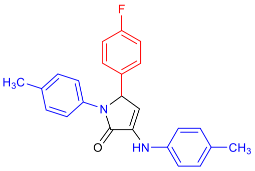

4f

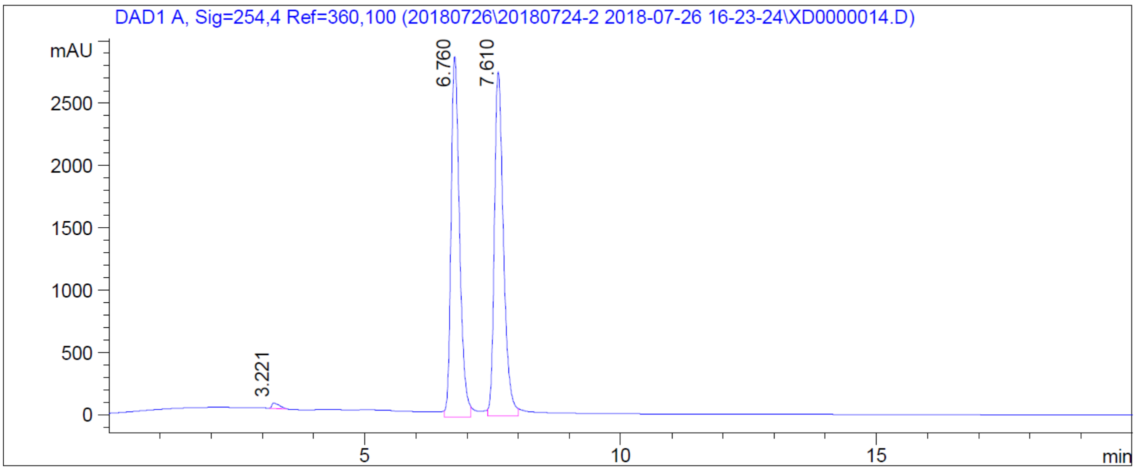

#	Meas. R	Respons	Respons	Height	Symmetr	Area %
###	3.221	412.204	0.606	0.727	0	0.606
###	6.760	3.350e4	49.217	50.772	1	49.217
###	7.610	3.415e4	50.178	48.501	1	50.178

5-(p-Nitrophenyl)-1-(p-tolyl)-3-(p-tolylamino)-1H-pyrrol-2(5H)-one (**4g**).
Purity: **99.0%**

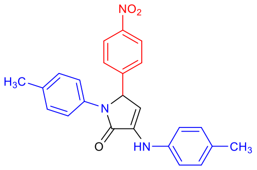

4g

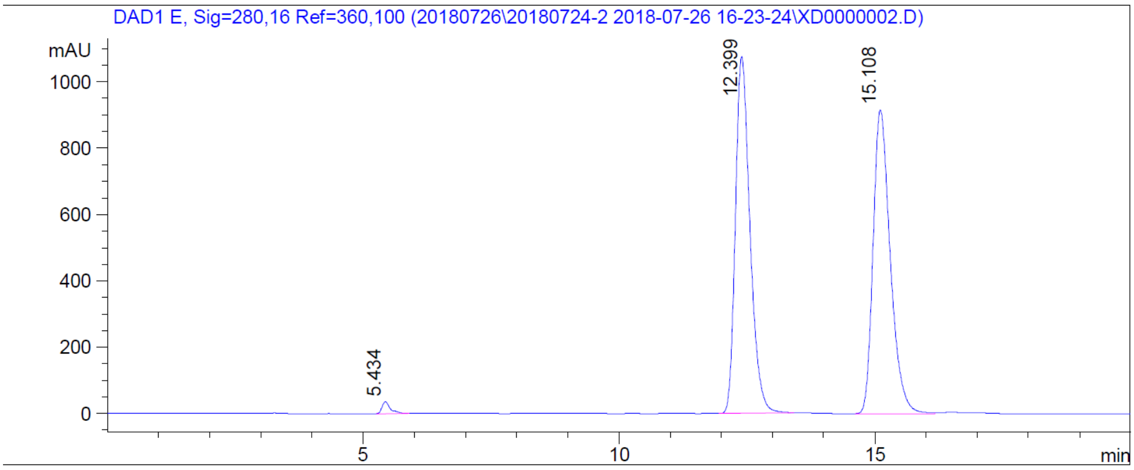

#	Meas. R	Respons	Respons	Height	Symmetr	Area %
###	5.434	407.962	0.946	1.775	1	0.946
###	12.399	2.131e4	49.403	53.062	1	49.403
###	15.108	2.142e4	49.651	45.162	1	49.651

5-(m-Nitrophenyl)-1-(p-tolyl)-3-(p-tolylamino)-1,5-dihydro-2H-pyrrol-2-one (**4h**).
Purity: **99.1%**

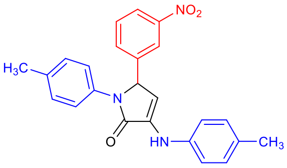

4h

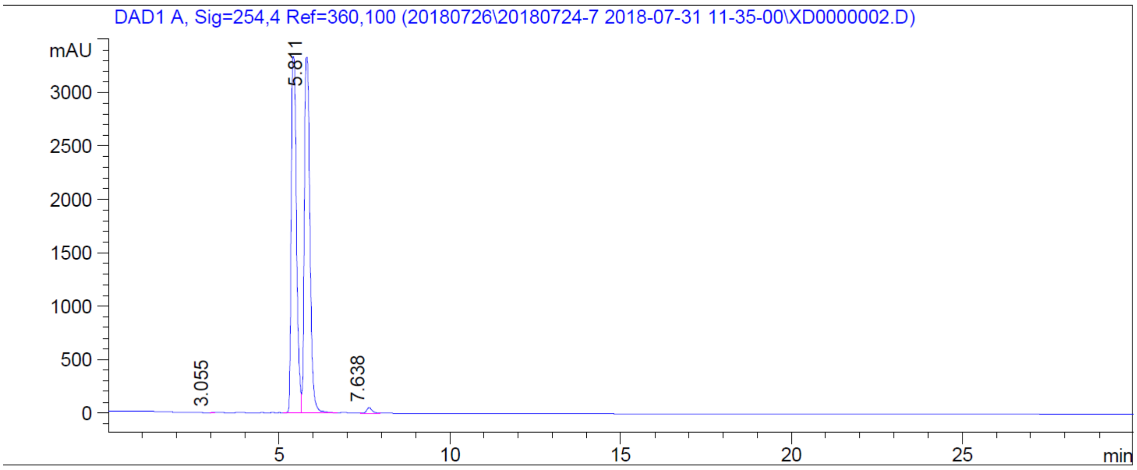

#	Meas. R	Respons	Respons	Height	Symmetr	Area %
###	3.055	9.401	0.013	0.028	1	0.013
###	5.423	3.577e4	48.526	49.614	1	48.526
###	5.811	3.732e4	50.632	49.557	1	50.632
###	7.638	611.548	0.830	0.801	1	0.830

1-(p-Tolyl)-3-(p-tolylamino)-5-(p-(trifluoromethyl)phenyl)-1,5-dihydro-2H-pyrrol-2-one (***4i***).
Purity: **97.4%**

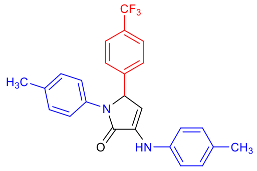

4i

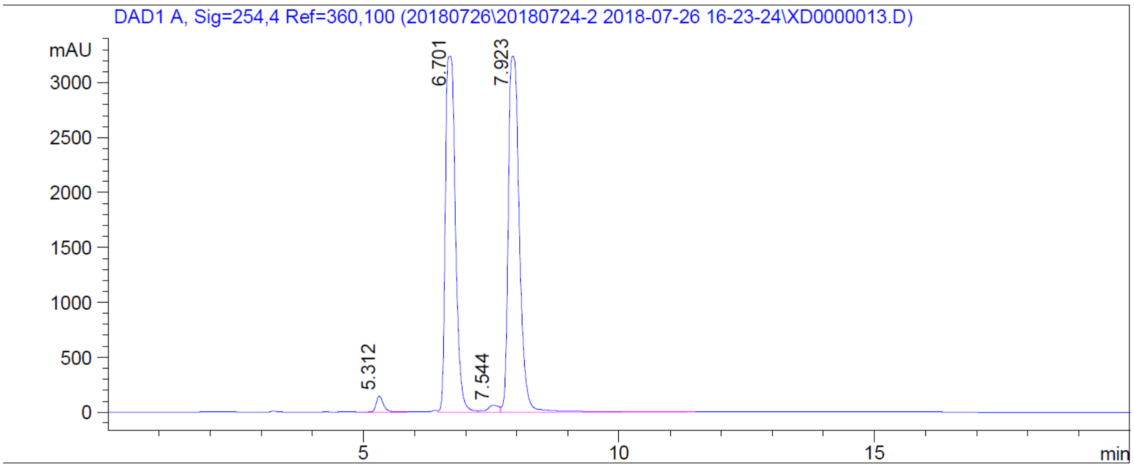

#	Meas. R	Respons	Respons	Height	Symmetr	Area %
###	5.312	1.538e3	1.606	2.196	1	1.606
###	6.701	4.452e4	46.461	48.412	1	46.461
###	7.544	975.539	1.018	0.960	1	1.018
###	7.923	4.878e4	50.915	48.431	1	50.915

5-(1-Methyl-1H-indol-6-yl)-1-(p-tolyl)-3-(p-tolylamino)-1,5-dihydro-2H-pyrrol-2-one (***4j***).
Purity: **98.9%**

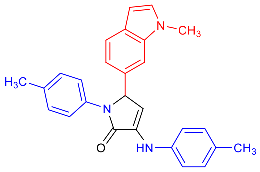

4j

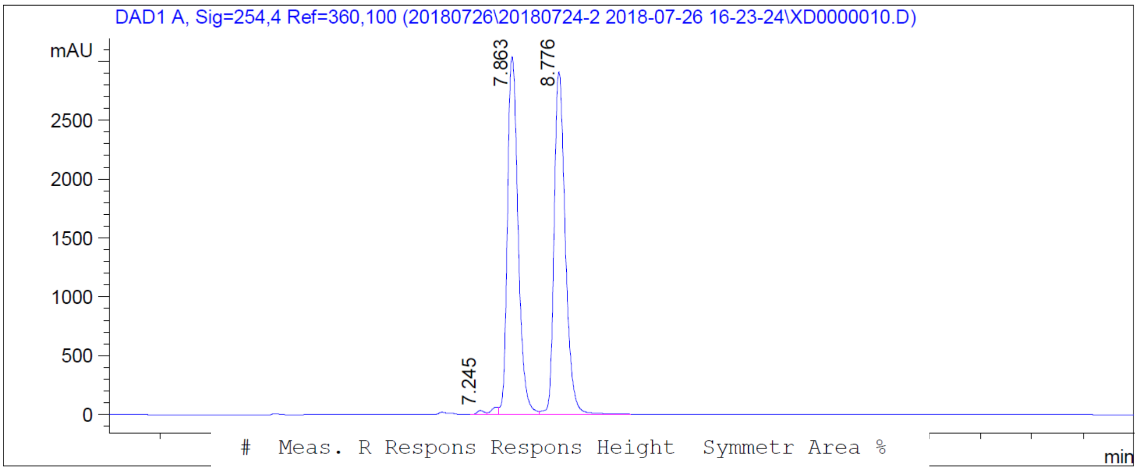

#	Meas. R	Respons	Respons	Height	Symmetr	Area %
###	7.245	339.474	0.382	0.568	1	0.382
###	7.552	577.522	0.649	1.030	2	0.649
###	7.863	4.380e4	49.225	50.278	1	49.225
###	8.776	4.426e4	49.744	48.125	1	49.744

5-(Furan-2-yl)-1-(p-tolyl)-3-(p-tolylamino)-1,5-dihydro-2H-pyrrol-2-one (**4k**).
Purity: **99.8%**

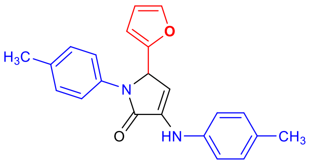

4k

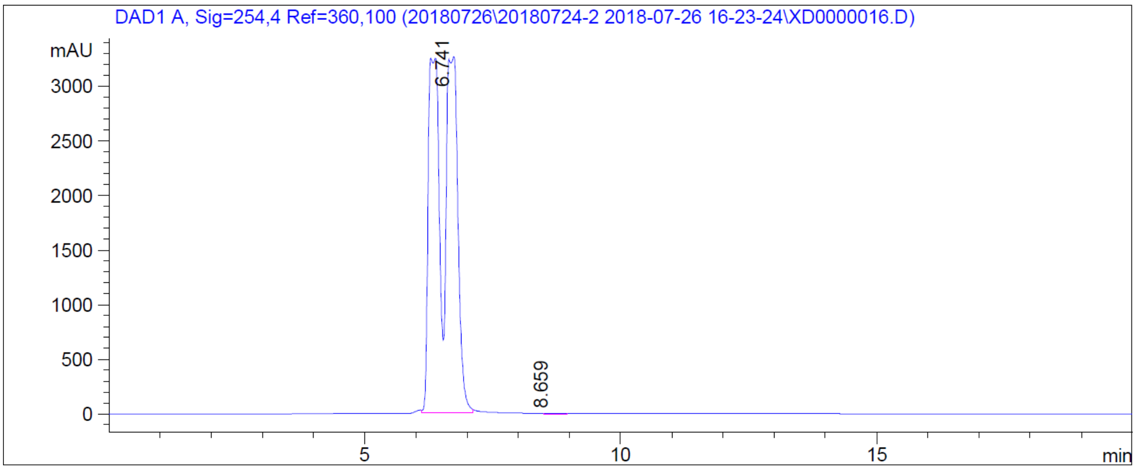

#	Meas. R	Respons	Respons	Height	Symmetr	Area %
###	6.741	9.917e4	99.835	99.804	4	99.835
###	8.659	163.441	0.165	0.196	0	0.165

5-(Thiophen-2-yl)-1-(p-tolyl)-3-(p-tolylamino)-1,5-dihydro-2H-pyrrol-2-one (**4l**).
Purity: **99.6%**

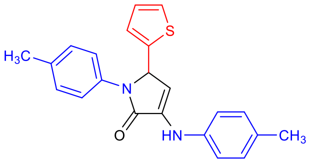

4l

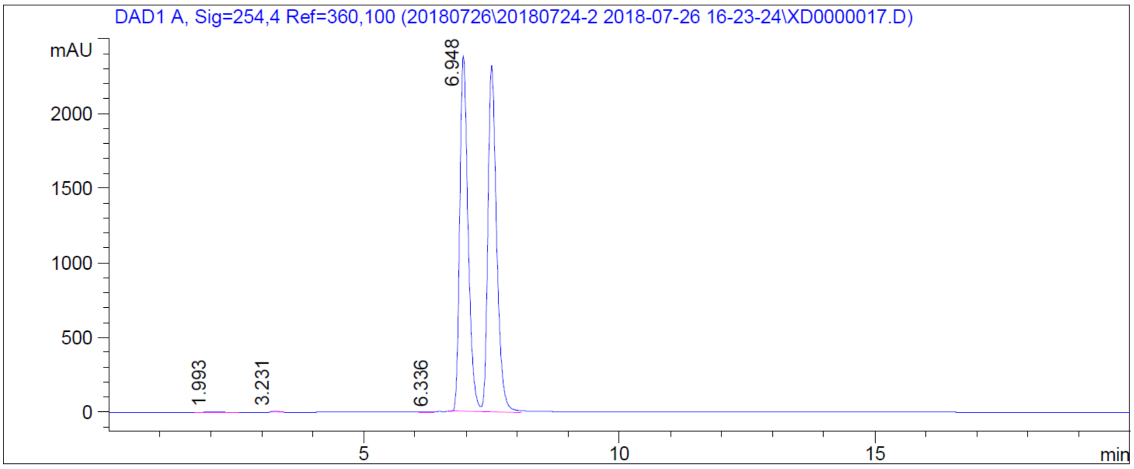

#	Meas. R	Respons	Respons	Height	Symmetr	Area %
###	1.993	72.748	0.133	0.055	0	0.133
###	3.231	53.902	0.099	0.272	0	0.099
###	6.336	101.904	0.186	0.271	6	0.186
###	6.948	5.446e4	99.582	99.402	0	99.582

5-(Naphthalen-2-yl)-1-(p-tolyl)-3-(p-tolylamino)-1,5-dihydro-2H-pyrrol-2-one (**4m**).
Purity: **97.7%**

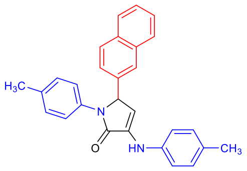

4m

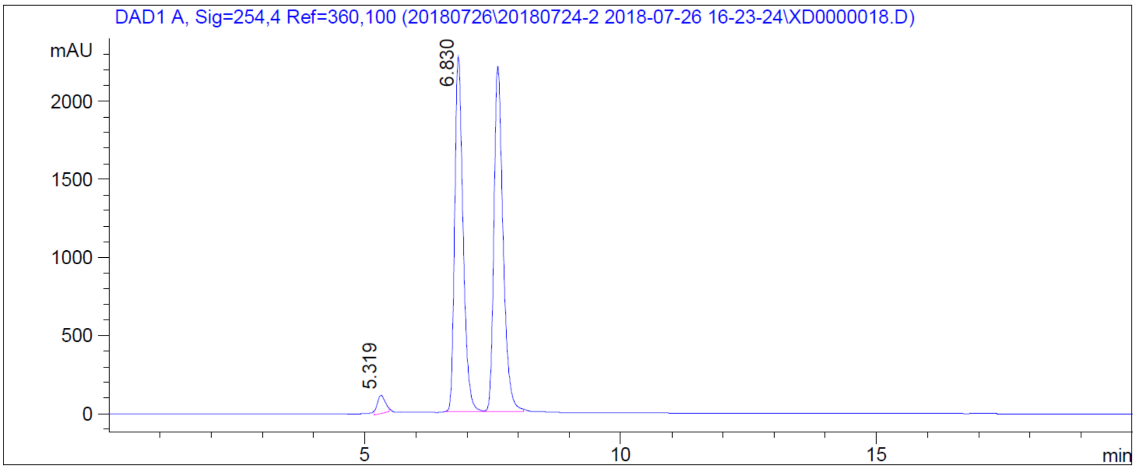

#	Meas. R	Respons	Respons	Height	Symmetr	Area %
###	5.319	1.262e3	2.301	4.892	1	2.301
###	6.830	5.362e4	97.699	95.108	0	97.699

5-Methyl-1-(p-tolyl)-3-(p-tolylamino)-1H-pyrrol-2(5H)-one (**4n**).
Purity: **99.1%**

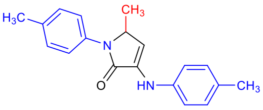

4n

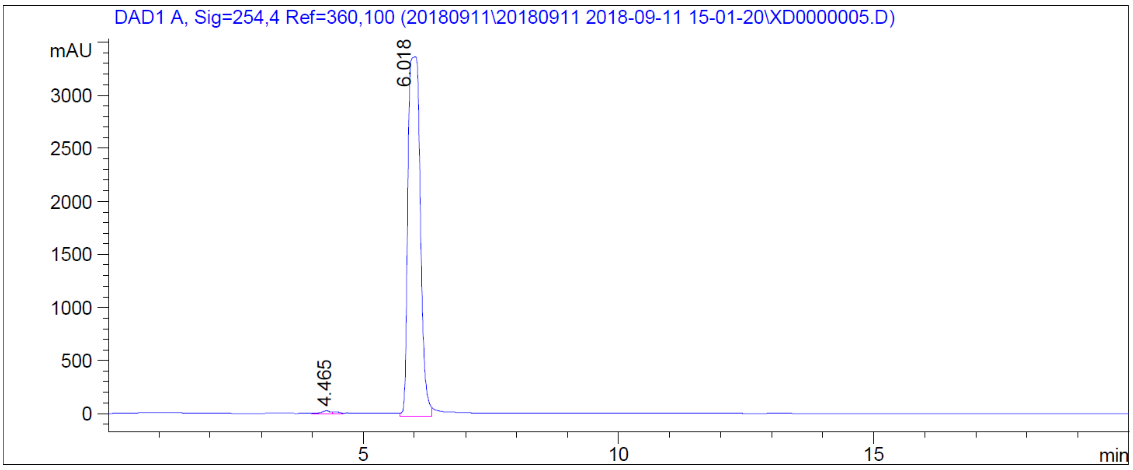

#	Meas. R	Respons	Respons	Height	Symmetr	Area %
###	4.280	352.716	0.616	0.817	2	0.616
###	4.465	182.397	0.318	0.556	1	0.318
###	6.018	5.676e4	99.066	98.628	1	99.066

5-Iso-propyl-1,3-di-p-tolyl-1H-pyrrol-2(5H)-one (**4o**).

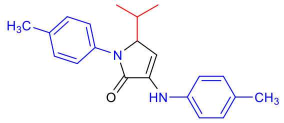

4o

Purity: **99.9 %**

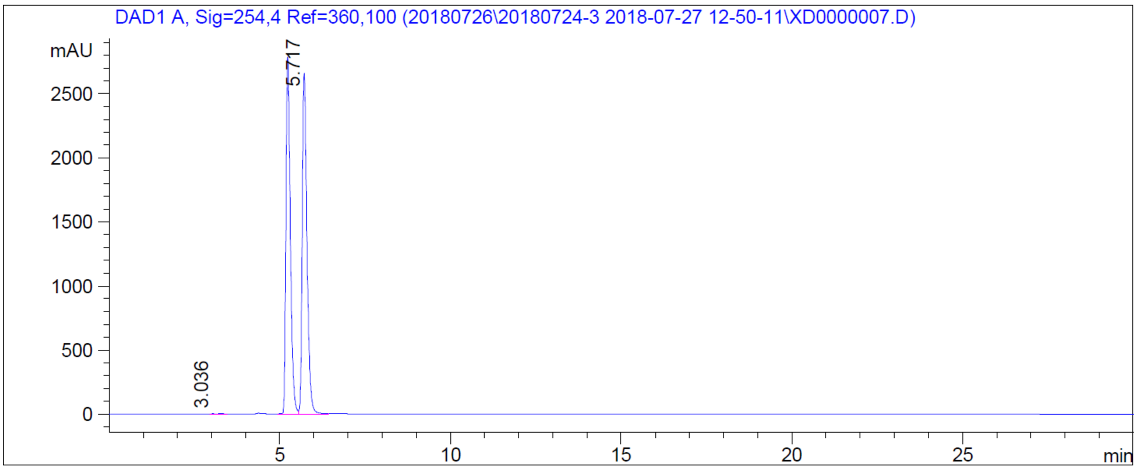

#	Meas. R	Respons	Respons	Height	Symmetr	Area %
###	3.036	17.310	0.034	0.043	0	0.034
###	3.264	32.012	0.064	0.067	1	0.064
###	5.235	2.495e4	49.522	51.079	1	49.522
###	5.717	2.538e4	50.380	48.811	1	50.380

5-Iso-butyl-1-(p-tolyl)-3-(p-tolylamino)-1,5-dihydro-2H-pyrrol-2-one (**4p**).

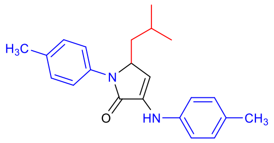

4p

Purity: **99.1%**

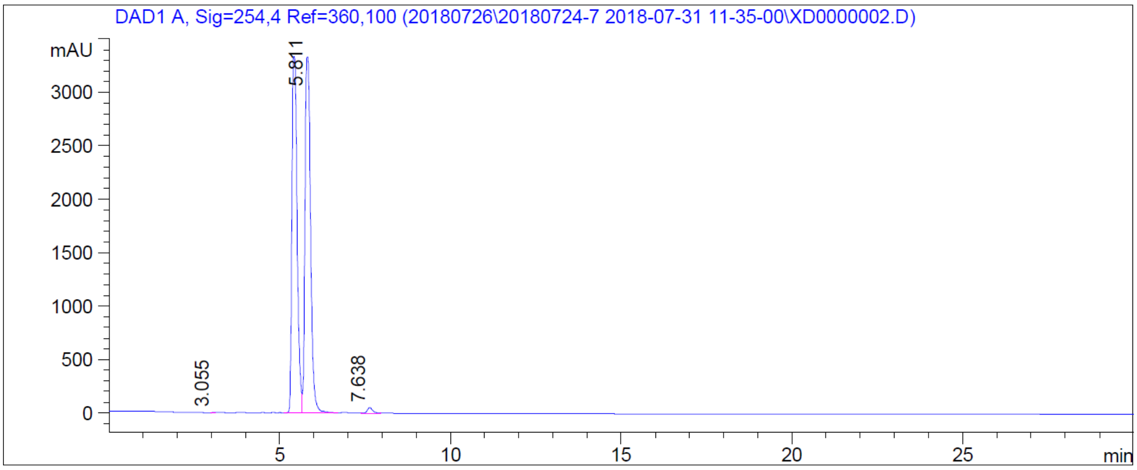

#	Meas. R	Respons	Respons	Height	Symmetr	Area %
###	3.055	9.401	0.013	0.028	1	0.013
###	5.423	3.577e4	48.526	49.614	1	48.526
###	5.811	3.732e4	50.632	49.557	1	50.632
###	7.638	611.548	0.830	0.801	1	0.830

1-Cyclohexyl-5-(p-tolyl)-3-(p-tolylamino)-1,5-dihydro-2H-pyrrol-2-one (**4q**).

Purity: **99.0%**

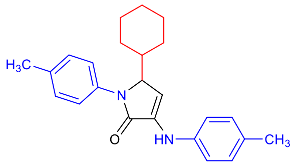

4q

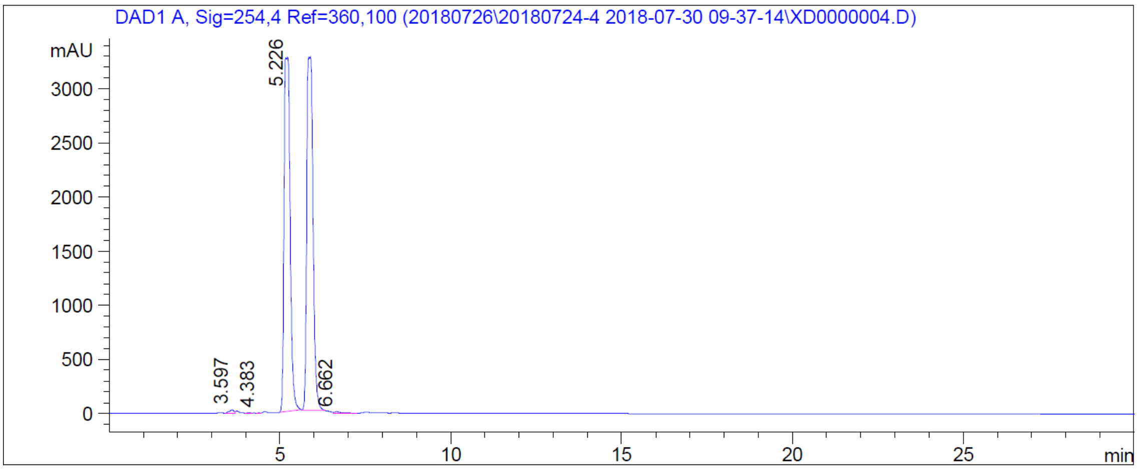

#	Meas. R	Respons	Respons	Height	Symmetr	Area %
###	3.597	347.810	0.422	0.501	2	0.422
###	4.088	58.050	0.070	0.086	1	0.070
###	4.383	34.456	0.042	0.081	1	0.042
###	5.226	4.047e4	49.127	49.562	1	49.127
###	5.893	4.113e4	49.921	49.514	1	49.921
###	6.662	344.045	0.418	0.257	0	0.418

(E)-5-Styryl-1-(p-tolyl)-3-(p-tolylamino)-1,5-dihydro-2H-pyrrol-2-one (**4r**).

Purity: **99.8%**

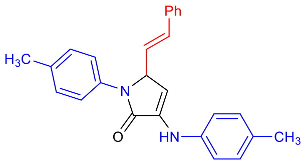

4r

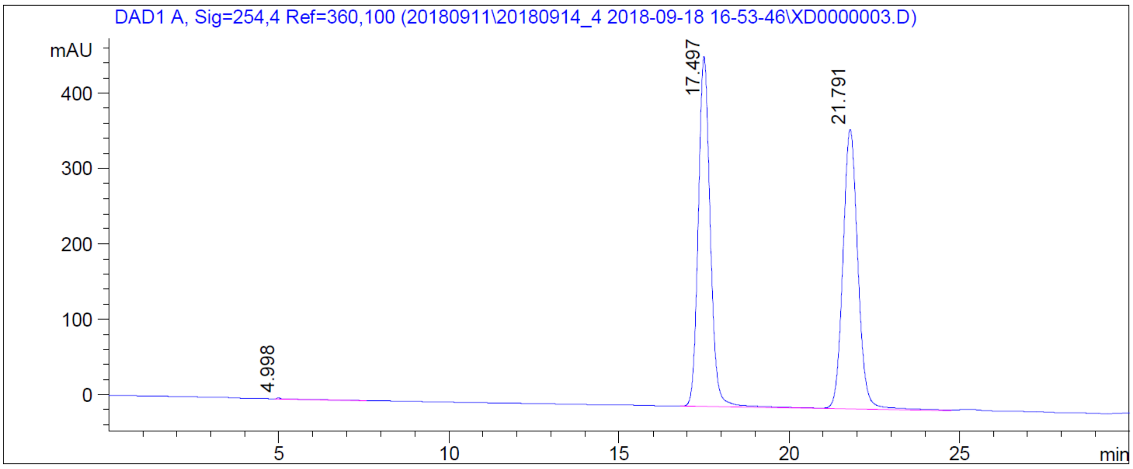

#	Meas. R	Respons	Respons	Height	Symmetr	Area %
###	4.998	32.831	0.144	0.239	0	0.144
###	17.497	1.138e4	49.959	55.478	1	49.959
###	21.791	1.136e4	49.897	44.283	1	49.897

Ethyl 5-oxo-1, 4-di-p-tolyl-2,5-dihydro-1H-pyrrol-2-carboxylate (**4s**).

Purity: **95.6%**

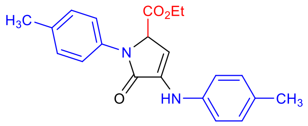

4s

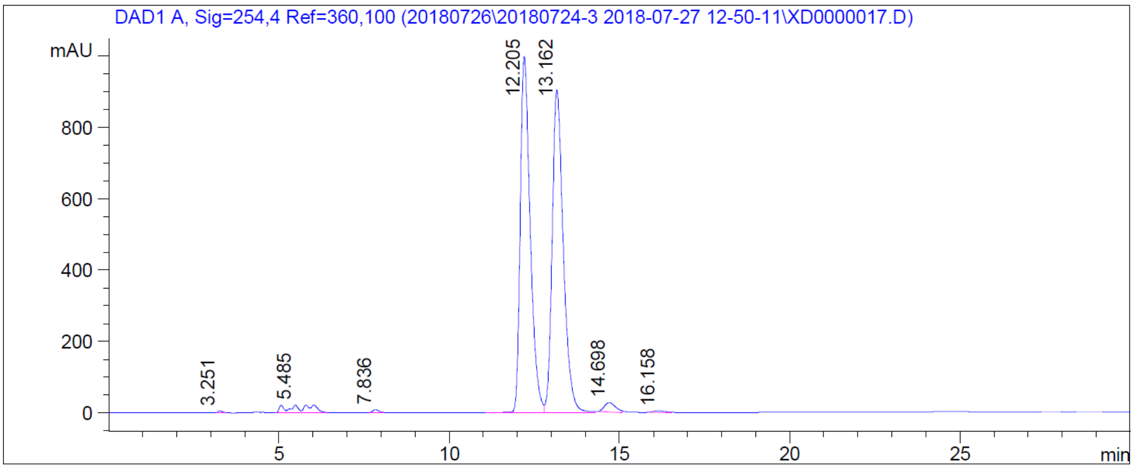

#	Meas. R	Respons	Respons	Height	Symmetr	Area %
###	3.251	46.181	0.108	0.262	0	0.108
###	5.485	1.008e3	2.352	1.079	1	2.352
###	7.836	111.112	0.259	0.439	1	0.259
###	12.205	2.034e4	47.461	50.674	1	47.461
###	13.162	2.063e4	48.138	45.960	1	48.138
###	14.698	610.470	1.424	1.343	1	1.424
###	16.158	110.490	0.258	0.242	1	0.258

5-(Perfluorophenyl)-1-(p-tolyl-3-(p-tolylamino)-1H-pyrrol-2(5H)-one (**4t**).

Purity: **99.7%**

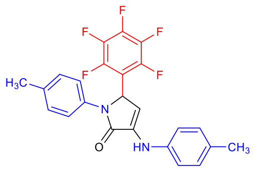

4t

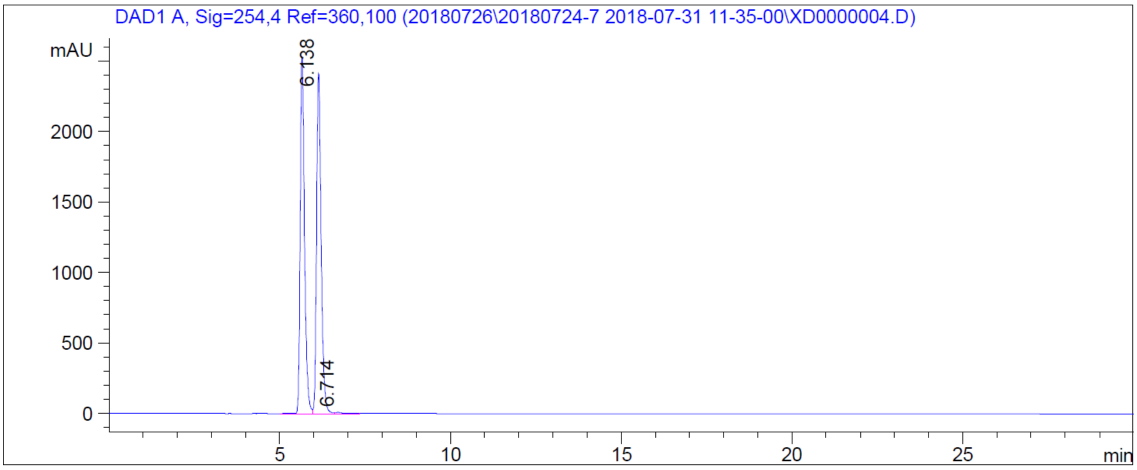

#	Meas. R	Respons	Respons	Height	Symmetr	Area %
###	5.656	2.235e4	49.582	51.101	1	49.582
###	6.138	2.258e4	50.091	48.709	1	50.091
###	6.714	147.239	0.327	0.191	0	0.327

1-(p-Tolyl)-3-(p-tolylamino)-5-(trifluoromethyl)-1,5-dihydro-2H-pyrrol-2-one (**4u**).

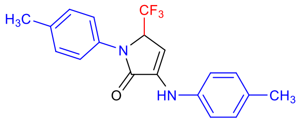

4u

Purity: **99.5%**

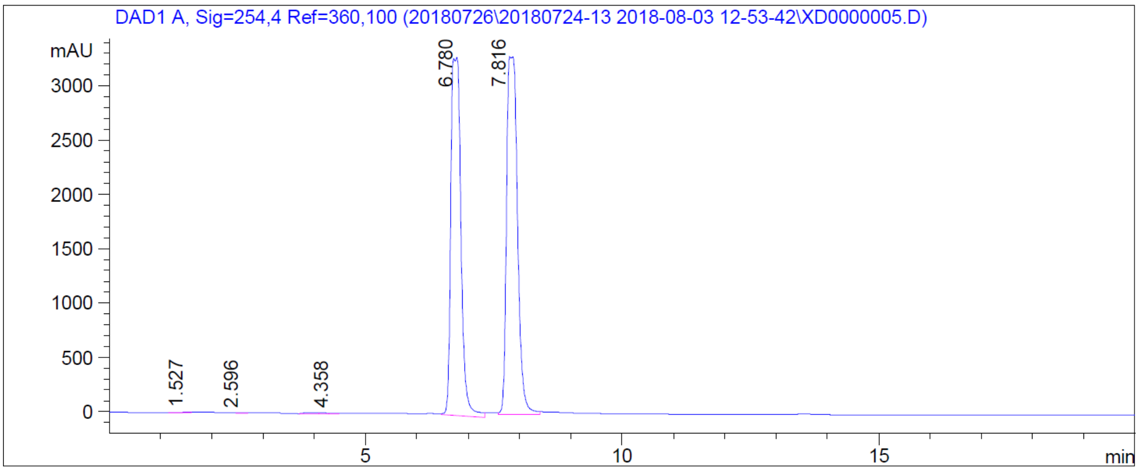

#	Meas. R	Respons	Respons	Height	Symmetr	Area %
###	1.527	77.797	0.083	0.085	2	0.083
###	2.596	66.084	0.070	0.076	1	0.070
###	3.955	161.818	0.172	0.126	2	0.172
###	4.160	95.198	0.101	0.134	1	0.101
###	4.358	74.769	0.079	0.106	1	0.079
###	6.780	4.527e4	48.100	49.784	1	48.100
###	7.816	4.838e4	51.395	49.688	0	51.395

Diethyl ((5-oxo-1-(p-tolyl)-4-(p-tolylamino)-2,5-dihydro-1H-pyrrol-2-yl)methyl)-phosphonate (**4v**).

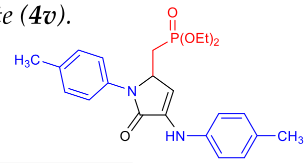

4v

Purity: **99.1%**

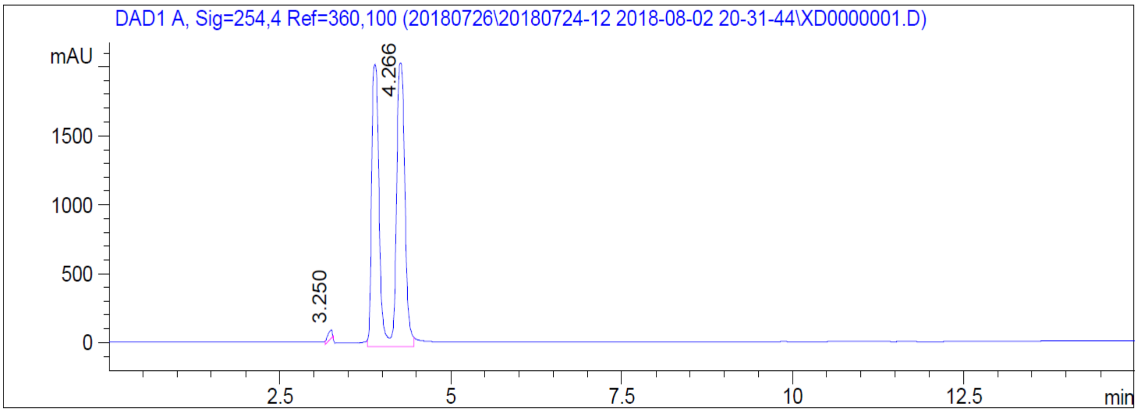

#	Meas. R	Respons	Respons	Height	Symmetr	Area %
###	3.250	299.378	0.917	3.027	4	0.917
###	4.266	3.236e4	99.083	96.973	2	99.083

*(S)-Diethyl ((5-oxo-1-(p-tolyl)-4-(p-tolylamino)-2,5-dihydro-1H-pyrrol-2-yl)methyl)phosphonate (**S-4v**).*

Purity: **98.3%**

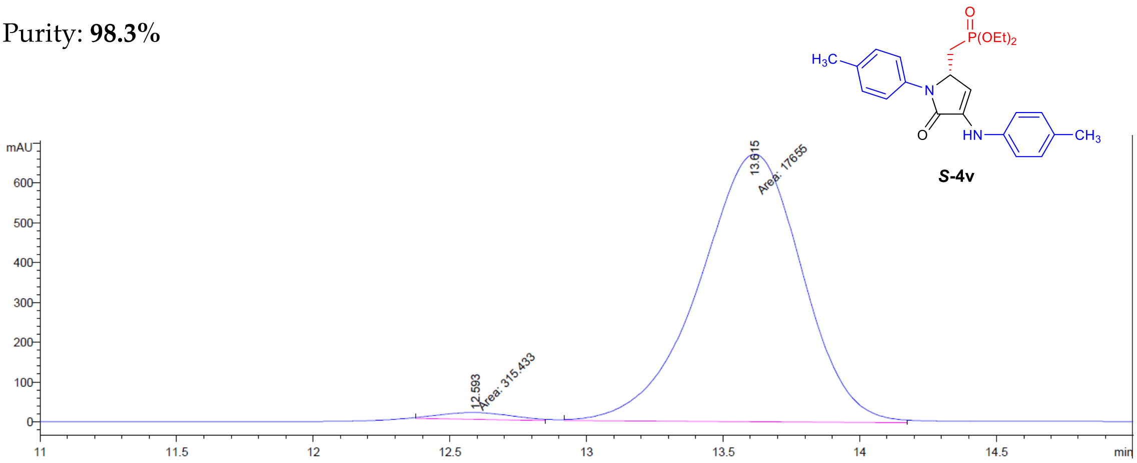

Peak #	RetTime [min]	Type	Width [min]	Area [mAU*s]	Height [mAU]	Area %
1	12.593	MM	0.3033	315.43341	17.33072	1.7553
2	13.615	MM	0.4373	1.76550e4	672.88861	98.2447

*(R)-Diethyl ((5-oxo-1-(p-tolyl)-4-(p-tolylamino)-2,5-dihydro-1H-pyrrol-2-yl)methyl)phosphonate (**R-4v**).*

Purity: **98.4%**

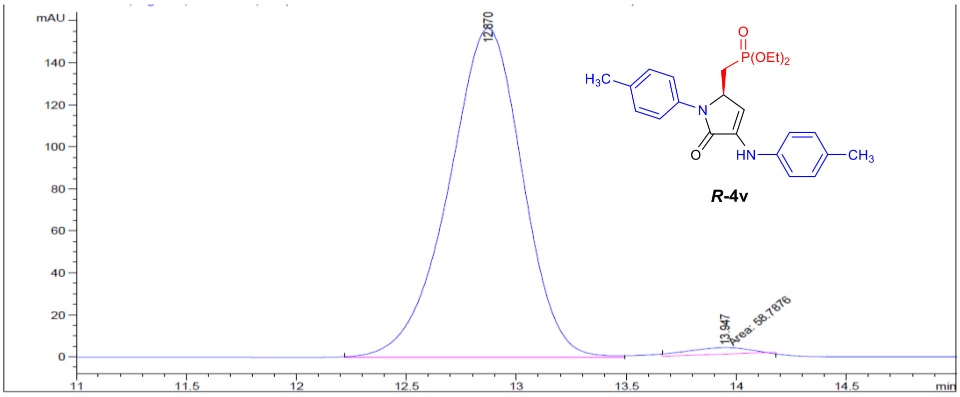

Peak #	RetTime [min]	Type	Width [min]	Area [mAU*s]	Height [mAU]	Area %
1	12.870	BB	0.3581	3652.83252	156.62250	98.4161
2	13.947	MM	0.3196	58.78765	3.06588	1.5839

*5-((Diphenylphosphoryl)methyl)-1-(p-tolyl)-3-(p-tolylamino)-1H-pyrrol-2(5H)-one (**4w**).*

Purity: **99.0%**

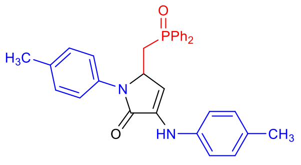

4w

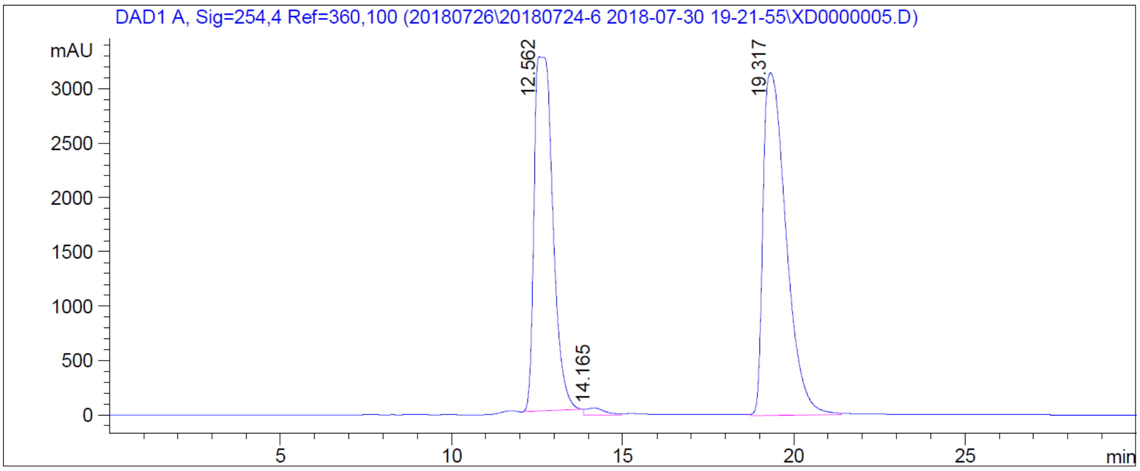

#	Meas. R	Respons	Respons	Height	Symmetr	Area %
###	12.562	1.238e5	45.130	50.316	0	45.130
###	14.165	2.457e3	0.896	0.999	1	0.896
###	19.317	1.480e5	53.974	48.686	0	53.974

*4-Methyl-5-(4-nitrophenyl)-1-(p-tolyl)-3-(p-tolylamino)-1,5-dihydro-2H-pyrrol-2-one (**5a**).*

Purity: **99.0%**

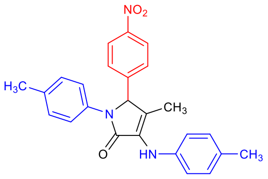

5a

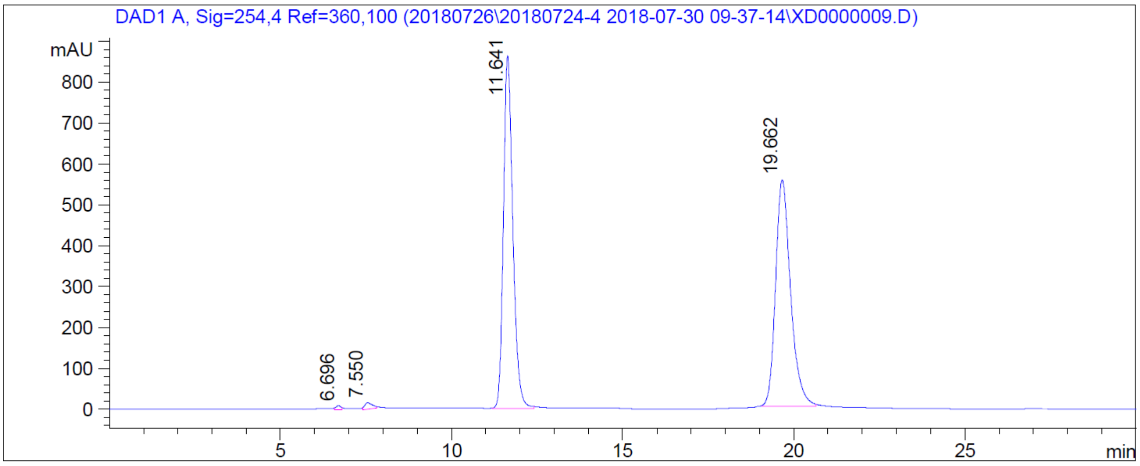

#	Meas. R	Respons	Respons	Height	Symmetr	Area %
###	6.696	92.639	0.275	0.639	1	0.275
###	7.550	246.799	0.732	1.082	1	0.732
###	11.641	1.648e4	48.879	59.875	1	48.879
###	19.662	1.690e4	50.115	38.405	1	50.115

4-Benzyl-5-(p-nitrophenyl)-1-(p-tolyl)-3-(p-tolylamino)-1,5-dihydro-2H-pyrrol-2-one (**5b**).

Purity: **97.6%**

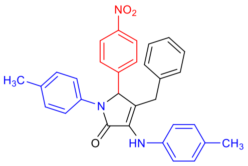

5b

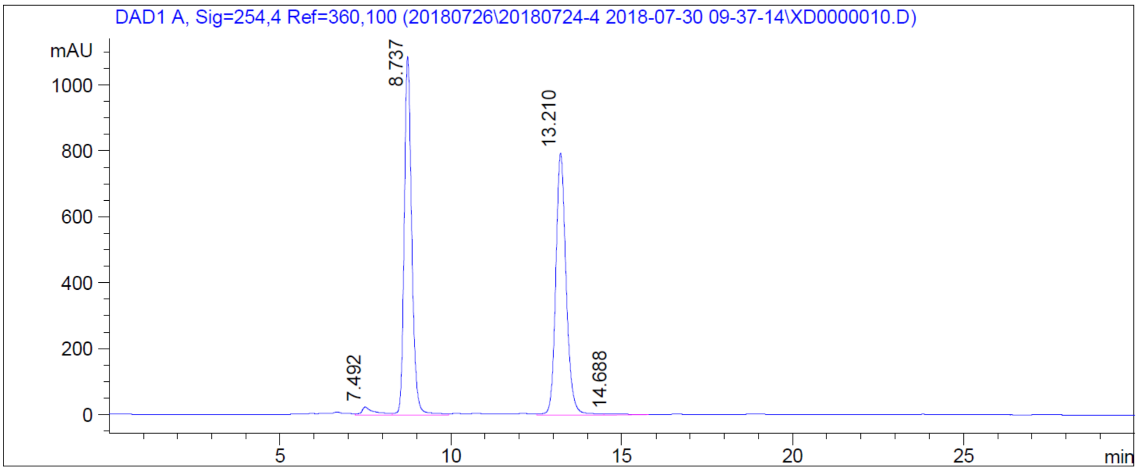

#	Meas. R	Respons	Respons	Height	Symmetr	Area %
###	7.492	645.454	1.883	1.269	0	1.883
###	8.737	1.673e4	48.806	56.972	1	48.806
###	13.210	1.674e4	48.842	41.610	1	48.842
###	14.688	160.494	0.468	0.149	0	0.468

Methyl 5-oxo-2-phenyl-1-(p-tolyl)-4-(p-tolylamino)-2,5-dihydro-1H-pyrrole-3-carboxylate (**5c**).

Purity: **99.4%**

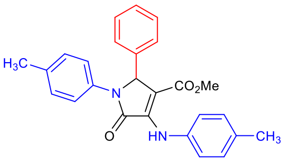

5c

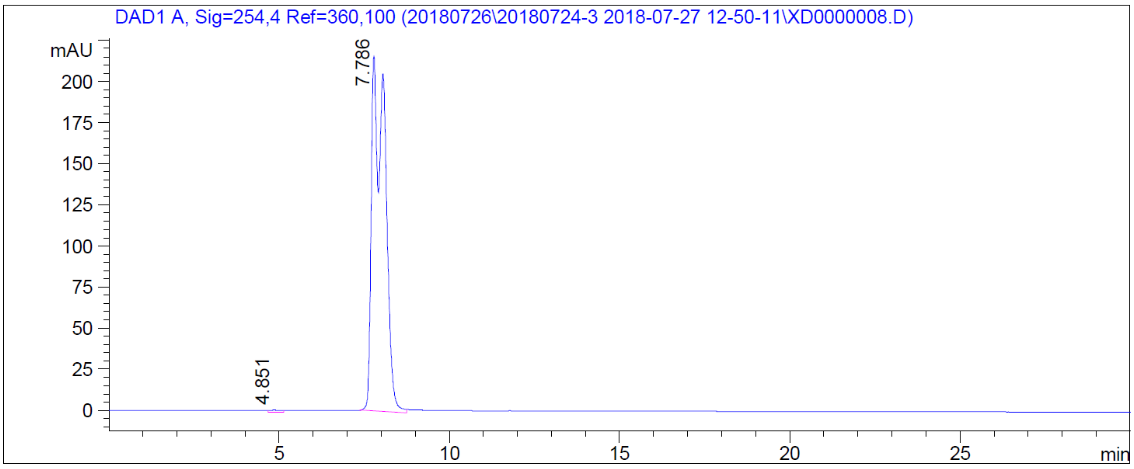

#	Meas. R	Respons	Respons	Height	Symmetr	Area %
###	4.851	34.798	0.587	0.649	1	0.587
###	7.786	5.898e3	99.413	99.351	0	99.413

*Diethyl (5-oxo-1-(p-tolyl)-4-(p-tolylamino)-2,5-dihydro-1H-pyrrol-3-yl)phosphonate (**5d**).*

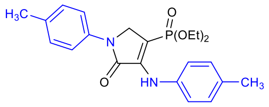

5d

Purity: **99.7%**

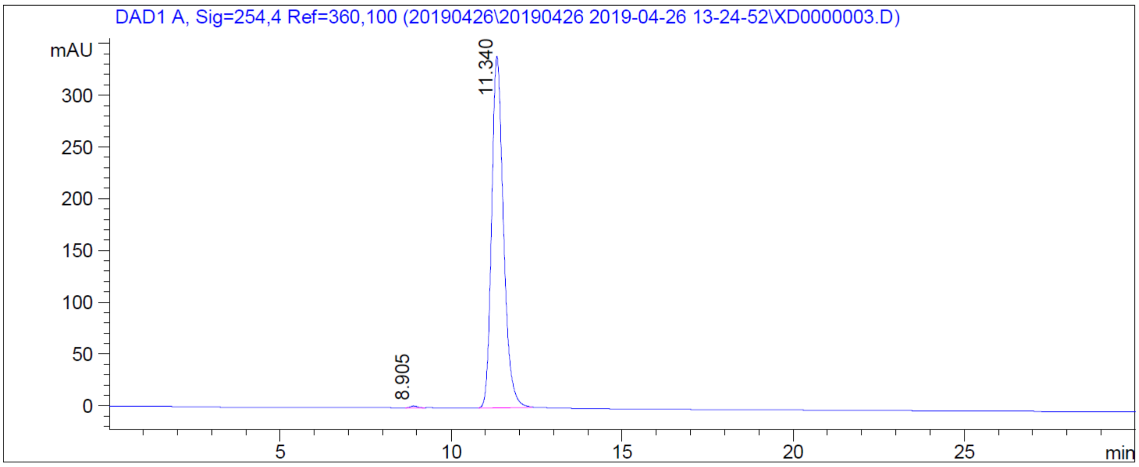

#	Meas. R	Respons	Respons	Height	Symmetr	Area %
###	8.905	23.731	0.288	0.523	1	0.288
###	11.340	8.203e3	99.712	99.477	1	99.712

*1-(p-Methoxyphenyl)-3-((p-methoxyphenyl)amino)-5-phenyl-1,5-dihydro-2H-pyrrol-2-one (**6a**).*

Purity: **99.8%**

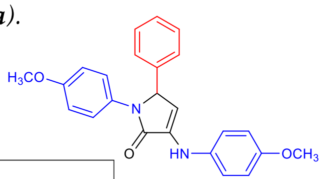

6a

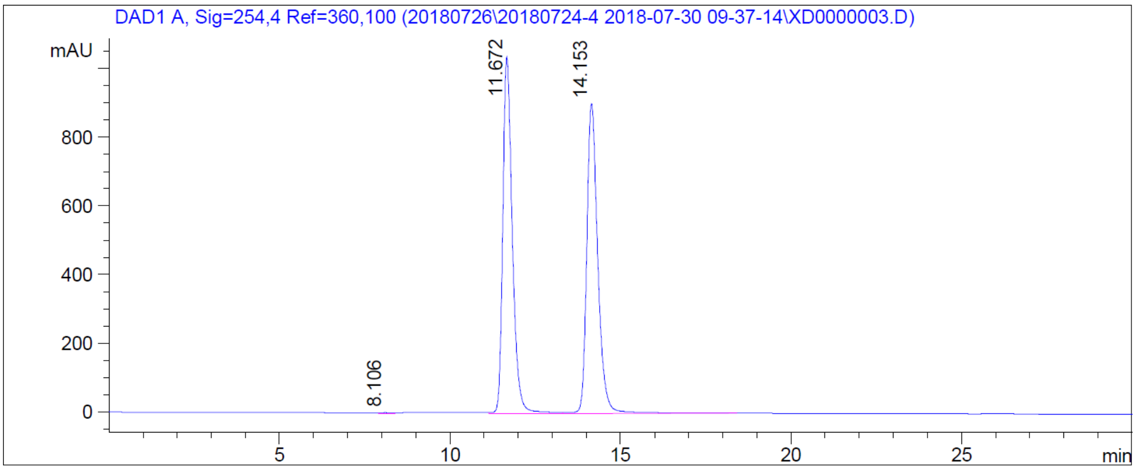

#	Meas. R	Respons	Respons	Height	Symmetr	Area %
###	8.106	52.097	0.130	0.104	1	0.130
###	11.672	1.998e4	49.764	53.454	1	49.764
###	14.153	2.012e4	50.106	46.442	1	50.106

*1-(p-Methoxyphenyl)-3-((p-methoxyphenyl)amino)-5-(p-nitrophenyl)-1H-pyrrol-2(5H)-one (**6b**).*

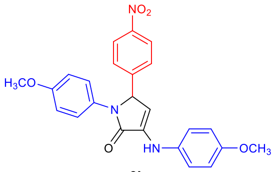

6b

Purity: **97.8%**

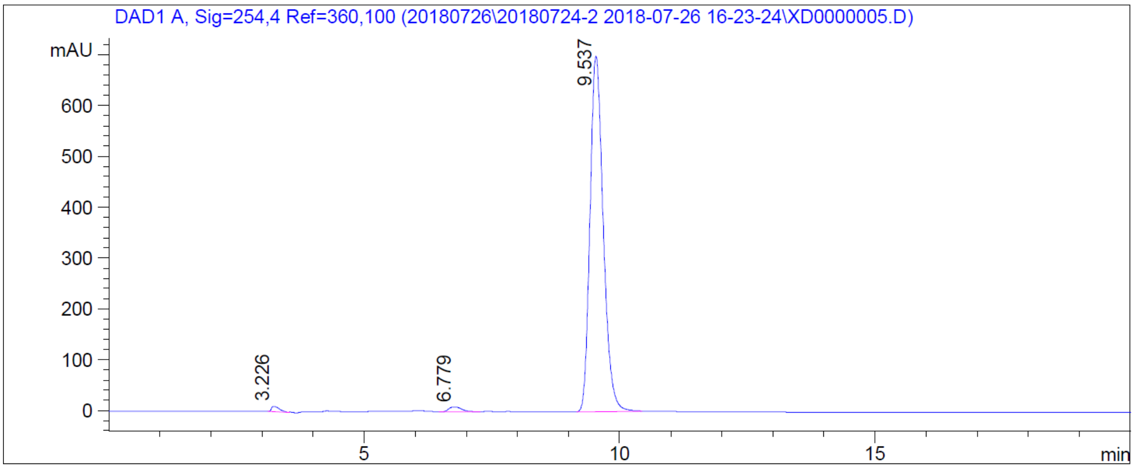

#	Meas. R	Respons	Respons	Height	Symmetr	Area %
###	3.226	122.018	0.946	1.501	0	0.946
###	6.779	159.253	1.235	1.273	1	1.235
###	9.537	1.262e4	97.820	97.225	1	97.820

*Diethyl ((1-(p-methoxyphenyl)-4-((p-methoxyphenyl)amino)-5-oxo-2,5-dihydro-1H-pyrrol-2-yl)methyl)phosphonate (**6c**).*

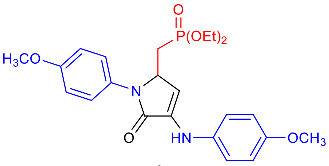

6c

Purity: **98.6%**

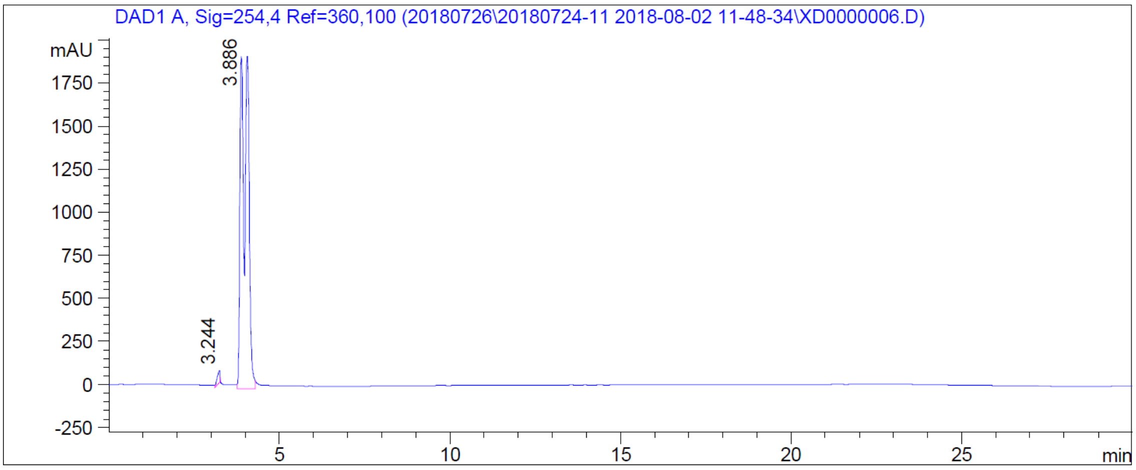

#	Meas. R	Respons	Respons	Height	Symmetr	Area %
###	3.244	419.003	1.427	3.741	5	1.427
###	3.886	2.894e4	98.573	96.259	0	98.573

*1-(p-Chlorophenyl)-3-((p-chlorophenyl)amino)-5-(p-nitrophenyl)-1,5-dihydro-2H-pyrrol-2-one (**7**).*

Purity: **98.4%**

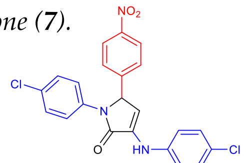

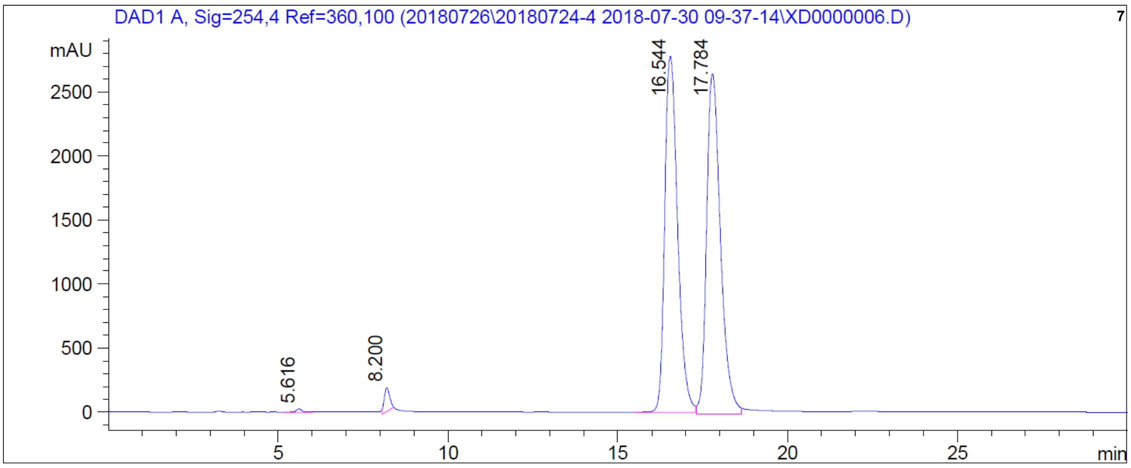

#	Meas. R	Respons	Respons	Height	Symmetr	Area %
###	5.616	372.097	0.247	0.469	1	0.247
###	8.200	2.010e3	1.336	3.300	1	1.336
###	16.544	7.271e4	48.316	49.166	1	48.316
###	17.784	7.540e4	50.101	47.064	1	50.101

*1,3-Bis(p-bromophenyl)-5-(p-nitrophenyl)-1H-pyrrol-2(5H)-one (**8a**).*

Purity: **98.0%**

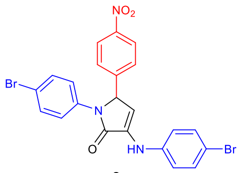

8a

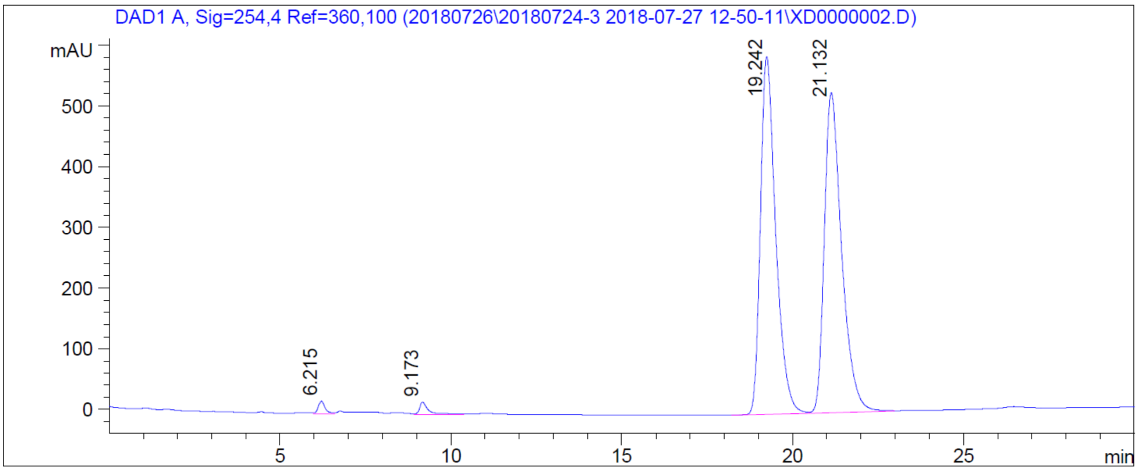

#	Meas. R	Respons	Respons	Height	Symmetr	Area %
###	6.215	319.533	0.849	1.835	1	0.849
###	9.173	389.693	1.035	1.768	0	1.035
###	19.242	1.835e4	48.759	50.856	1	48.759
###	21.132	1.858e4	49.356	45.542	1	49.356

1-(p-Bromophenyl)-3-((p-bromophenyl)amino)-5-(p-(trifluoromethyl)phenyl)-1,5-dihydro-2H-pyrrol-2-one (**8b**).

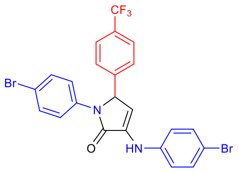

8b

Purity: **99.4%**

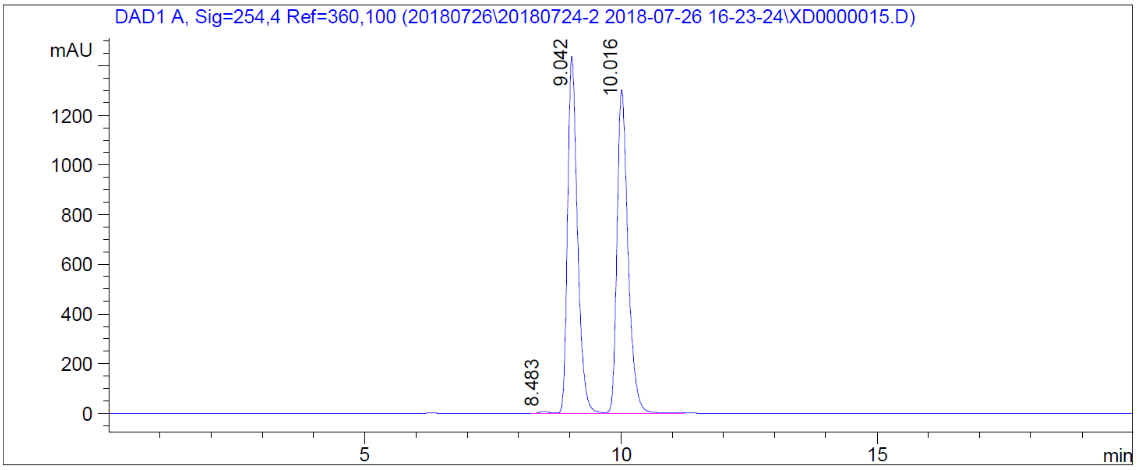

#	Meas. R	Respons	Respons	Height	Symmetr	Area %
###	8.483	103.132	0.264	0.297	1	0.264
###	9.042	1.943e4	49.744	52.304	1	49.744
###	10.016	1.953e4	49.992	47.398	1	49.992

1-(m-Chlorophenyl)-3-((m-chlorophenyl)amino)-5-(p-nitrophenyl)-1,5-dihydro-2H-pyrrol-2-one (**9**).

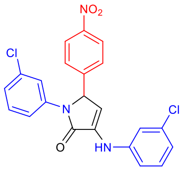

9

Purity: **98.9%**

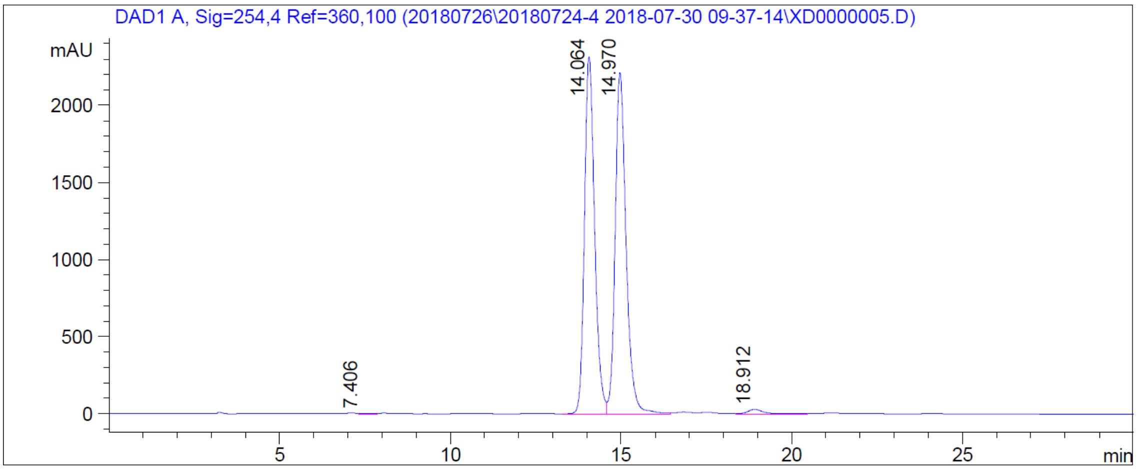

#	Meas. R	Respons	Respons	Height	Symmetr	Area %
###	7.406	66.716	0.068	0.087	0	0.068
###	14.064	4.750e4	48.581	50.800	1	48.581
###	14.970	4.922e4	50.339	48.462	1	50.339
###	18.912	990.094	1.013	0.651	1	1.013

*1-(o-Fluorophenyl)-3-((o-fluorophenyl)amino)-5-(p-nitrophenyl)-1H-pyrrol-2(5H)-one (**10**).*

Purity: **99.5%**

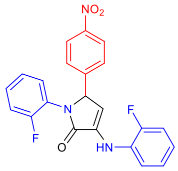

10

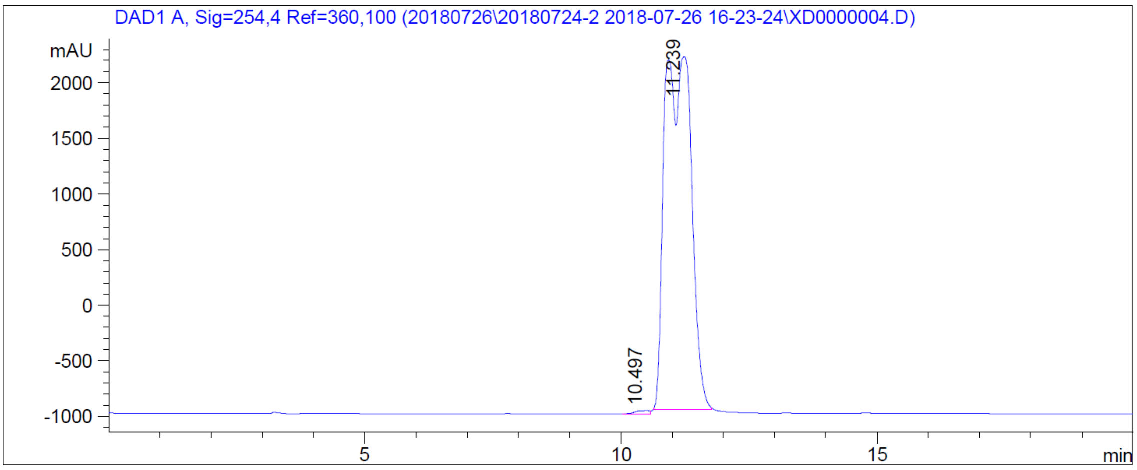

#	Meas. R	Respons	Respons	Height	Symmetr	Area %
###	10.497	532.769	0.455	0.880	3	0.455
###	11.239	1.167e5	99.545	99.120	2	99.545

*1-(m-(Trifluoromethyl)phenyl)-3-((m-(trifluoromethyl)phenyl)amino)-1,5-dihydro-2H-pyrrol-2-one (**11a**).*

Purity: **99.9%**

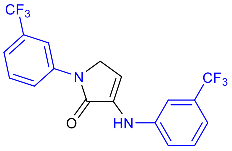

11a,

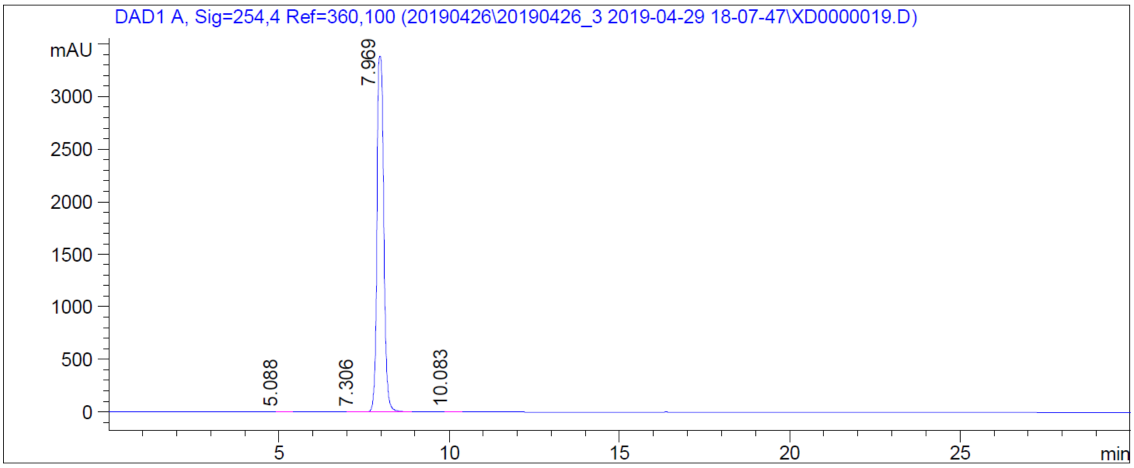

#	Meas. R	Respons	Respons	Height	Symmetr	Area %
###	5.088	16.204	0.034	0.037	0	0.034
###	7.306	11.217	0.024	0.030	2	0.024
###	7.969	4.701e4	99.902	99.887	1	99.902

*5-(p-Nitrophenyl)-1-(m-(trifluoromethyl)phenyl)-3-((m-(trifluoromethyl)phenyl)amino)-1H-pyrrol-2(5H)-one (**11b**).*

Purity: **99.2%**

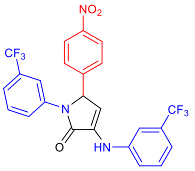

11b

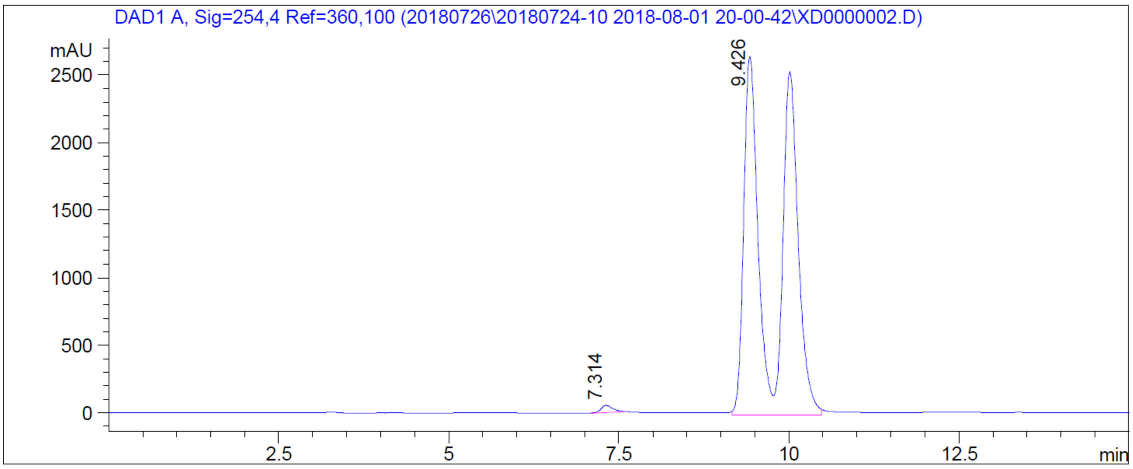

#	Meas. R	Respons	Respons	Height	Symmetr	Area %
###	7.314	609.448	0.782	1.949	1	0.782
###	9.426	7.730e4	99.218	98.051	0	99.218

*5-(p-Nitrophenyl)-1-(quinolin-3-yl)-3-(quinolin-3-ylamino)-1H-pyrrol-2(5H)-one (**12**).*

Purity: **96.9%**

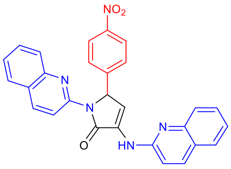

12

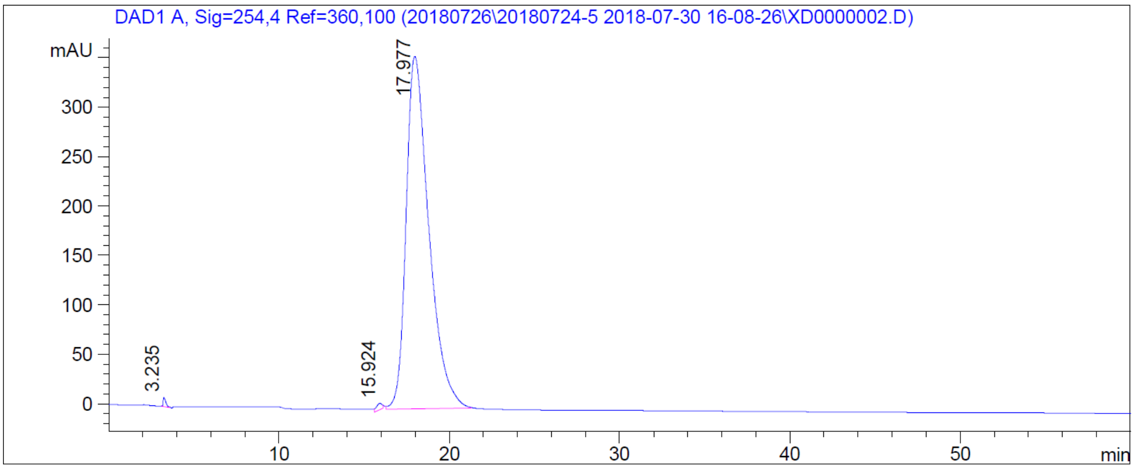

#	Meas. R	Respons	Respons	Height	Symmetr	Area %
###	3.235	101.366	0.304	2.550	0	0.304
###	15.924	141.096	0.423	1.655	2	0.423
###	17.977	3.314e4	99.274	95.796	1	99.274

*Ethyl 2-methyl-5-oxo-1-(p-tolyl)-4-(p-tolylamino)-2,5-dihydro-1H-pyrrole-2-carboxylate (**16**).*

Purity: **99.8%**

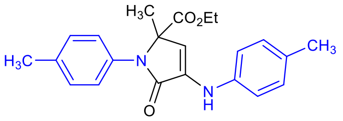

16

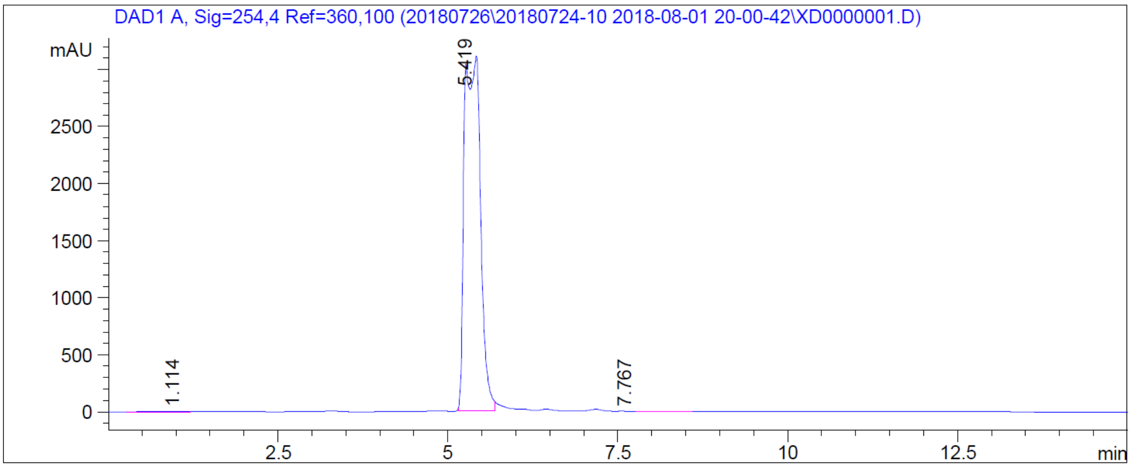

#	Meas. R	Respons	Respons	Height	Symmetr	Area %
###	1.114	47.633	0.093	0.049	5	0.093
###	5.419	5.114e4	99.766	99.848	2	99.766
###	7.767	72.453	0.141	0.102	0	0.141

Ethyl (2S, 4R*)-2-methyl-5-oxo-1-(p-tolyl)-4-(p-tolylamino)pyrrolidine-2-carboxylate (**17**).*

Purity: **96.7%**

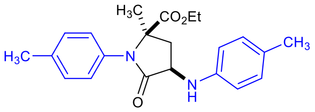

17

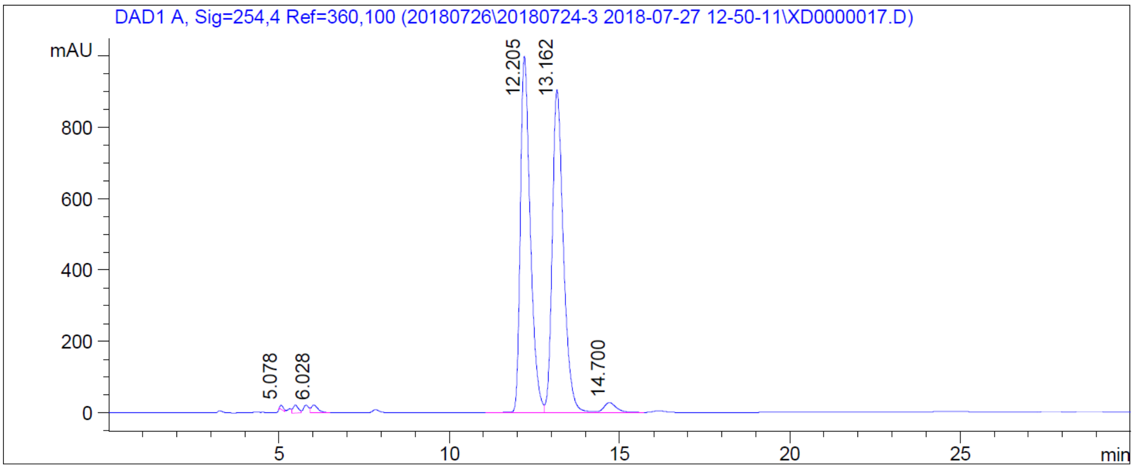

#	Meas. R	Respons	Respons	Height	Symmetr	Area %
###	5.078	84.633	0.200	0.625	0	0.200
###	5.485	247.213	0.584	1.128	1	0.584
###	6.028	319.646	0.756	1.114	1	0.756
###	12.205	2.031e4	48.004	50.198	1	48.004
###	13.162	2.061e4	48.714	45.524	1	48.714
###	14.700	736.895	1.742	1.411	1	1.742

((3R, 5R*)-1-(p-tolyl)-3-(p-tolylamino)-5-(trifluoromethyl)pyrrolidin-2-one (**18a**).*

Purity: **98.3%**

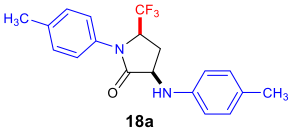

18a

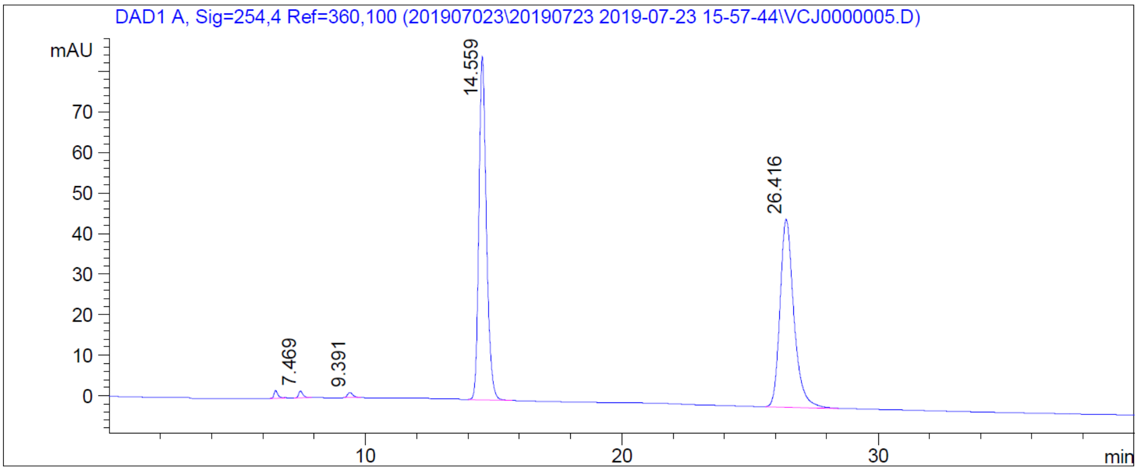

#	Meas. R	Respons	Respons	Height	Symmetr	Area %
###	6.498	21.128	0.595	1.428	1	0.595
###	7.469	19.950	0.562	1.243	1	0.562
###	9.391	18.969	0.534	0.950	1	0.534
###	14.559	1.742e3	49.074	62.286	1	49.074
###	26.416	1.748e3	49.234	34.092	1	49.234

Diethyl (((2R, 4R*)-5-oxo-1-(p-tolyl)-4-(p-tolylamino)pyrrolidin-2-yl)methyl)phosphonate (18b).*

Purity: **98.6%**

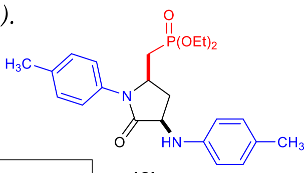

18b

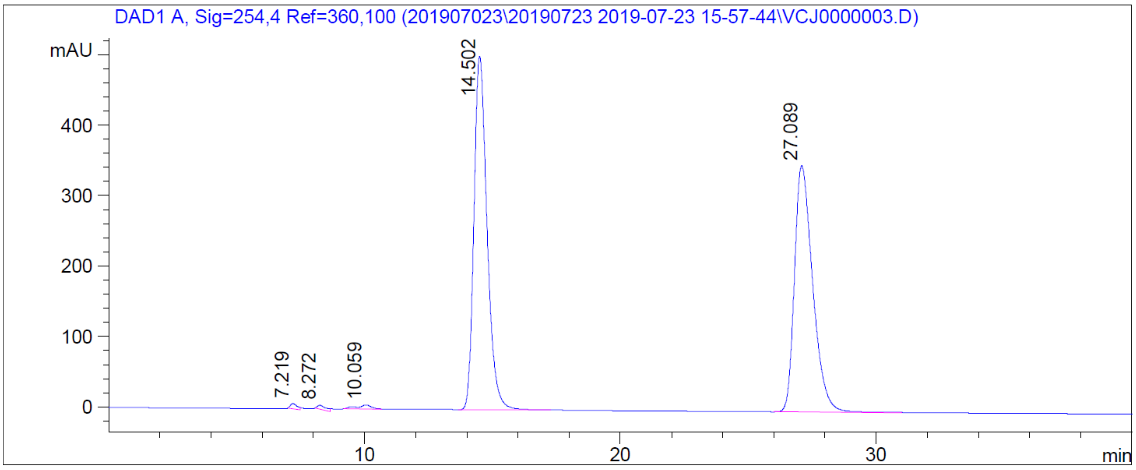

#	Meas. R	Respons	Respons	Height	Symmetr	Area %
###	7.219	130.617	0.365	0.821	1	0.365
###	8.272	124.708	0.348	0.602	0	0.348
###	10.059	232.178	0.648	0.679	2	0.648
###	14.502	1.800e4	50.258	57.724	1	50.258
###	27.089	1.733e4	48.381	40.174	1	48.381

(3R, 5R*)-1-(p-tolyl)-3-((p-methoxyphenyl)amino)-5-phenylpyrrolidin-2-one* (**18c**).

Purity: **98.6%**

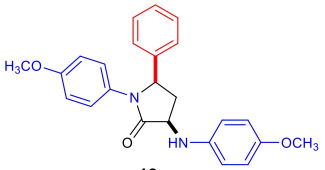

18c

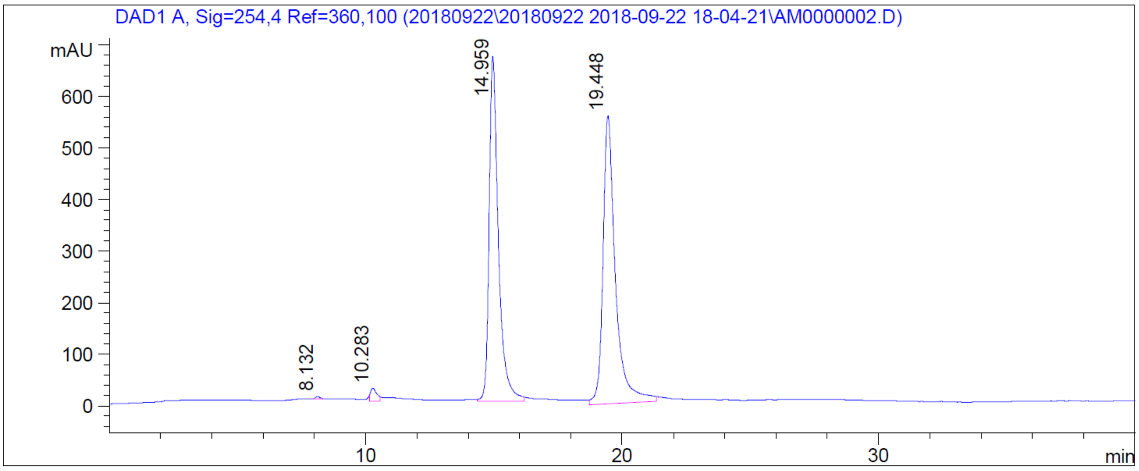

#	Meas. R	Respons	Respons	Height	Symmetr	Area %
###	8.132	49.665	0.137	0.349	1	0.137
###	10.283	452.467	1.247	2.029	1	1.247
###	14.959	1.688e4	46.526	53.236	1	46.526
###	19.448	1.890e4	52.090	44.387	1	52.090

Diethyl ((3,4-dimethyl-5-oxo-1-(p-tolyl)-4-(p-tolylamino)-4,5-dihydro-1H-pyrrol-2-yl)methyl)phosphonate (**19**).

Purity: **99.6%**

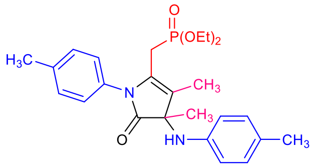

19

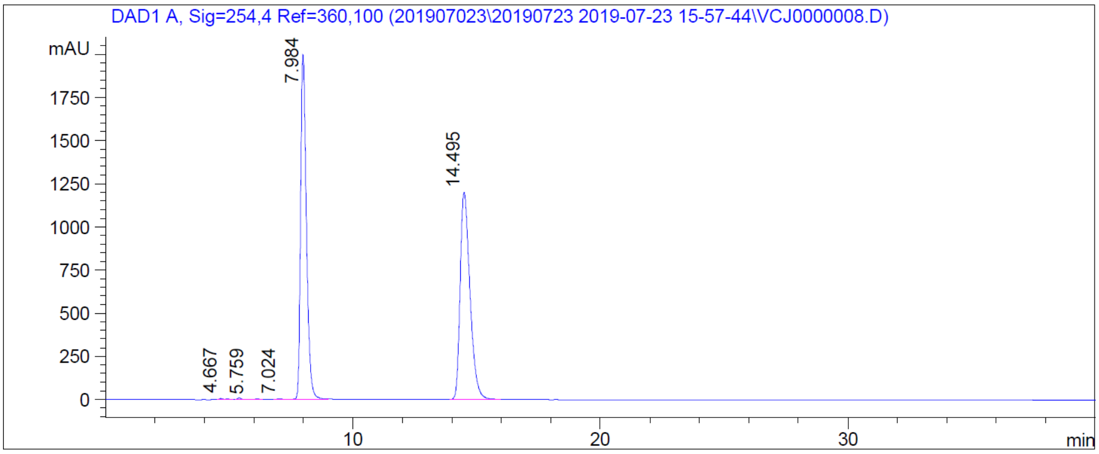

#	Meas. R	Respons	Respons	Height	Symmetr	Area %
###	4.667	39.363	0.060	0.145	1	0.060
###	4.927	27.108	0.041	0.093	1	0.041
###	5.404	88.937	0.134	0.336	1	0.134
###	5.759	7.826	0.012	0.031	1	0.012
###	6.132	39.840	0.060	0.155	1	0.060
###	7.024	44.119	0.067	0.073	0	0.067
###	7.984	3.273e4	49.477	61.939	1	49.477
###	14.495	3.317e4	50.149	37.227	1	50.149

*(2Z, 3E, 5E)-6-Phenyl-N-(p-tolyl)-2-(p-tolyl imino)hexa-3,5-dienamide (**20a**).*

Purity: **98.4%**

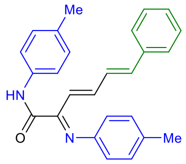

20a

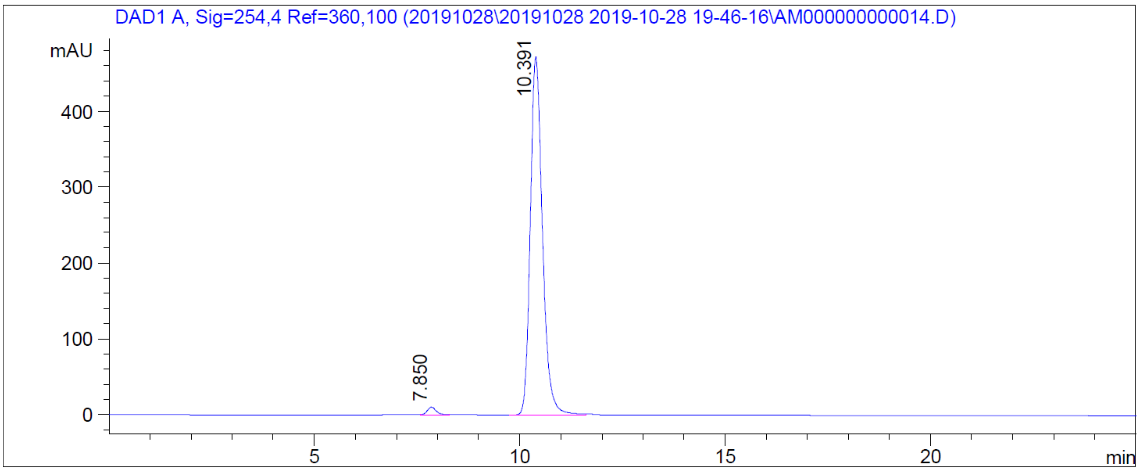

#	Meas. R	Respons	Respons	Height	Symmetr	Area %
###	7.850	162.190	1.637	2.162	1	1.637
###	10.391	9.746e3	98.363	97.838	1	98.363

*(2Z, 3E, 5E)-N-(p-Tolyl)-2-(p-tolylimino)-6-(p-(trifluoromethyl)phenyl)hexa-3,5-dienamide (**20b**).*

Purity: **99.7%**

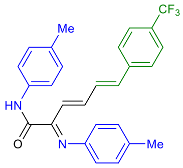

20b

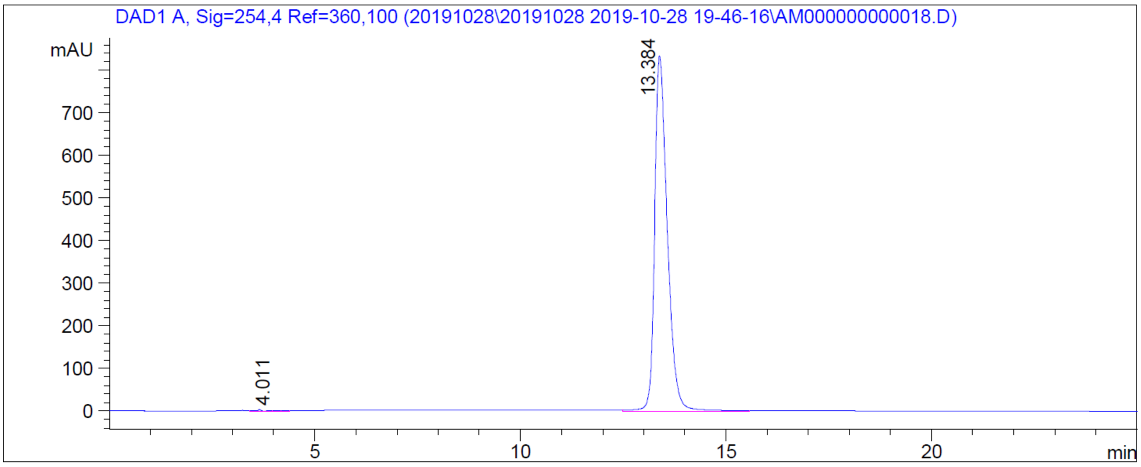

#	Meas. R	Respons	Respons	Height	Symmetr	Area %
###	3.653	23.748	0.132	0.395	2	0.132
###	3.900	7.659	0.043	0.129	1	0.043
###	4.011	10.024	0.056	0.115	0	0.056
###	4.289	8.702	0.048	0.116	1	0.048
###	13.384	1.791e4	99.721	99.245	1	99.721

(Z)-5-phenyl-1-(p-tolyl)-3-(p-tolylimino)-1,3-dihydro-2H-pyrrol-2-one (**21**).

Purity: **99.9%**

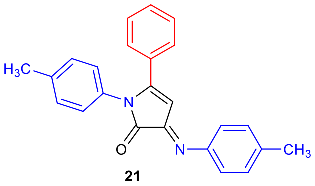

21

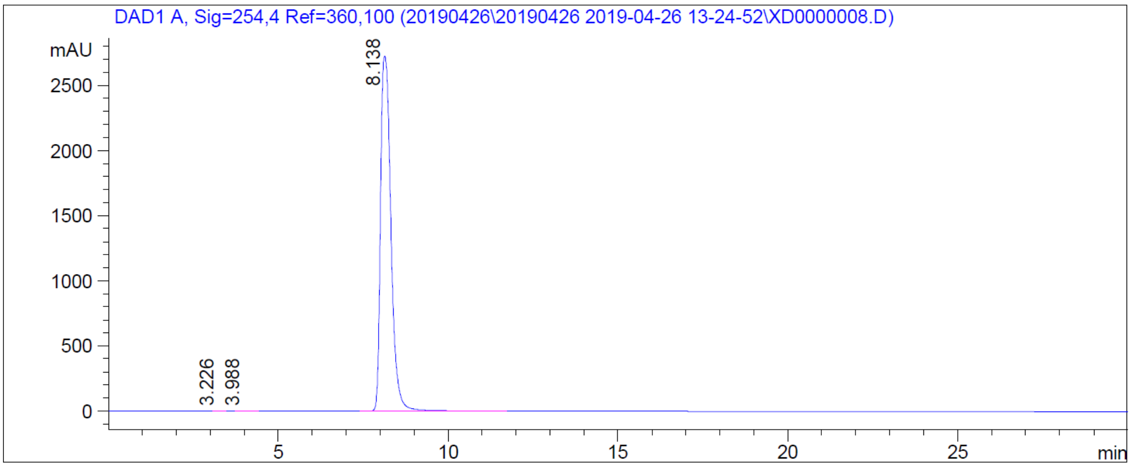

#	Meas. R	Respons	Respons	Height	Symmetr	Area %
###	3.226	15.179	0.026	0.042	1	0.026
###	3.988	15.607	0.027	0.021	0	0.027
###	8.138	5.774e4	99.947	99.937	1	99.947

4. Flow cytometric assays on A-549 cells at different exposure time after addition of compound 4b.

Non-treated cells. FL1 negative, FL3 negative.

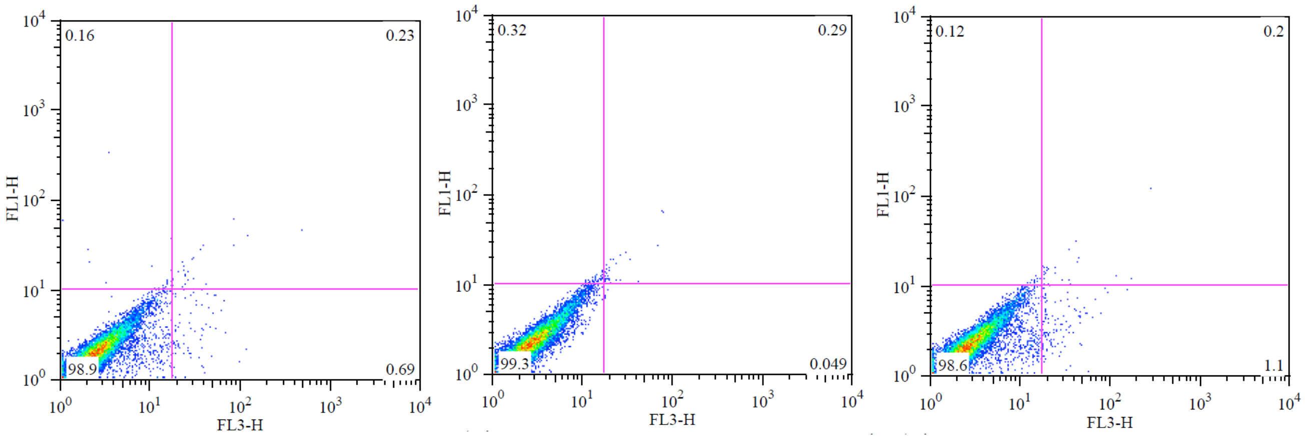

Necrotic cells, treated with Ethanol: FL1 positive, FL3 negative.

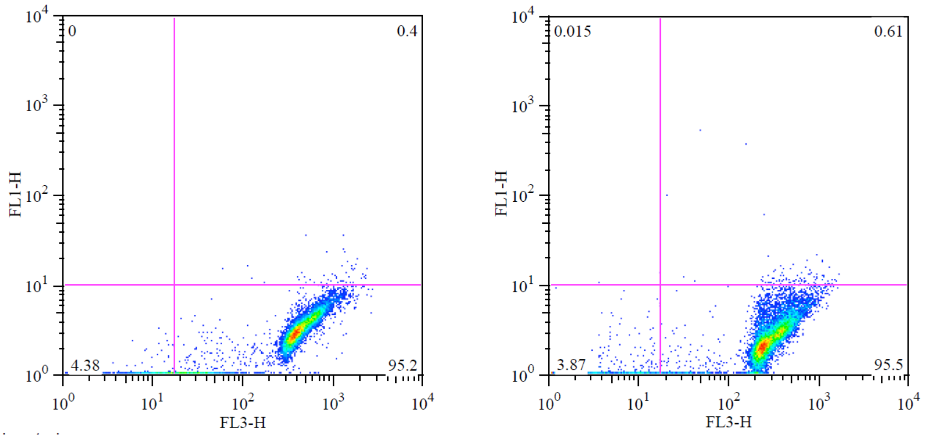

Early apoptotic cells, treated with 1 μM of Camptothecin for 24h: FL1 negative, FL3 positive.

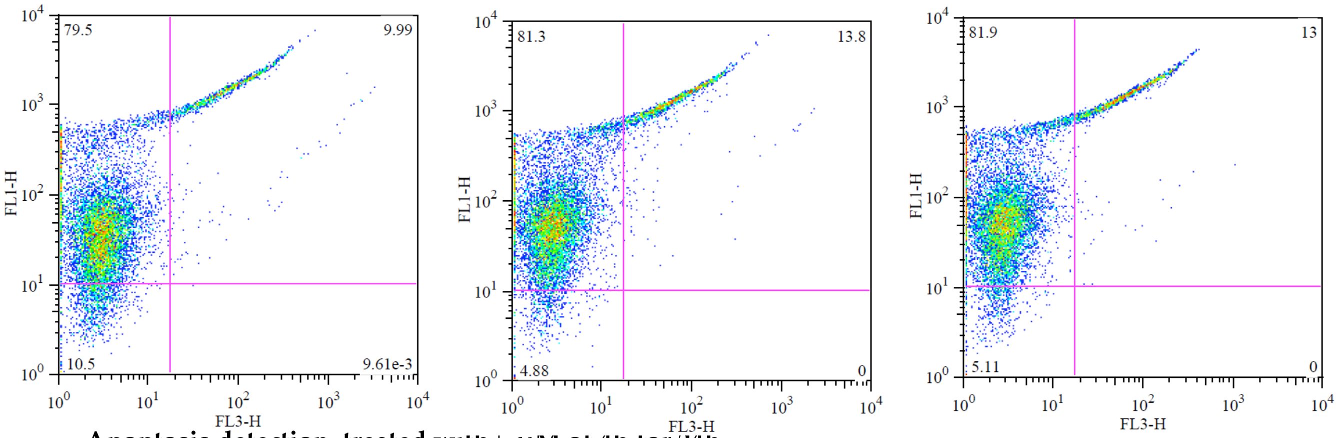

Apoptosis detection, treated with 5 μM of 4b for 24h.

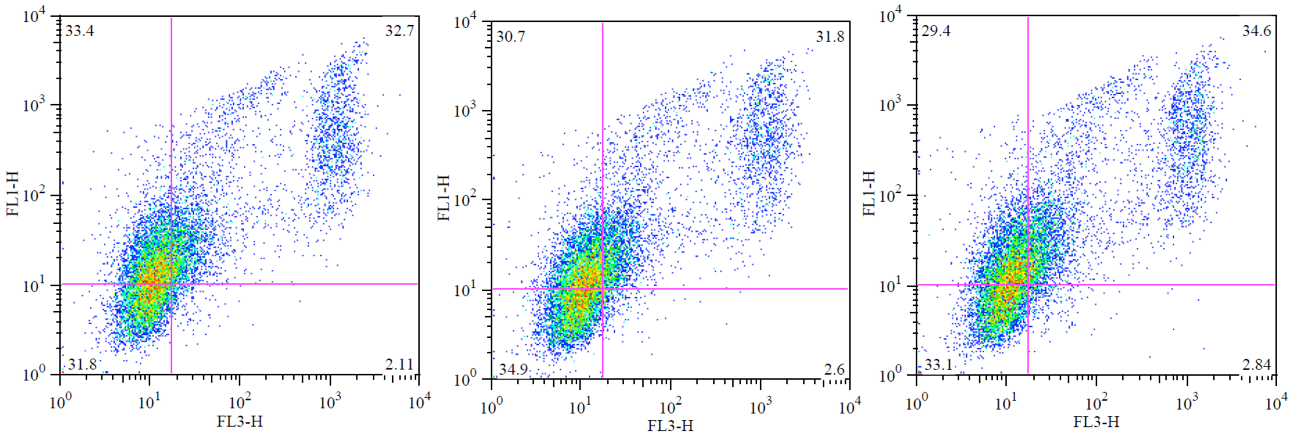

Apoptosis detection, treated with 5 μM of 4b for 24h.

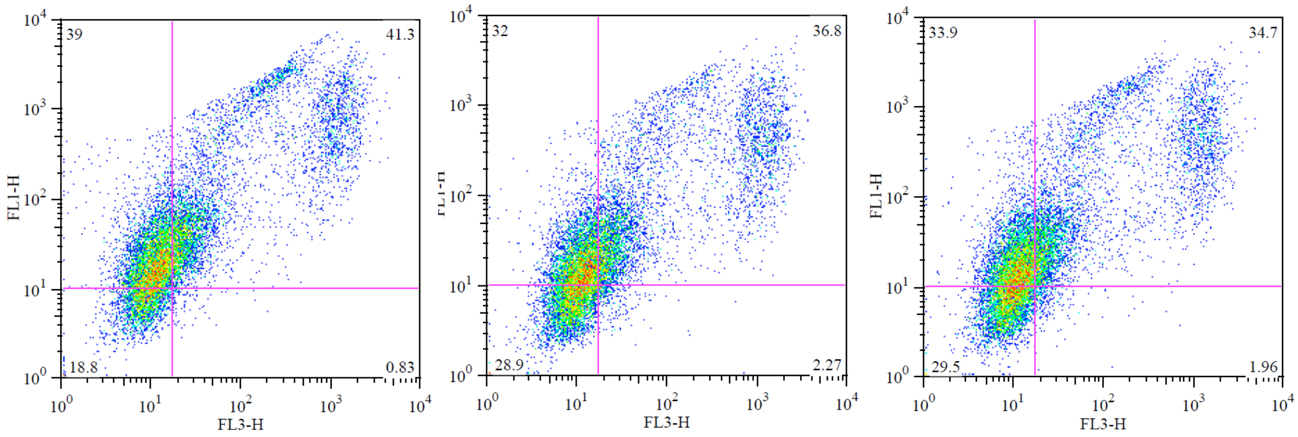

Apoptosis detection, treated with 5 μM of 4b for 24h.

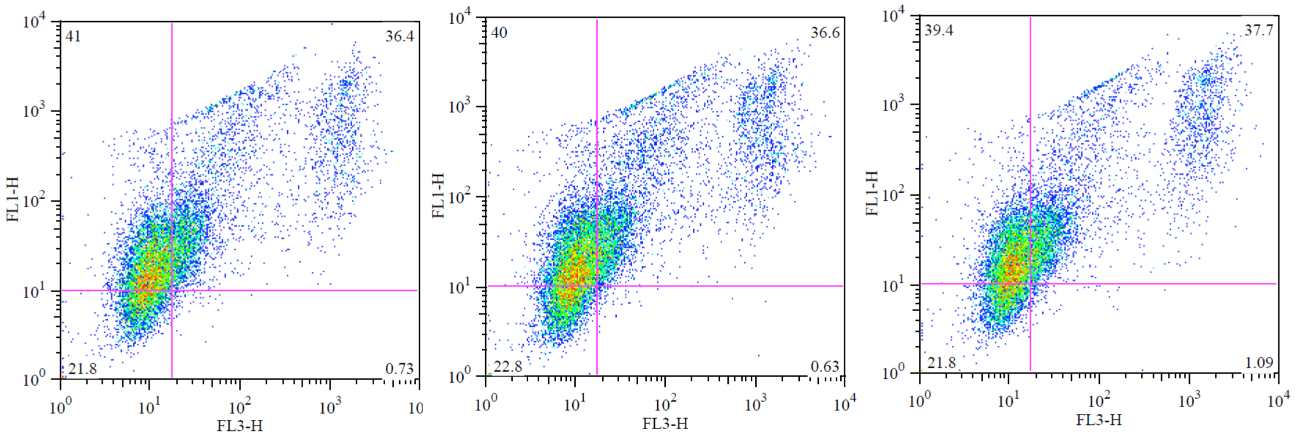

5. Visualization of cell growth and morphology of A-549 cells at different exposure time after addition of compound 4b.

Cell morphology visualization with X4 lens at different exposure times to compound **4b**. Scale bar: 300 μm.

a) A549 cells treated with 15 μM of **4b** at 24h.

b) A549 cells treated with 10 μM of **4b** at 24h.

c) A549 cells treated with 1 μM of **4b**.

d) A549 cells treated with 5 μM of **4b**.

e) A549 cells without **4b**.

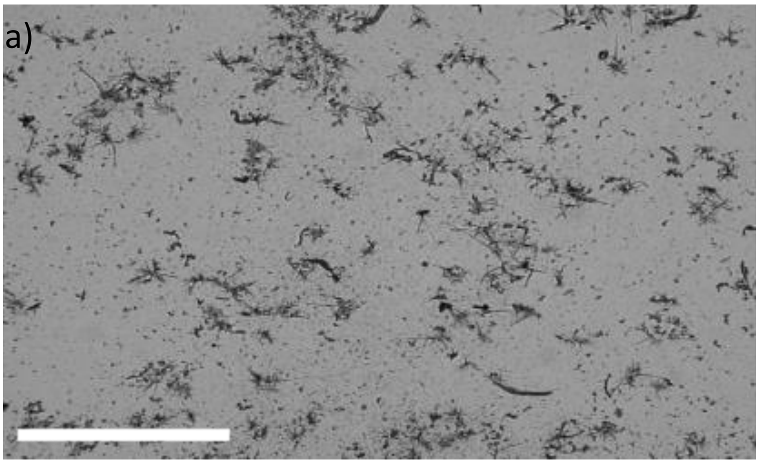

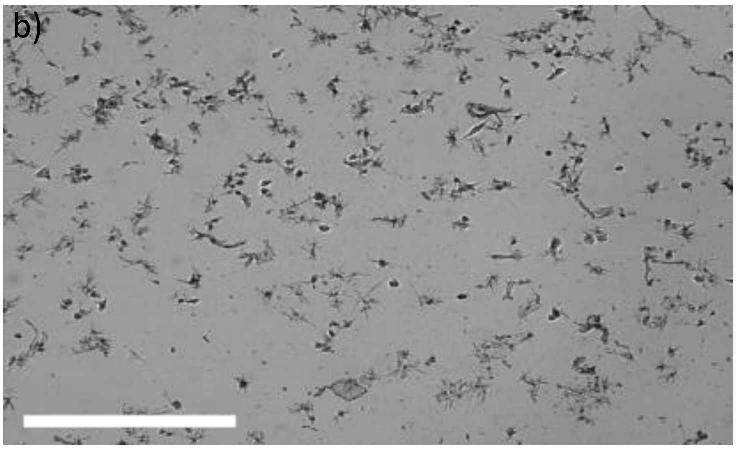

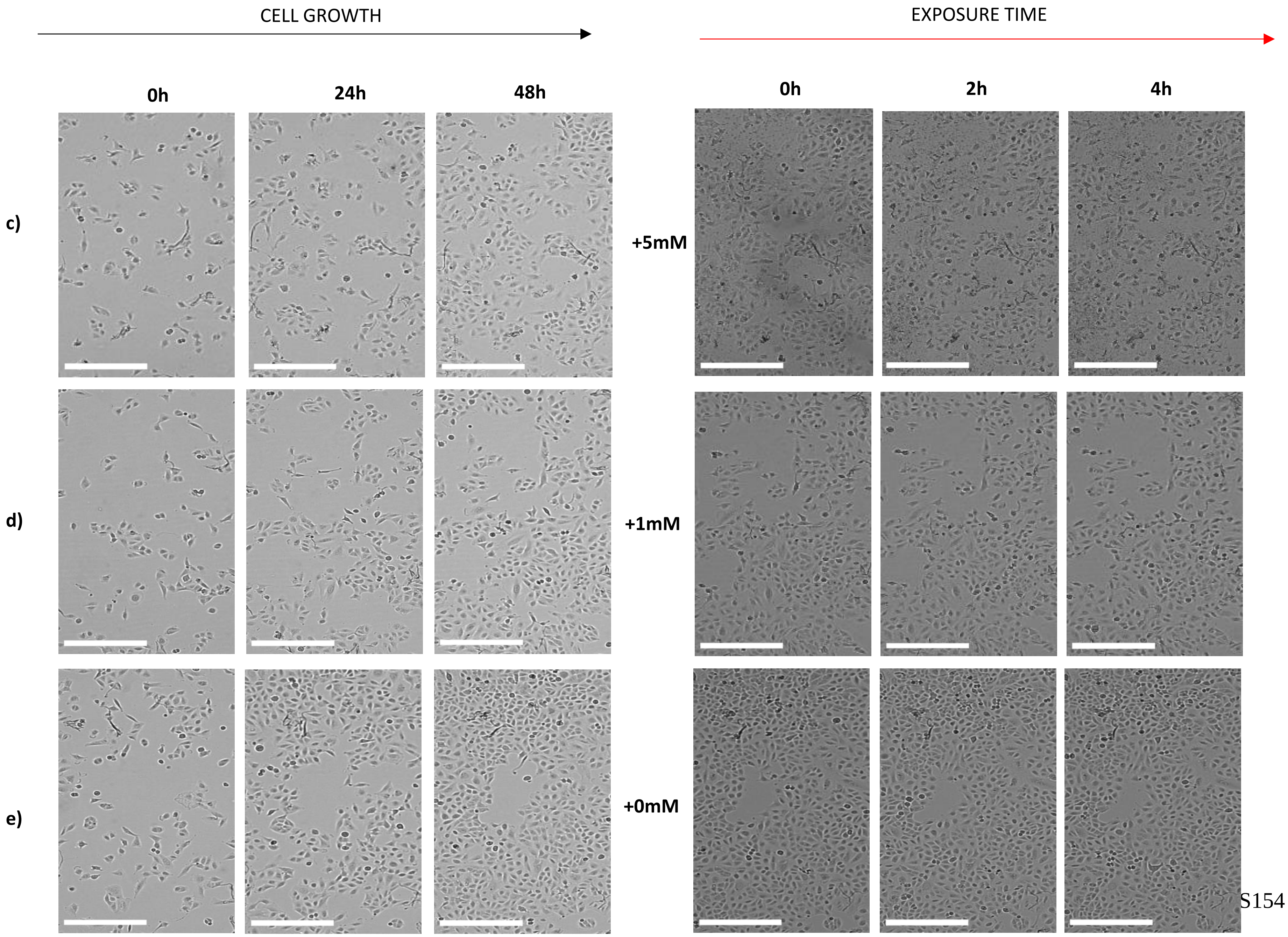
CELL GROWTH
EXPOSURE TIME
0h
24h
48h
0h
2h
4h
c)
+5mM
d)
+1mM
e)
+0mM

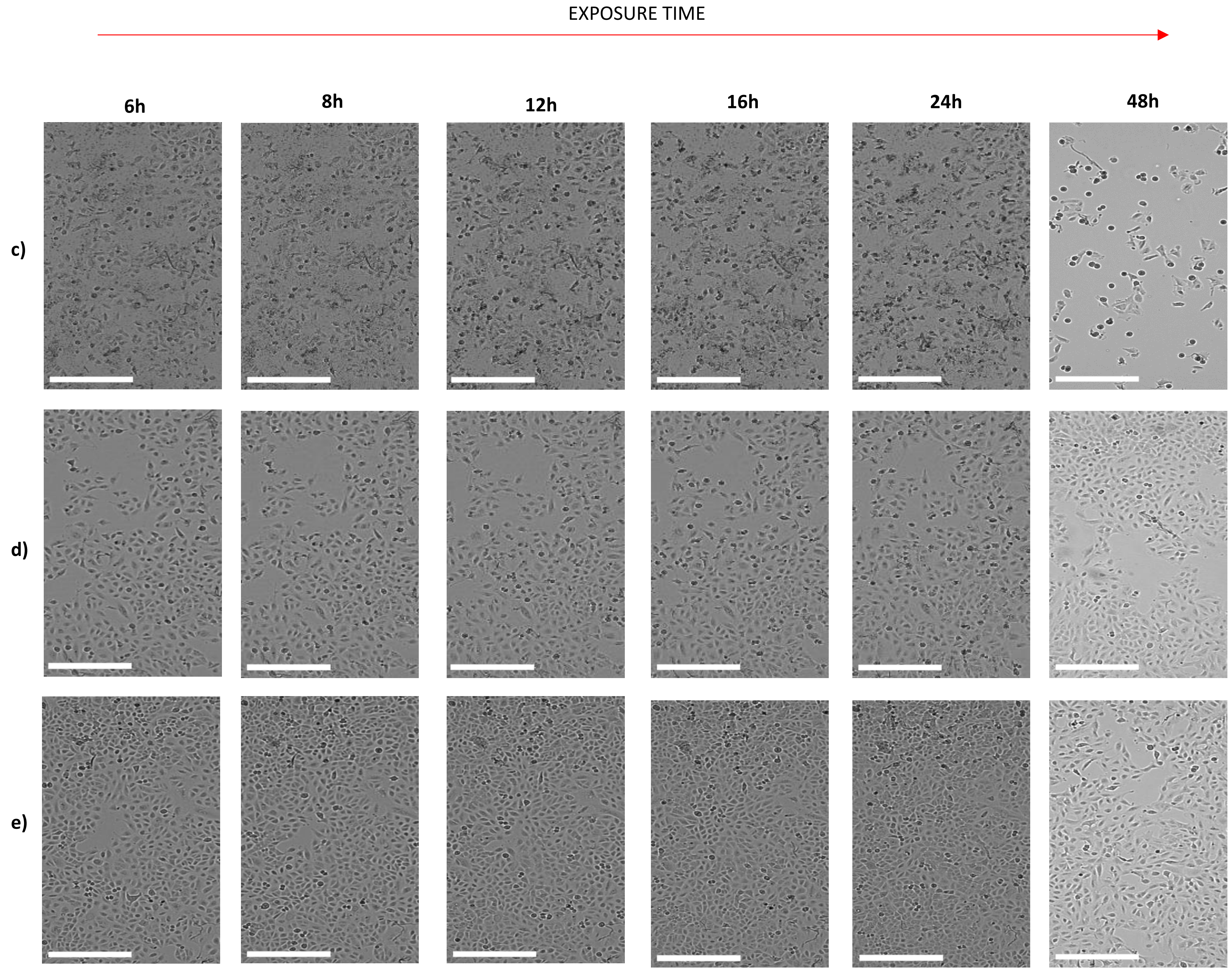
EXPOSURE TIME
6h
8h
12h
16h
24h
48h
c)
d)
e)

6. Calculation of Lipinski's rule of five and prediction of ADME properties.

In the following table some calculations had been develop to predict ADME parameters, and to determine the medicinal chemistry friendliness of our small molecules. Lipinski rules are used to evaluate if a compound is likely to be an orally active drug in humans. According to Lipinski's rule, an orally active drug has no more than one violation of the following criteria: MW ≤ 500, LogP ≤ 4.15, N or O atoms ≤ 10 and NH or OH atoms ≤ 5.[4] These calculations were performed using *SwissADME* program from the Swiss Institute of Bioinformatics (http://www.swissadme.ch/).

Cpd.	MW (g/mol)	LogP	HBD	HBA	RB	TPSA (Å^2)	Lipinski (Violations)	GI Abs.	BBB Permeation
**4a**	278.35	3.26	1	1	3	32.34	Yes (0)	High	Yes
**4b**	354.44	4.41	1	1	4	32.34	Yes (0)	High	Yes
**4c**	368.47	4.73	1	1	4	32.34	Yes (1)	High	Yes
**4d**	368.47	4.74	1	1	4	32.34	Yes (1)	High	Yes
**4e**	368.47	4.74	1	1	4	32.34	Yes (1)	High	Yes
**4f**	372.43	4.71	1	2	4	32.34	Yes (1)	High	Yes
**4g**	399.44	3.64	1	3	5	78.16	Yes (0)	High	No
**4h**	399.44	3.64	1	3	5	78.16	Yes (0)	High	No
**4i**	422.44	5.43	1	4	5	32.34	Yes (1)	Low	No
**4j**	407.51	4.58	1	1	4	37.27	Yes (0)	High	Yes
**4k**	344.41	3.76	1	2	4	45.48	Yes (0)	High	Yes
**4l**	360.47	4.43	1	1	4	60.58	Yes (0)	High	Yes
**4m**	404.50	5.30	1	1	4	32.34	Yes (0)	High	Yes
**4n**	291.39	4.11	1	1	3	29.10	Yes (0)	High	Yes
**4o**	320.43	4.05	1	1	4	32.34	Yes (0)	High	Yes
**4p**	334.45	4.37	1	1	5	32.34	Yes (0)	High	Yes
**4q**	360.49	4.78	1	1	4	32.34	Yes (1)	High	Yes
**4r**	380.48	4.97	1	1	5	32.34	Yes (1)	High	Yes
**4s**	350.41	3.37	1	3	6	58.64	Yes (0)	High	Yes
**4t**	444.40	5.84	1	6	4	32.34	Yes (1)	Low	No
**4u**	346.35	4.11	1	1	4	32.34	Yes (0)	High	Yes
**4v**	414.43	3.52	1	4	8	77.68	Yes (0)	High	No
**4w**	478.52	4.85	1	2	6	59.22	Yes (0)	High	Yes
**5a**	413.47	3.94	1	3	5	78.16	Yes (0)	High	No
**5b**	489.56	5.03	1	3	7	78.16	Yes (1)	High	No
**5c**	471.50	3.90	1	5	8	104.5	Yes (0)	High	No
**5d**	414.43	3.59	1	4	8	77.68	Yes (0)	High	No
**5e**	478.52	5.02	1	2	6	59.22	Yes (0)	High	Yes
**6a**	386.44	3.72	1	3	6	50.80	Yes (0)	High	Yes
**6b**	431.44	2.98	1	5	7	96.62	Yes (0)	High	No
**6c**	460.46	3.06	1	6	11	96.14	Yes (0)	High	No
**7**	440.28	4.26	1	3	5	78.16	Yes (1)	High	No

*LogP= Partition Coefficient, Lipophilicity.; HBD= Num. H-bond donors.; HBA= Num. H-bond acceptors; RB= Num. rotatable bonds.; GI Abs= Gastro-Intestinal Absortion.; BBB Permeation= Blood-Brain-Barrier Permeation.

[4] Lipinski, C.A.; Lombardo, F.; Dominy, B. W.; Feeney, P. J. Experimental and computational approaches to estimate solubility and permeability in drug discovery and development settings. *Adv. Drug. Deliv. Rev.* **2001**, *64*, 4-17. DOI: 10.1016/s0169-409x(00)00129-0.

Cpd.	MW (g/mol)	LogP	HBD	HBA	RB	TPSA (Å^2)	Lipinski (Violations)	GI Abs.	BBB Permeation
**8a**	529.18	4.42	1	3	5	78.16	No (2)	High	No
**8b**	552.18	6.07	1	4	5	32.34	No (2)	Low	No
**9**	440.28	4.29	1	3	5	78.16	Yes (1)	High	No
**10**	407.37	3.84	1	5	5	78.16	Yes (0)	High	No
**11a**	386.29	4.61	1	7	5	32.34	Yes (0)	Low	No
**11b**	507.38	5.13	1	9	7	78.16	No (2)	Low	No
**12**	473.48	3.90	1	5	5	103.9	Yes (0)	Low	No
**16**	364.44	3.69	1	3	6	58.64	Yes (0)	High	Yes
**17**	366.45	3.60	1	3	6	58.64	Yes (0)	High	Yes
**18a**	348.36	4.12	1	4	4	32.34	Yes (0)	High	Yes
**18b**	430.48	3.69	1	4	9	77.68	Yes (0)	High	No
**18c**	388.46	3.72	1	3	6	50.80	Yes (0)	High	Yes
**19**	456.51	4.04	1	4	9	77.68	Yes (0)	High	No
**20a**	380.48	5.52	1	2	7	41.46	Yes (1)	High	No
**20b**	448.48	6.56	1	5	8	41.46	Yes (1)	Low	No
**21**	352.43	4.69	0	2	3	32.67	Yes (0)	High	Yes

*LogP= Partition Coefficient, Lipophilicity.; HBD= Num. H-bond donors.; HBA= Num. H-bond acceptors; RB= Num. rotatable bonds.; GI Abs= Gastro-Intestinal Absortion.; BBB Permeation= Blood-Brain-Barrier Permeation.
